# FloralArea: AI-powered algorithm for automated calculation of floral area from flower images to support plant and pollinator research

**DOI:** 10.1371/journal.pone.0332165

**Published:** 2025-09-12

**Authors:** Edward I. Amoah, Khayri White, Harland M. Patch, Christina M. Grozinger

**Affiliations:** 1 Intercollege Graduate Degree Program in Ecology, Huck Institutes of the Life Sciences, Penn State University, University Park, Pennsylvania, United States of America; 2 Department of Entomology, Center for Pollinator Research, Huck Institutes of the Life Sciences, Penn State University, University Park, Pennsylvania, United States of America; 3 Undergraduate Degree Program in Computer Engineering, Department of Electrical Engineering and Computer Science, Howard University, Howard, Washington District of Columbia, United States of America; National Institute of Agricultural Research - INRA, MOROCCO

## Abstract

Floral area is a major predictor of the attractiveness of a flowering plant for pollinators, yet the measurement of floral area is time-consuming and inconsistent across studies. Here, we developed an AI-powered algorithm, FloralArea, to automate floral area measurement from an image. The FloralArea algorithm has two main components: an object segmentation module and an area estimation module. The object segmentation module extracts the pixels of flowers and the reference object in an image. The area estimation module predicts floral area based on the ratio between flower and reference object pixels. We fine-tuned two YOLOv8 segmentation models for flower and reference object segmentation. The flower segmentation model achieved moderate precision, recall, mAP0.5, and mAP0.5-0.95 of 0.794, 0.68, 0.741, and 0.455 on the test dataset, while the reference object model achieved an impressive performance of 0.907, 0.940, 0.933, and 0.832. We evaluated FloralArea using 75 images of flowering plants. We used ImageJ to calculate the actual floral area for all the images and compared them with the predicted floral area from FloralArea. The predicted floral area correlated well with the measured floral area with a coefficient of determination (R^2^) of 0.93 and a root mean square error of 20.58 cm^2^. The FloralArea algorithm reduced the time it takes to calculate floral area from an image by 99.24% compared with traditional methods with image processing tools like ImageJ. By streamlining floral area estimation, the FloralArea algorithm provides a scalable, efficient, consistent, and accessible tool for researchers, particularly to aid in assessing plant attractiveness to different pollinator groups.

## Introduction

Pollinators provide ecosystem services critical for food security and ecosystem function, but populations are in decline in many parts of the world [1–4]. Pollinators forage on flowering plants for pollen and nectar, which provide pollinators with proteins, lipids, carbohydrates, and micronutrients [5,6]. While foraging, pollinators transfer pollen from one flower to another, thereby supporting plant reproduction [7]. Loss of diversity and abundance of flowering plants due to land use change is a major driver of pollinator declines [8], and mounting evidence indicates that climate change can negatively influence flowering and floral resource production [9–11]. Incorporating pollinator-attractive flowering plant species into urban, agricultural, and natural landscapes can increase pollinator abundance and diversity [5,12]. However, identifying the most attractive and beneficial flowering plant species and cultivars for diverse pollinator groups is challenging and time-consuming [13].

The nutritional demands of different species of pollinators vary, and flowering plants have evolved to have substantial differences in floral traits and nutritional quality to attract and support different pollinator groups [14]. Studies have shown that different taxonomic groups of pollinators tend to be attracted to different suites of floral nutritional traits (e.g., pollen with different ratios of protein versus lipid), morphological traits (color, shape), or floral volatiles [15,16]. However, these “pollinator syndromes” do not consistently predict attraction across plant species and plant-pollinator communities [17,18]. Moreover, when breeding ornamental flowering plants, floral traits and nutritional quality may become uncoupled, such that some cultivars with attractive flowers may no longer support pollinators’ nutritional needs [19]. For researchers and breeders to identify plant stocks that can support diverse communities of pollinators, there is a need to develop methodologies that can rapidly evaluate the attractiveness of flowering plants to different pollinator groups.

Numerous methodologies can be used to evaluate the attractiveness of a flowering plant to different taxonomic groups of pollinators [13]. These methodologies include monitoring the number and types of pollinator visitors to a focal plant species and molecular analysis of pollen gathered by pollinators [13]. Monitoring pollinator visitors is the most commonly used method to evaluate the attractiveness of flowering plants to pollinators [15]. However, this method is time-consuming and resource-intensive, and the data collection methods are often inconsistent across studies [15]. Recently, Erickson et al. (2022) developed a protocol that requires only a short (10 min) observation to quantify the attractiveness of focal plants [15]. The protocol can effectively rank focal plant stocks according to their attractiveness to different taxonomic groups of pollinators (i.e., bee, fly, butterfly). Large-scale implementation of this protocol would allow researchers, conservationists, gardeners, and plant breeders to evaluate plant stocks and cultivars for pollinator attractiveness efficiently.

Floral area is a major driver of pollinators’ attraction to flowering plants [20–22]. Most researchers measure the floral area in a three-step protocol [23–25]. First, a photographic image of a flower and reference object of known size (such as a measuring stick or caliper) is captured in the field. Second, the boundaries of the flowers are delineated to extract the flower pixels from the image using image processing tools such as Adobe Photoshop or ImageJ [25,26]. Subsequently, the floral area of the flowering plant is calculated from the pixels of the flower by using the reference object as a scale. This process requires several steps and takes significant handling time for each image.

Computer vision and artificial intelligence (AI) enabled algorithms can simplify floral area measurement by automating flower and reference object pixel extraction from an image. Previous studies have used computer vision-AI to identify individual flowers in images of vegetation patches and distinguish between plant species based on floral photos [27, 28]. However, until now, this approach has not been used for floral area measurement from images. The objective of this study is to develop an AI-powered computer vision algorithm to automate floral area measurement from an image of flowering plants. This study has three sub-objectives. First, we present the design of FloralArea, a modular AI-powered algorithm that integrates instance segmentation and photogrammetry to automate floral area measurement from an image. Second, we fine-tune YOLOv8 instance segmentation models to detect flowers and reference objects with high accuracy. Third, we evaluate the accuracy, robustness and time-efficiency of the FloralArea algorithm against manual measurements with traditional methods (i.e., ImageJ) across multiple flowers with diverse floral traits and imaging conditions. The methods and results sections of this study are structured around the sub-objectives of this study.

## Materials and methods

The materials and methods are presented in three sub-sections, each corresponding to one of the sub-objectives of this study. Section 1 describes the modular design of the FloralArea algorithm. Section 2 details the fine-tuning of the YOLOv8 models for instance segmentation models in the FloralArea algorithm. Section 3 presents the evaluation of the FloralArea algorithm’s accuracy, robustness and time-efficiency, as well as benchmarking its performance against manual measurements using traditional image processing tools (i.e., ImageJ).

### FloralArea algorithm design

**Fig 1:**
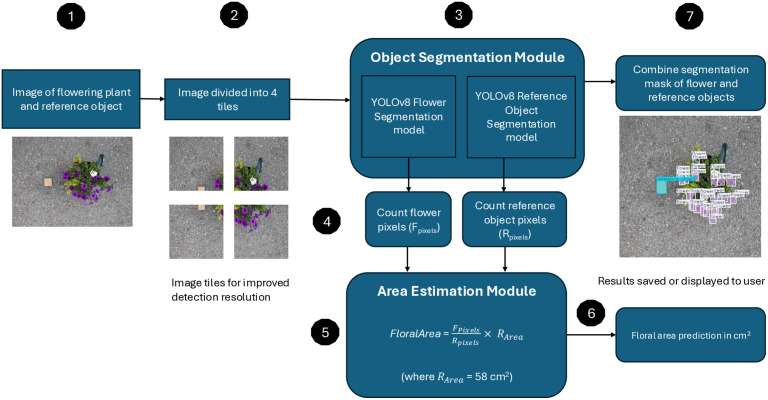
Global workflow architecture of the FloralArea algorithm. The FloralArea algorithm estimates floral area from an image of a flowering plant and a reference object of known size (1). To improve detection resolution, the input image is divided into four tiles (2), which are processed through the object segmentation module (3). This module includes two YOLOv8 instance segmentation models: one fine-tuned to detect flowers, and the other to detect the reference object. The resulting segmentation masks are used to count flower pixels (*F*_*Pixels*_) and reference object pixels (*R*_*Pixels*_) (4). These counts are passed to the area estimation module (5), which applies the formula $\text{FloralArea}\;=\;\frac{F_{Pixels}}{R_{Pixels}}\times R_{Area}$, where R_Area_ = 58 cm^2^. The estimated floral area (6) is returned to the user, and segmentation mask for both flower and reference object can be optionally combined and visualized (7).

The overall structure of the FloralArea algorithm is illustrated in the global workflow architecture (Fig 1). The algorithm consists of two main modules: the object segmentation module, which extracts the number of pixels belonging to flowers and a reference object, and the area estimation module, which uses pixel counts ratio to calculate floral area.

The object segmentation module automates the image processing typically performed manually using tools like Adobe Photoshop or ImageJ [25]. This module uses two fine-tuned YOLOv8 instance segmentation models from Ultralytics [29]: one fine-tuned to segment flower pixels and the other fine-tuned to segment reference object pixels. YOLOv8, the eighth version of the You Only Look Once (YOLO) framework [29,30], is widely used for real-time object detection across domain such as robotics, medical imaging, autonomous vehicles, and agriculture [29]. We selected YOLOv8 because it achieves a strong balance between detection accuracy, inference speed, and ease of deployment suitable for ecologist with minimal technical background. Comparative studies have shown that although recent YOLO models like YOLOv9 can achieve higher precision and recall, the YOLOv8 models offer faster training and inference time compared to YOLOv9 models [31,32]. We also considered the Segment Anything Model (SAM), which can offer a prompt-based zero-shot segmentation [33]. However, while SAM excels at general-purpose segmentation across diverse domains, it requires guided prompting to achieve highly accurate segmentation masks [34,35]. Additionally, we considered other deep learning models commonly used for segmentation tasks like Mask R-CNN [36–38]; however, studies have shown that YOLOv8 models outperform Mask R-CNN for the segmentation of objects in field environments while maintaining superior inference time [39].

To improve segmentation accuracy, the FloralArea algorithm divides the input image into four tiles (Fig 1, step 2) and processes each tile independently using the YOLOv8 models (Fig 1, step 3). The confidence threshold for detection was set to 0.5 to ensure only high-confidence flower and reference object detections were included for the flower and reference object pixel counts (Fig 1, step 4). The calculated flower pixels (*F*_*Pixels*_) and the reference object pixels (*R*_*Pixels*_) are passed to the area estimation module.

The Area Estimation module uses a pixel-ratio based formula grounded in the principle of two-dimensional (2D) photogrammetry [40,41] to estimate floral area (Fig 1, step 5). Specifically, the flower area is computed as the ratio of flower pixels to reference object pixels, multiplied by the known area of the reference object (Eqn 1):


$$FloralArea=\;\frac{F_{Pixels}}{R_{Pixels}}\times \;R_{Area}$$ (1)

In this equation, floral area is estimated in square centimeters, *F*_*Pixels*_ is the number of segmented flower pixels, *R*_*Pixels*_ is the number of segmented reference object pixels, and *R*_*Area*_ is 58 cm^2^ (i.e., the known size of the reference object). For this study, we used a square piece of brown cardboard (7.6 cm × 7.6 cm) as the reference object. This material was chosen because it is low-cost, non-reflective, and easy for future users to replicate. The calculated area and a combined mask for both flower and reference object segmentation can be visualized or saved for the user (Fig 1, step 6 and 7).

The modular design of the FloralArea algorithm with separate segmentation models for flowers and the reference object makes the algorithm flexible and extensible. For instance, the flower segmentation model can be fine-tuned to detect different objects, such as leaves or fruit, which will allow the same algorithm to be used for leaf or fruit area calculation. Likewise, the reference object segmentation model can be fine-tuned to detect different reference objects, such as white paper or a ruler, without affecting the flower segmentation model performance.

### Segmentation model development

**Fig 2:**
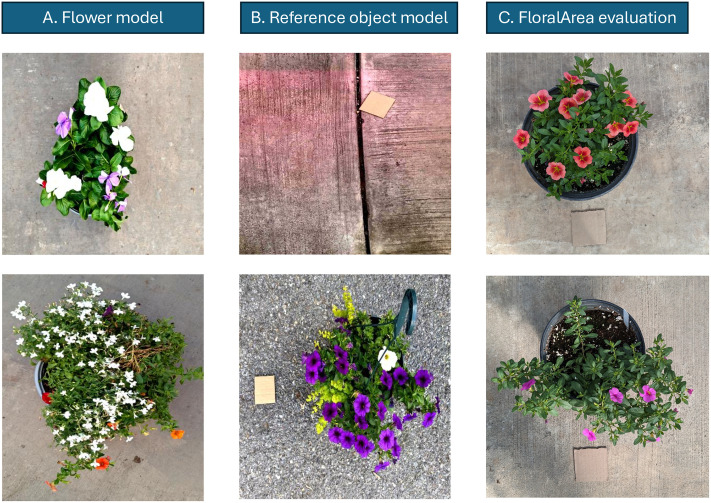
Sample image datasets. **(A)** Examples of the images used to fine-tune the flower segmentation YOLOv8 model. **(B)** Examples of the images used to fine-tune the reference object segmentation YOLOv8 model. **(C)** Examples of the images used to evaluate the accuracy and time-efficiency of the FloralArea algorithm compared with traditional image analysis tools (ImageJ).

We fine-tuned two segmentation models. One is for flower segmentation, and the second is for reference object segmentation. The process of fine-tuning a segmentation model has three steps. First, we took images of flowering plants from garden centers and supermarkets in State College, Pennsylvania. Second, we annotated the images on the Roboflow platform (v1.119) [42]. We used the polygon tool to draw a boundary around flowers and reference objects in the selected images. We also annotated leaves, stems, buds, dead flowers, and some objects in the background on a subset of images to help the model avoid confusing these objects as flowers or reference objects. The third step is model training. We divided the dataset into 70% for training, 20% for validation, and 10% for testing. The dataset for the flower segmentation model had 379 images. The dataset for reference object segmentation model had 204 images. Examples of the image datasets used for flower and reference object model fine-tuning as well as for the evaluation of the FloralArea algorithm can be found in Fig 2. The segmentation models were fine-tuned from the pre-trained YOLOv8 models provided by Ultralytics (https://www.ultralytics.com/). The models were fine-tuned with the default Ultralytics hyperparameters for YOLOv8 (Table 1).

**Table 1:** Key parameters set for training models.

Model	Flower and reference object
Pre-trained model	YOLOv8m
Epoch	100
Momentum	0.937
Initial learning rate	0.01
Weight decay	0.0005
Optimizer	Adam
Input image size	640 x 640
Tiling	2x2
Auto-contrast	Applied
Auto-Orient	Applied

To evaluate the performance of the fine-tuned models, we used precision, recall, maAP0.5, and maAP:0.5–0.95. *Precision* is the ratio between the true positive detections and the number of all detected. *Recall* is the ratio of the number of positive samples correctly predicted by the model to the number of positive samples that appeared. The *average precision* (AP) equals the area under the precision-recall curve. *Mean average precision* (mAP) is the result obtained by the weighted average of AP values of the sample categories, which is used to measure the detection performance of the model in all categories [43].

### FloralArea algorithm evaluation

We evaluated the FloralArea algorithm by comparing floral area estimation from the algorithm with manual floral area measurements using traditional image processing tool (ImageJ). The accuracy, robustness and time-efficiency of the FloralArea algorithm was evaluate with seventy-five flower images (Fig 2C). These images were independent of those used to fine-tune the segmentation models described earlier. The evaluation image dataset was collected from 23 flowering plants grown in pots in the Penn State College of Agricultural Sciences greenhouses (University Park, Pennsylvania). For each flowerpot, images were taken at 60 cm, 80 cm, and 100 cm above the ground. The floral area of each image was predicted using the FloralArea algorithm. Following a standardized protocol, ground-truth measurements of floral area were also determined using the ImageJ software [26].

### Direct ImageJ measurement protocol

ImageJ software (v1.54i) was obtained from the National Institutes of Health [26]. To begin measurement, a flower image from the evaluation dataset is opened, and a line tool is used along the length of the reference object (7.62 cm) to set the scale of the image (S1 Fig). This sets the pixel-to-centimeter ratio according to the reference object to accurately measure the floral area. Next, the free form drawing tool is used to draw a boundary around each flower or group of flowers, and the area of each flower is measured and added to a data table using the ROI Manager tool. The total floral area of flowers in the images was calculated as the sum of all the area measurements in the ROI Manager tool (S1 Fig).

### Evaluating the accuracy of the FloralArea algorithm

The predicted and measured floral areas were compared using correlation analysis using the Python Scikit-Learn library (v1.5.0). The regression model’s coefficient of determination (R^2^) and root mean squared error (RMSE) were the primary metrics for evaluating algorithm accuracy.

### Evaluating the effect of variation in flower type and image capture on the accuracy of the FloralArea algorithm

We evaluated the robustness of the algorithm’s performance with varying flower colors, petal display, and image capture distance categories. We developed a metric (termed “bias”) to measure how closely the predicted floral area aligns with the measured floral area for the various categories (Eqn 2).


$$\begin{array}{c} bias\;=\frac{1}{n}\sum {\mathrm{\backslash nolimits}}_{i=1}^{n}\left(predict-measure\right)\; \end{array}$$ (2)

Flower images were subjectively organized into five color categories (details in S1 Table), three petal display categories, and three image capture distance categories (details in S2 Table). The color categories were T1: red, orange, and yellow mixed flowers on a single plant; T2: pinkish-purple flowers; T3: pink flowers; T4: red flowers; T5: purple flowers. The petal display categories were subjectively classified C1: compound petals, C2: continuous petals, C3: clumps of loose petals. The image capture distance categories were 60 cm, 80 cm, and 100. A one-way ANOVA was conducted to test for statistical differences in bias across the flower color, petal display, and image capture distance categories.

### Evaluating the efficiency of FloralArea versus direct measurement

We quantified the time-efficiency of the FloralArea algorithm by comparing the time required to analyze images with that of traditional methods. We recorded the time taken to estimate the floral area for ten images (n = 10) using the ImageJ protocol. We compared it to the processing time for the same images using the FloralArea algorithm on an 18GB-RAM 12-core CPU Apple M3 Pro chip laptop computer.

## Results

The results are presented in two sections. Section 1 presents the results of fine-tuning the YOLOv8 segmentation models for flowers and reference objects. Section 2 presents the results for the accuracy, robustness and time-efficiency of the FloralArea algorithm.

### Segmentation model evaluation

#### Flower segmentation model.

The flower segmentation model achieved a precision of 0.794 and a recall of 0.68 (Table 2). The mean average precision (mAP) at a 0.5 Intersection over Union (IoU) threshold was 0.741, and the mAP across IoU thresholds from 0.5 to 0.95 (mAP:0.5–0.95) was 0.455 (Table 2). These results reflect moderate detection accuracy for the flower segmentation model.

**Table 2:** Results for evaluating the flower and reference object segmentation model with the test dataset. The flower segmentation model achieved moderate performance across all metrics (i.e., precision, recall, mAP0.5, mAP:0.5-0.95), however the reference object segmentation model achieved superior performance across all evaluation metrics.

Model	Flower	Reference object
Precision	0.794	0.907
Recall	0.68	0.940
mAP0.5	0.741	0.933
mAP:0.5–0.95	0.455	0.832

#### Reference object segmentation model.

The reference object segmentation model demonstrated superior performance across all metrics. It achieved a precision of 0.907 and a recall of 0.94 (Table 2), indicating nearly perfect detection with minimal false positives. The mAP at a 0.5 IoU threshold was 0.933, and the mAP:0.5–0.95 was 0.832, showcasing the model’s robustness and high accuracy in identifying reference objects at varying levels of overlap.

### FloralArea algorithm evaluation

#### Accuracy of the FloralArea algorithm.

**Fig 3:**
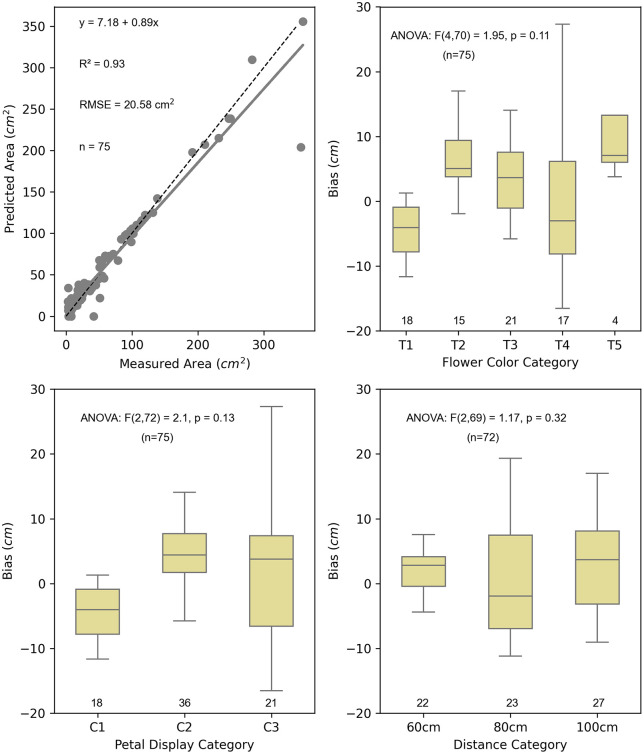
FloralArea algorithm regression analysis evaluation. **(A)** Evaluation of the accuracy of the FloralArea algorithm with regression analysis. Seventy-five images were analyzed using FloralArea and traditional manual annotation using ImageJ software. The predicted floral area (cm^2^) is on the y-axis, and the measured floral area (cm^2^) is on the x-axis. The two methods resulted in very similar measurements, with the regression model showing an r^2^ = 0.93 and RMSE of 20.58 cm^2^ for predicted vs. measured floral area. **(B)** Evaluation of bias due to color. Color categories are denoted as T1-T5; see Supplementary S1 Table for more details. **(C)** Evaluation of bias due to petal display categories denoted as C1-C3; see Supplementary S2 Table for more details. **(D)** Evaluation of bias due to image camera distance from the ground at 60 cm, 80 cm, and 100 cm. The flower sample size in each category is displayed on the x-axis. ANOVA results for each category indicate no statistically significant differences in bias across the tested categories (α = 0.05).

Regression analysis results indicate that the predicted (using the FloralArea algorithm) and measured floral area (using ImageJ) values are comparable (Fig 3A). The coefficient of determination for the regression line is 0.93, and the root mean square error (RMSE) of the FloralArea algorithm was 20.58 cm^2^.

#### Evaluating the effect of variation in flower type and image capture on the accuracy of the FloralArea algorithm.

The results indicate that the FloralArea algorithm provides robust and unbiased floral measurements irrespective of the flower color, petal display, or image capture distance category. A one-way ANOVA revealed no statistically significant difference in mean bias between the flower color categories (ANOVA F4,70 = 1.95, p = 0.11) (Fig 3B), petal display categories (ANOVA F2,72 = 2.10, p = 0.13) (Fig 3C), or image capture distance categories (ANOVA F2,69 = 1.17, p = 0.32) (Fig 3D).

#### Efficiency of using the FloralArea algorithm.

The FloralArea algorithm decreases the time required to calculate floral area from an image by 99.24%. On average, it takes 232.37 seconds or 3.87 minutes to calculate the floral area from an image using ImageJ. In contrast, the FloralArea algorithm can take about 1.76 seconds to estimate the floral area of an image on an 18GB-RAM 12-core CPU Apple M3 Pro chip laptop computer.

## Discussion

### Metrics for accuracy and bias of FloralArea

The FloralArea algorithm improves automates floral area measurement, addressing critical limitations of traditional manual methods. Our study demonstrates that FloralArea is a robust and time-efficient tool for accurate floral area estimation, achieving a high coefficient of determination (R² = 0.93), low root mean squared error (RMSE = 20.58 cm²), and time-efficiency of 99.24% compared to floral area measurement from an image using traditional image processing tool (ImageJ). The study also demonstrates that the FloralArea algorithm can provide unbiased floral area estimation for flowers with different colors, petal display categories, and image capture distances. These metrics validate the algorithm’s ability to offer unbiased measurements efficiently with remarkable accuracy and minimal error.

### Segmentation model performance and enhancements

The FloralArea algorithm’s performance depends on the accuracy of the segmentation models employed to delineate and extract flower pixels from images. The YOLOv8 segmentation models, fine-tuned for flowers and reference objects, performed at different levels of accuracy. The flower segmentation model achieved a moderate precision of 0.794 and a recall of 0.68, and the model for the reference object achieved a remarkable precision of 0.907 and a recall of 0.940. The moderate accuracy of the flower segmentation model is likely due to the low sample size of the image datasets used to fine-tune the flower (n = 379), which can lead to underfitting and reduce generalization performance [44]. To overcome the moderate flower detection in our study, we implemented an image tilling system, where each image was divided into four pieces for the flower segmentation model. Tilling has been shown to enhance segmentation performance by increasing resolution and reducing context noise, particularly in complex scenes [45]. However, this approach comes at the cost of increased computational time and memory usage.

Future studies can improve the flower segmentation model by expanding the training dataset to include a great diversity of flowers, in different contexts [46]. The current flower segmentation model was fine-tuned with 379 images of flowers from potted plants taken at garden centers. While there were many different colors and petal display categories include in the dataset, there are thousands of cultivars and species of flowering plants available that are currently not included in the fine-tuning dataset. Moreover, the flower segmentation model will likely will not perform well for images of flowering plants with complex background context such as flowers in gardens or in the field. Thus, the inclusion of a wider variety of plants and contexts will increase the utility of the FloralArea algorithm.

Additionally, future studies can also incorporate advance computer vision techniques to improve floral area estimation. For instance, future studies could utilize depth estimation models to expand the measurement of floral area from a two-dimensional (2D) measurement to a three-dimensional (3D) measurement, where the distance from a camera to flowering plants can be estimated from an image for floral area estimation [47]. This could also eliminate the need for a reference object for floral area measurement. Another approach for three-dimensional measurement is by using 2D to 3D diffusion model [48], which can render a 2D flower image into a 3D flower model for floral area measurements.

### Best practices for future FloralArea users

The FloralArea algorithm achieved the highest accuracy and lowest error when the camera was positioned closest to the flowering plant (S2 Fig). Thus, users of the FloralArea algorithm should take pictures close to the flowering plant. Users should also take photos of flowering plants on a simple background, such as potted plants placed on a concrete floor with the reference object. Taking pictures on a simple background will make it easier for the segmentation model to segment the flowers and the reference object from the background.

### Efficiency gains and scalability

The efficiency gains achieved by the FloralArea algorithm over traditional manual methods are a standout feature. In our evaluation, the algorithm reduced the time required for floral area measurements from an image by 99.24%. The efficiency of the algorithm opens new opportunities for assessing plant-pollinator interactions across space and time, an essential factor in selecting and breeding plant to support diverse pollinator communities [19]. Floral display area has been shown to be a key predictor of pollinator visitation [15,21], yet manual estimation techniques are time-intensive and prone to error [15]. FloralArea addresses this challenge by providing a consistent, repeatable, and automated approach for capturing floral area data from images. Moreover, the modular design of the FloralArea algorithm allows adaptation to other traits quantification tasks (e.g., leaf area estimation) [49], demonstrating its broader utility for research in ecology.

## Conclusion

This study presents FloralArea, an automated, scalable, and modular algorithm for estimating floral area from images. The algorithm achieves high accuracy (R^2^ = 0.93, RMSE = 20.58 cm^2^) and offers substantial efficiency gains by reducing the time associated with floral measurement from an image by over 99%. By streamlining floral quantification, FloralArea supports large-scale ecological studies and plant-pollinator research, offering a practical tool for monitoring floral traits across space, time and environmental gradients [15]. The FloralArea algorithm can be accessed online at: https://floralarea.vmhost.psu.edu/. The source code and user instructions are available for running the FloralArea algorithm manually can be found on the GitHub repository (https://github.com/eai6/FloralArea_Web.git). The FloralArea algorithm will be integrated into an application for mobile devices called FloraCount (unpublished) that allows plant breeders and evaluators to use the rapid evaluation protocol developed by Erickson et al. (2022) to ensure high-quality data collection, facilitating large-scale assessments of flowering plant attractiveness to pollinators [15]. This tool can play a pivotal role in informing flowering plant selection and breeding to improve the availability of plants that support diverse pollinators and their use in habitat restoration and management.

## Supporting information

S1 Fig ImageJ measurement protocol. (A) represents a sample of the evaluation images. The reference object was used to set the scale for ImageJ measurement (B). The free-form tool was then used to draw a boundary around all the flowers in the images to get the floral area for all the flowers in the image (C).
(TIF)

S2 Fig FloralArea accuracy (i.e., Predicted area vs. Measured area) for images taken at 60 cm, 80 cm, and 100 cm above the ground. Accuracy was evaluated with regression analysis. The regression line, coefficient of determination (R^2^), root mean squared error (RMSE), and sample size (n) are shown on the plots. The predicted floral area (cm^2^) is on the y-axis, and the measured floral area (cm^2^) is on the x-axis. (A) FloralArea accuracy for images taken at 60 cm above the ground. (B) FloralArea accuracy for images taken at 80 cm above the ground. (C) FloralArea for images taken at 80 cm. One outlier was removed from the regression analysis and the root mean square error calculation. This outlier is indicated as red color on the plot. (D) FloralArea accuracy for images taken at 100 cm above the ground.
(TIF)

S3 Fig FloralArea algorithm website application demonstration. This is the Gradio interface for demonstrating the FloralArea algorithm for flower area measurement.
(TIF)

S1 Table Flower color chart. Twenty-three flowering plants were used to evaluate the efficiency and accuracy of the FloralArea algorithm, and images were taken of these plants at multiple heights, resulting in 75 total images. These flowering plants were sorted into five categories (T1-5) to investigate the influence of color on the algorithm’s performance.
(DOCX)

S2 Table Flower petal display category chart. The flowering plants were organized into three categories (C1-3) according to flower traits to investigate the influence of flower traits on the algorithm’s performance.
(DOCX)
