# Supplementary figures and images for: FloralArea: AI-powered algorithm for automated calculation of floral area from flower images to support plant and pollinator research
Source: PLoS One. 2025 Sep 12;20(9):e0332165. doi: 10.1371/journal.pone.0332165 (PMC12431086; doi:10.1371/journal.pone.0332165)

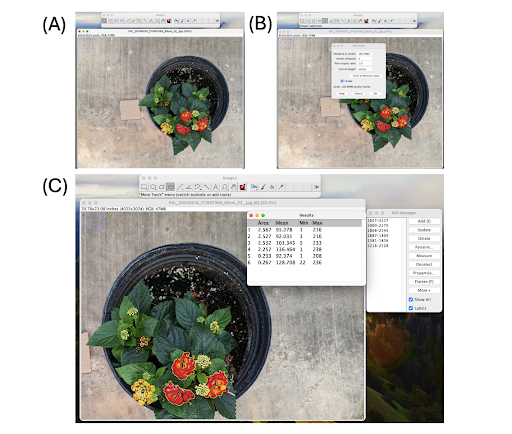

Supplement: S1 Fig — (A) represents a sample of the evaluation images. The reference object was used to set the scale for ImageJ measurement (B). The free-form tool was then used to draw a boundary around all the flowers in the images to get the floral area for all the flowers in the image (C). (TIF) [file pone.0332165.s001.tif]

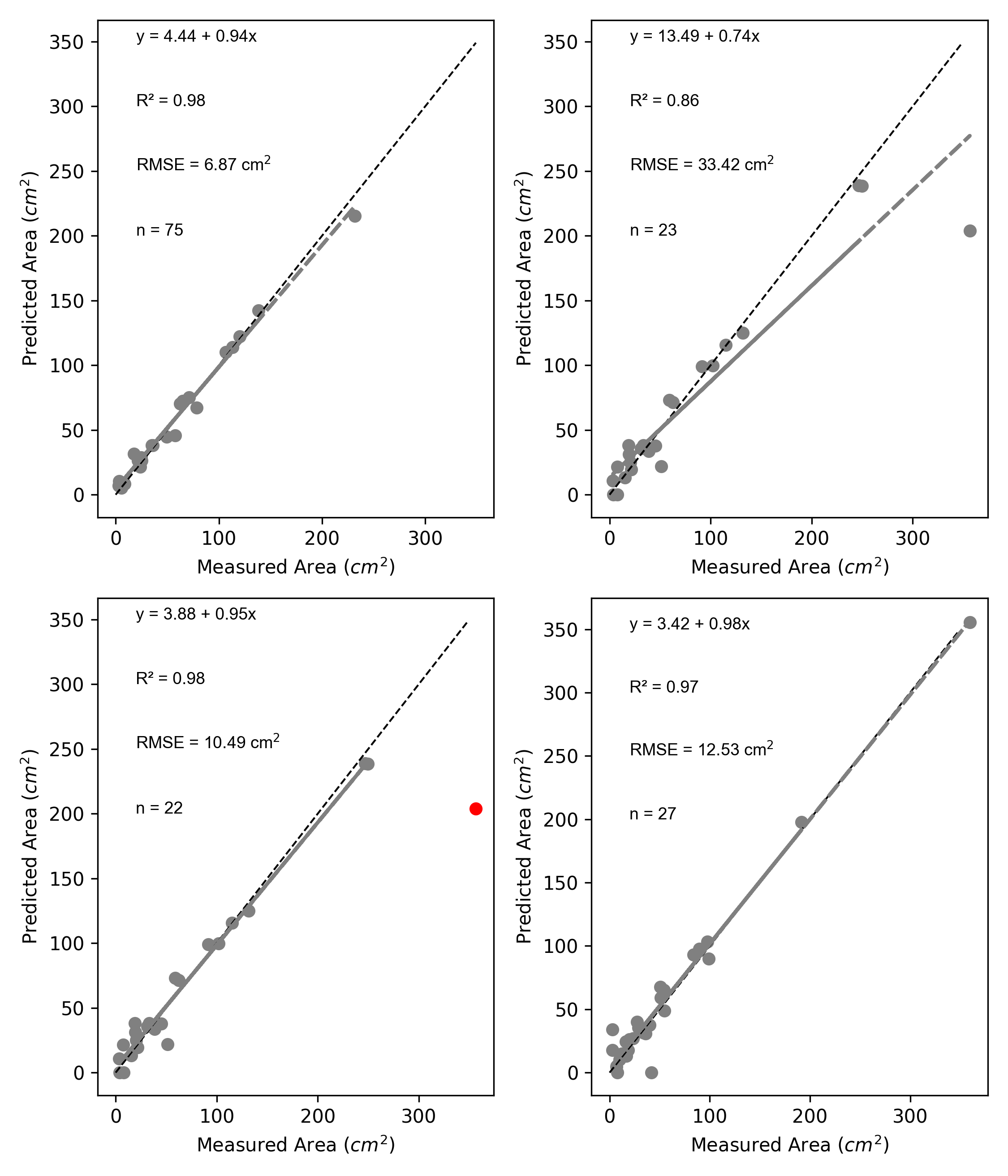

Supplement: S2 Fig — Accuracy was evaluated with regression analysis. The regression line, coefficient of determination (R2), root mean squared error (RMSE), and sample size (n) are shown on the plots. The predicted floral area (cm2) is on the y-axis, and the measured floral area (cm2) is on the x-axis. (A) FloralArea accuracy for images taken at 60 cm above the ground. (B) FloralArea accuracy for images taken at 80 cm above the ground. (C) FloralArea for images taken at 80 cm. One outlier was removed from the regression analysis and the root mean square error calculation. This outlier is indicated as red color on the plot. (D) FloralArea accuracy for images taken at 100 cm above the ground. (TIF) [file pone.0332165.s002.tif]

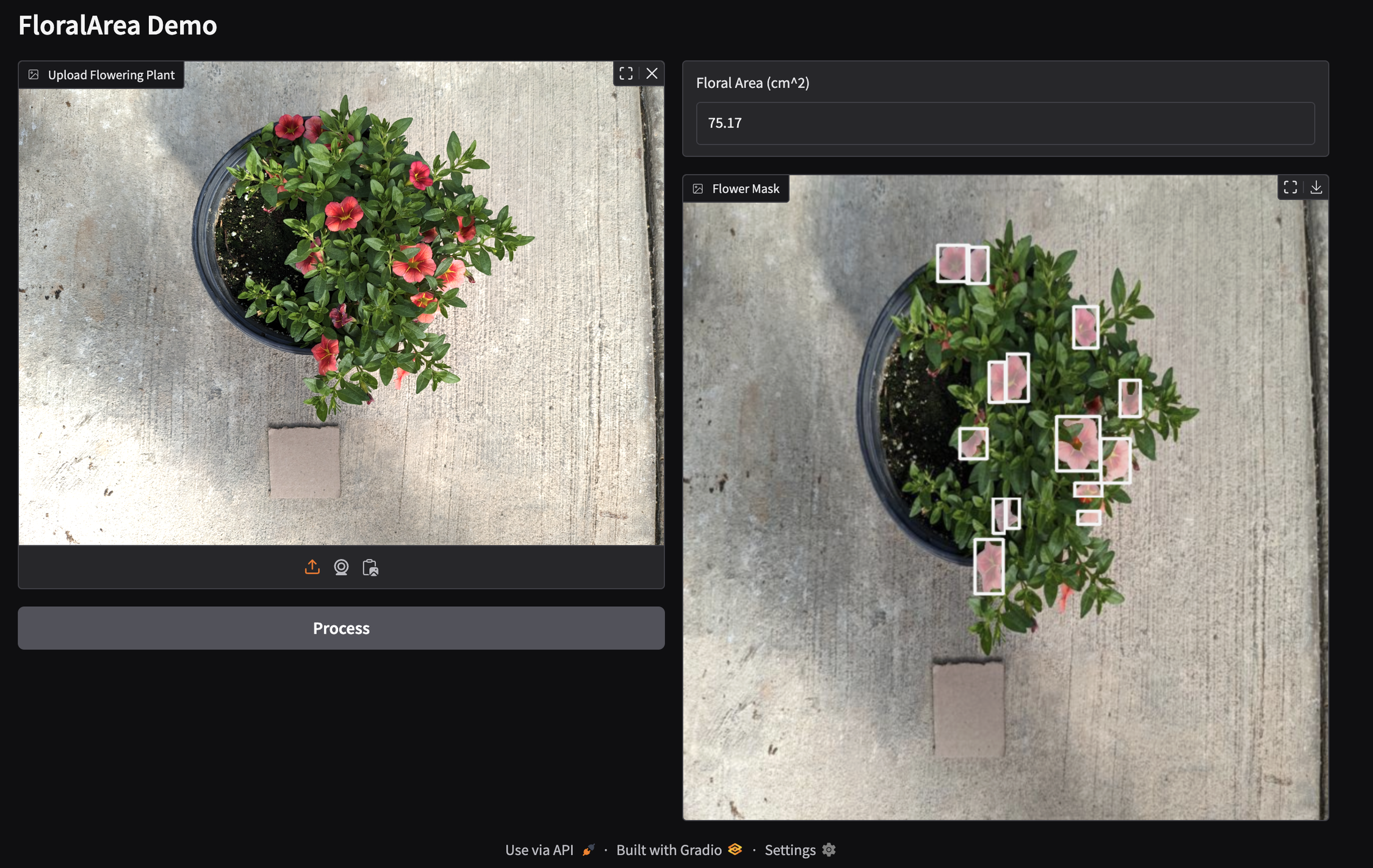

Supplement: S3 Fig — This is the Gradio interface for demonstrating the FloralArea algorithm for flower area measurement. (TIF) [file pone.0332165.s003.tif]

**Supplementary Table** **S1. Flower Color Chart.**

| **T1 - Red, Orange, Yellow** | **T2 - Fuchsia** | **T3 - Hotpink** | **T4 - Red** | **T5 - Purple** |
| --- | --- | --- | --- | --- |
| 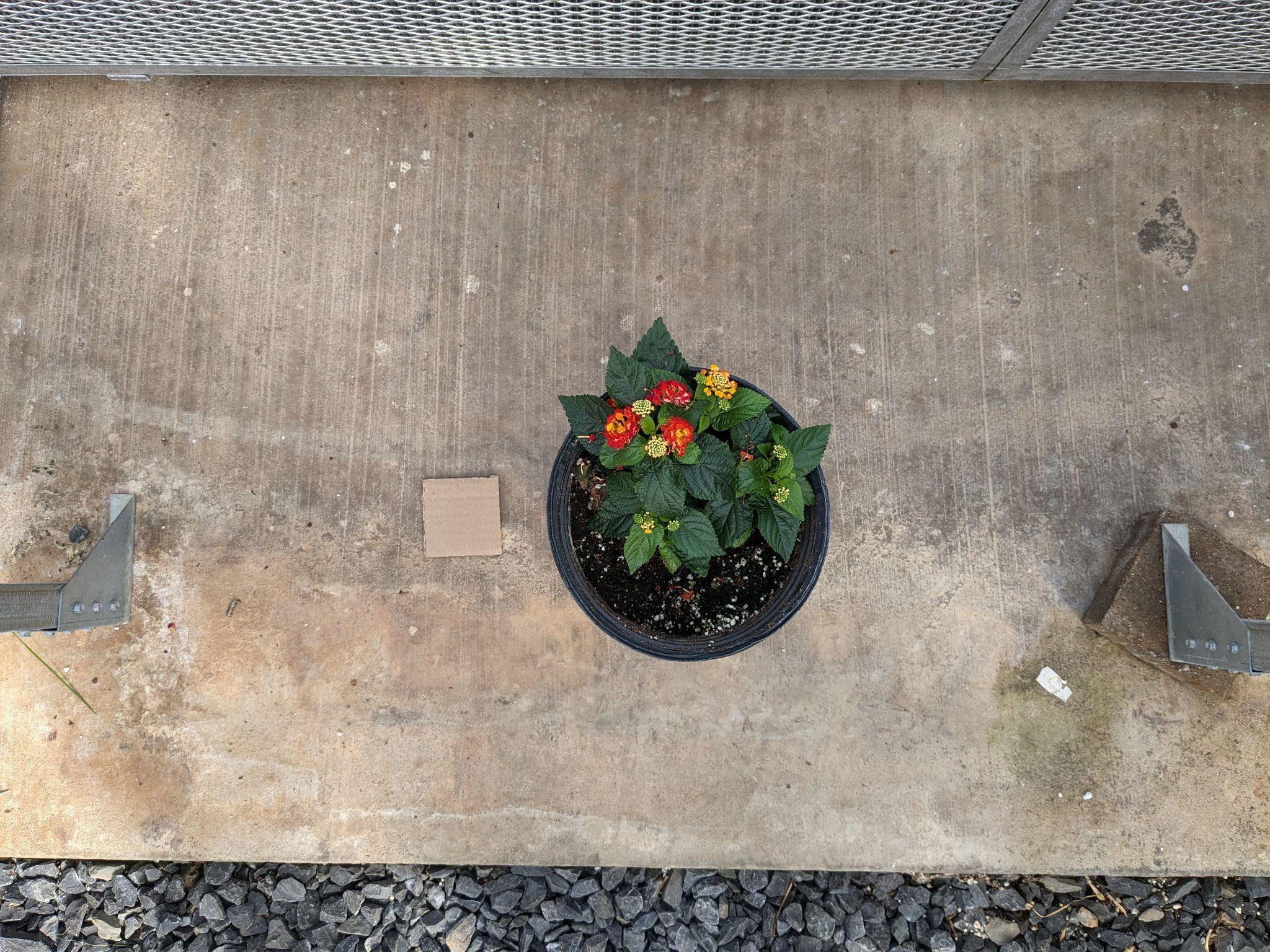 | 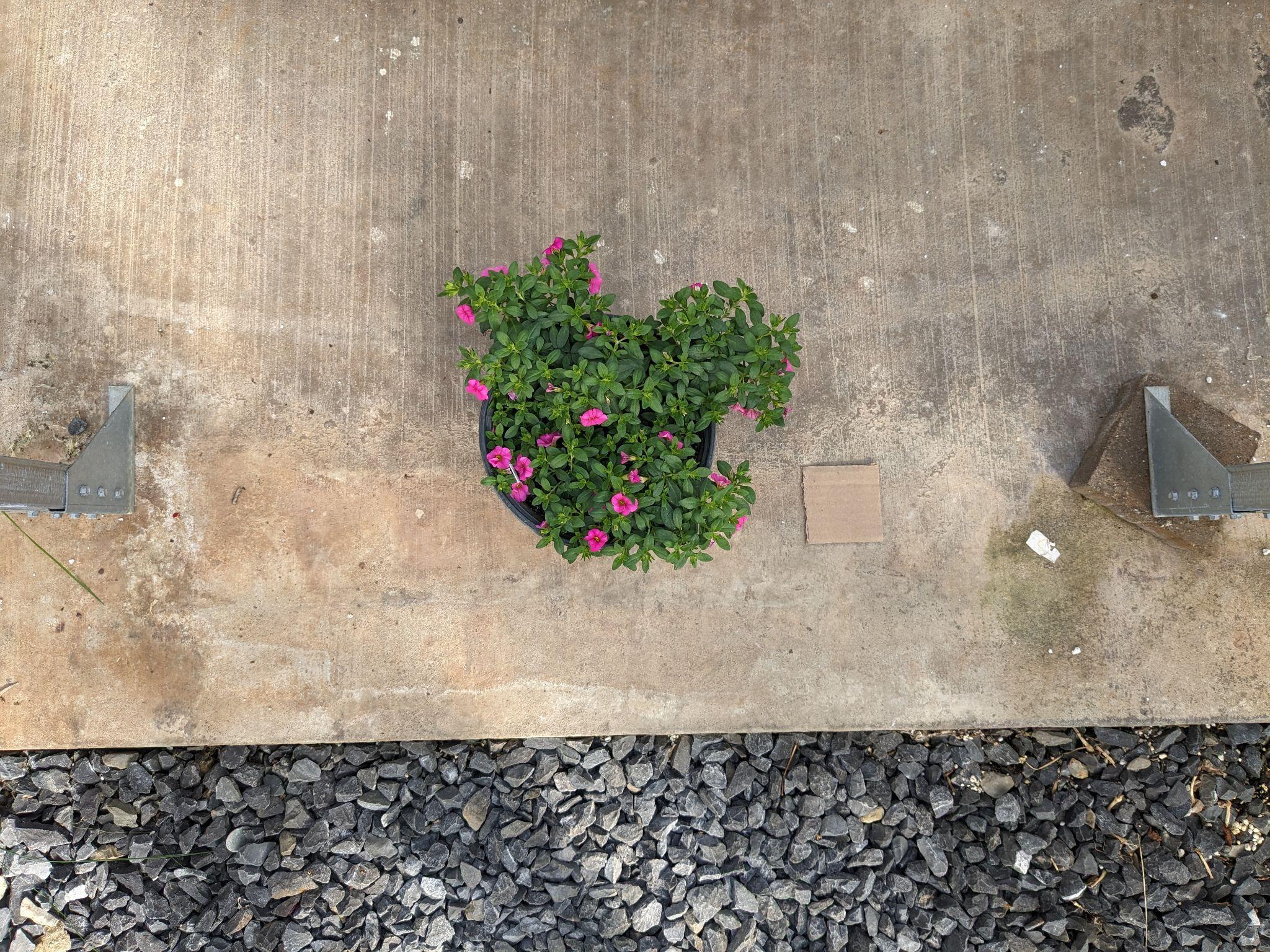 | 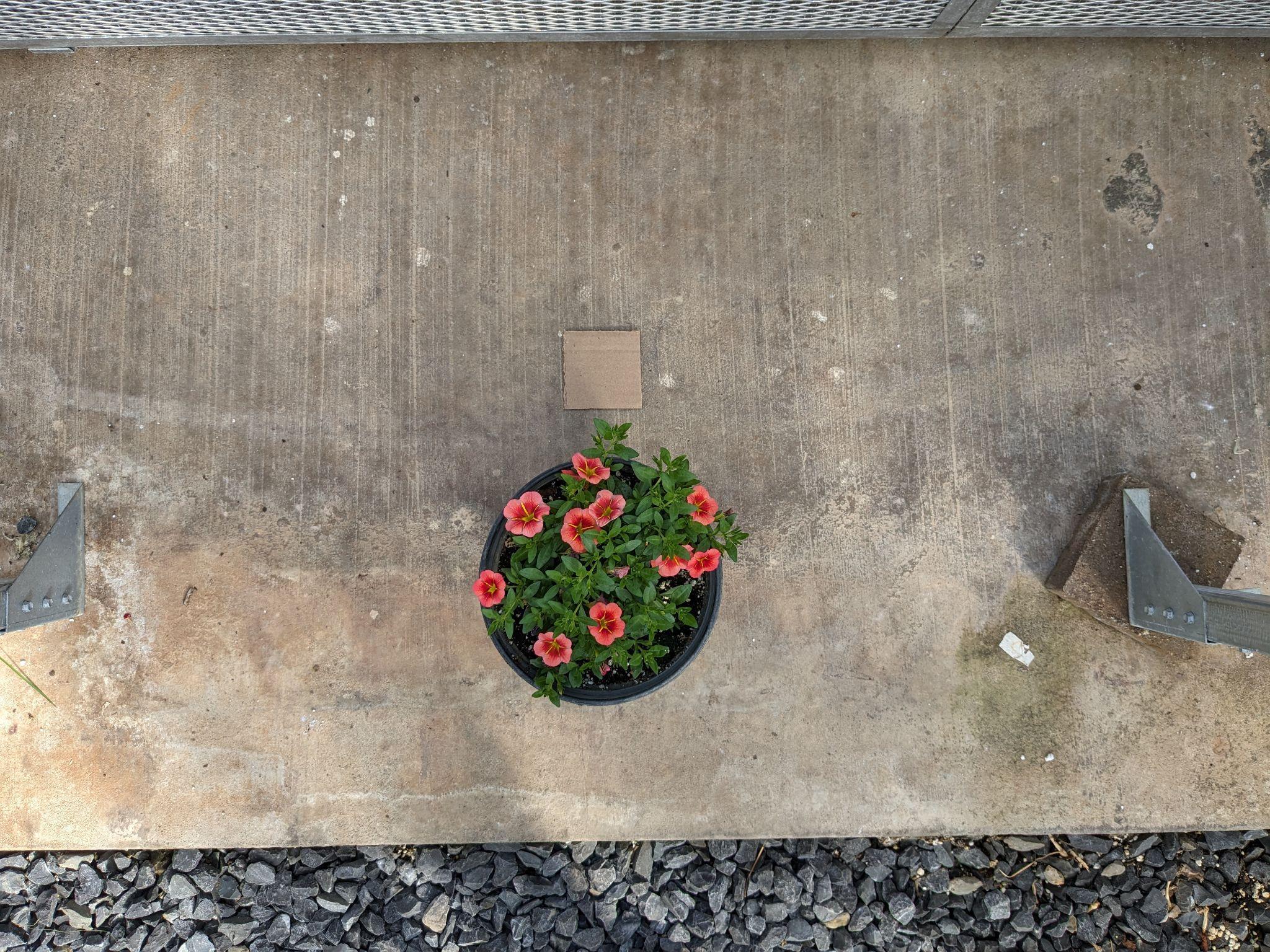 | 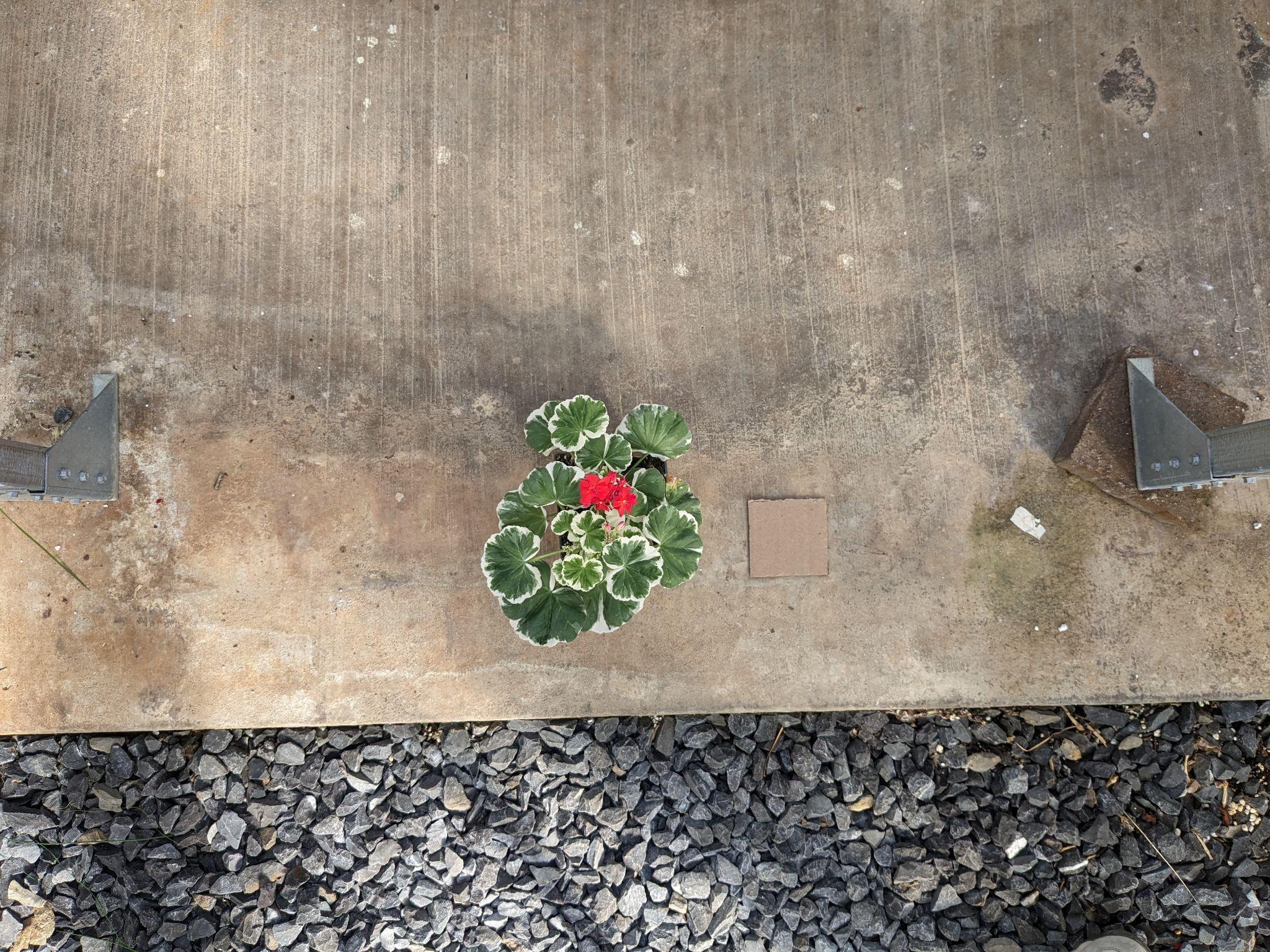 | 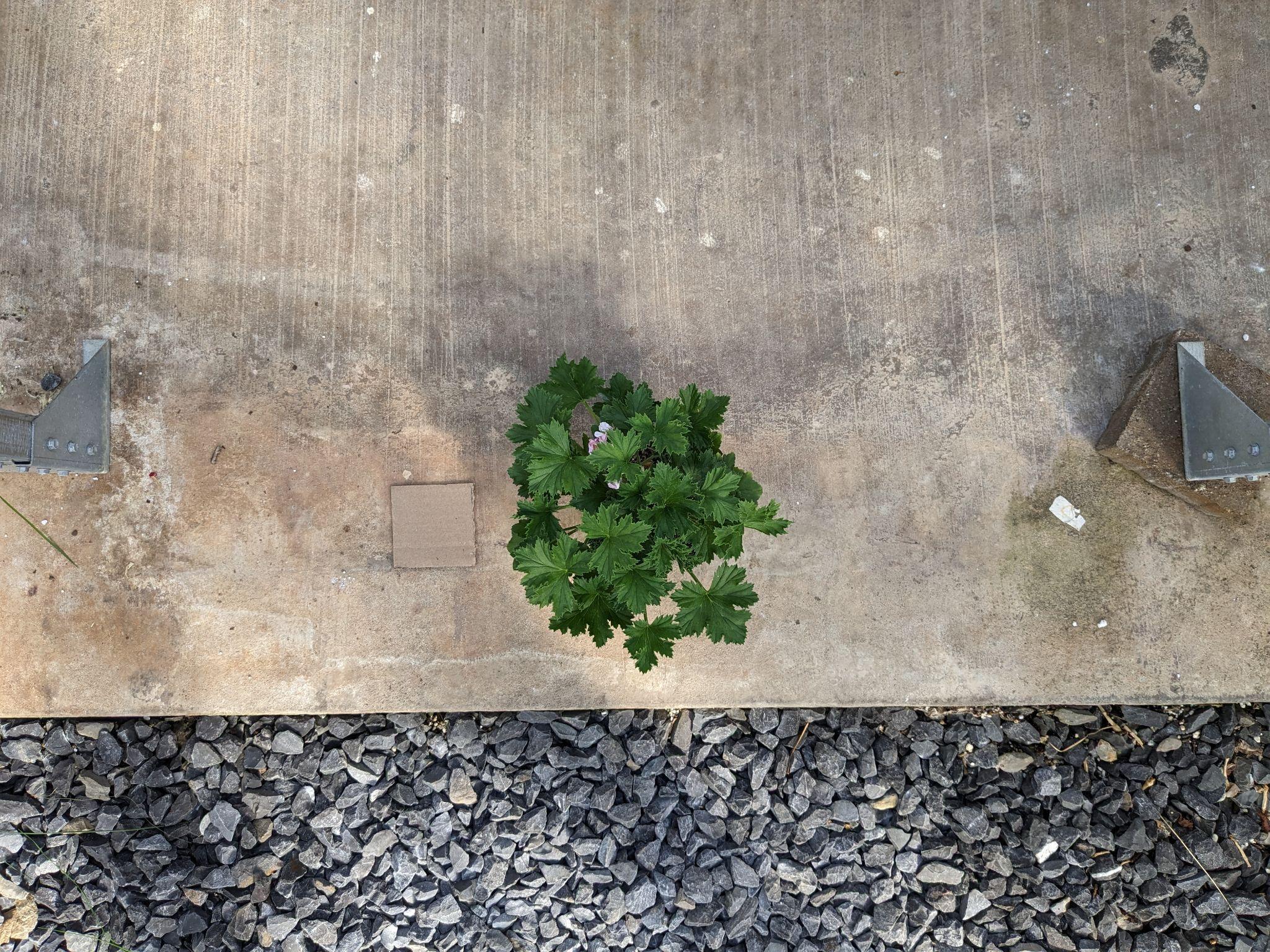 |
| 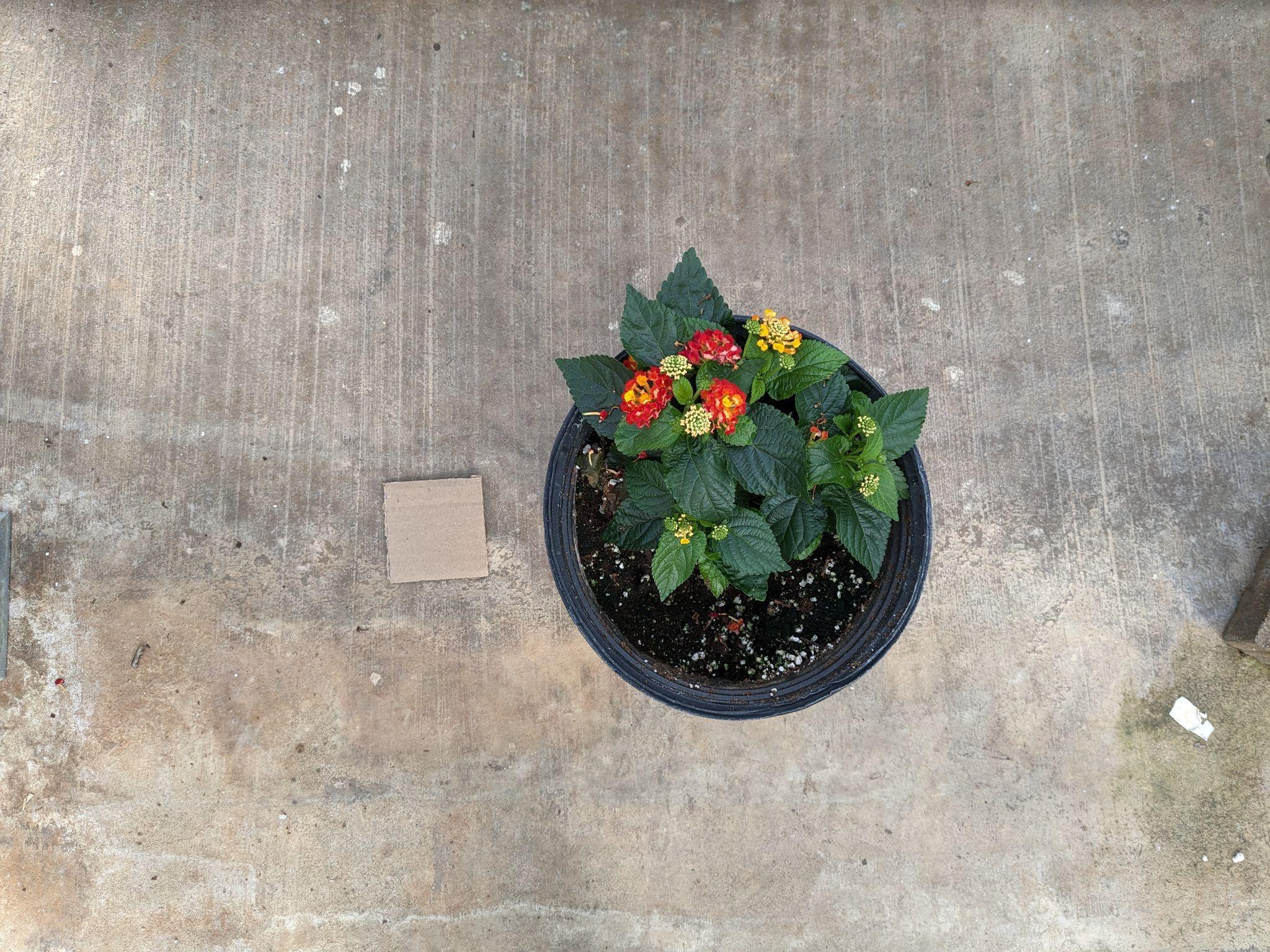 | 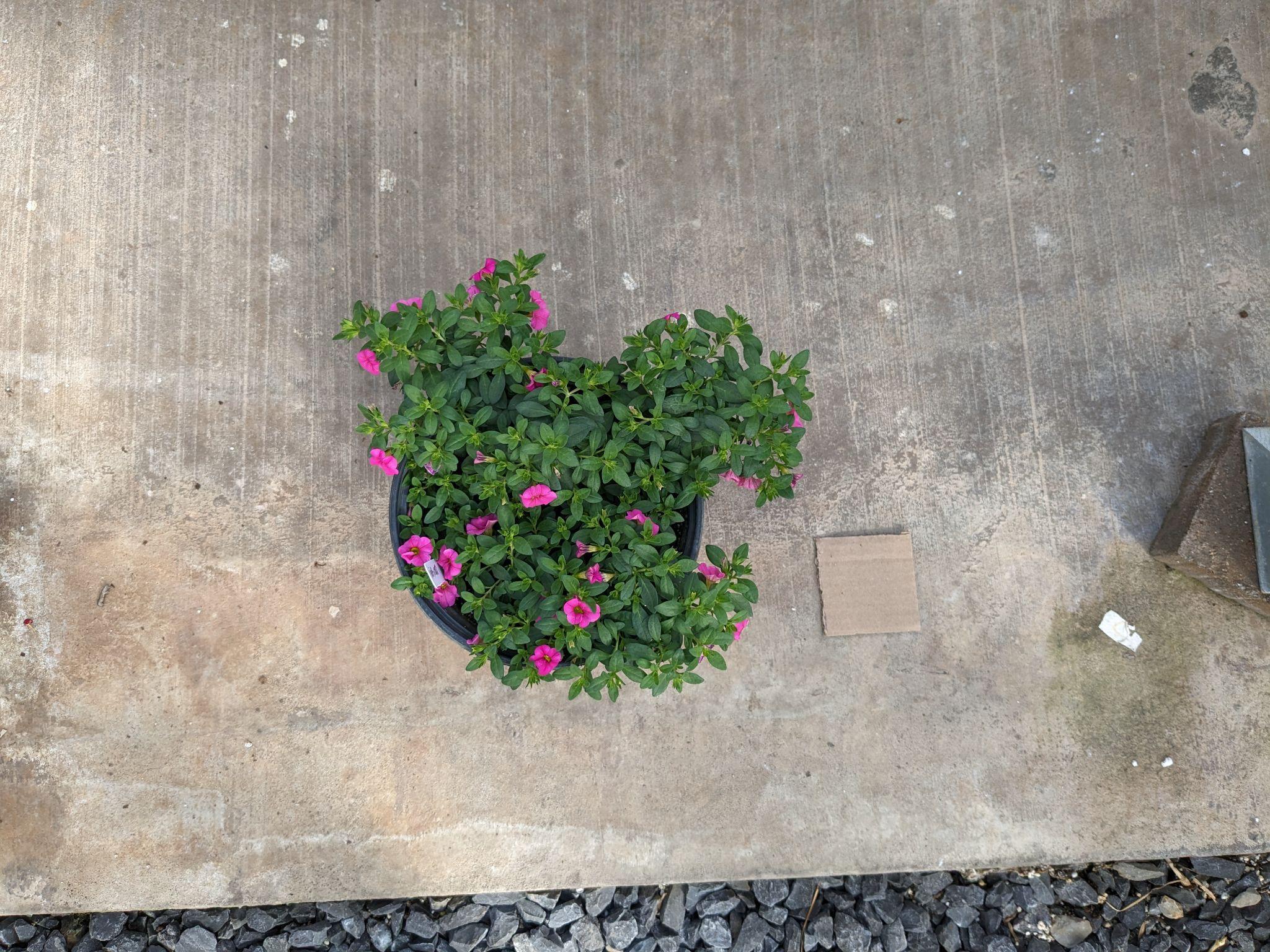 | 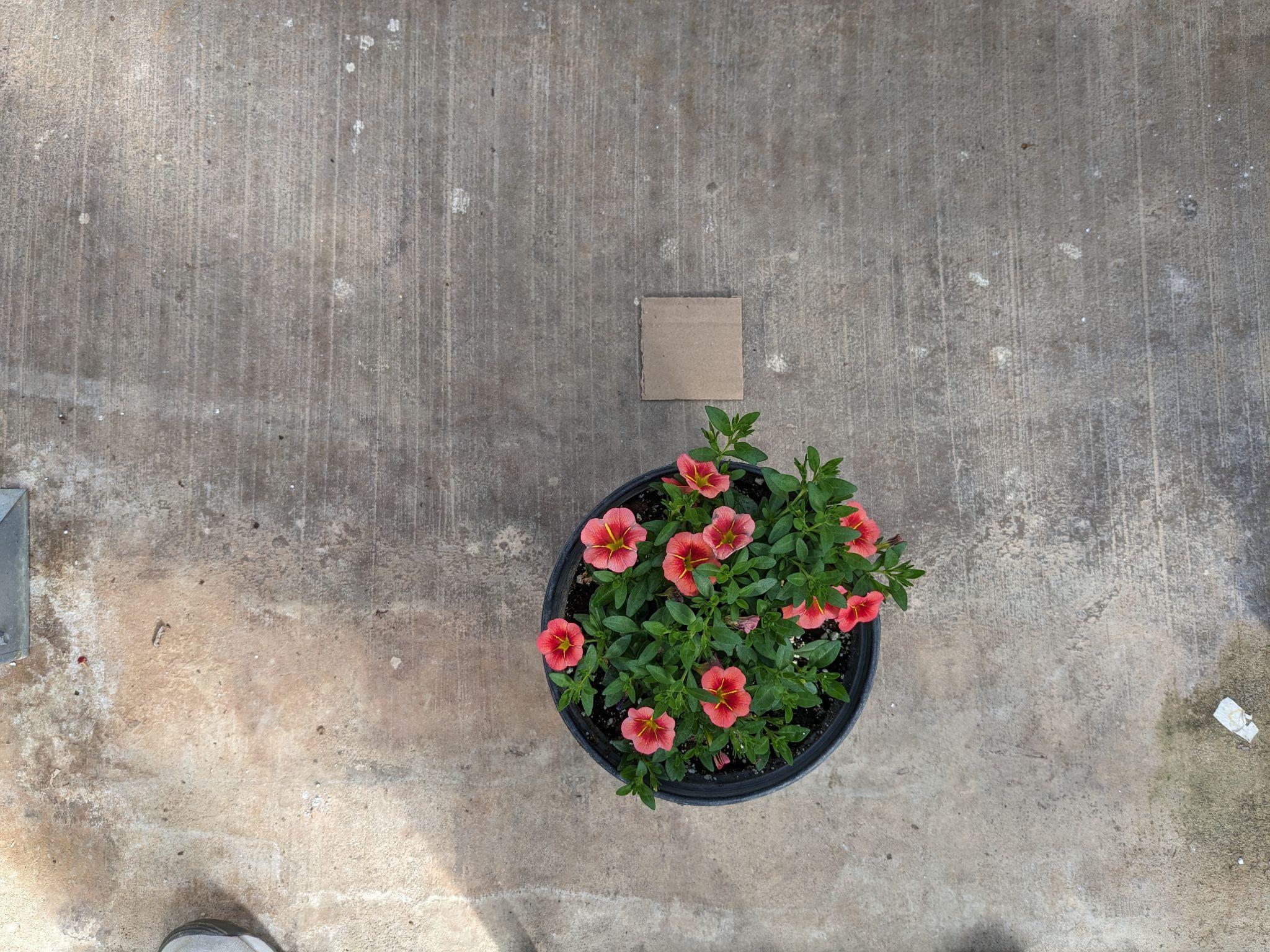 | 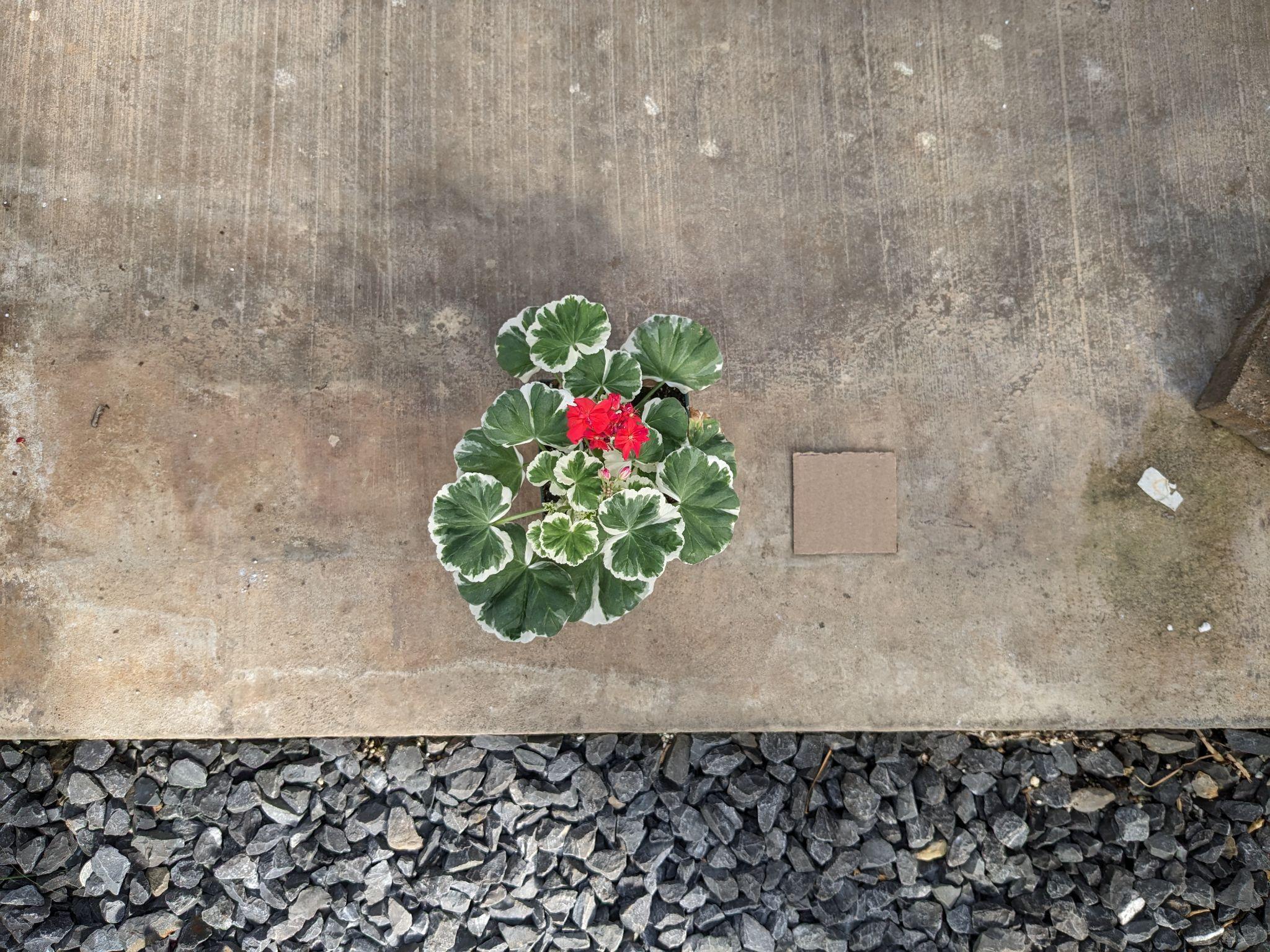 | 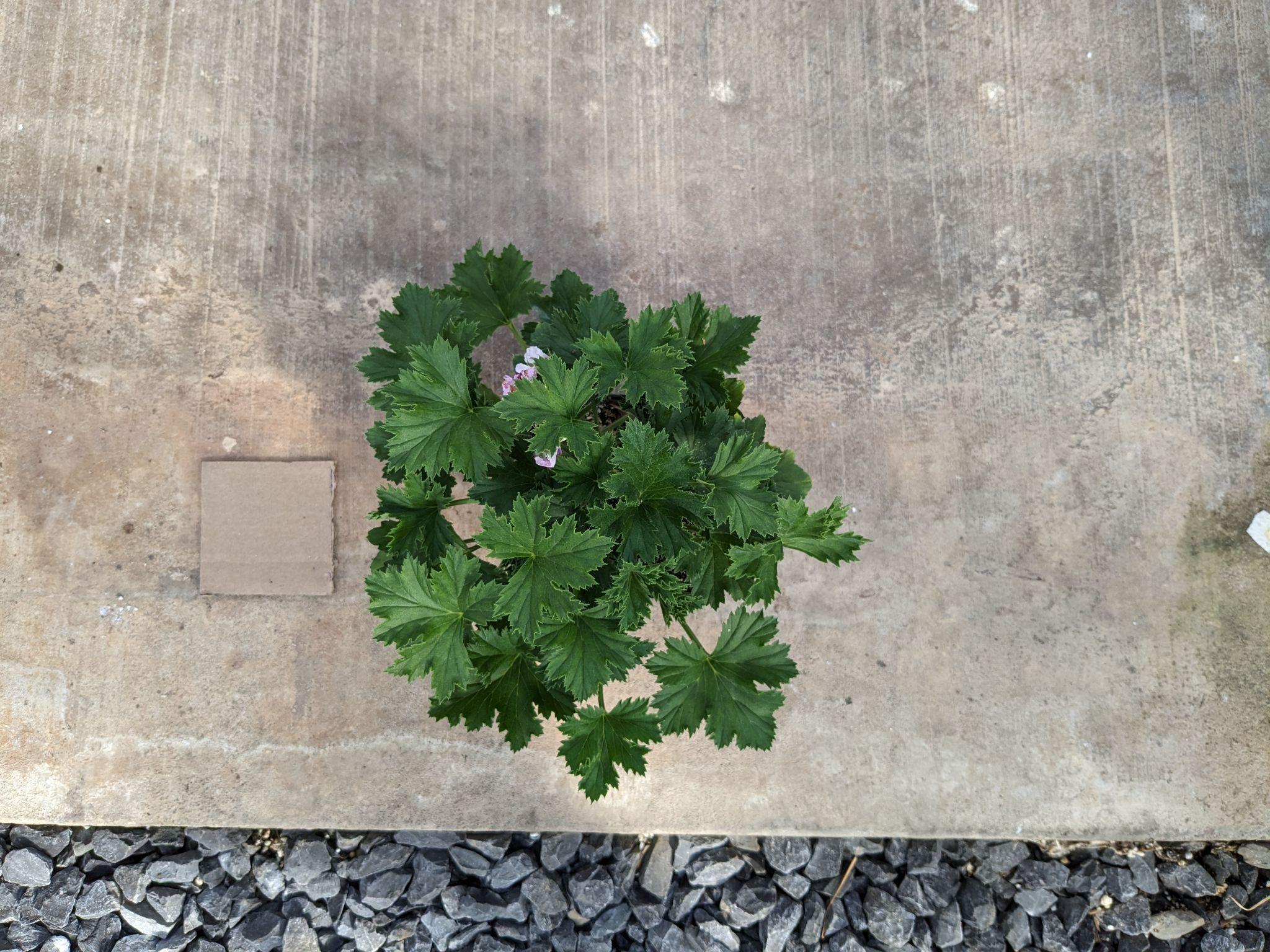 |
| 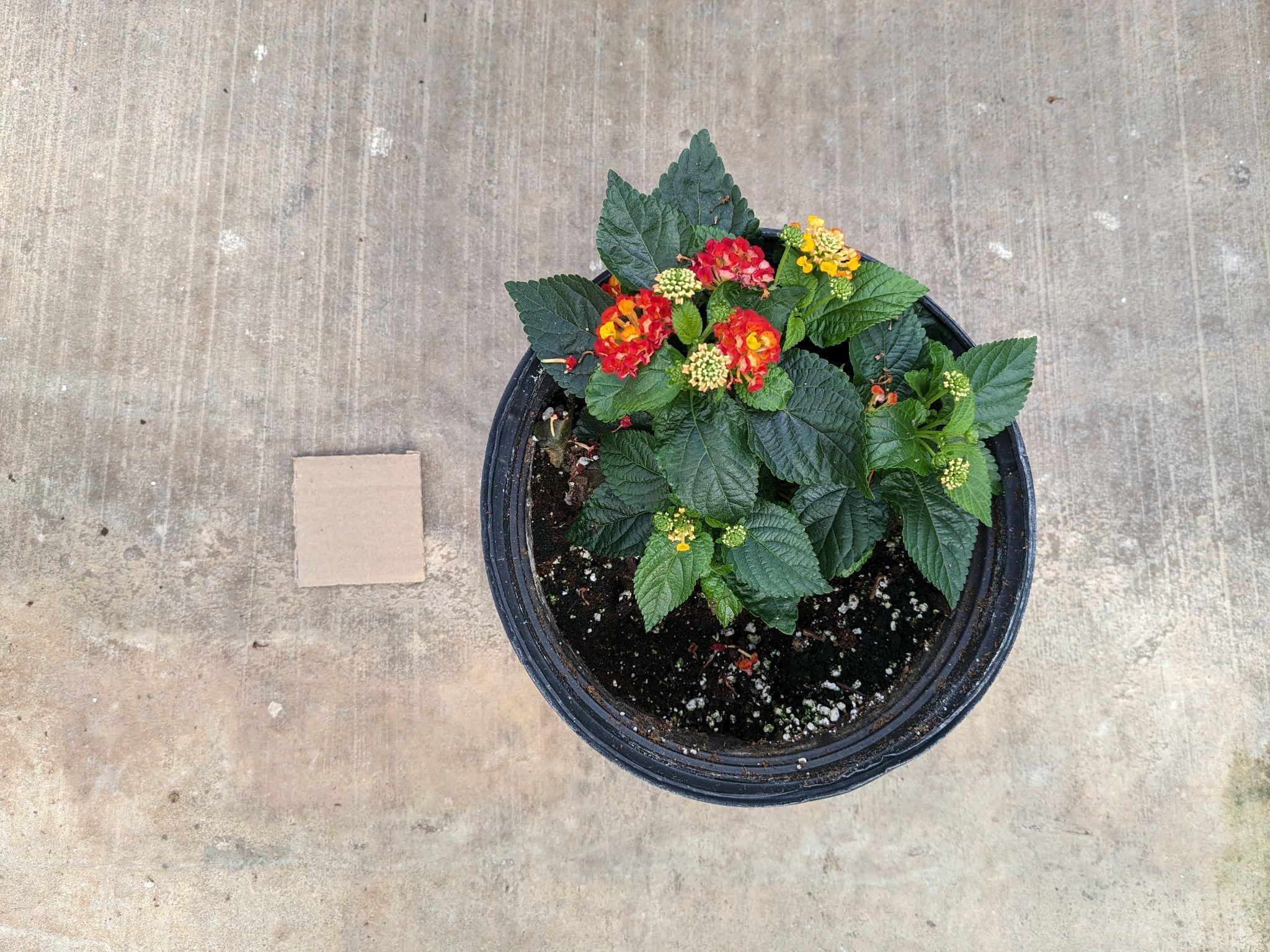 | 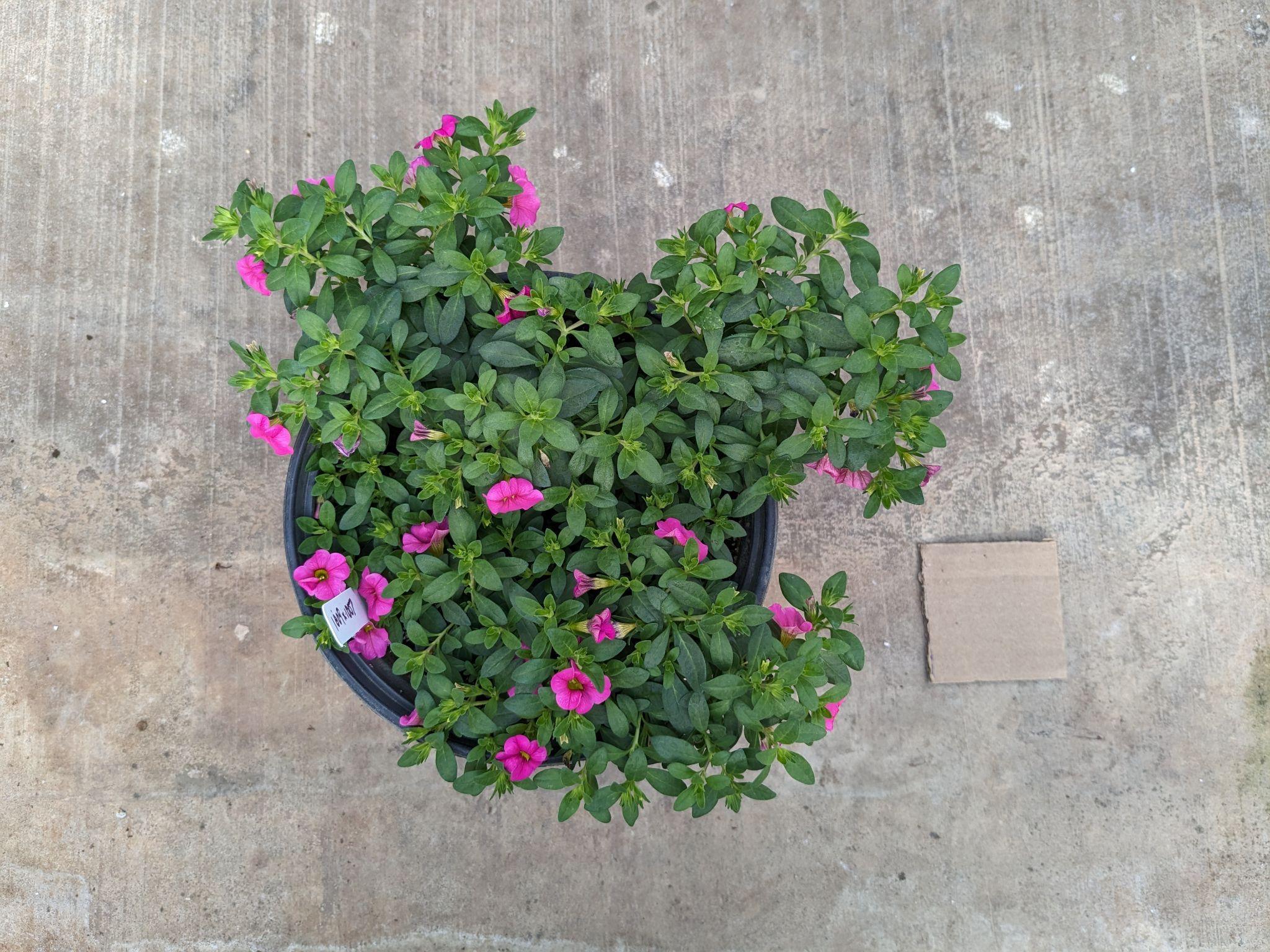 | 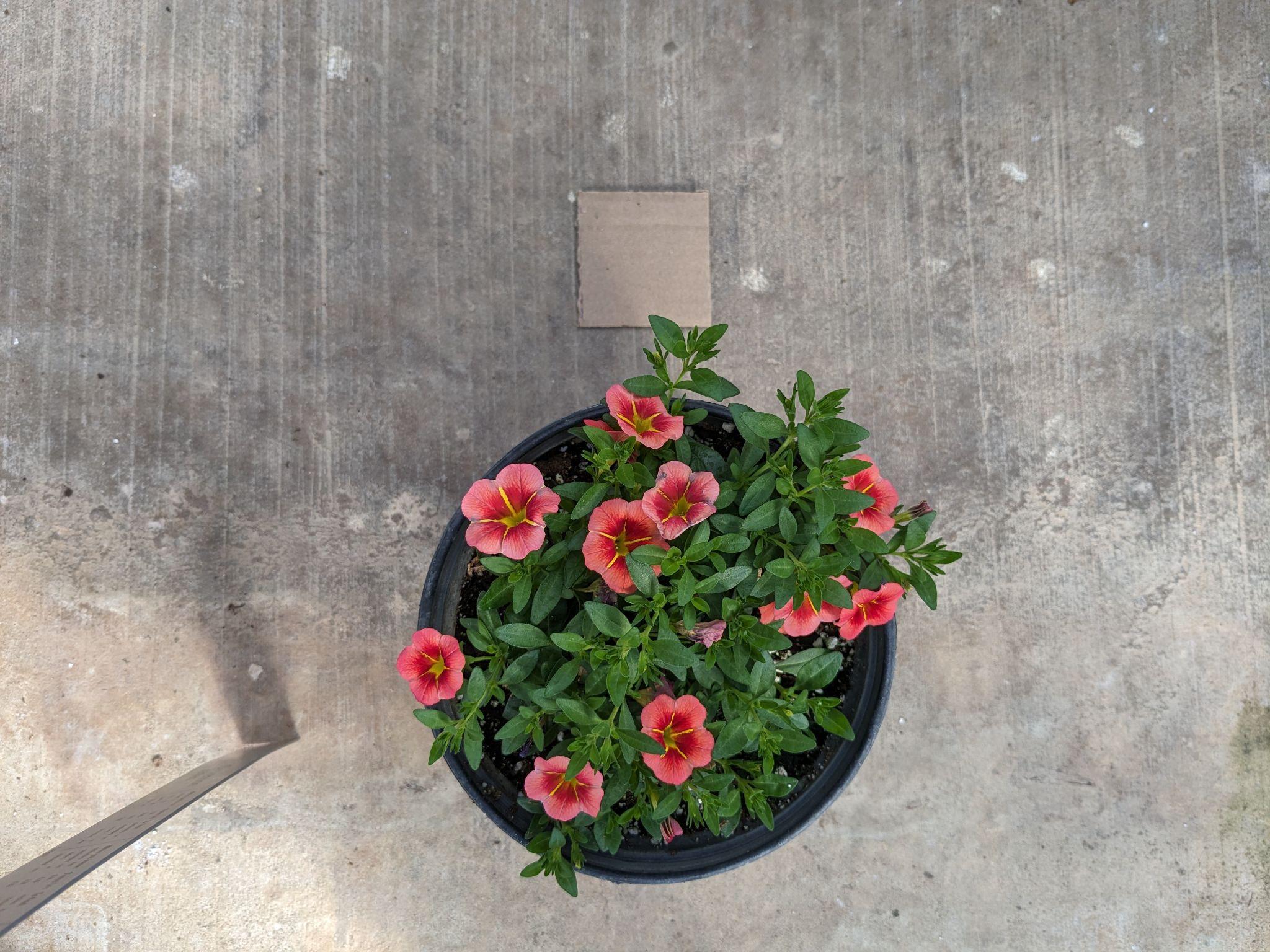 | 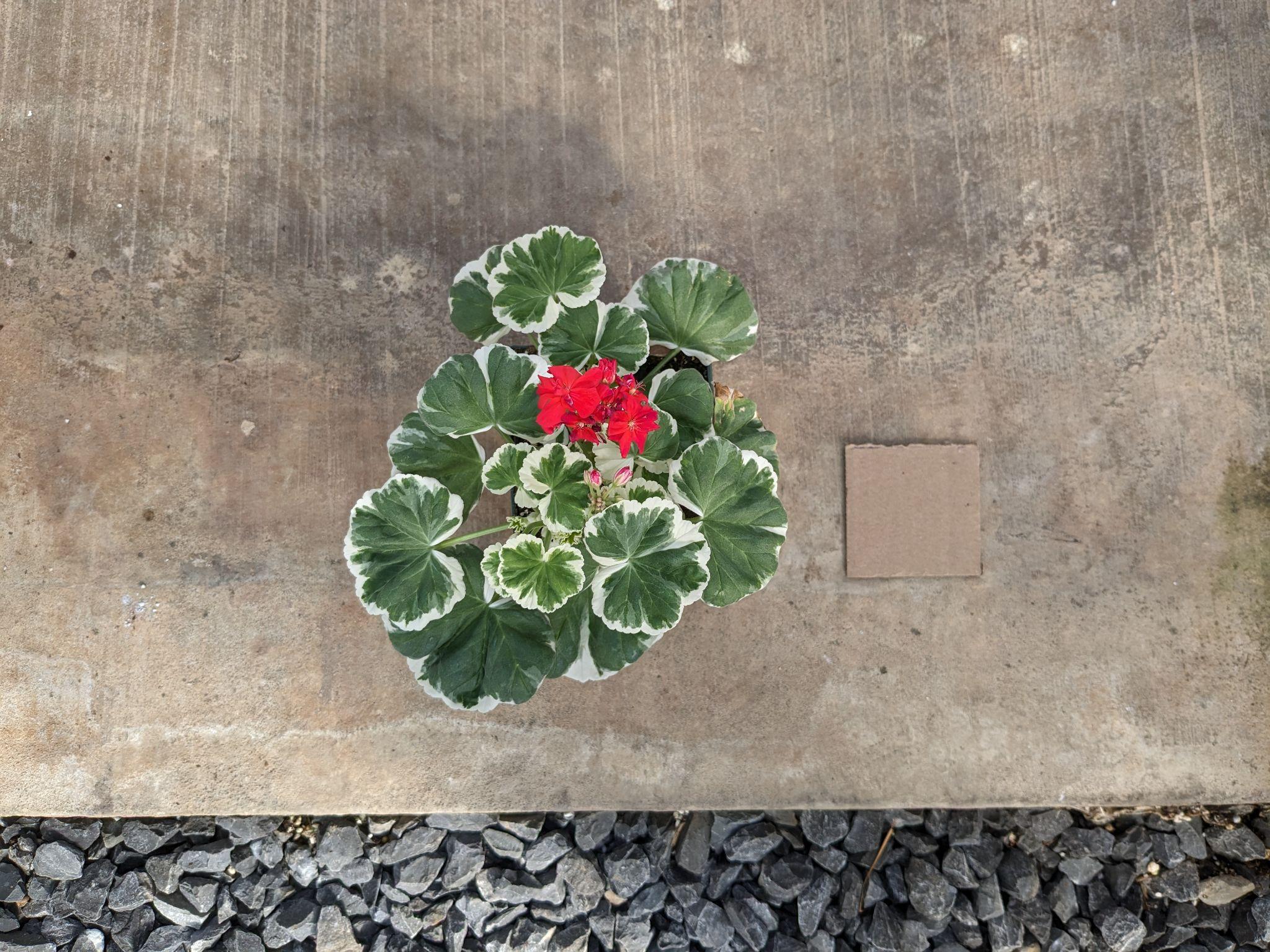 | 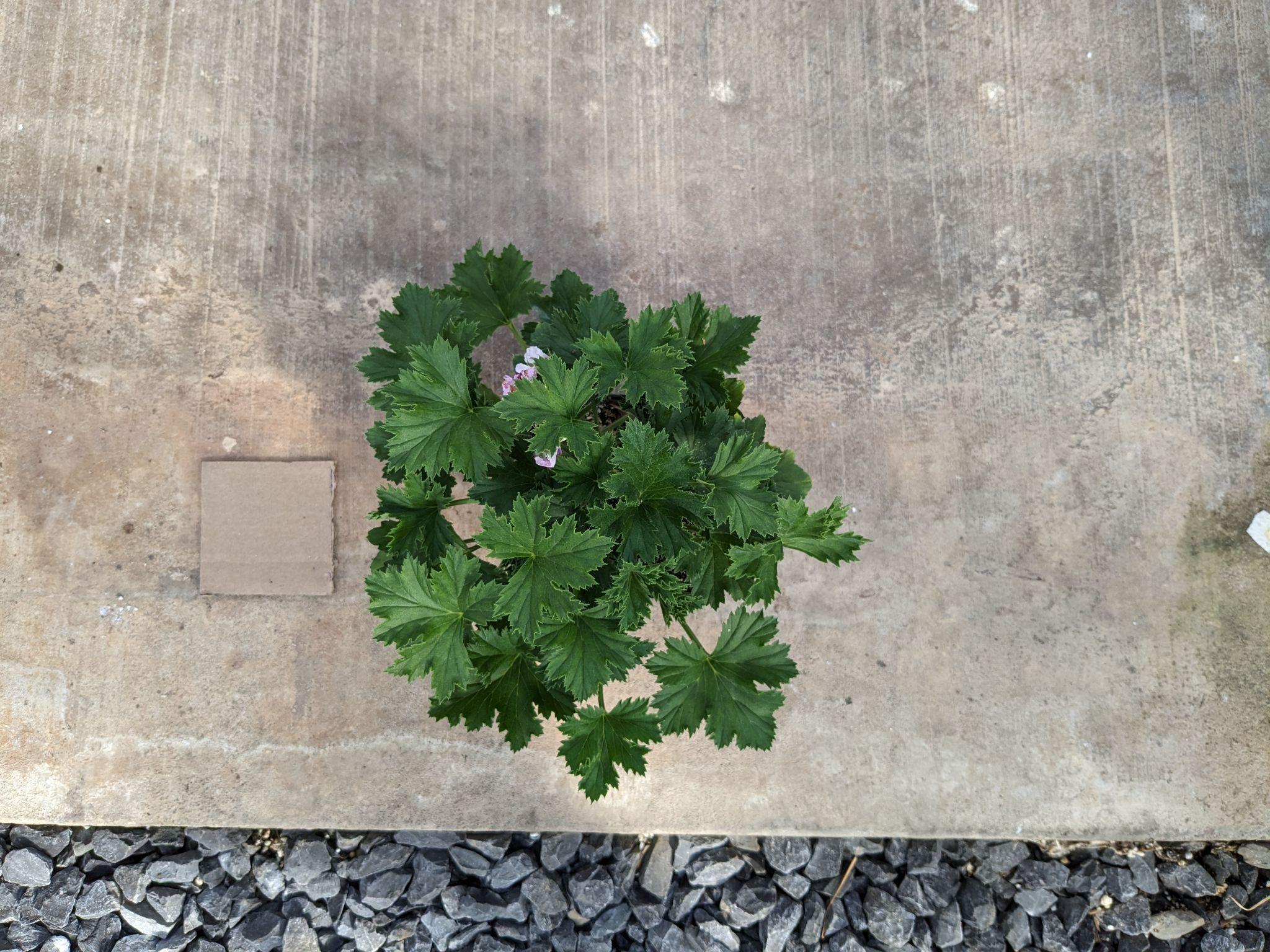 |
| 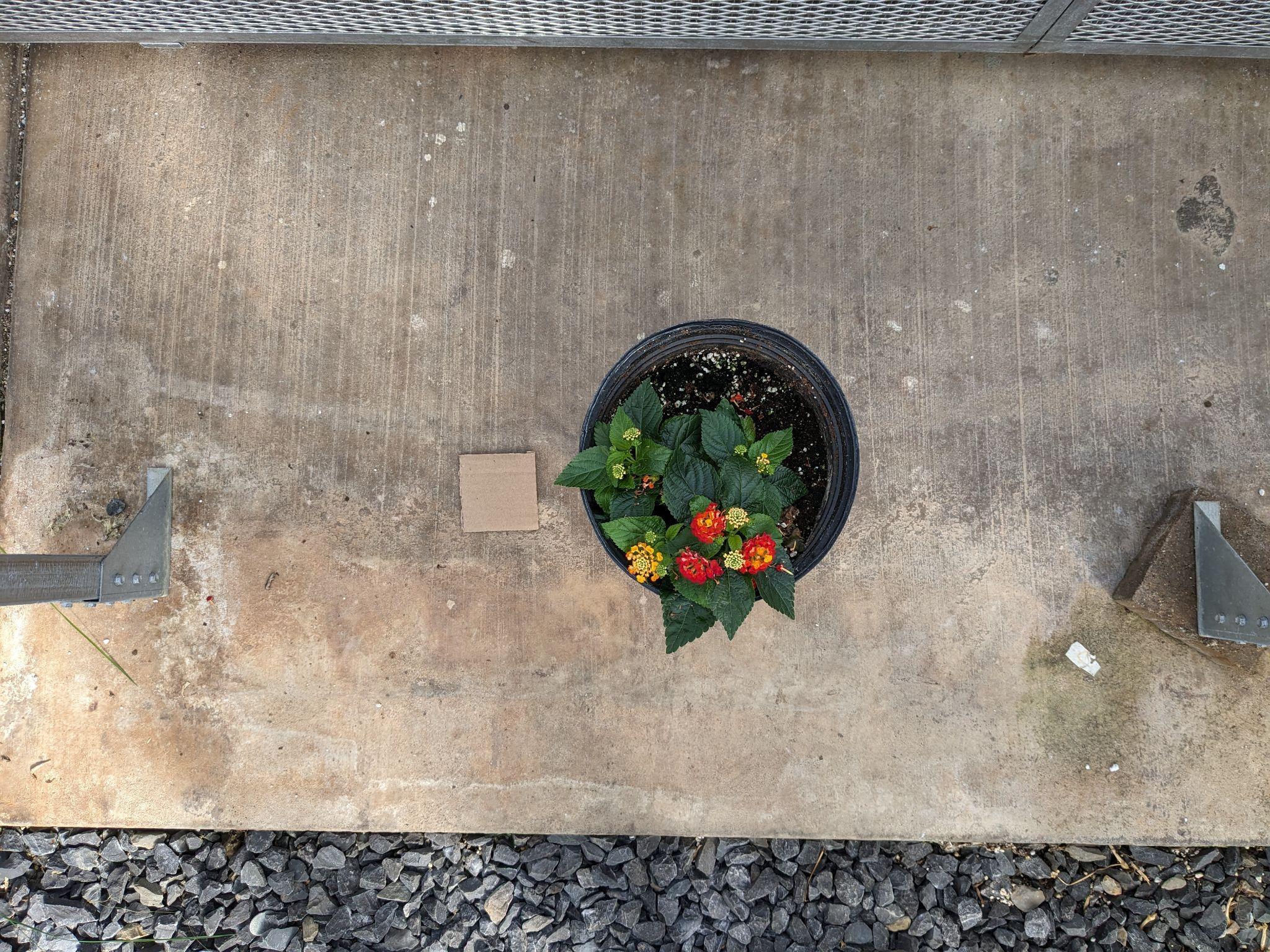 | 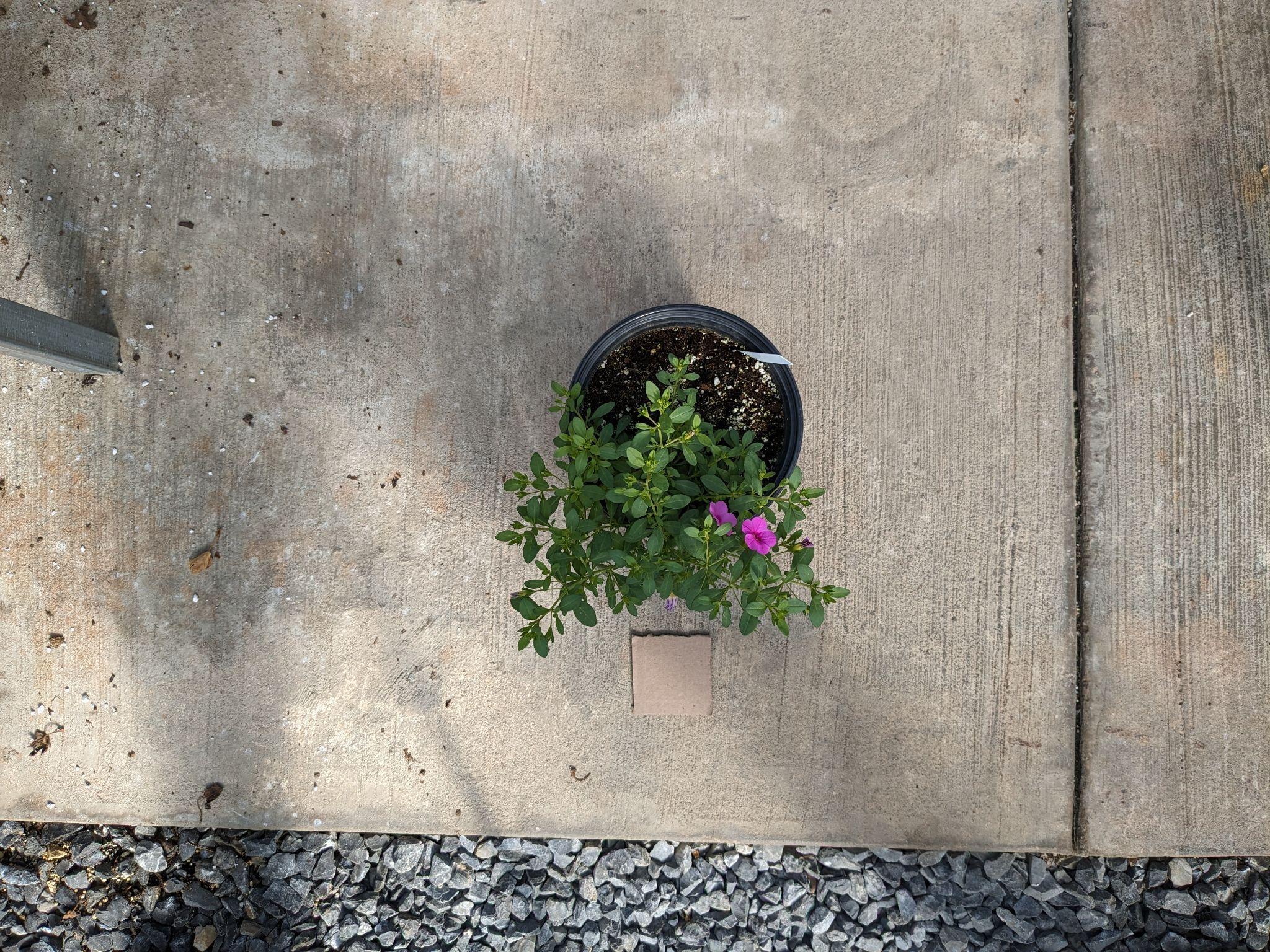 | 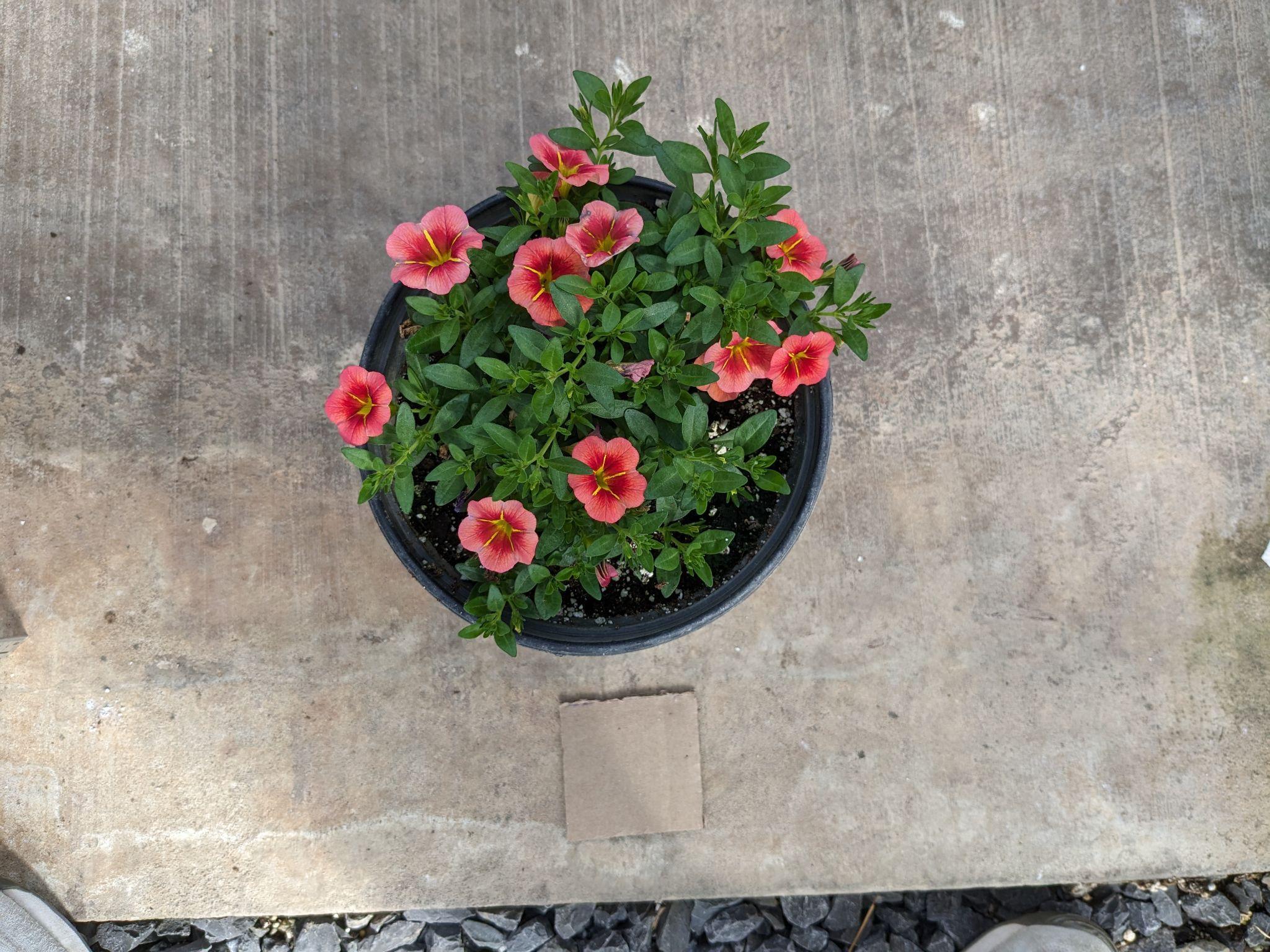 | 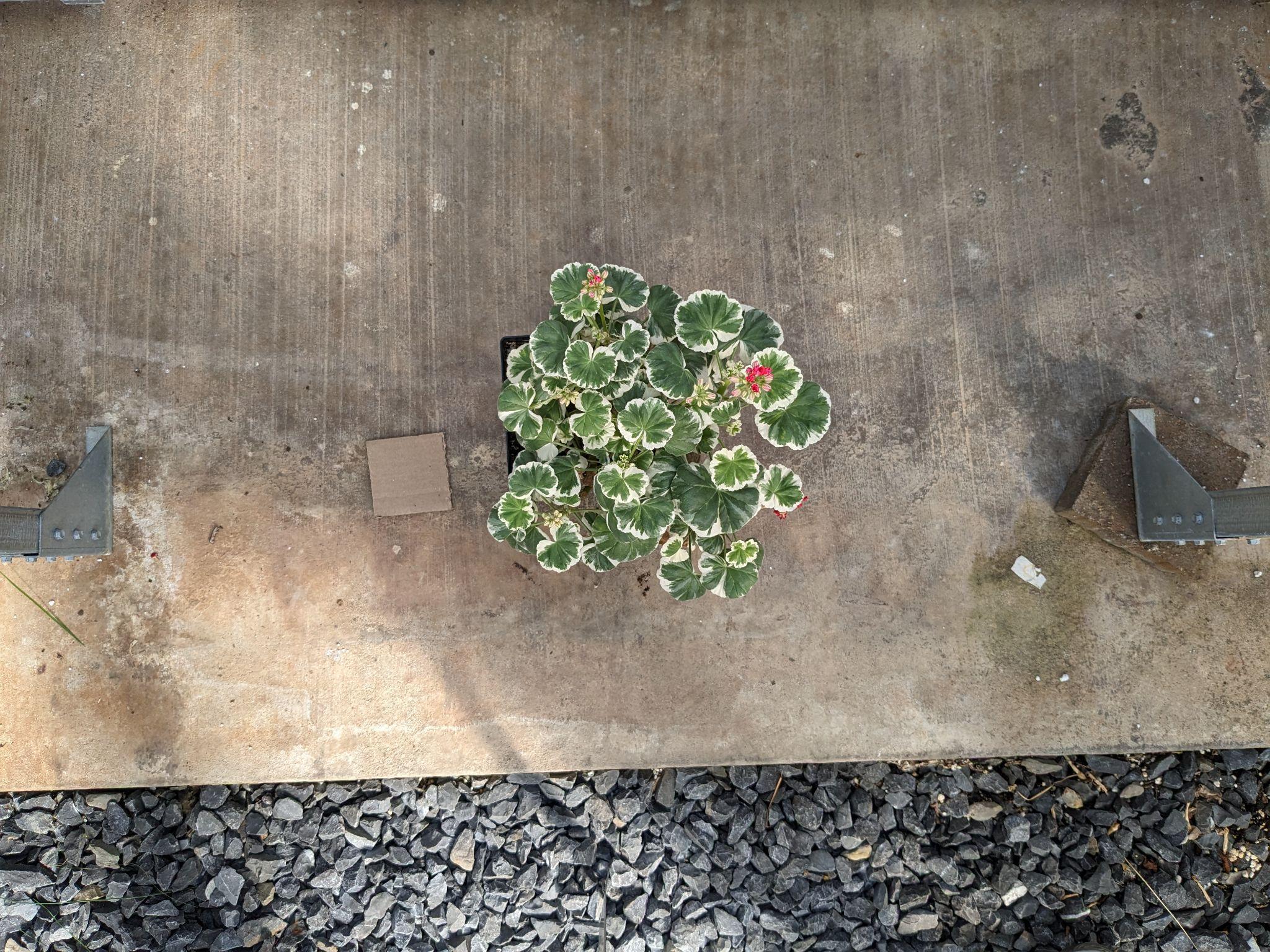 | 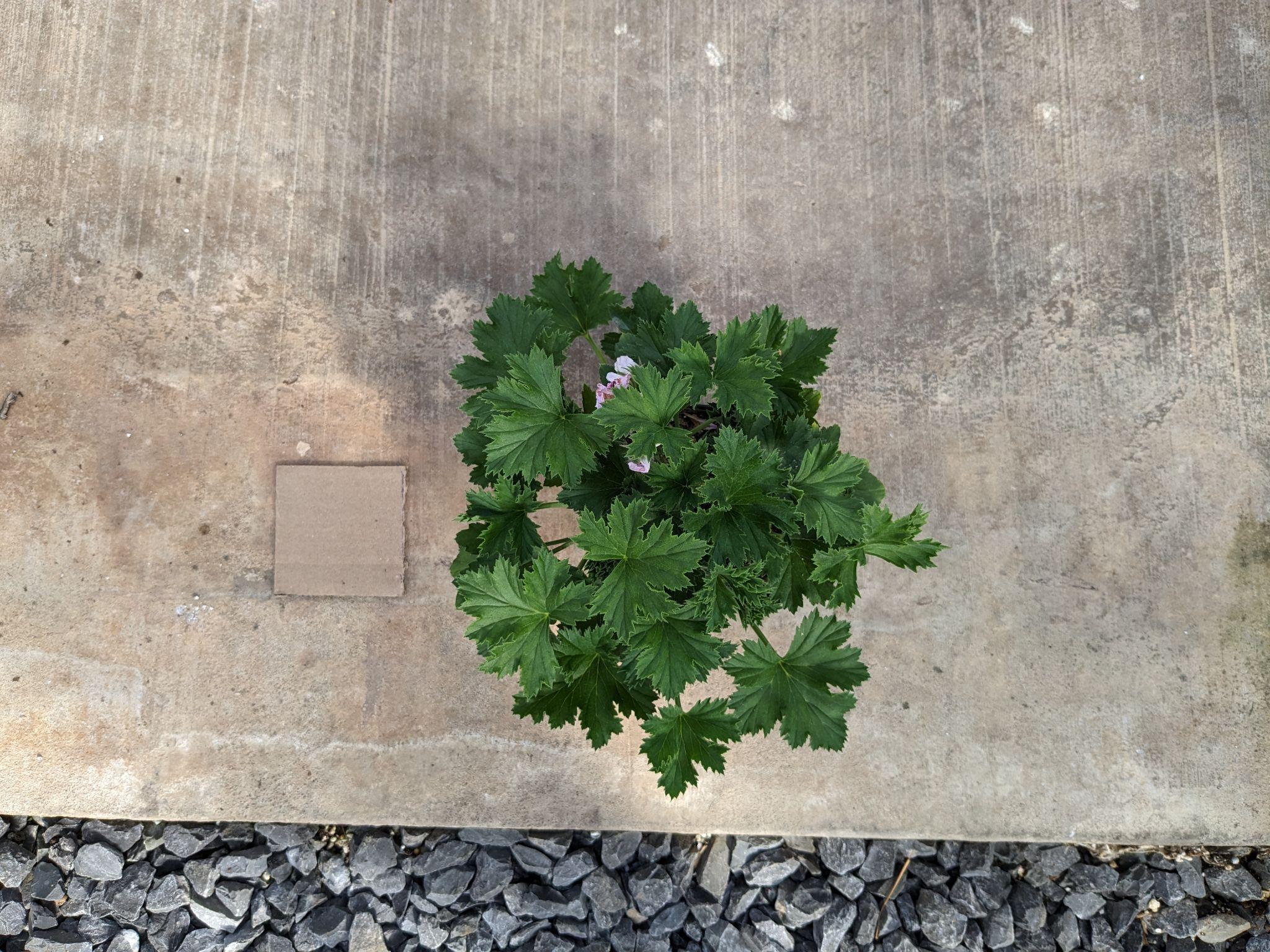 |
| 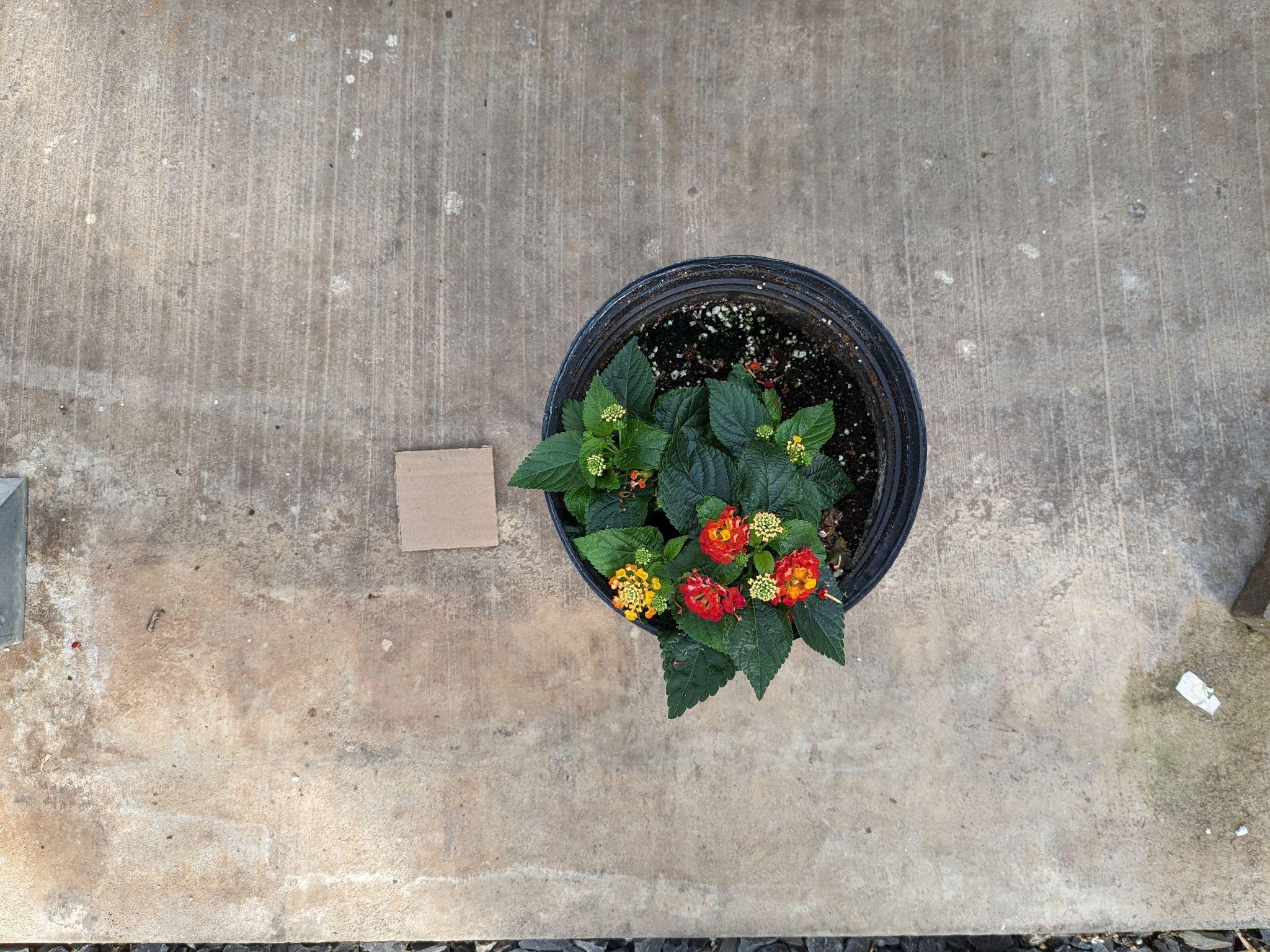 | 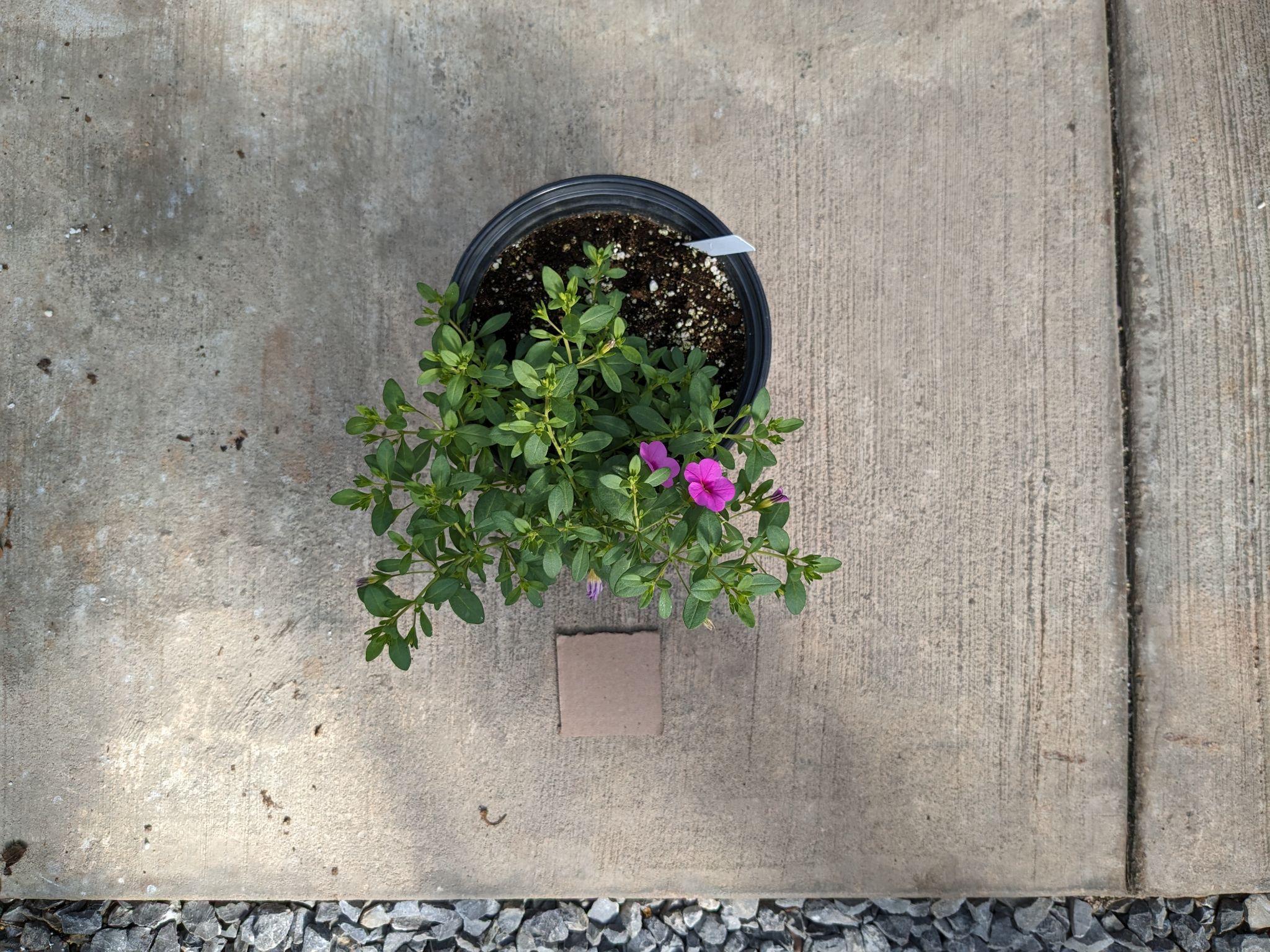 | 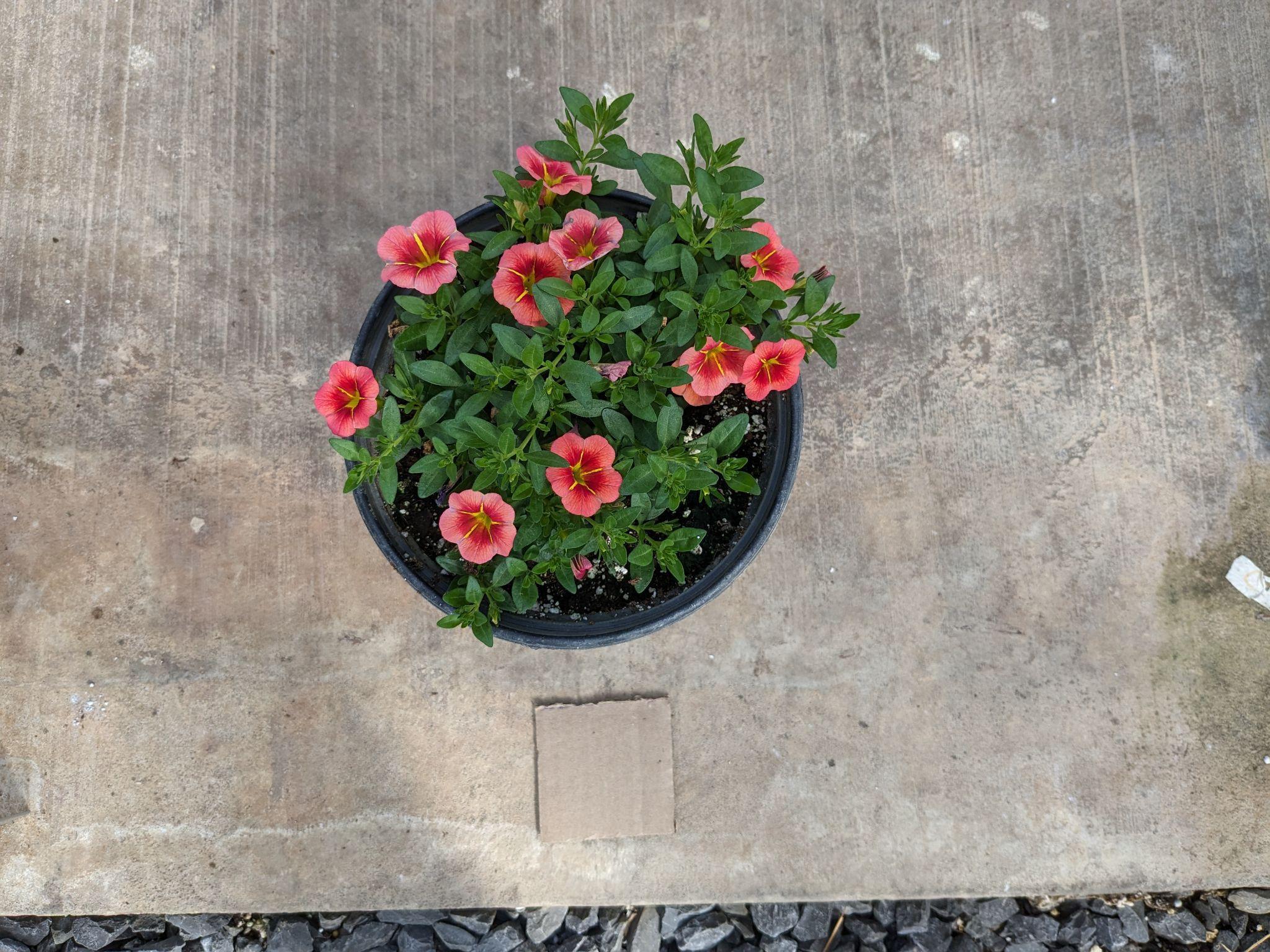 | 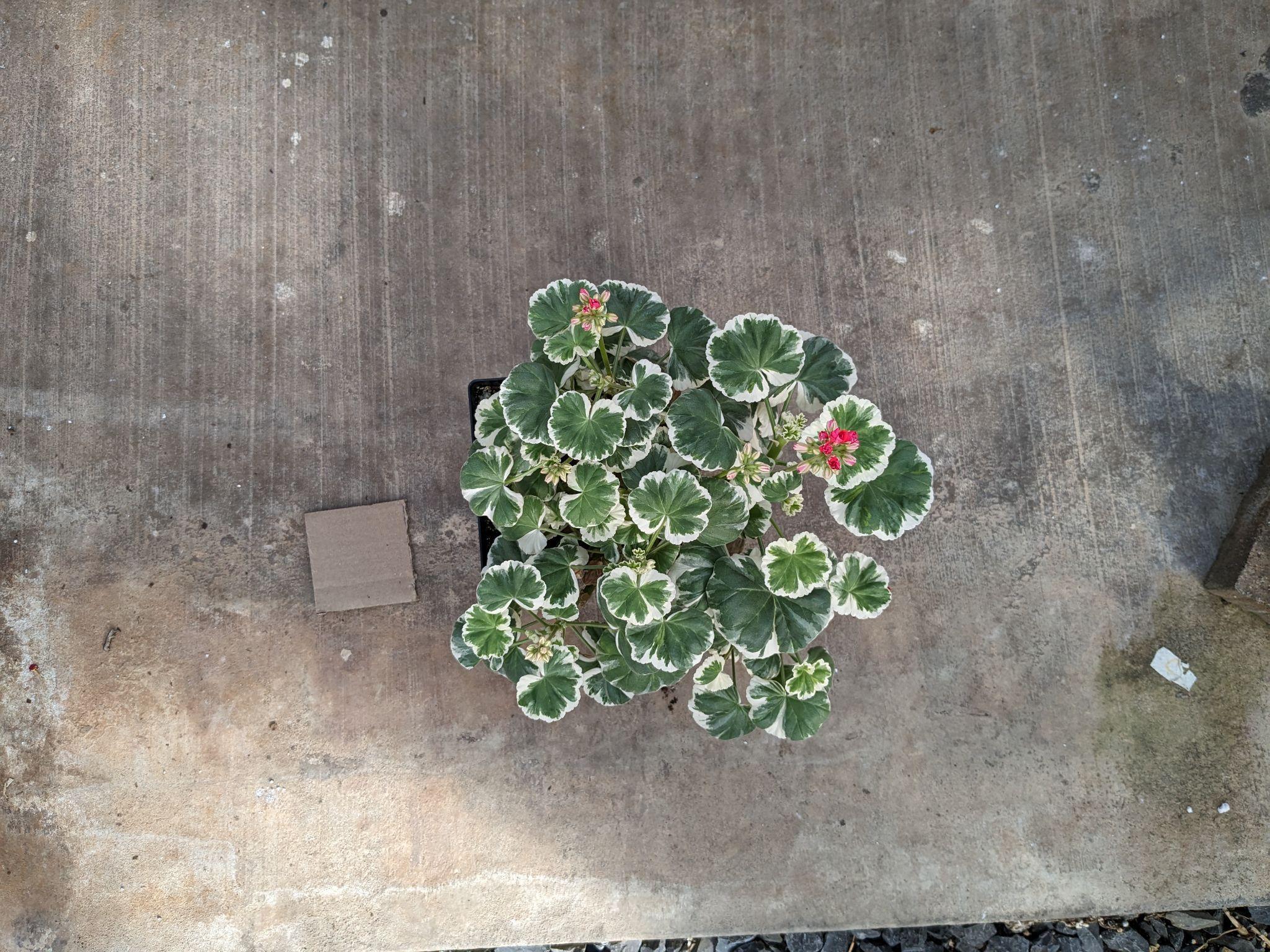 |  |
| 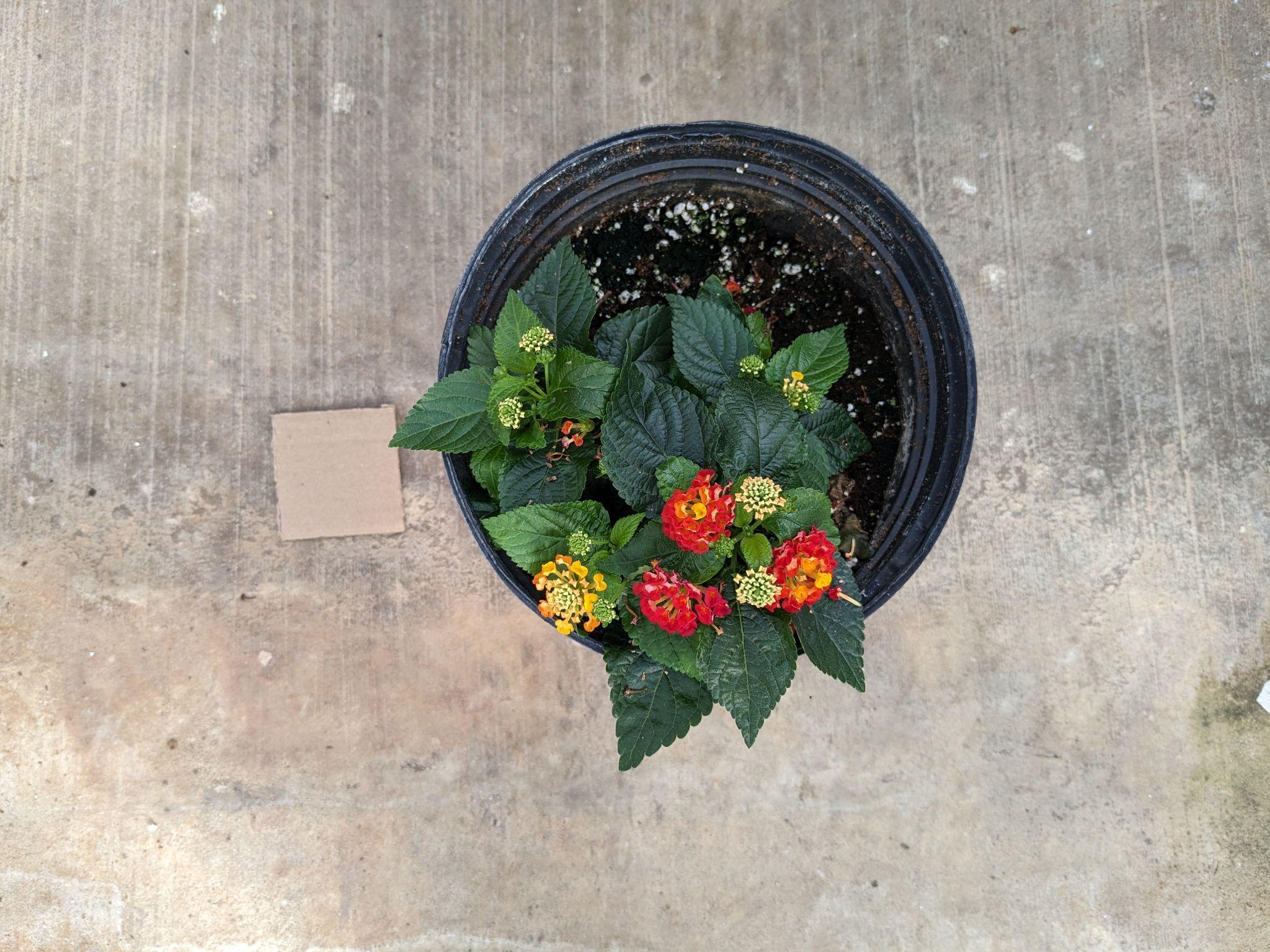 | 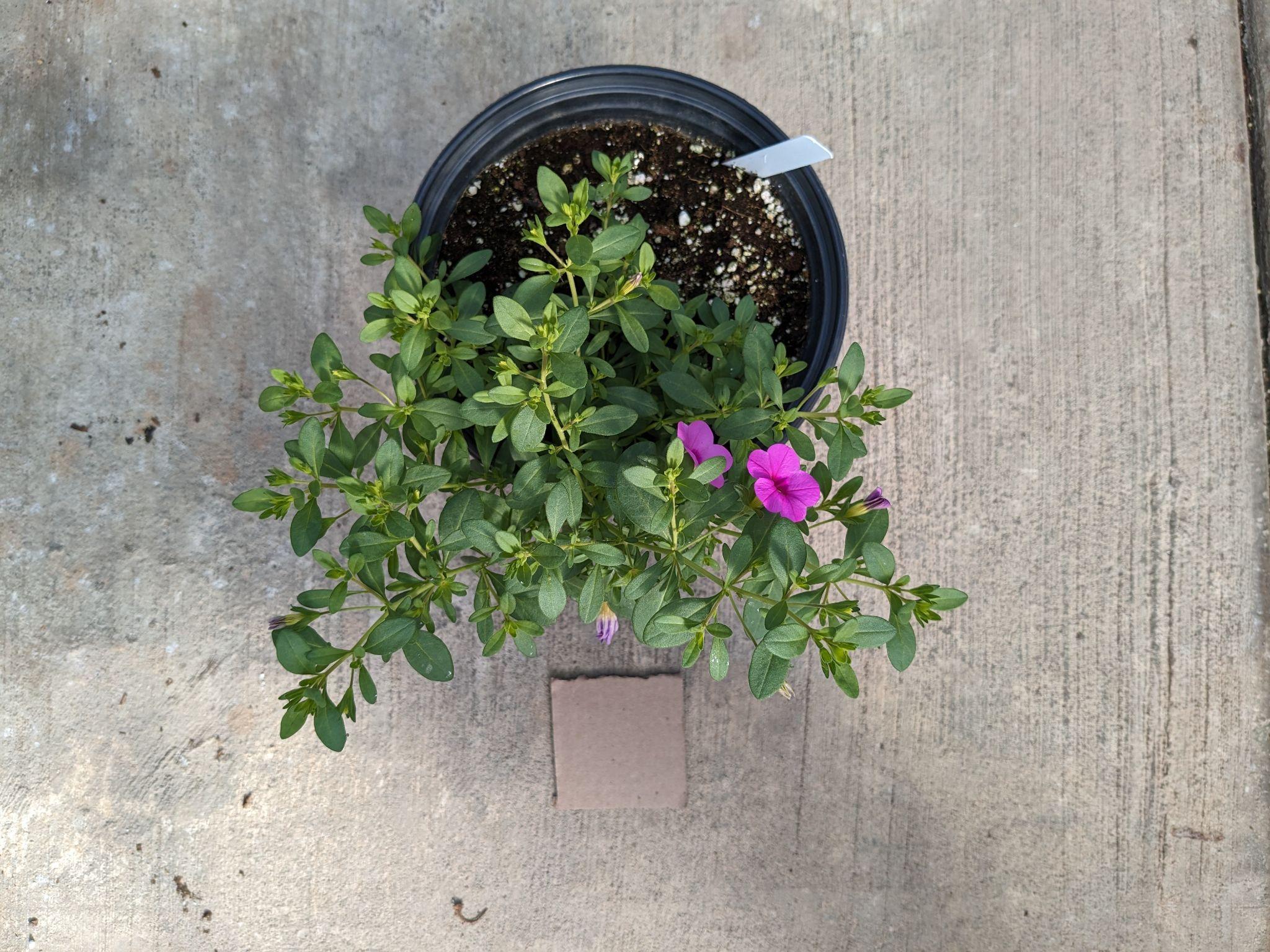 | 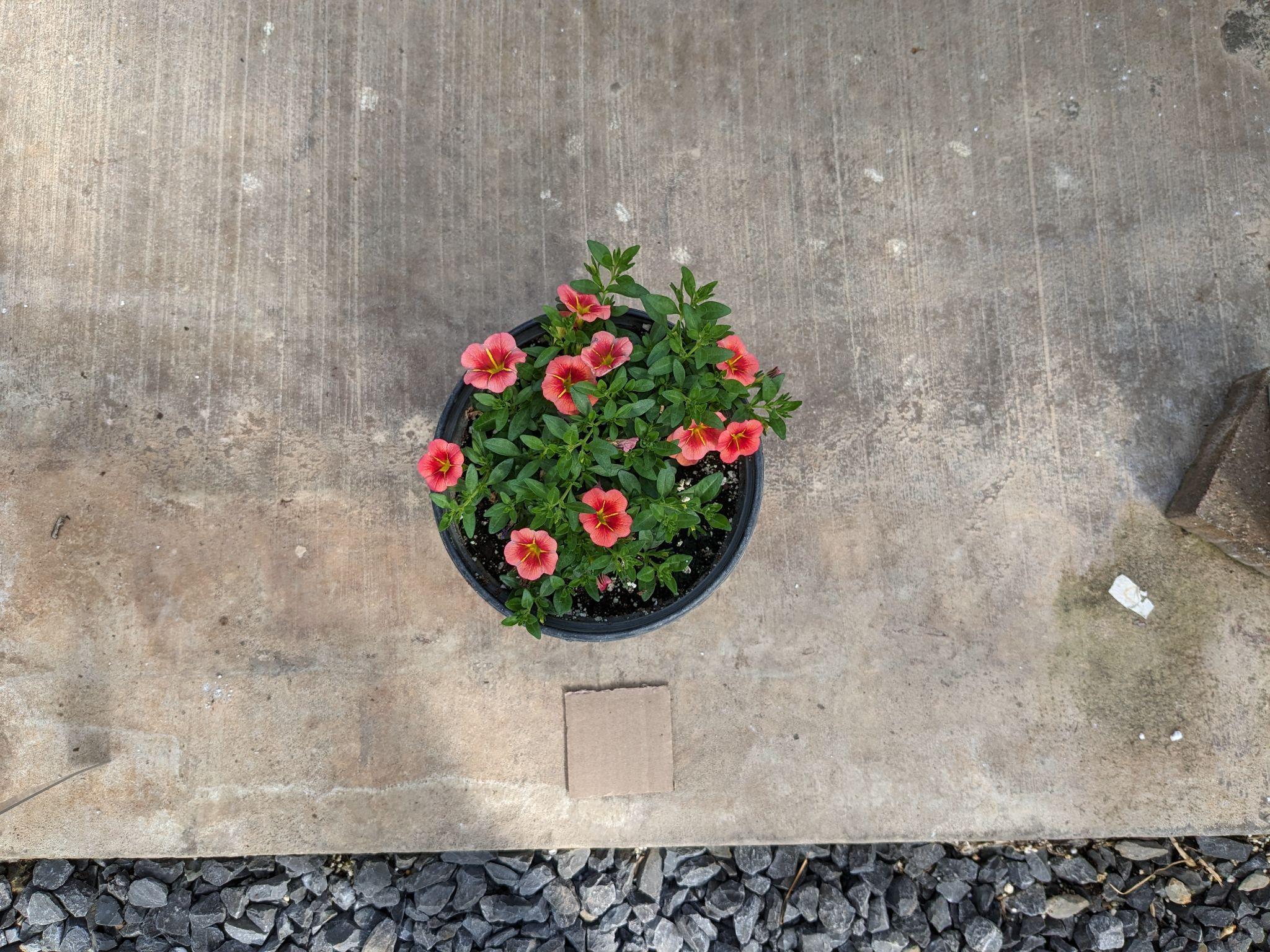 | 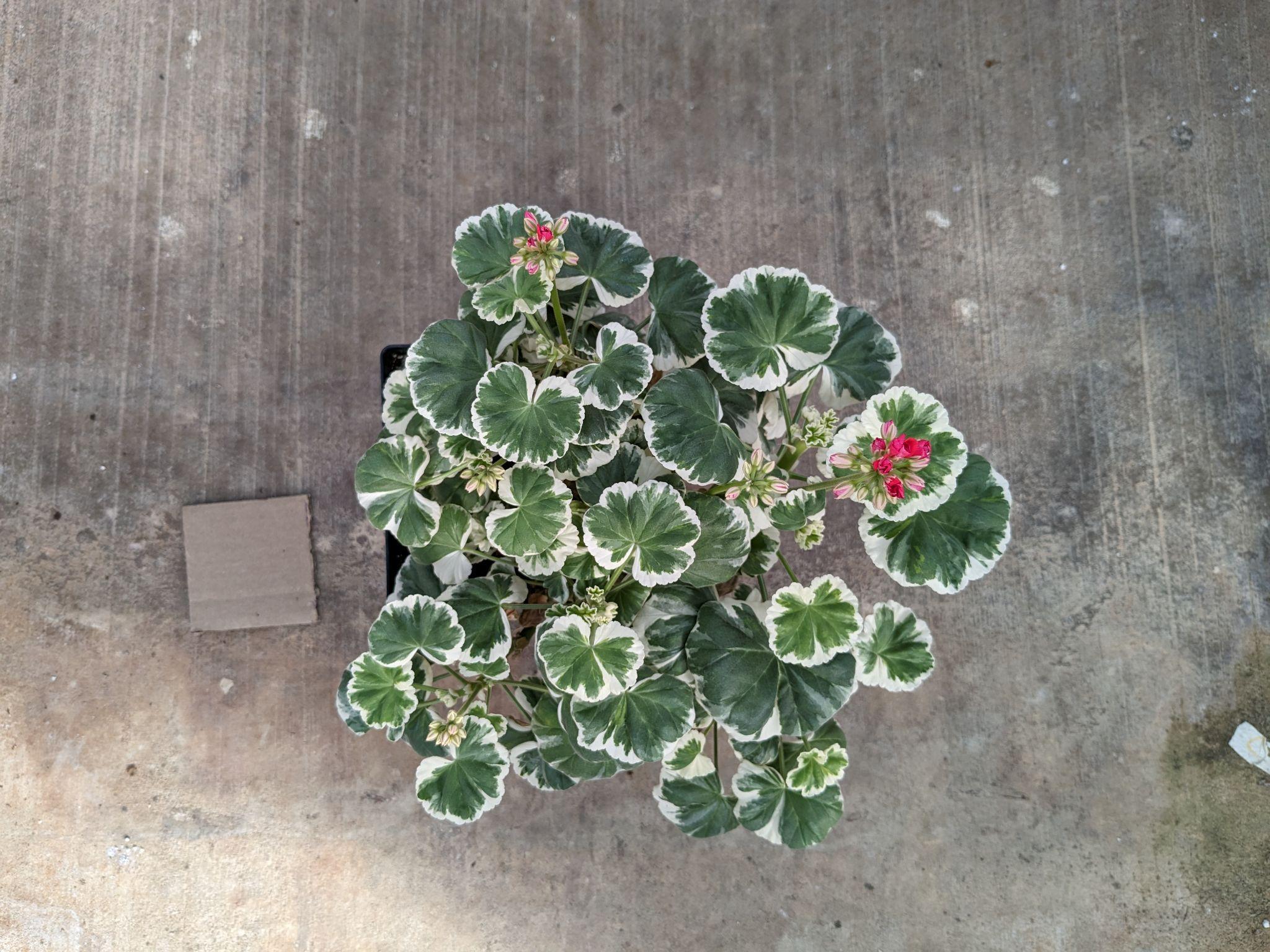 |  |
| 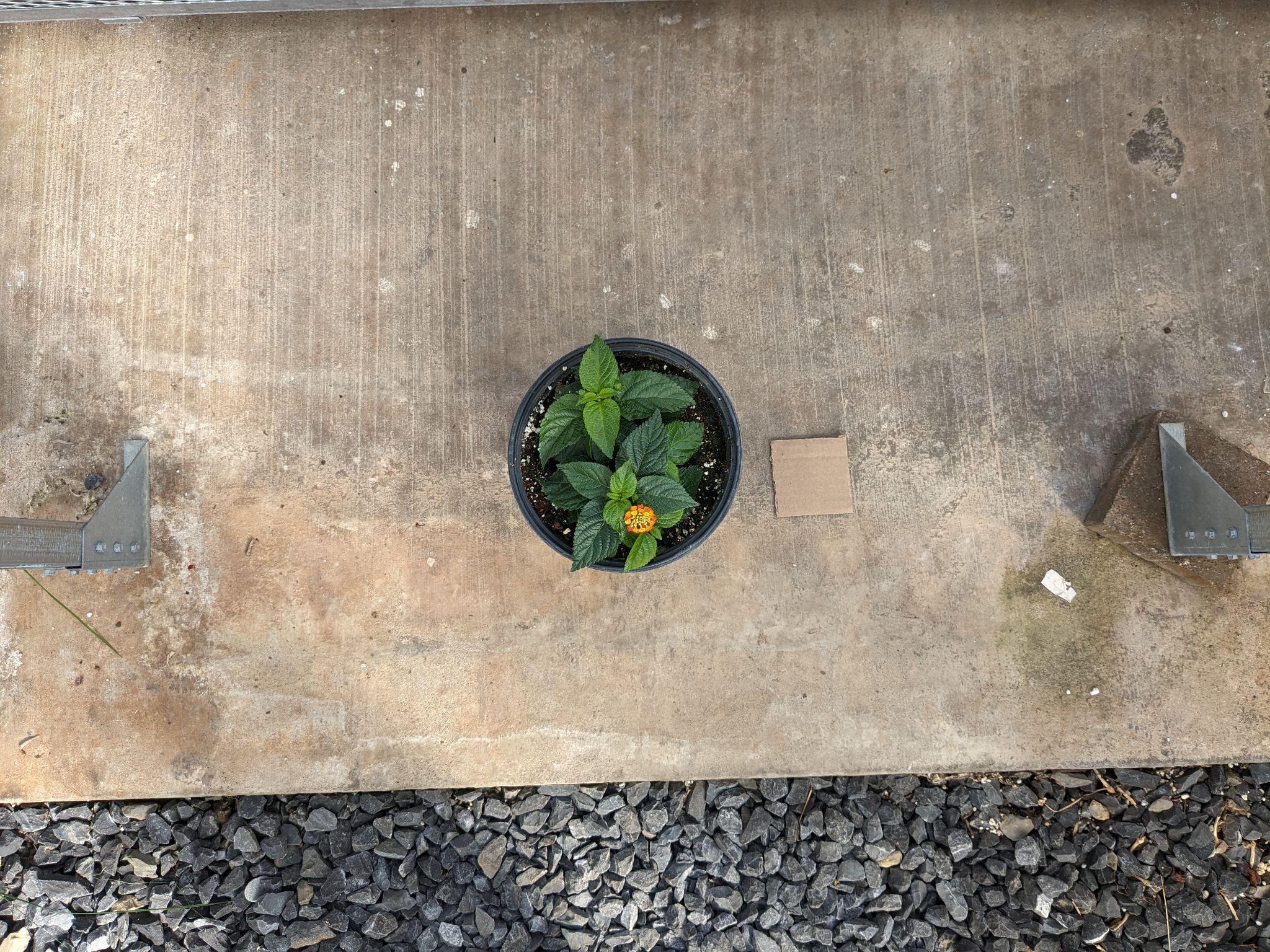 | 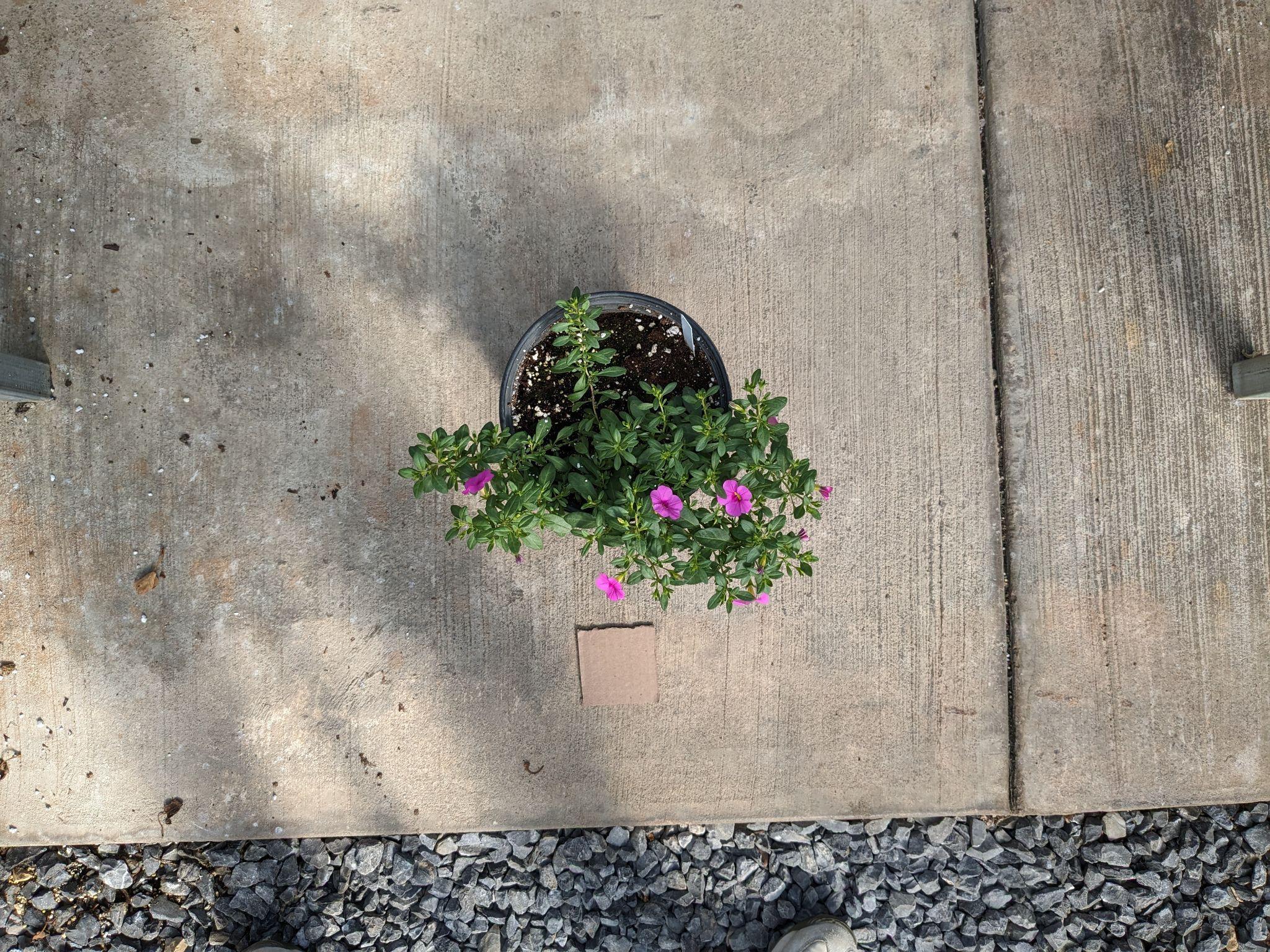 | 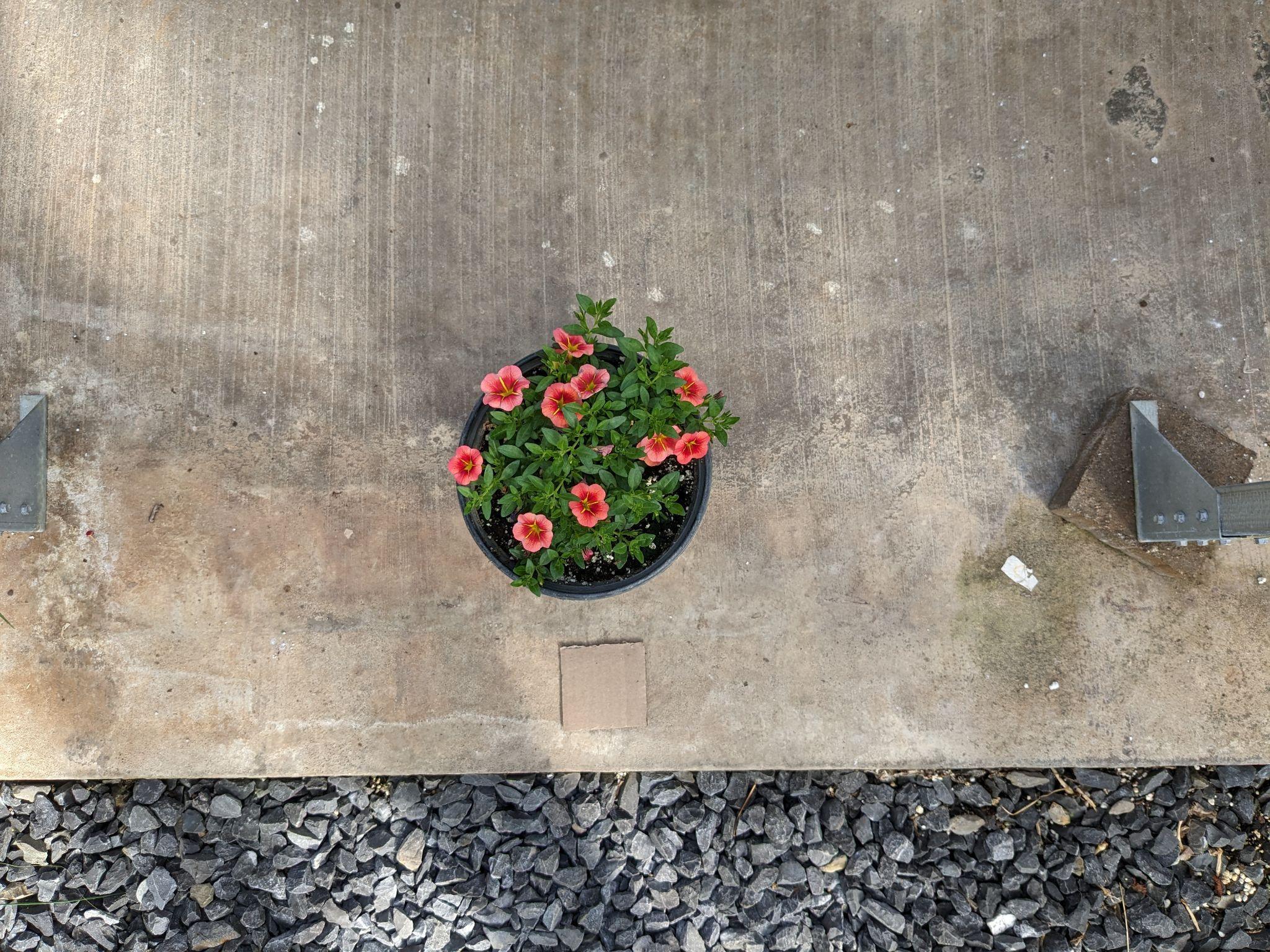 | 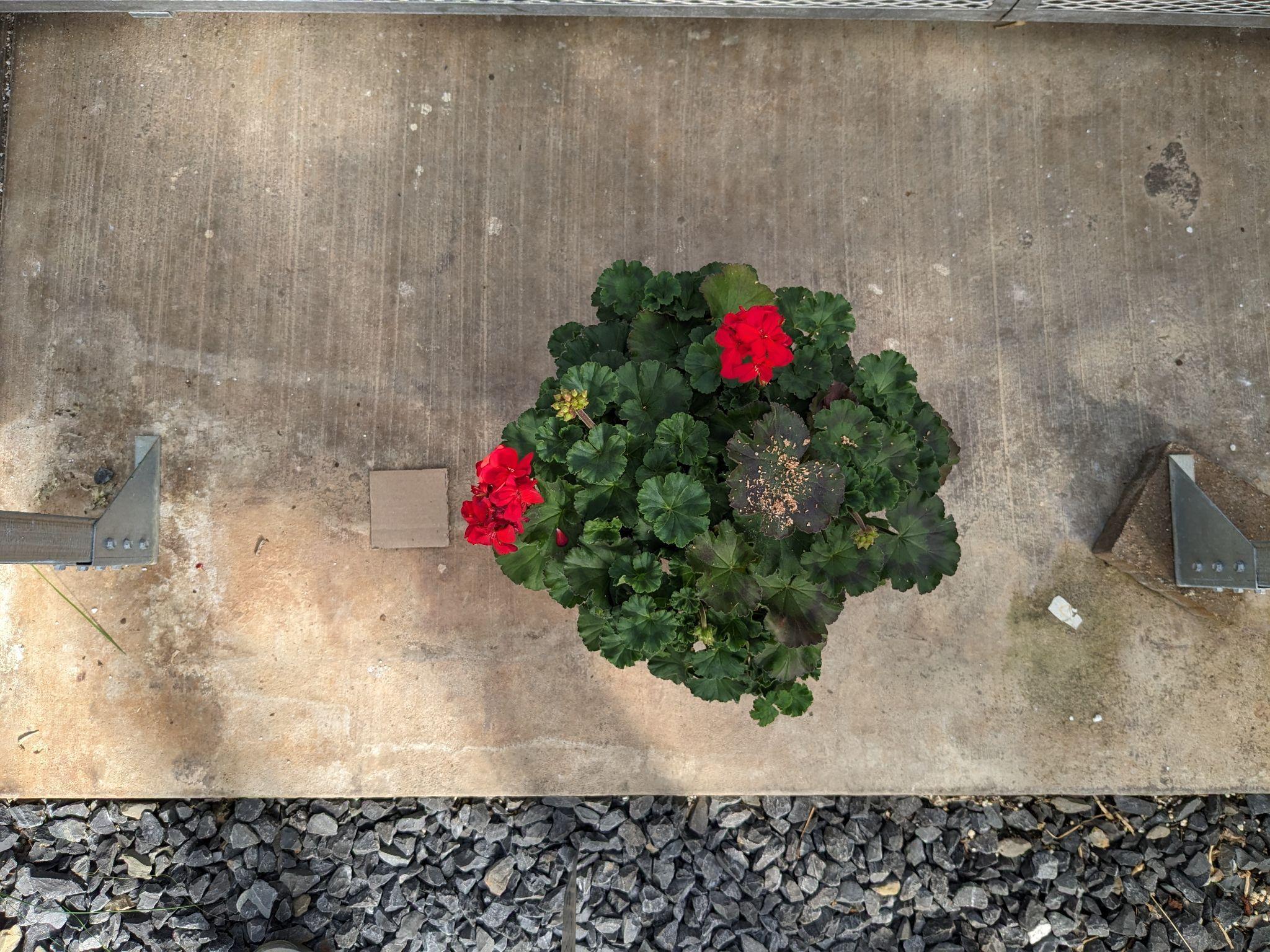 |  |
| 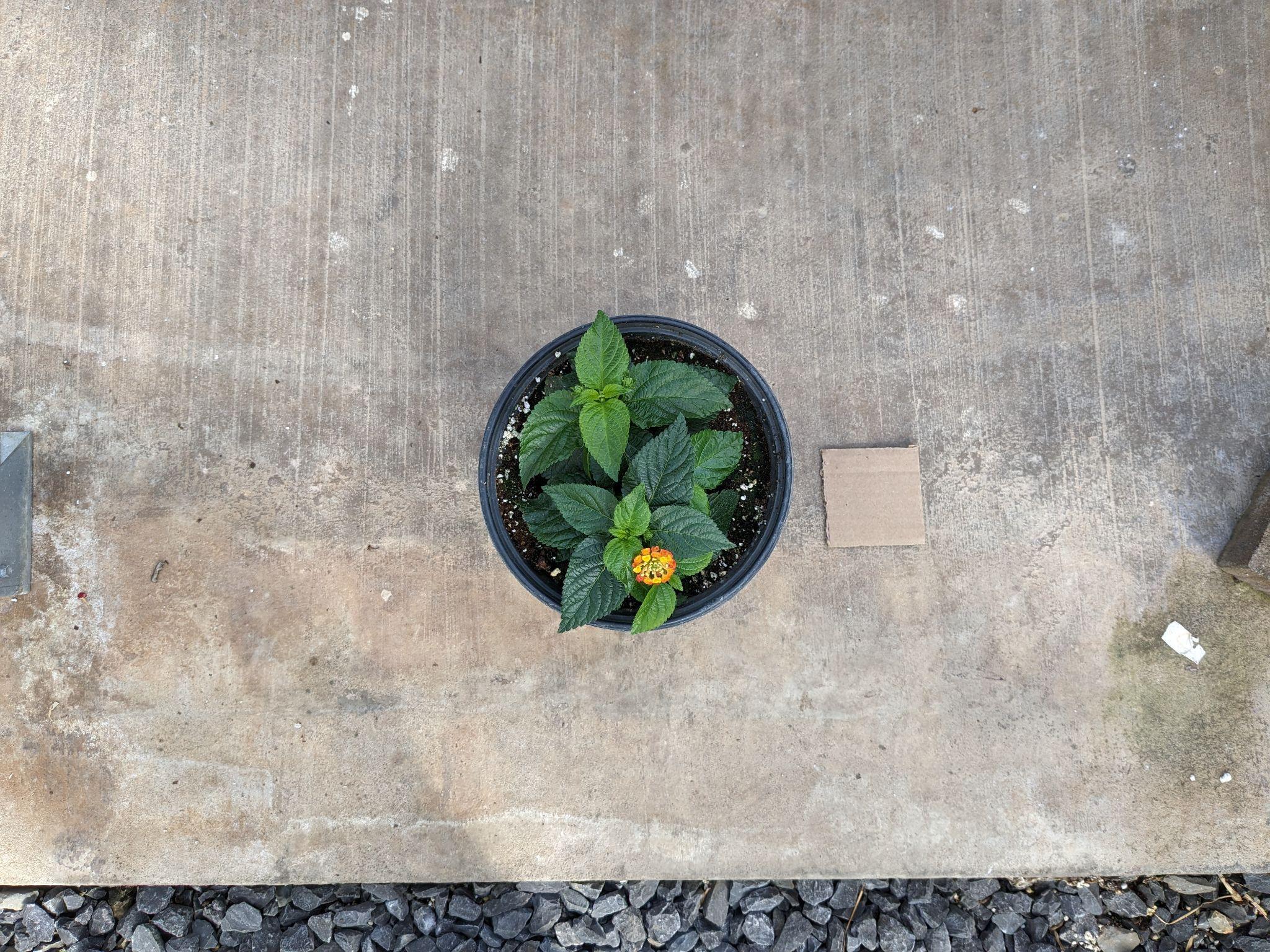 | 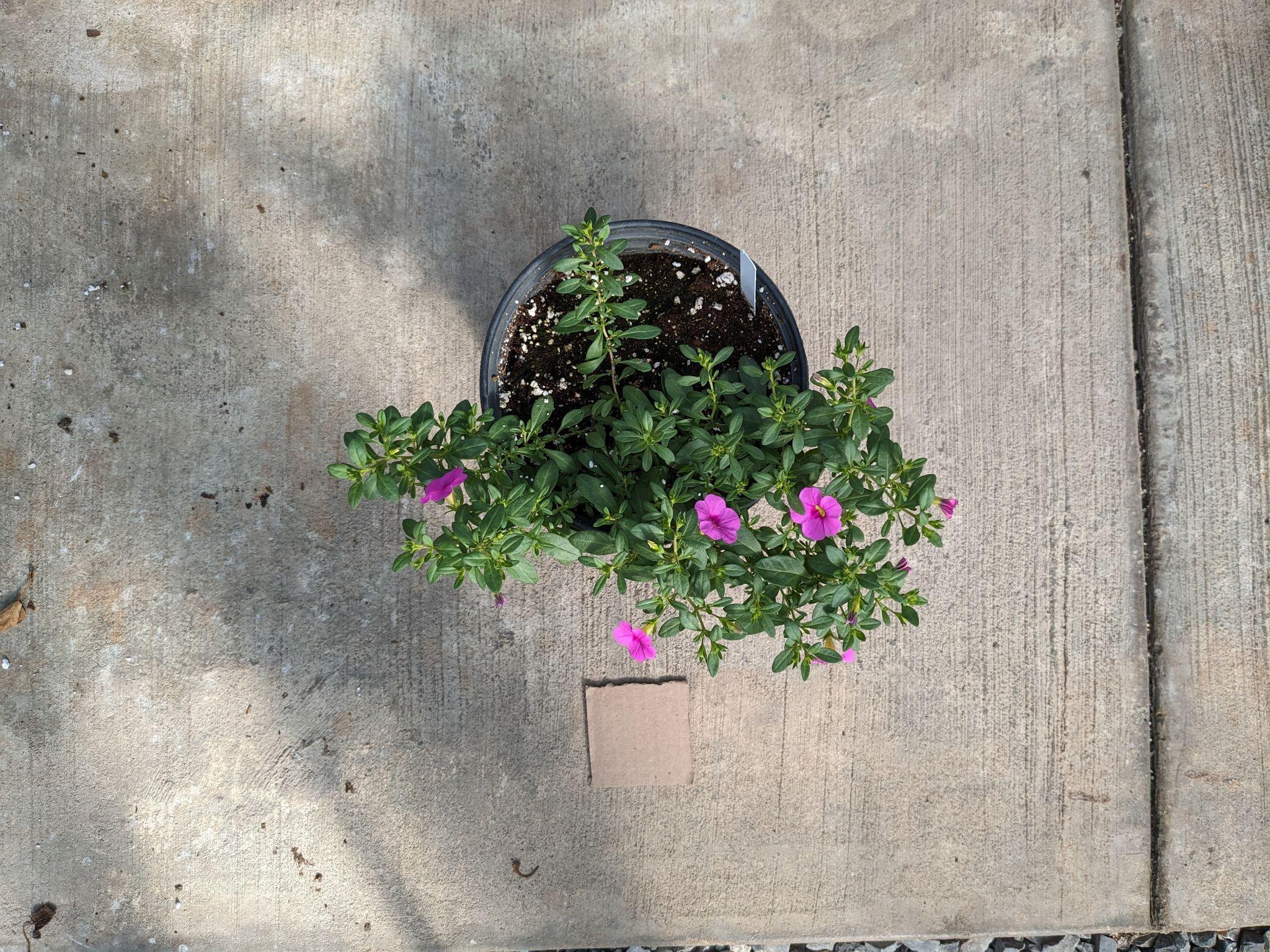 | 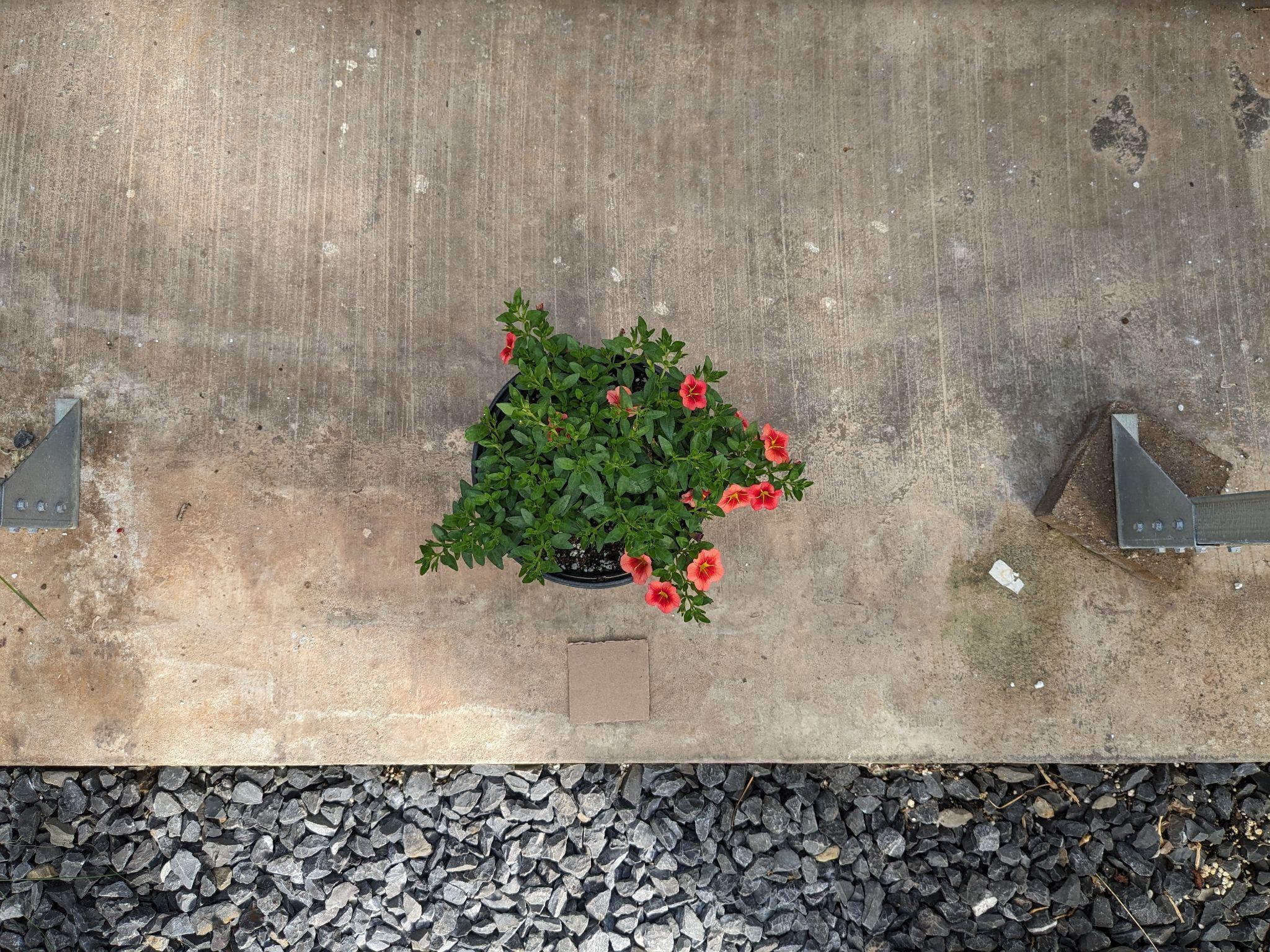 | 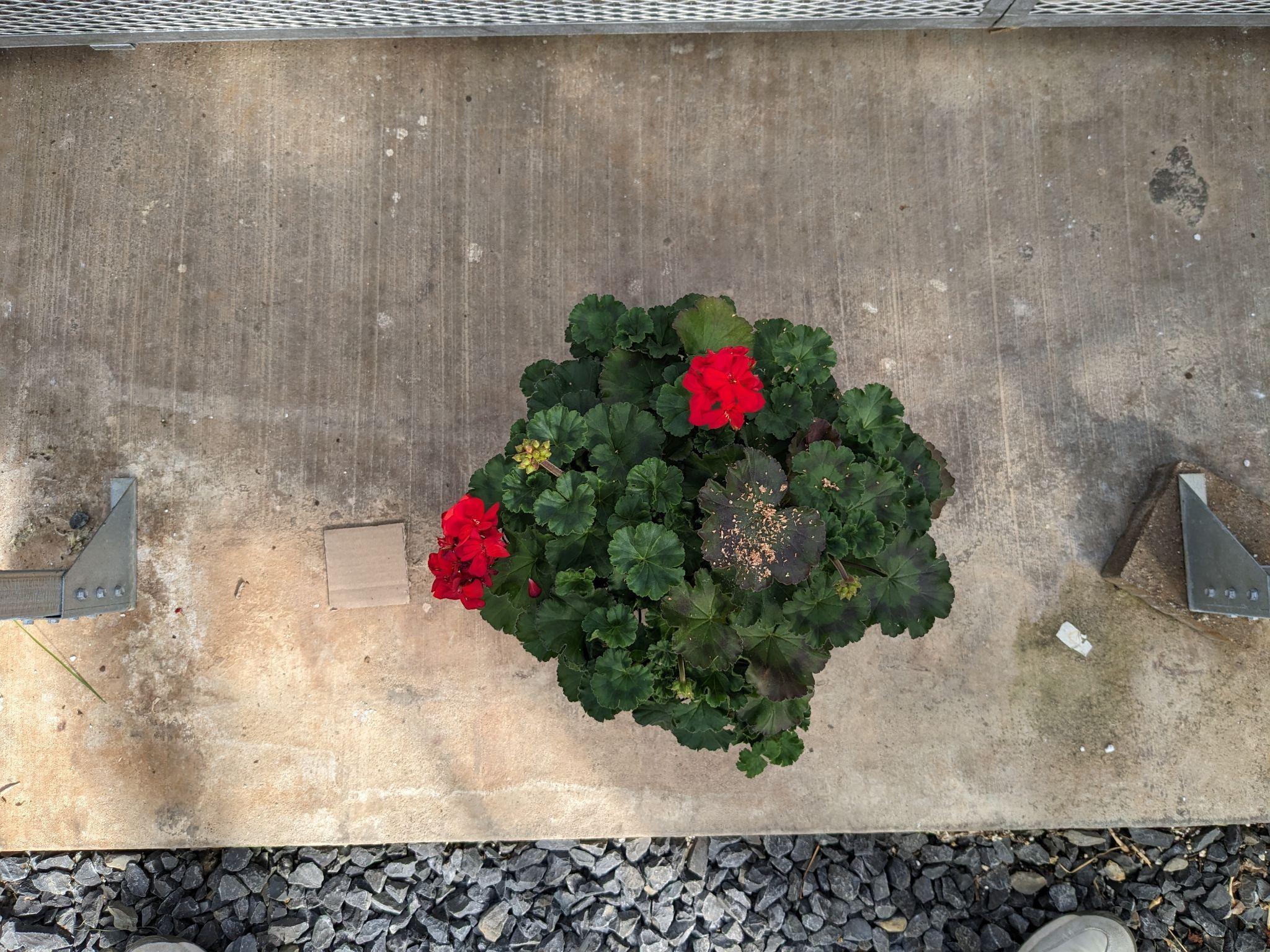 |  |
| 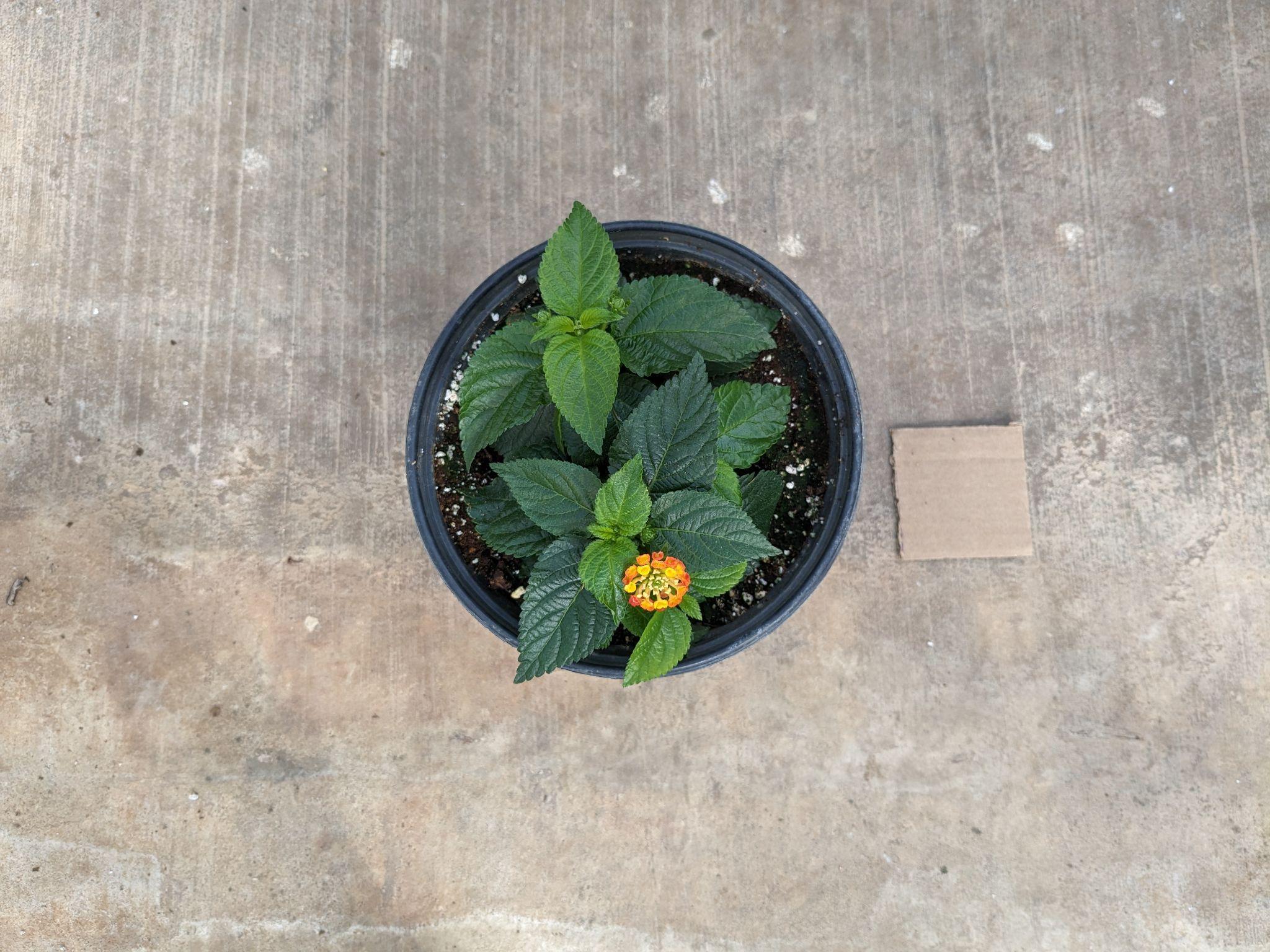 | 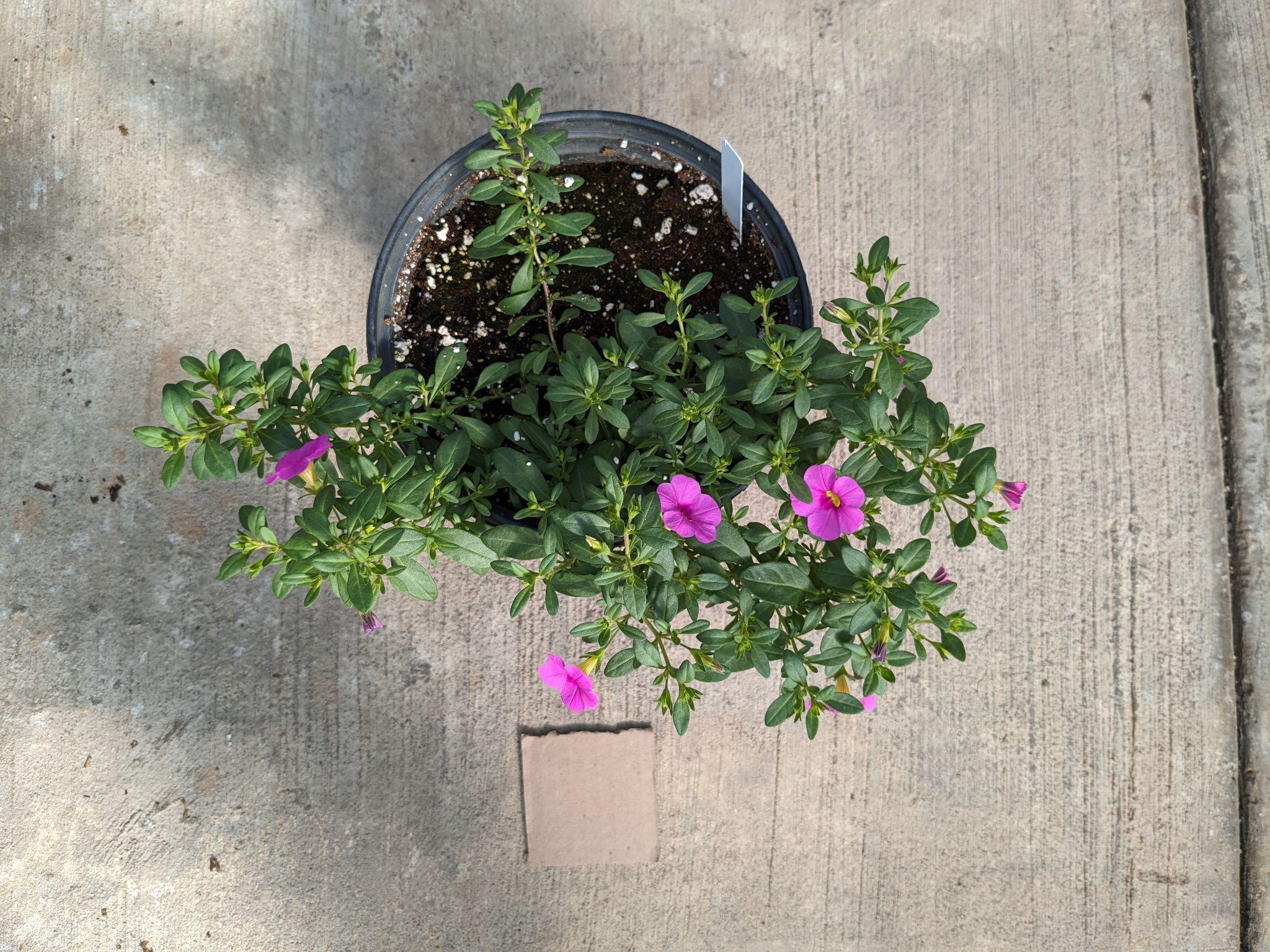 | 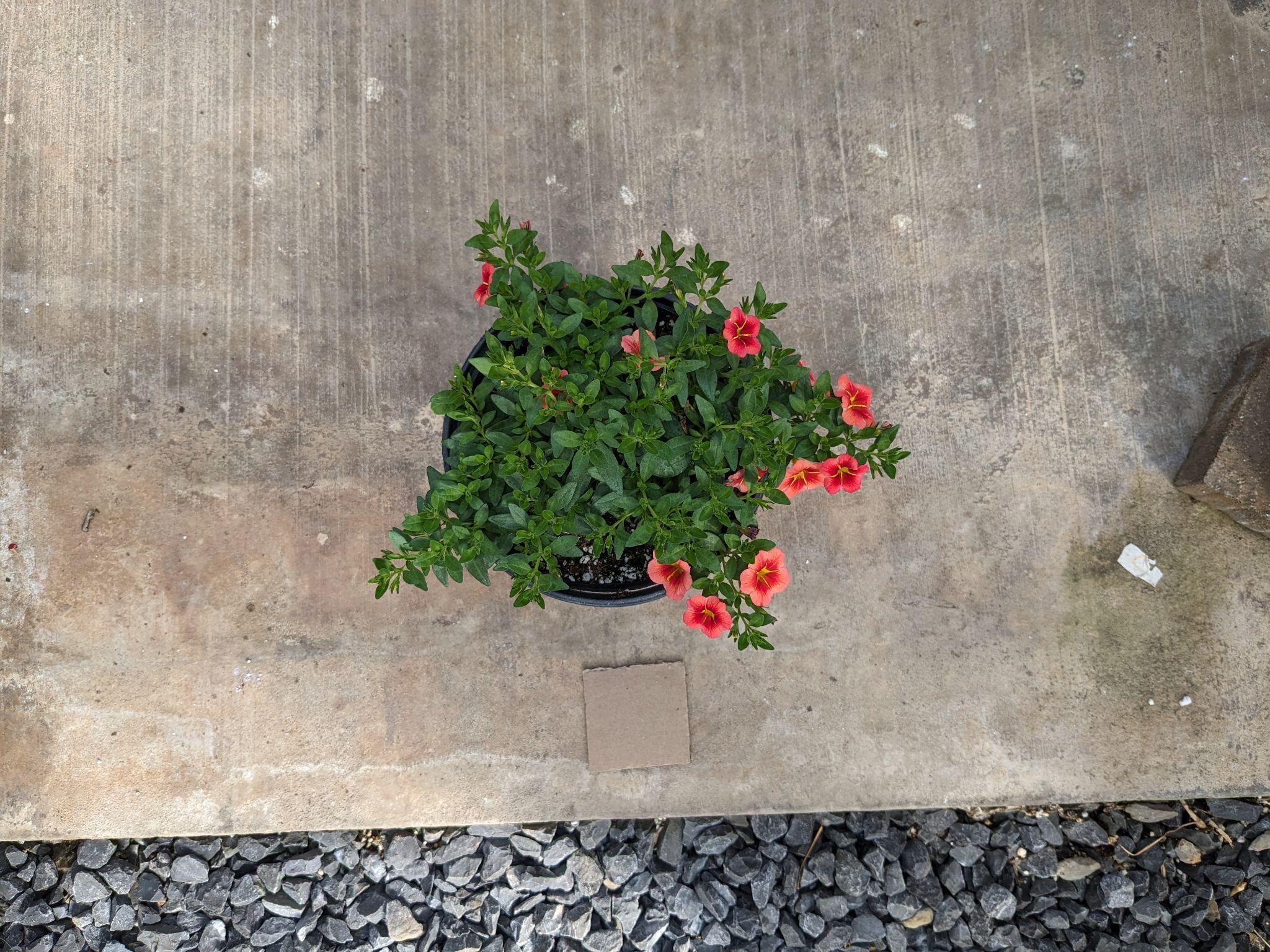 | 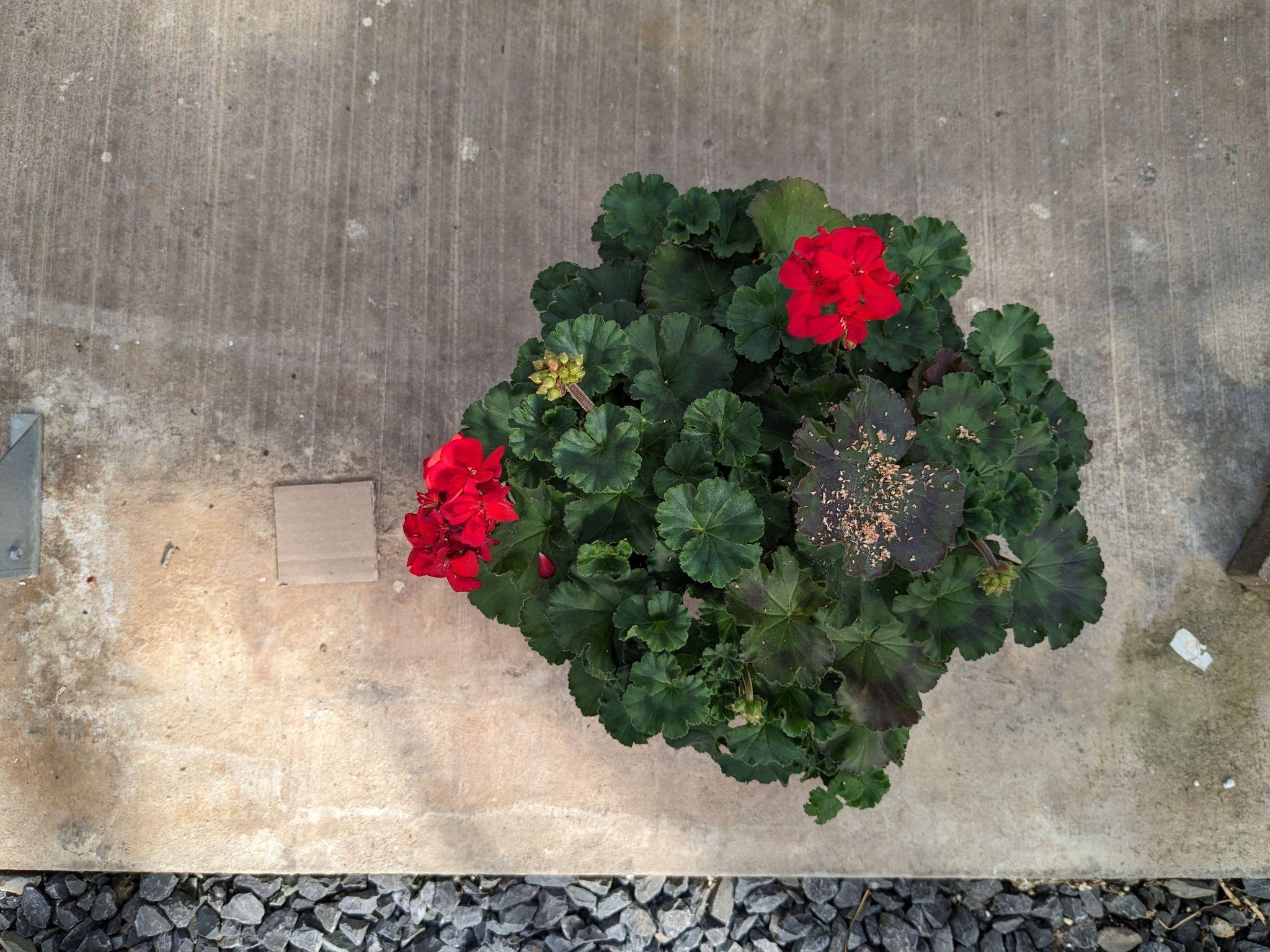 |  |
| 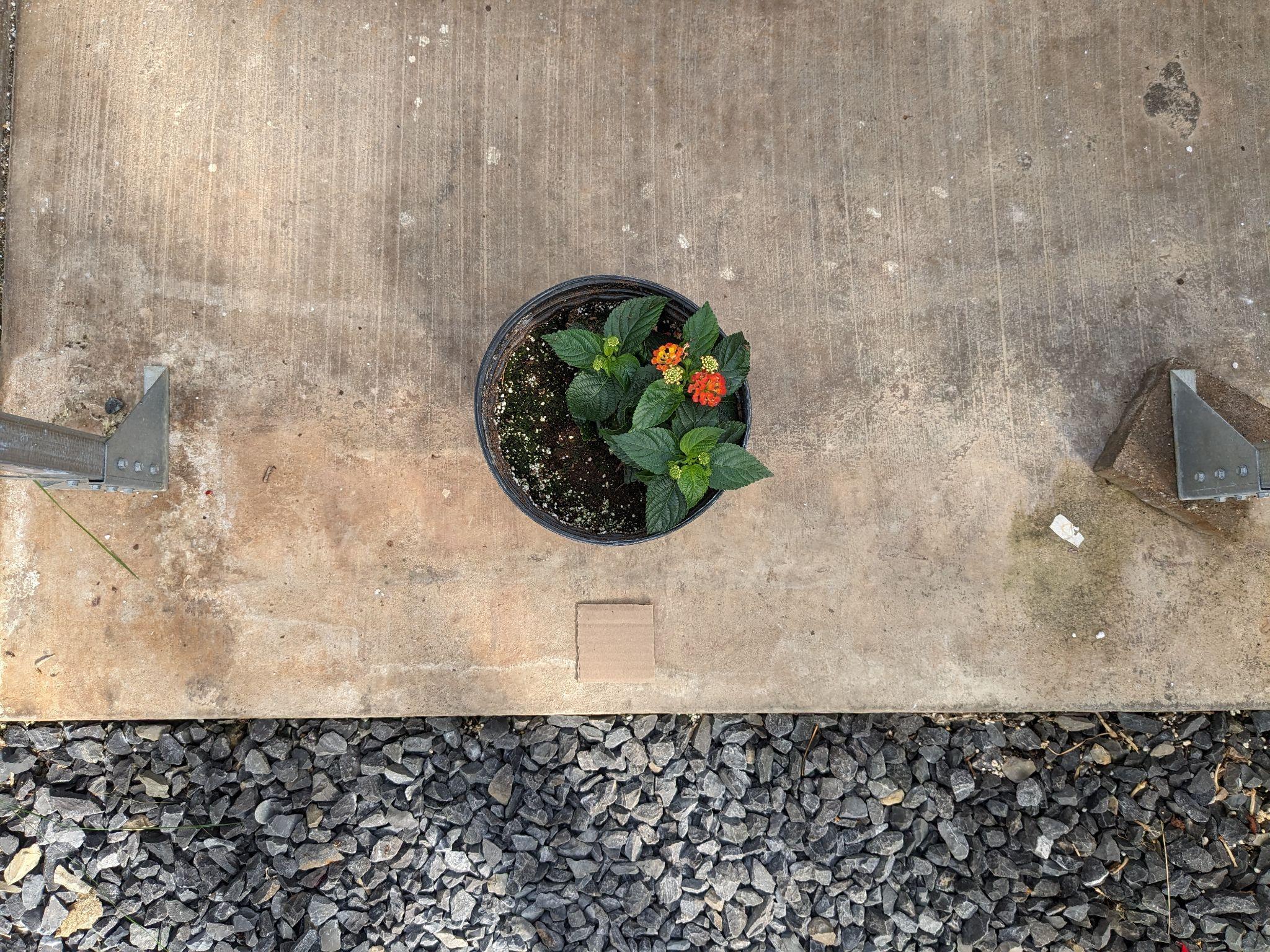 | 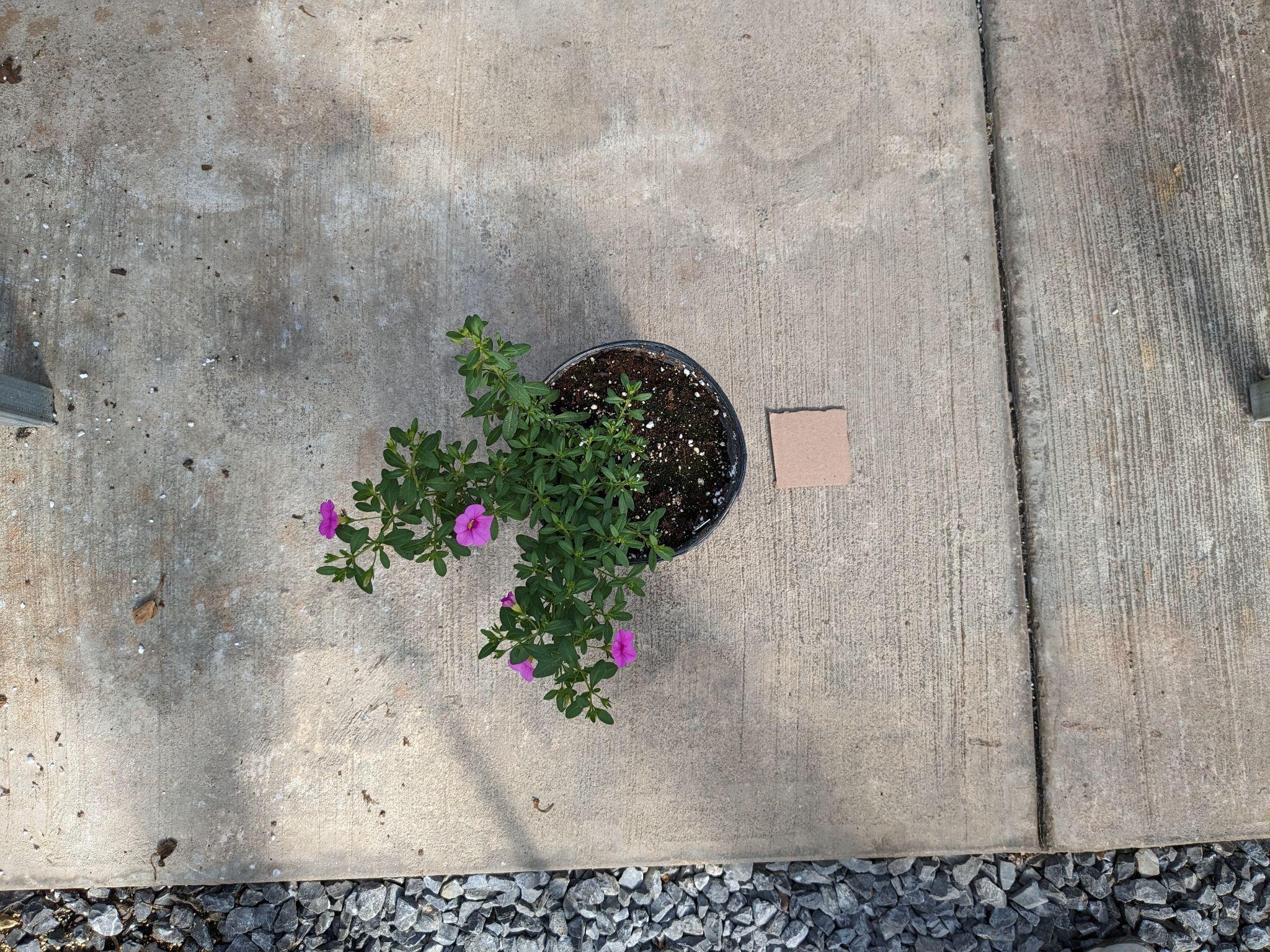 | 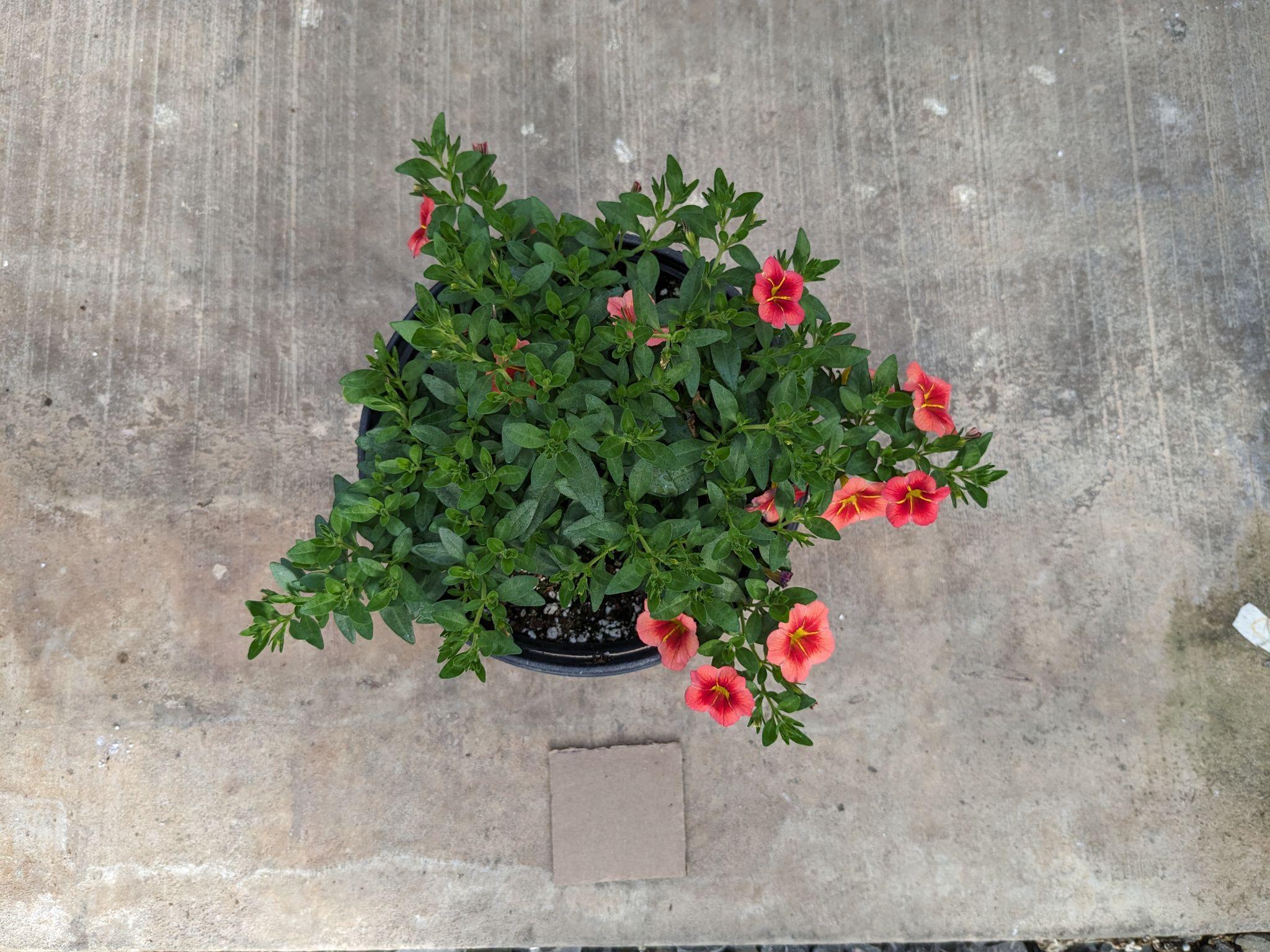 | 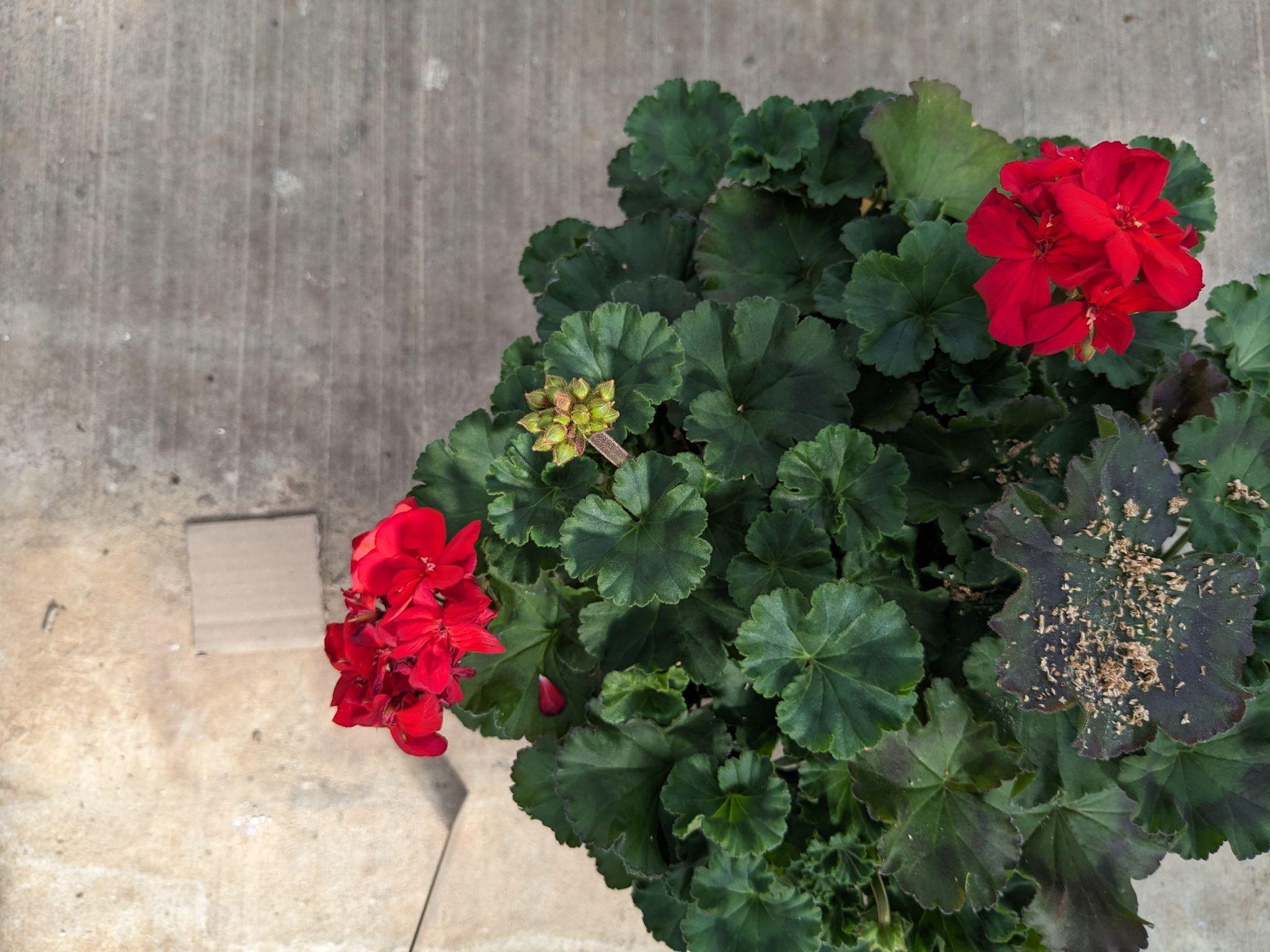 |  |
| 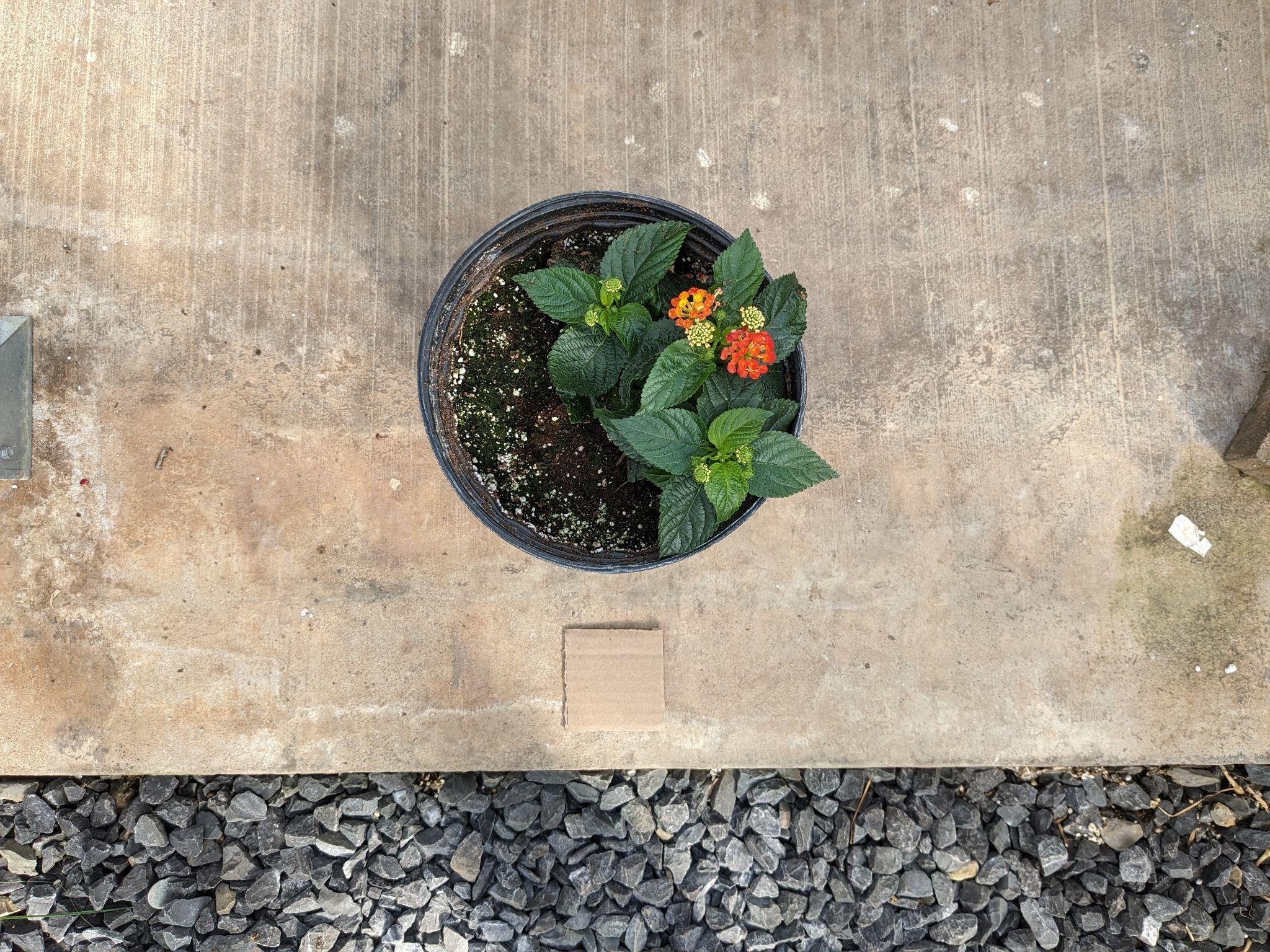 | 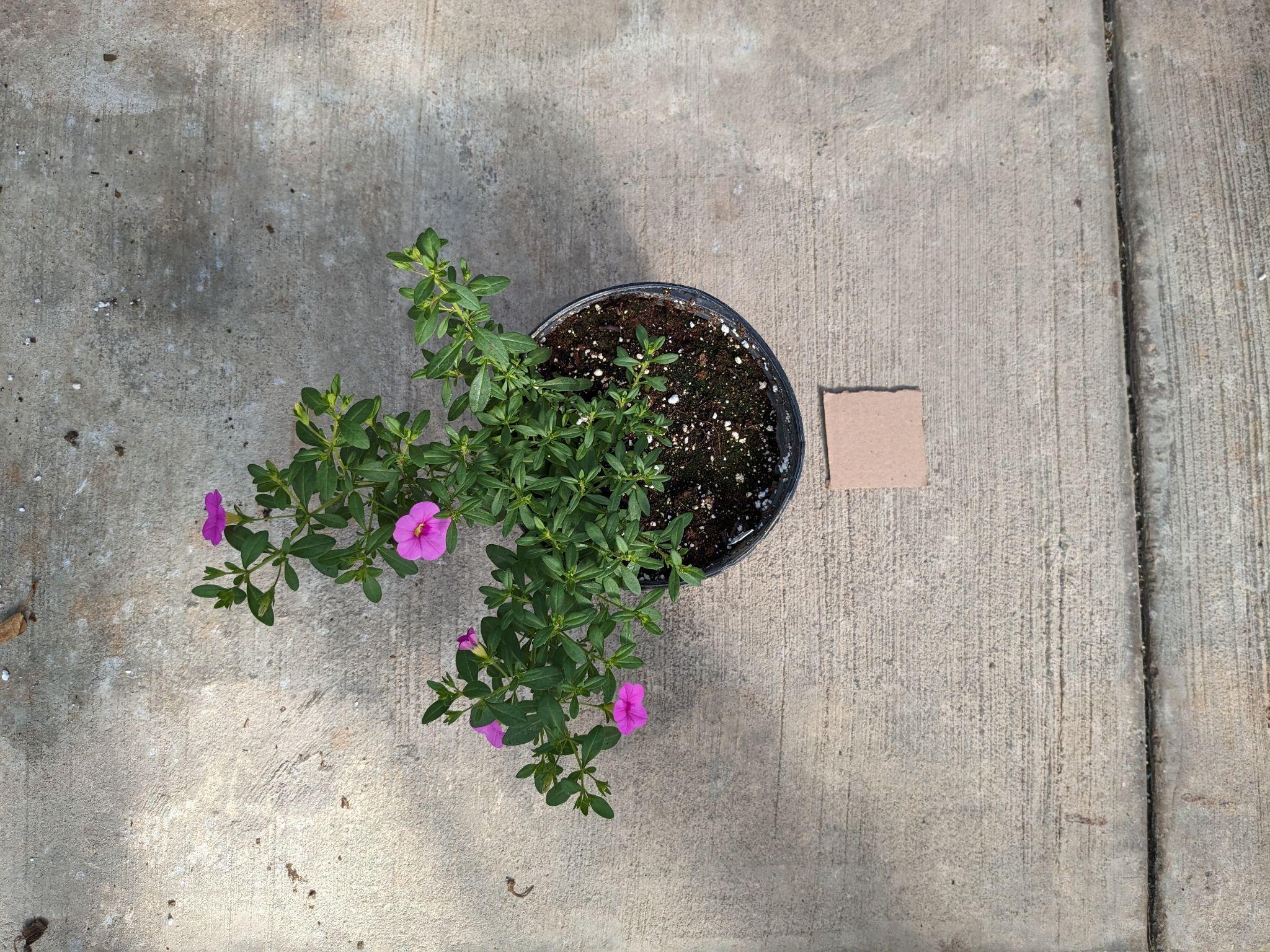 | 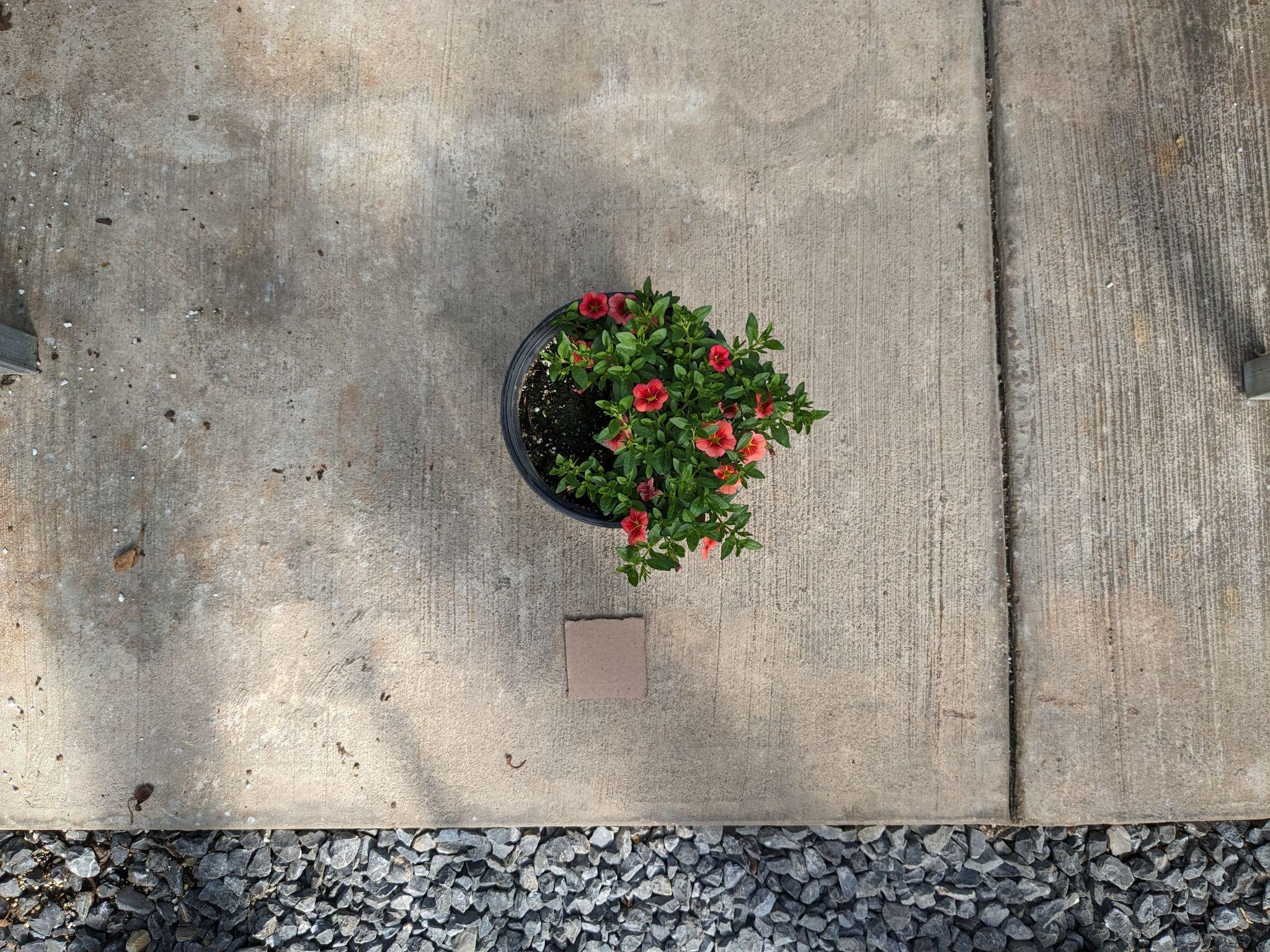 | 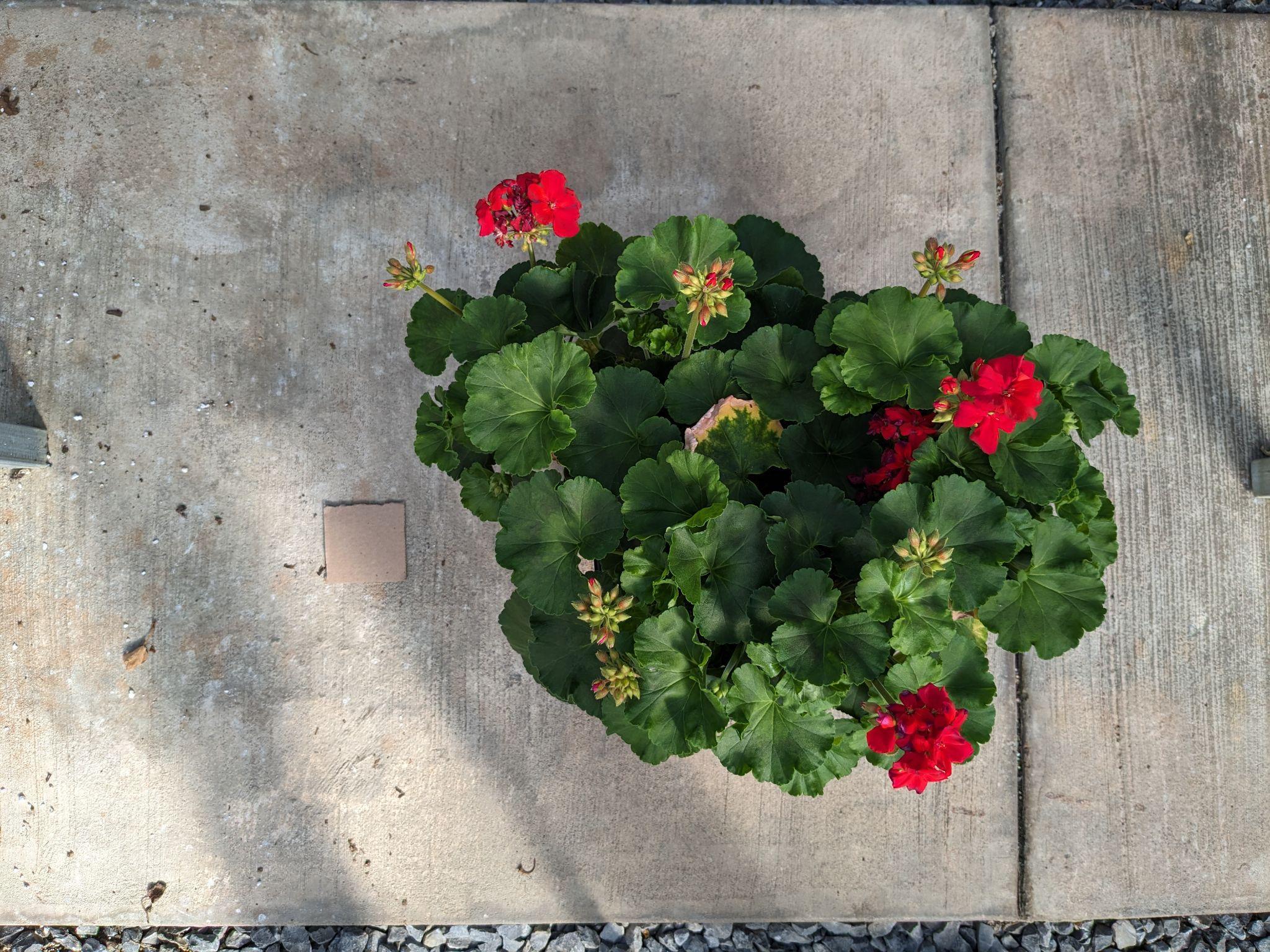 |  |
| 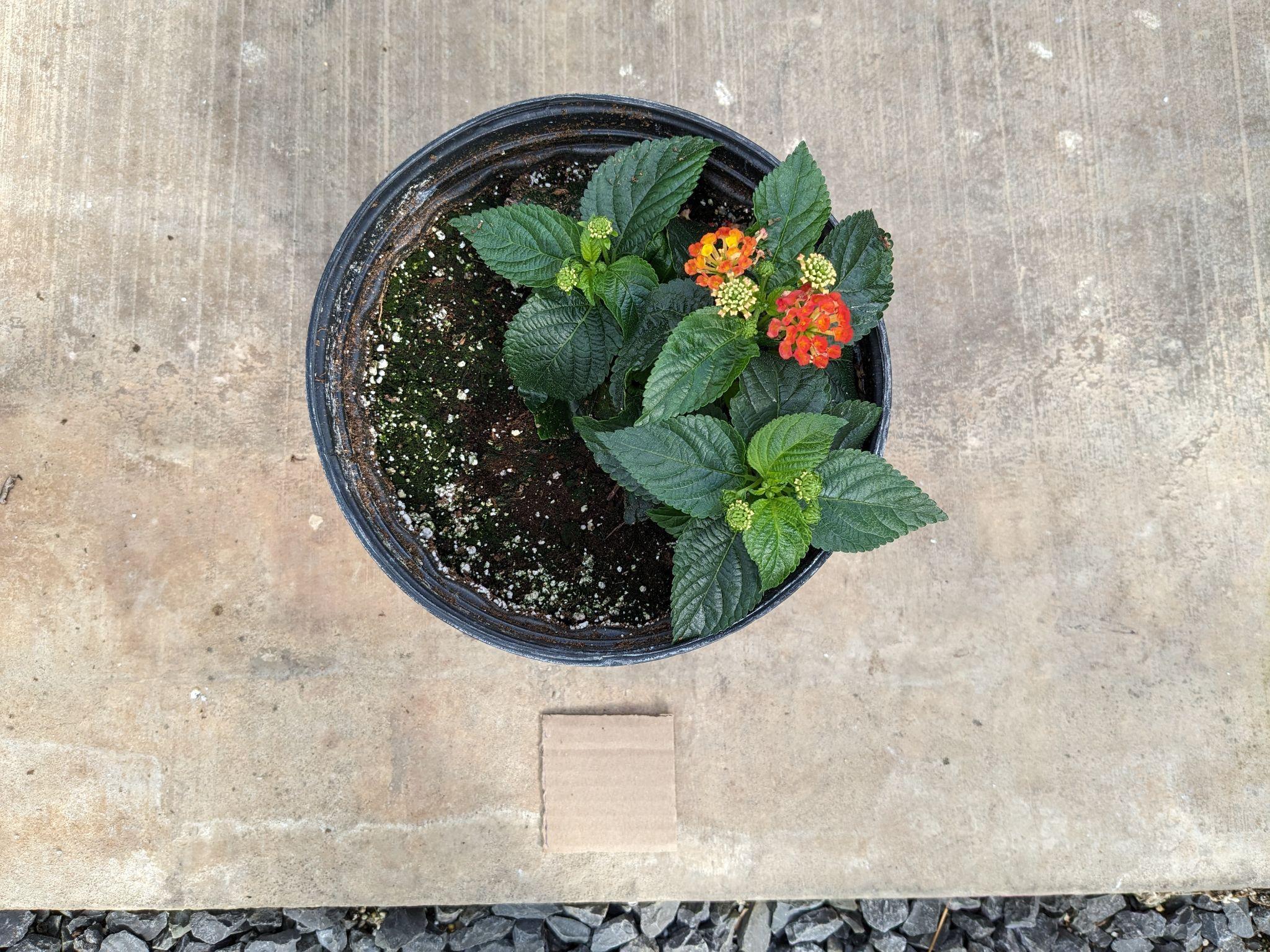 | 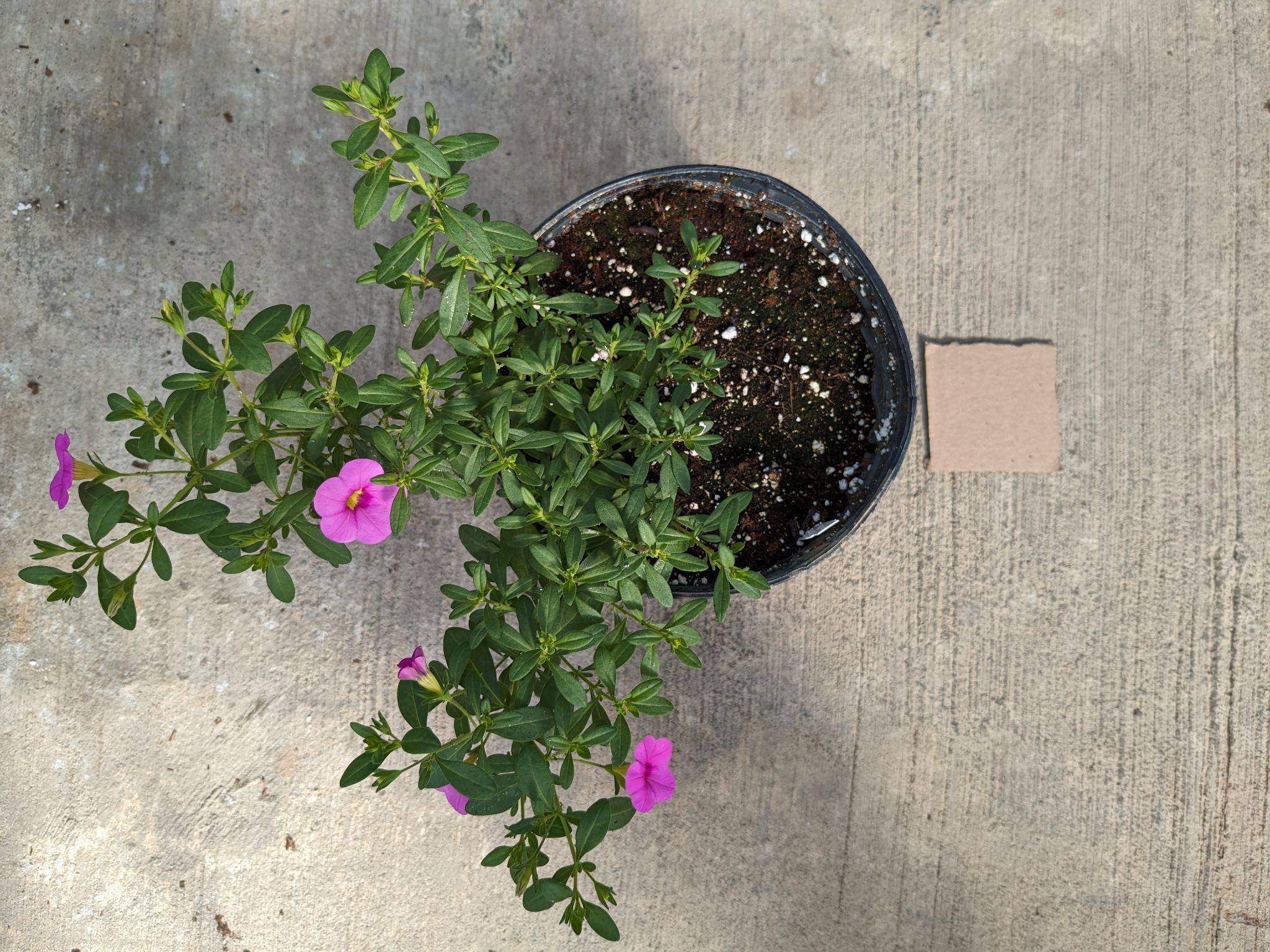 | 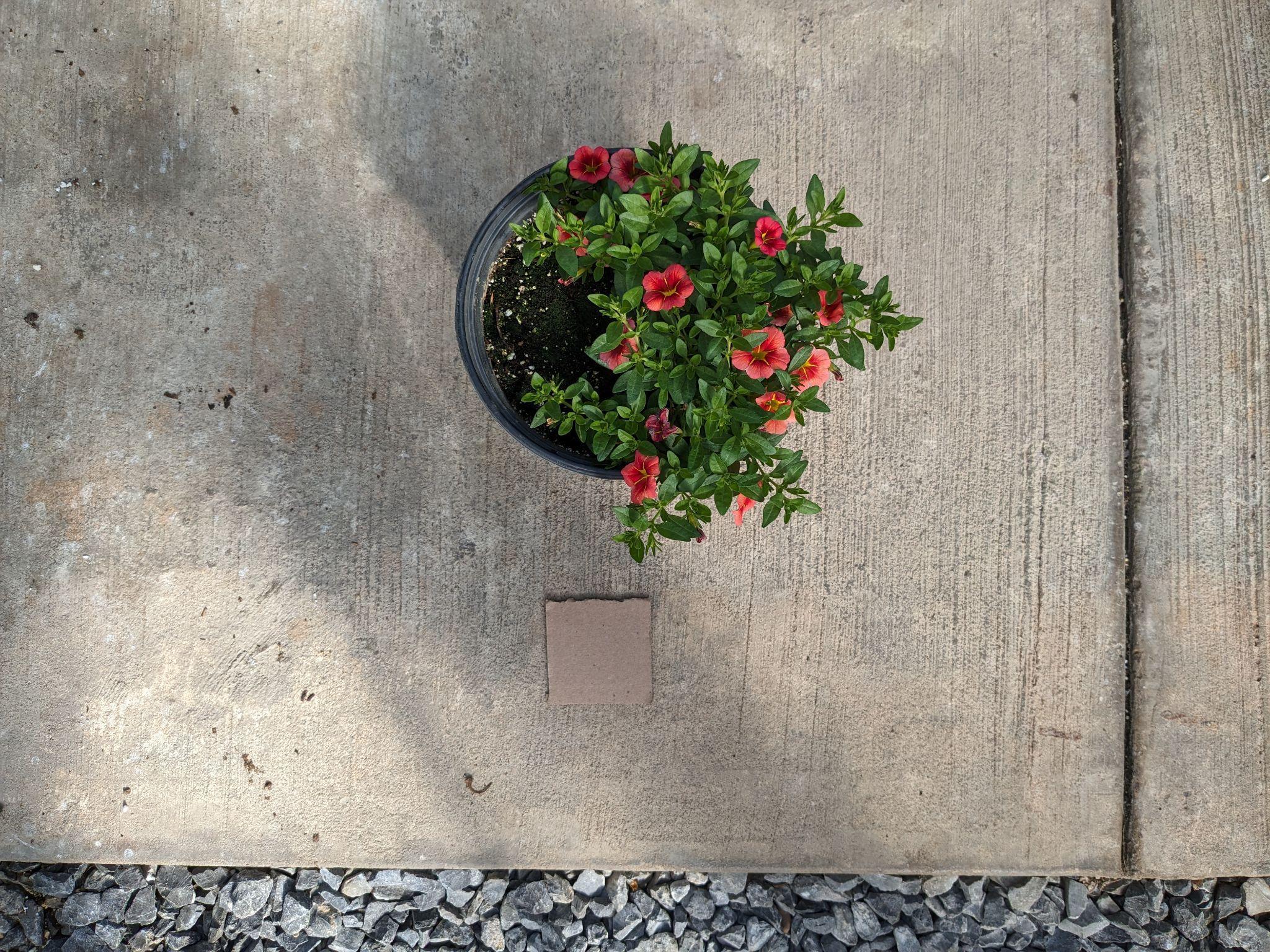 | 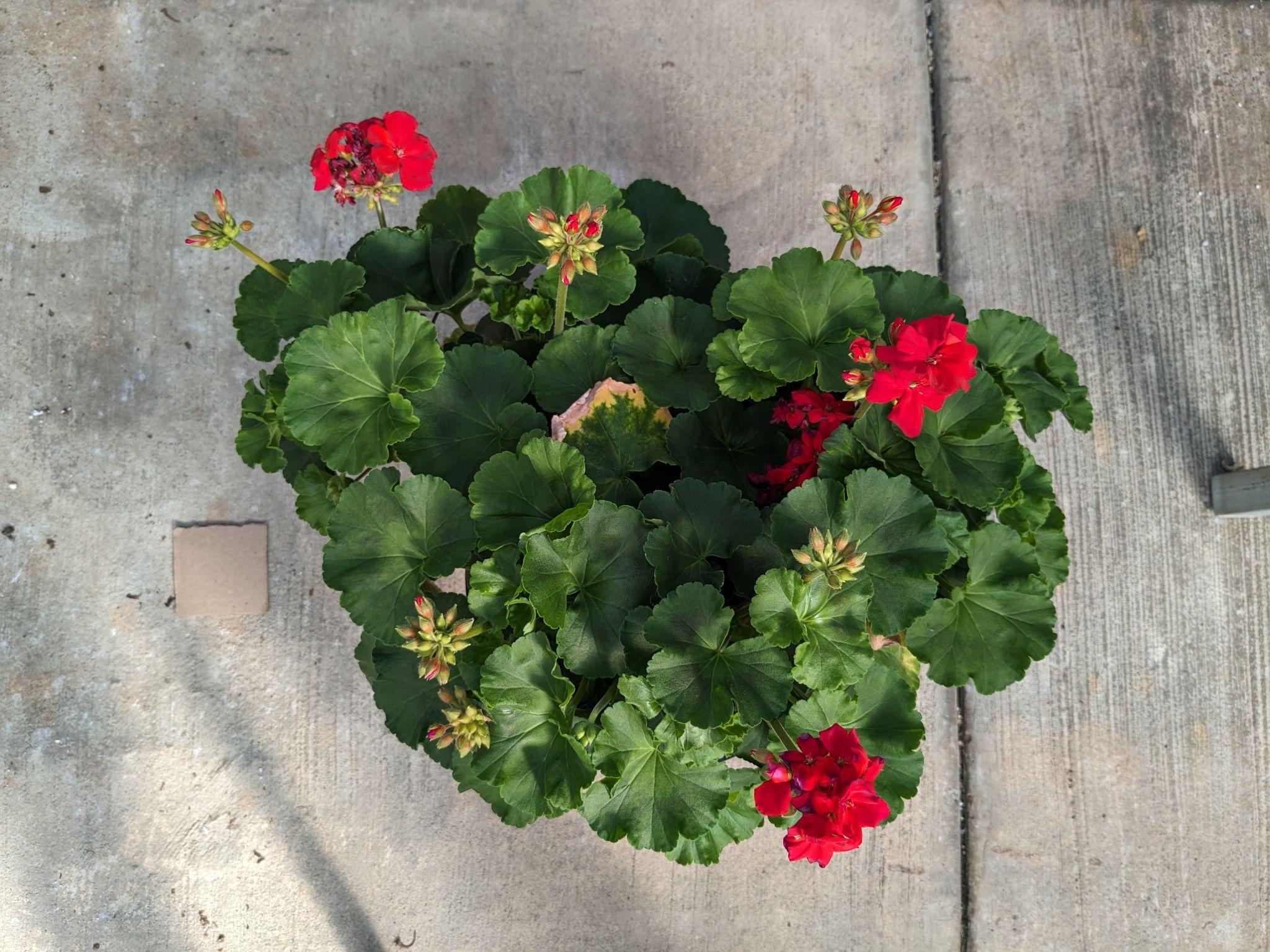 |  |
| 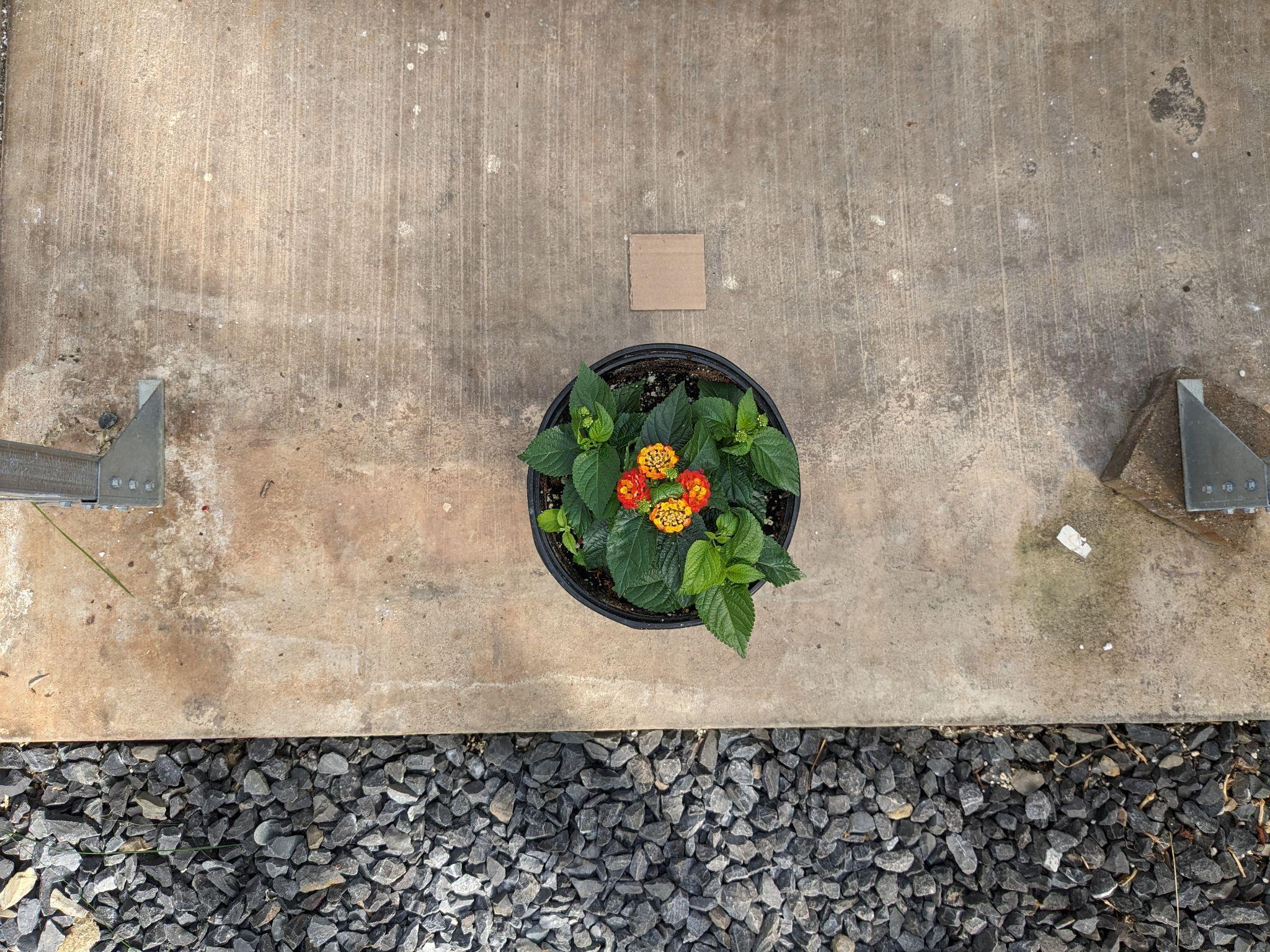 | 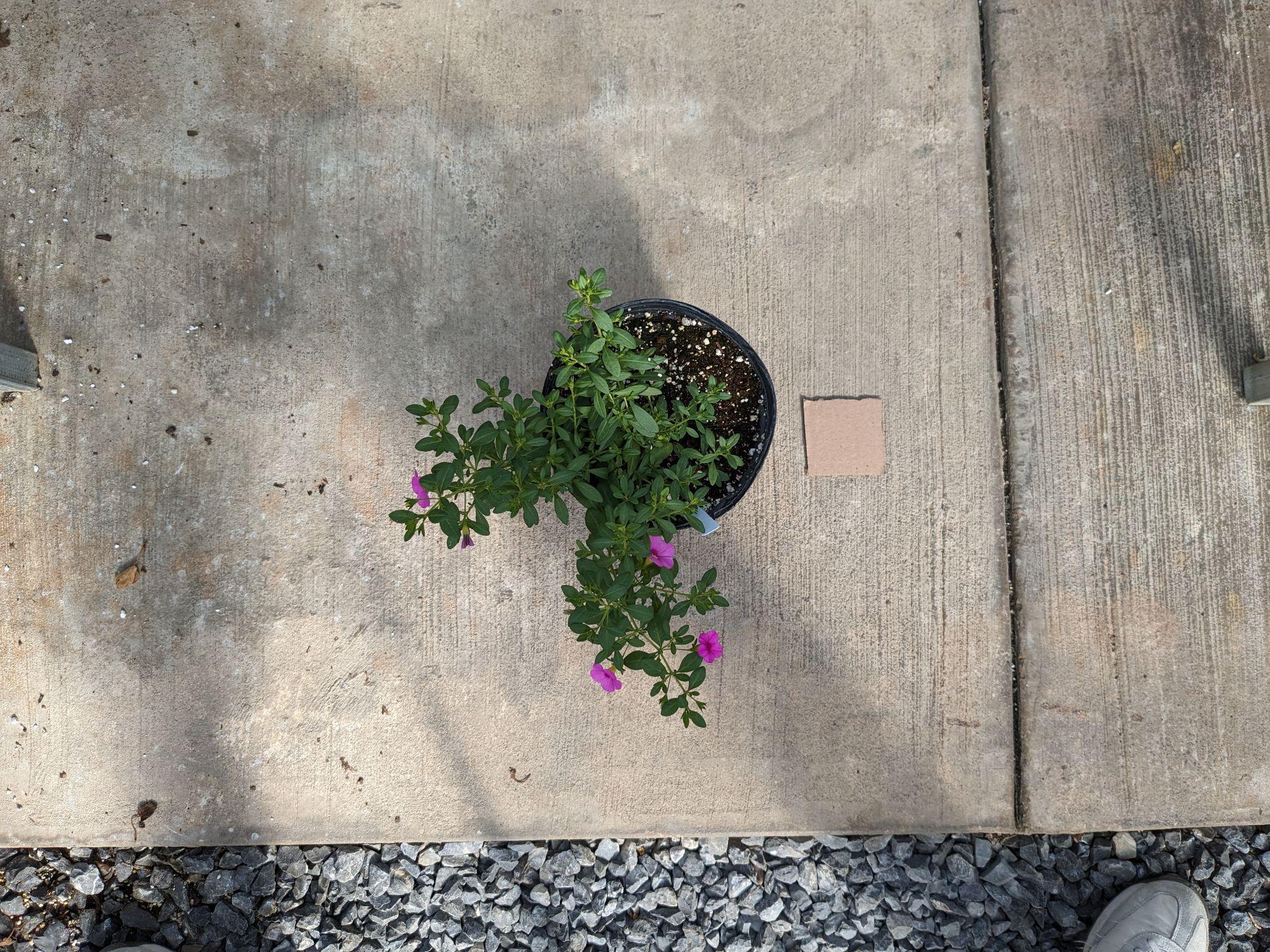 | 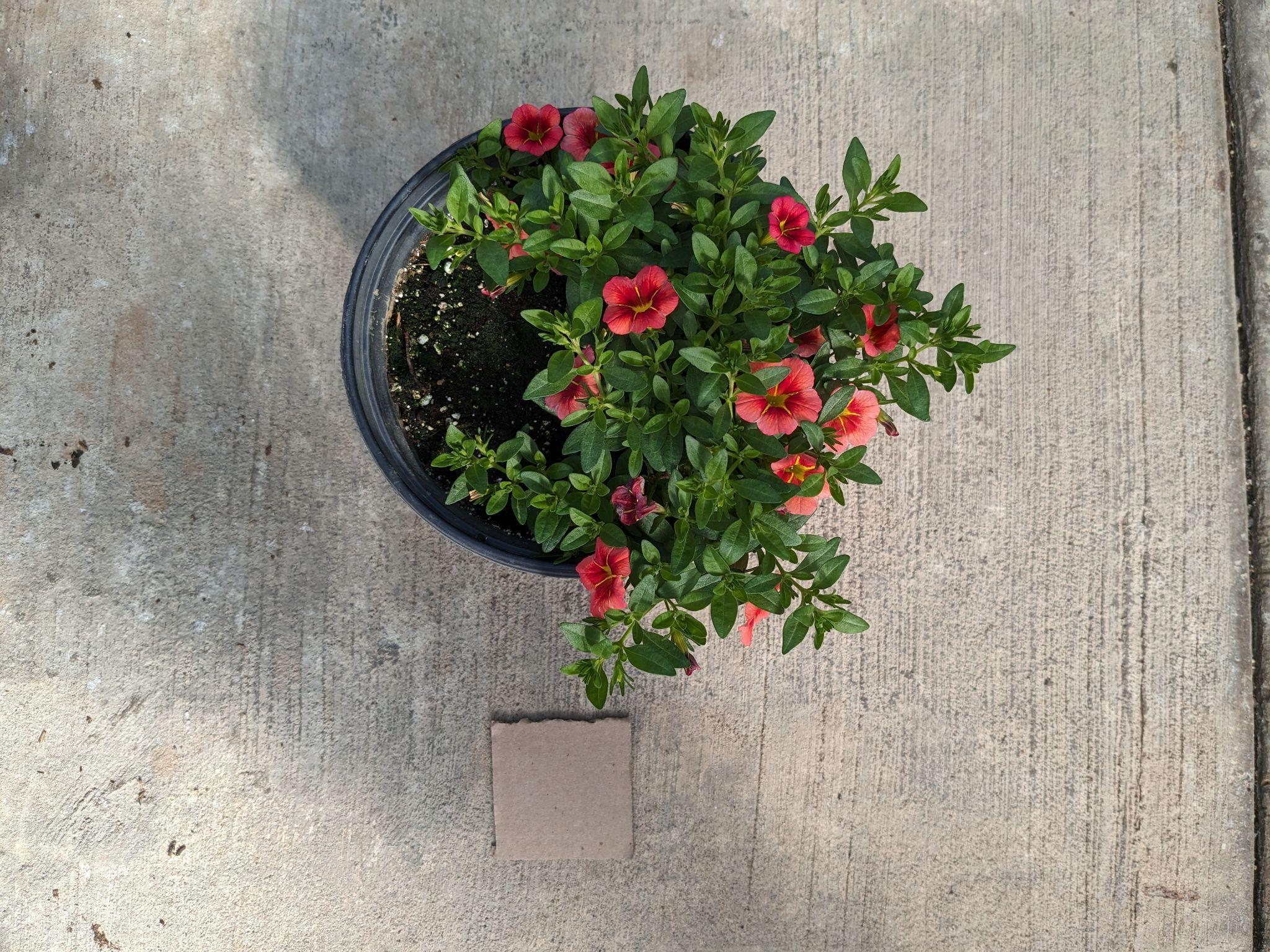 | 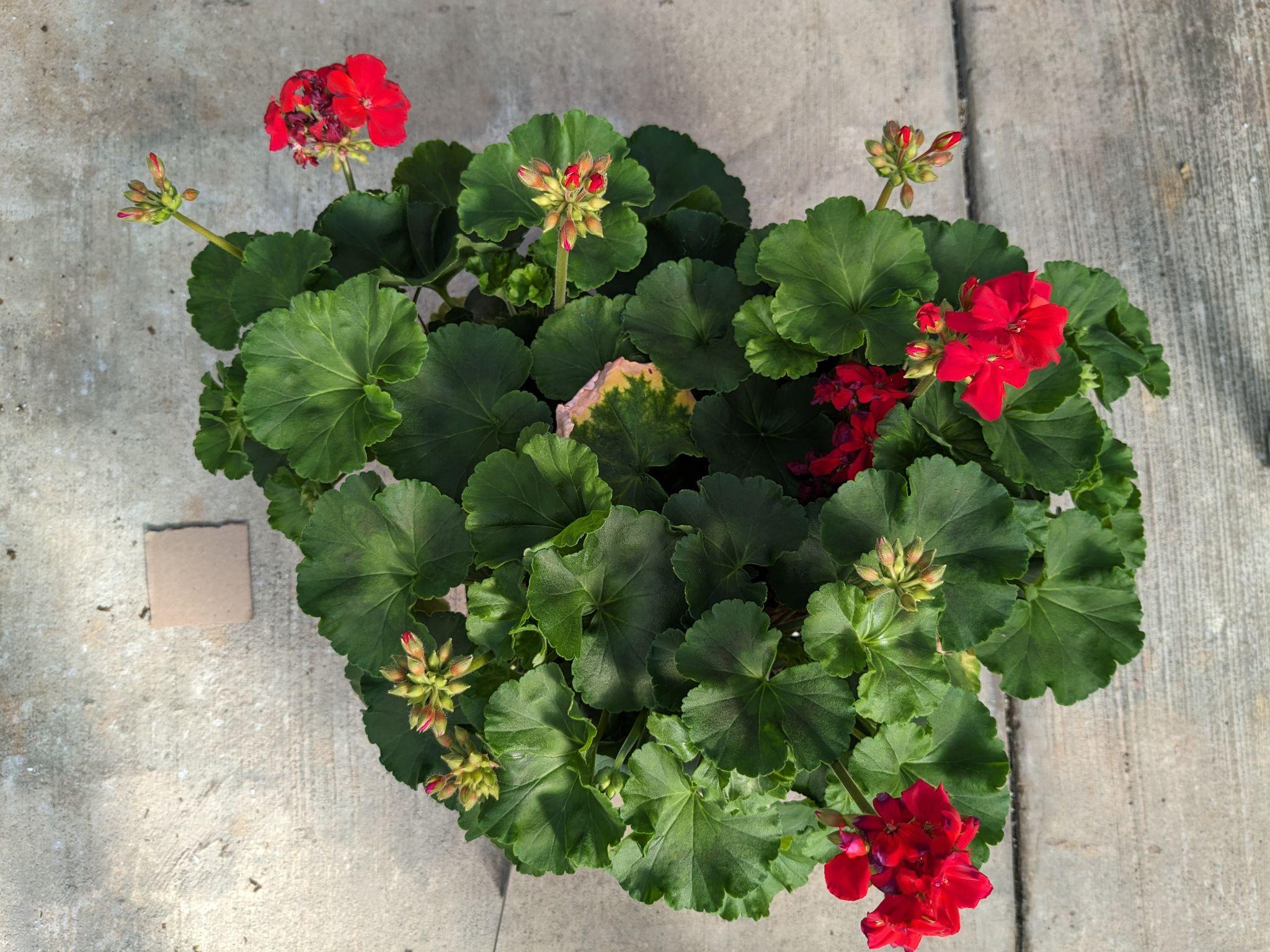 |  |
| 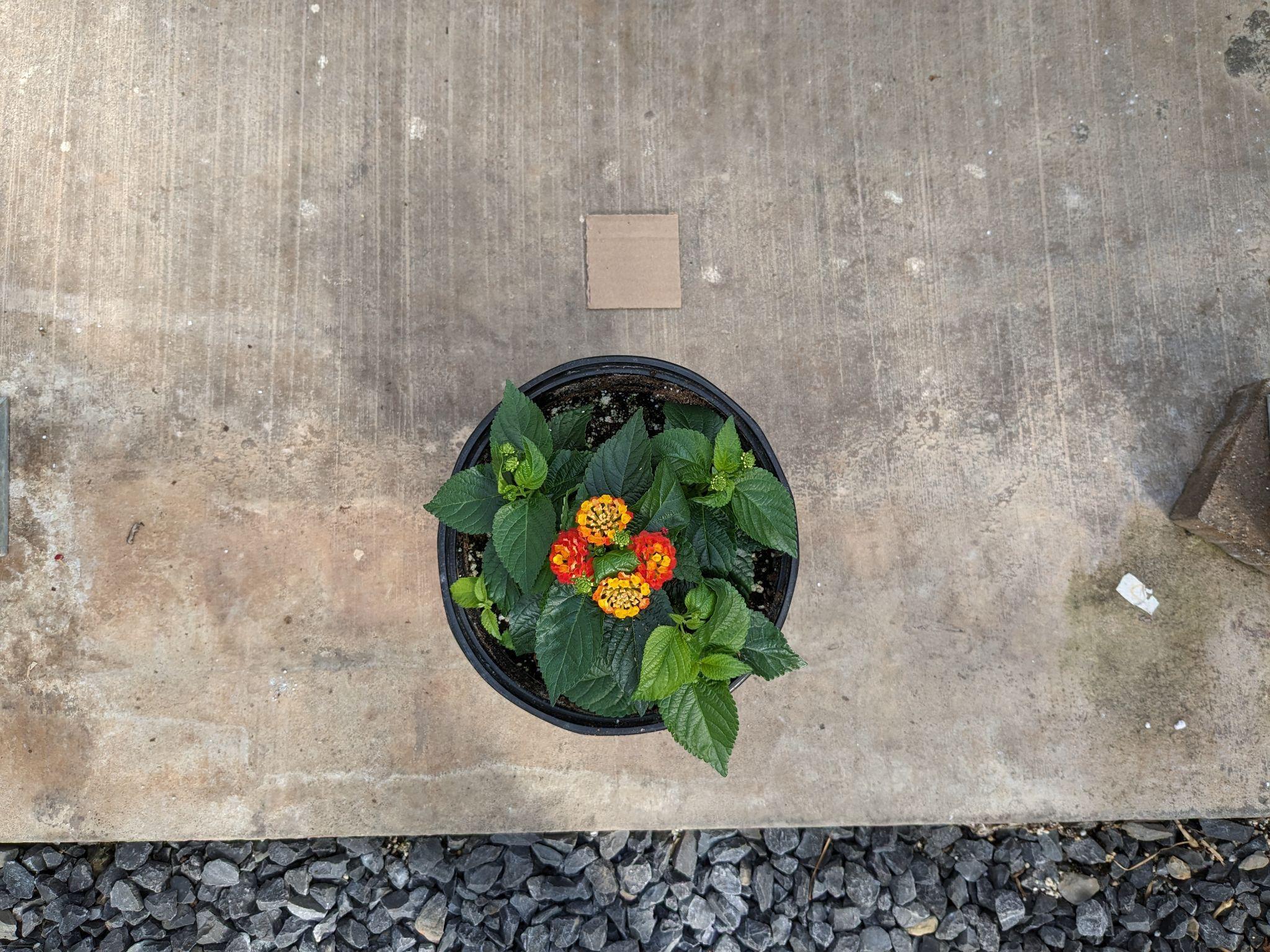 | 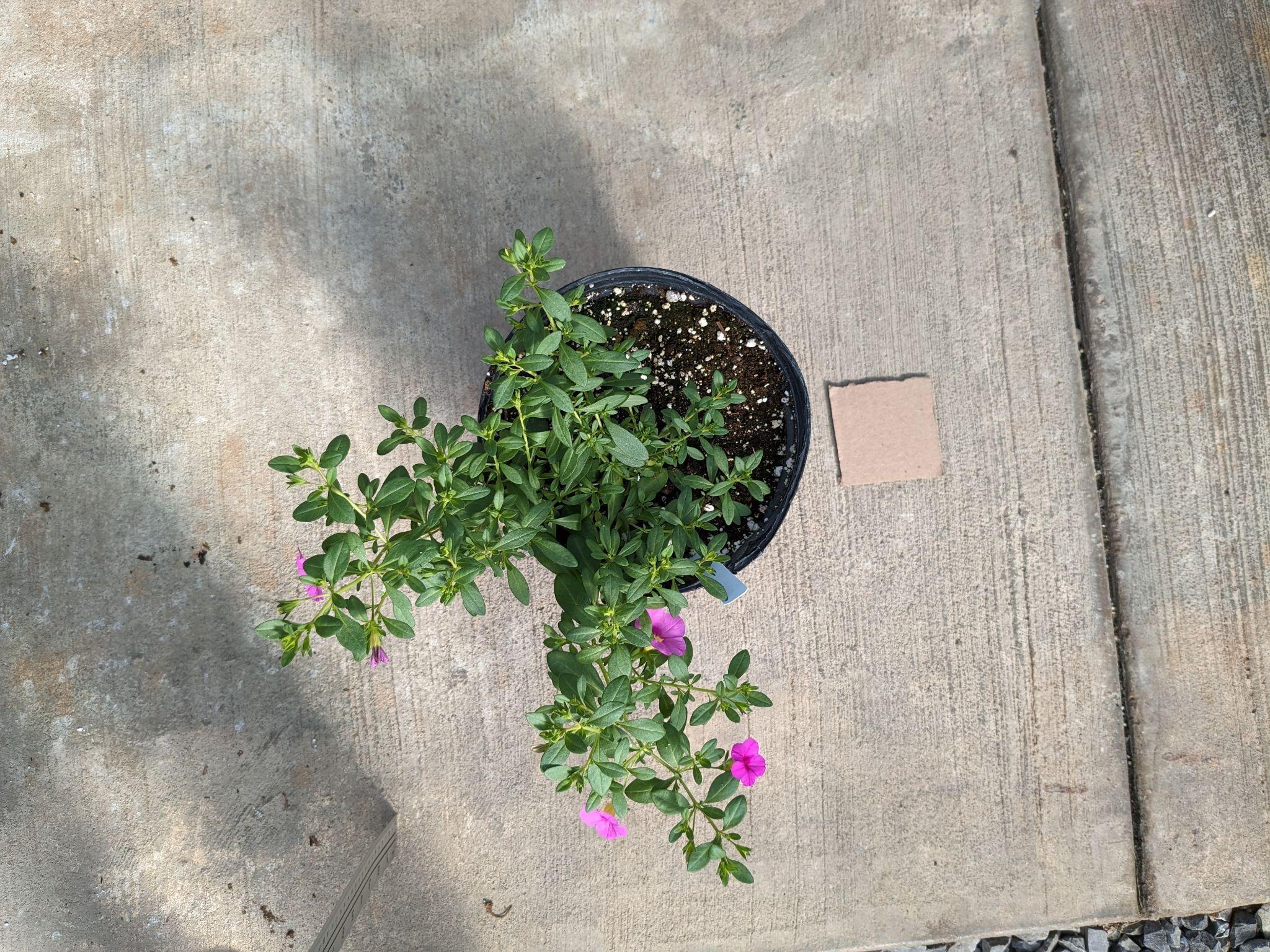 | 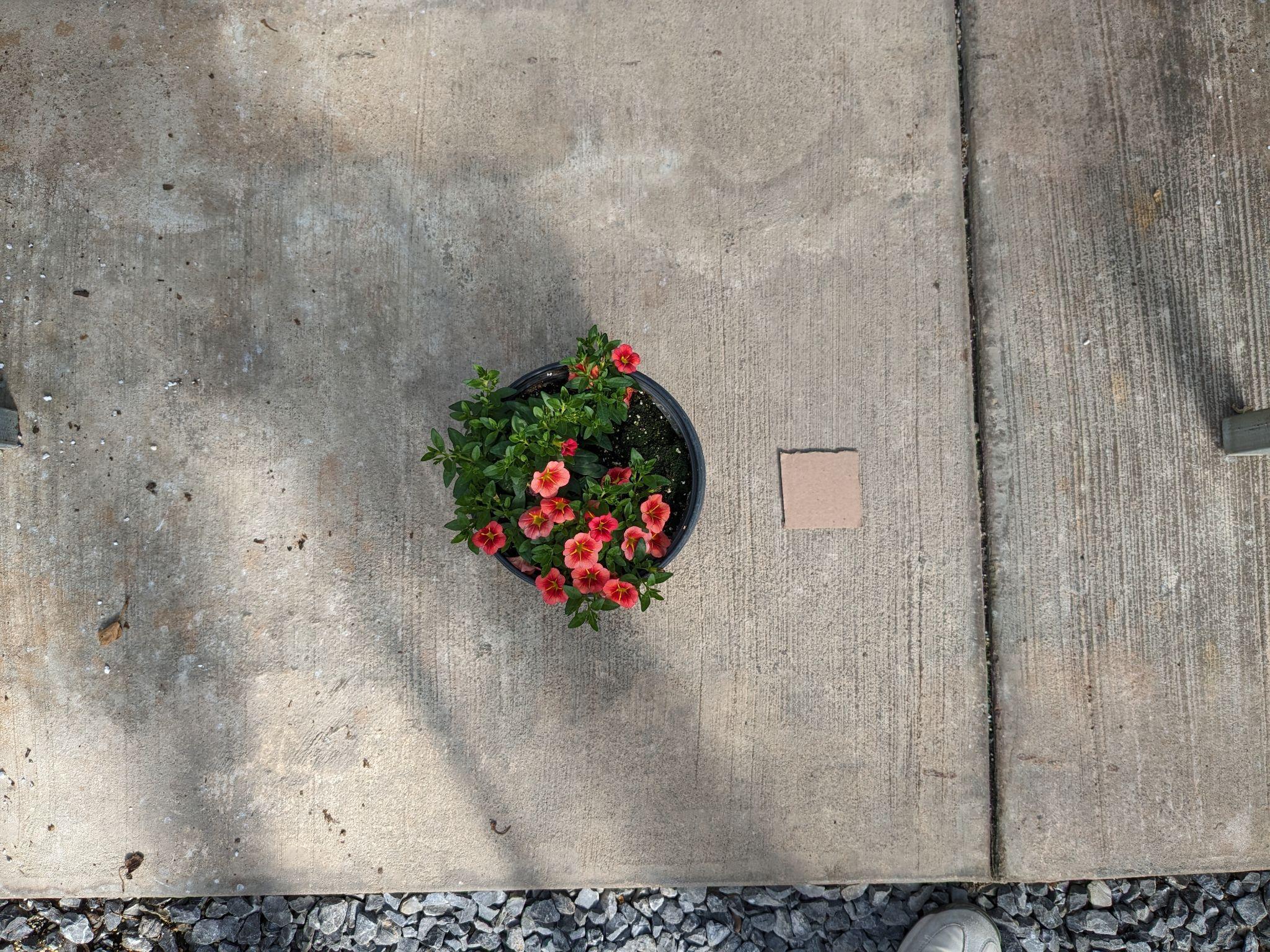 | 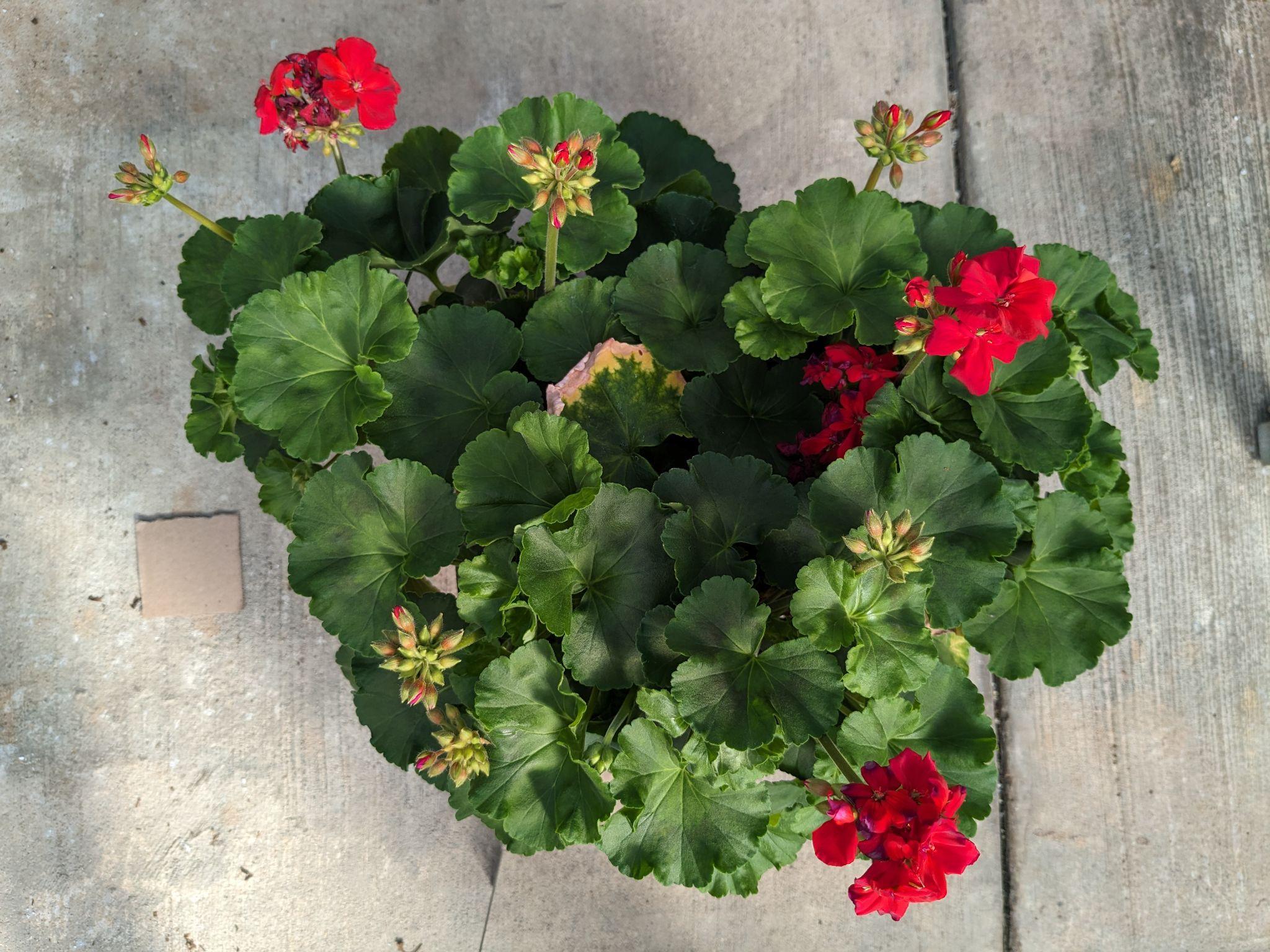 |  |
| 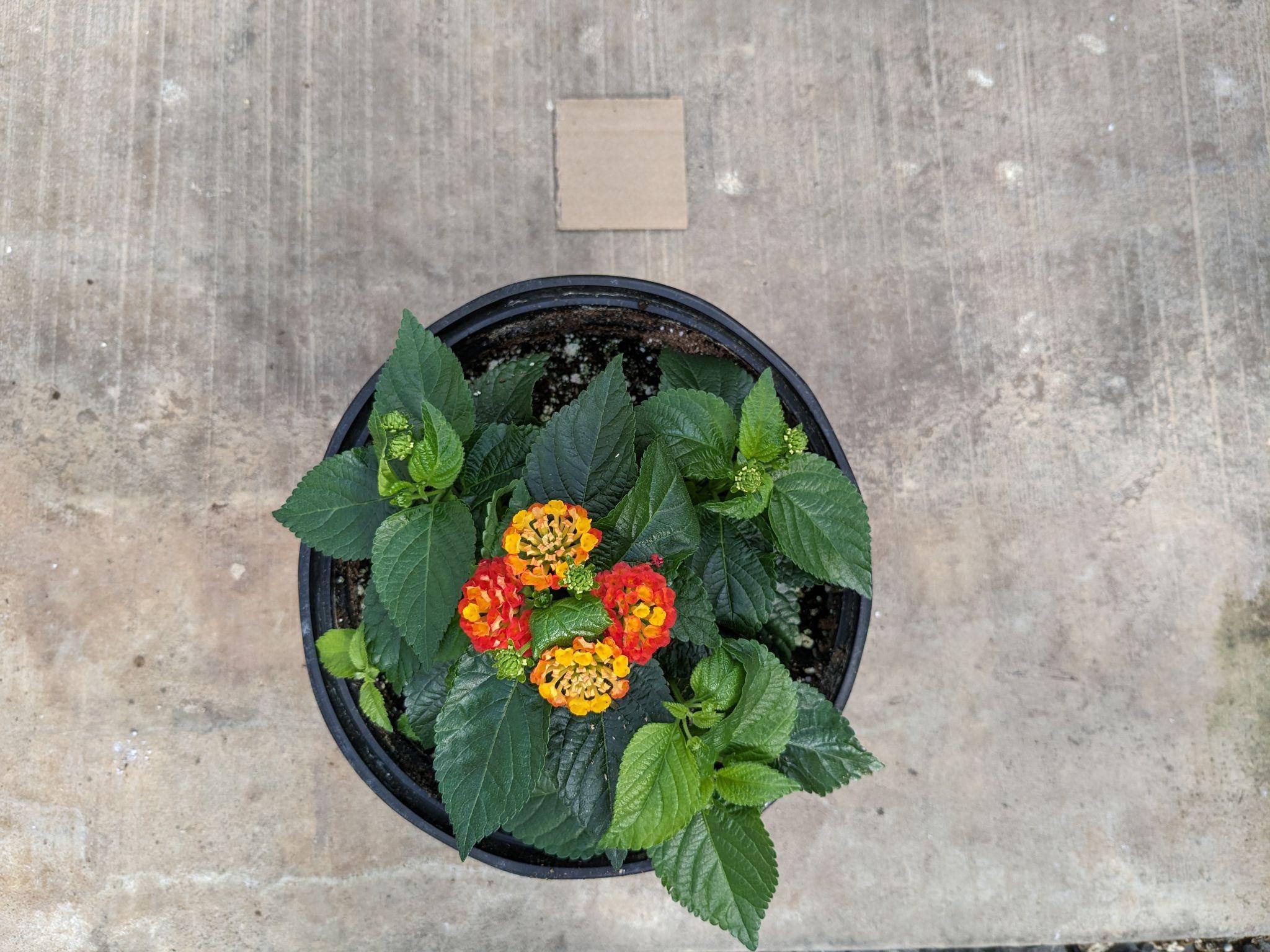 | 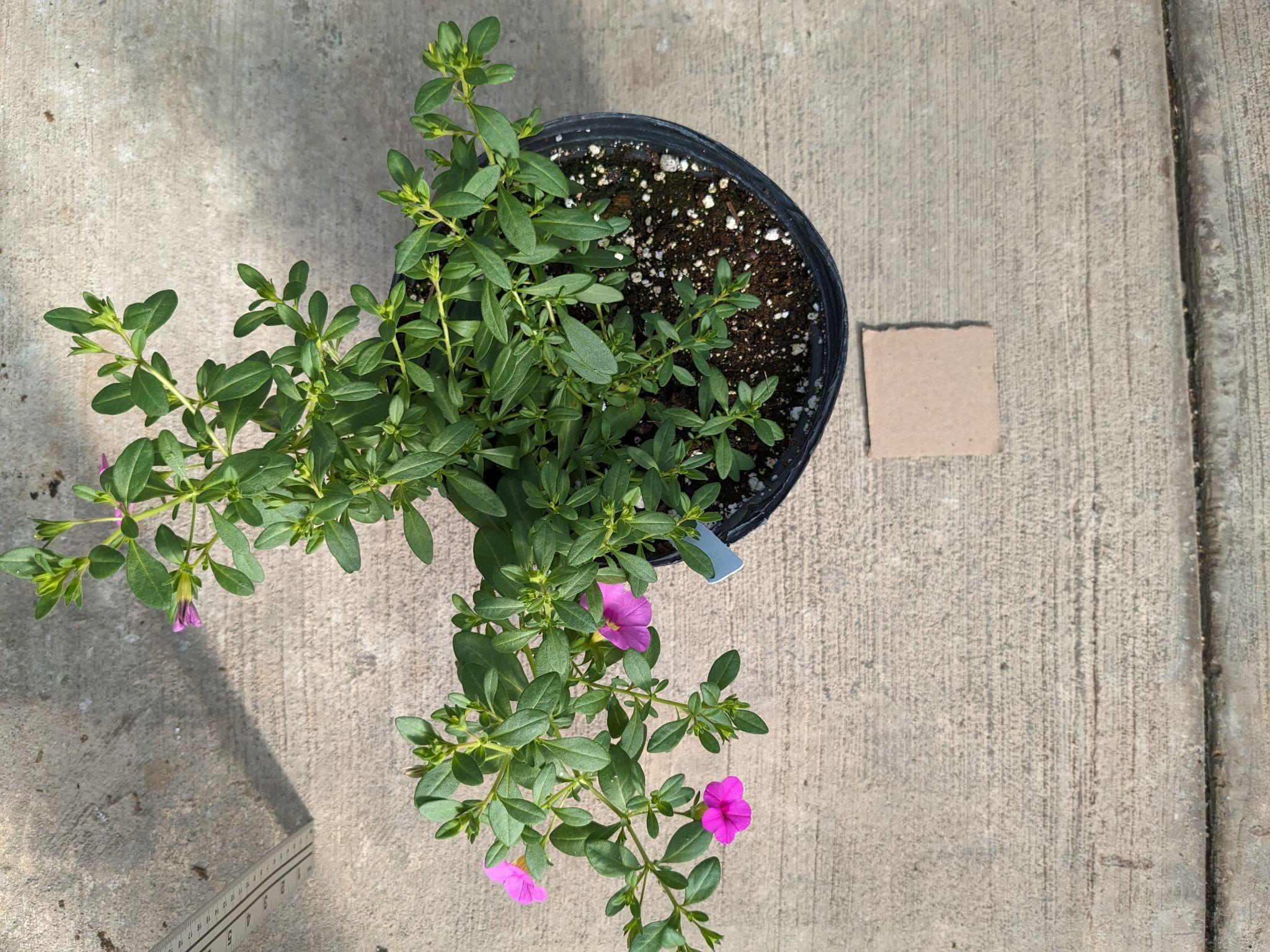 | 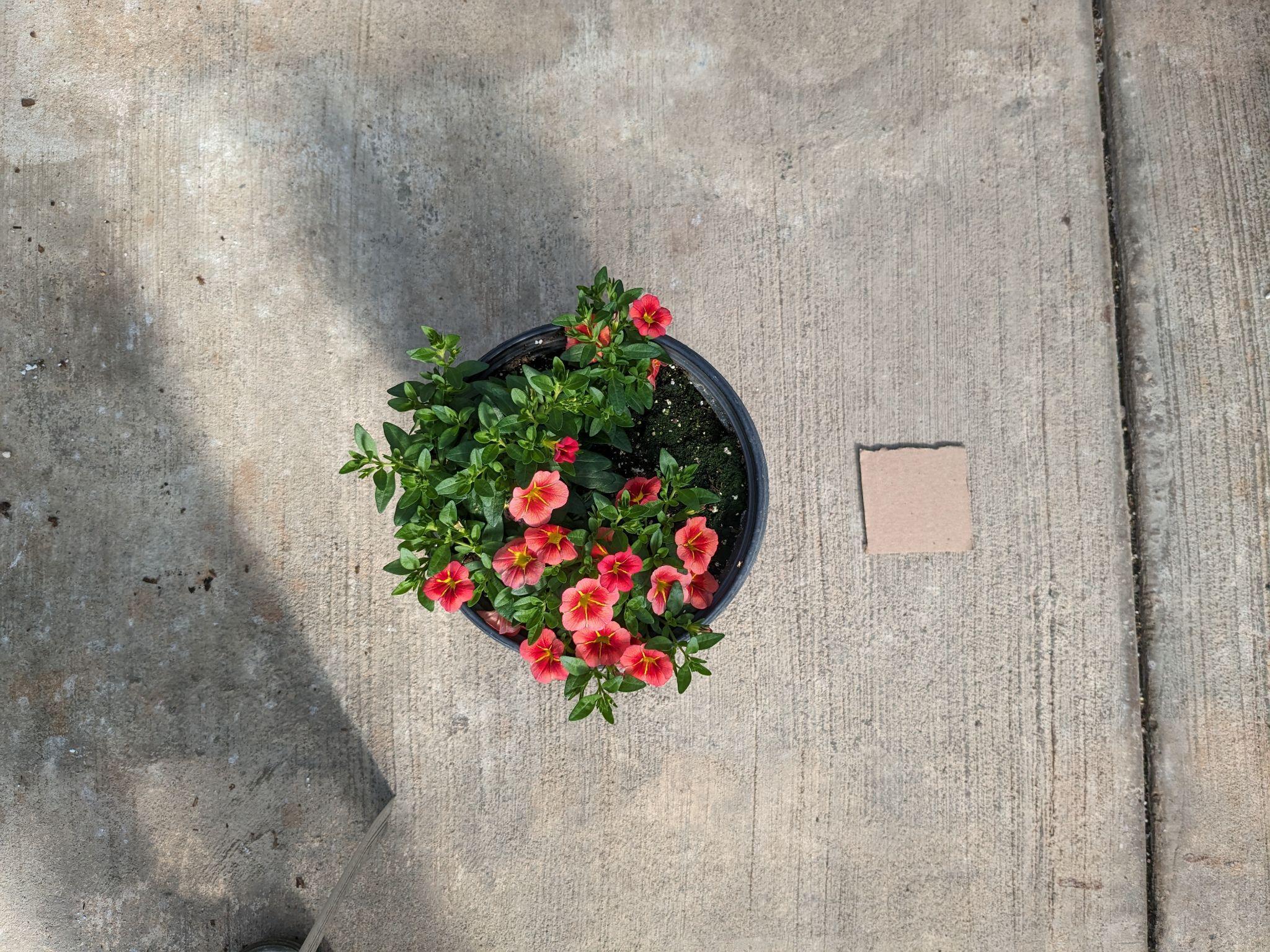 | 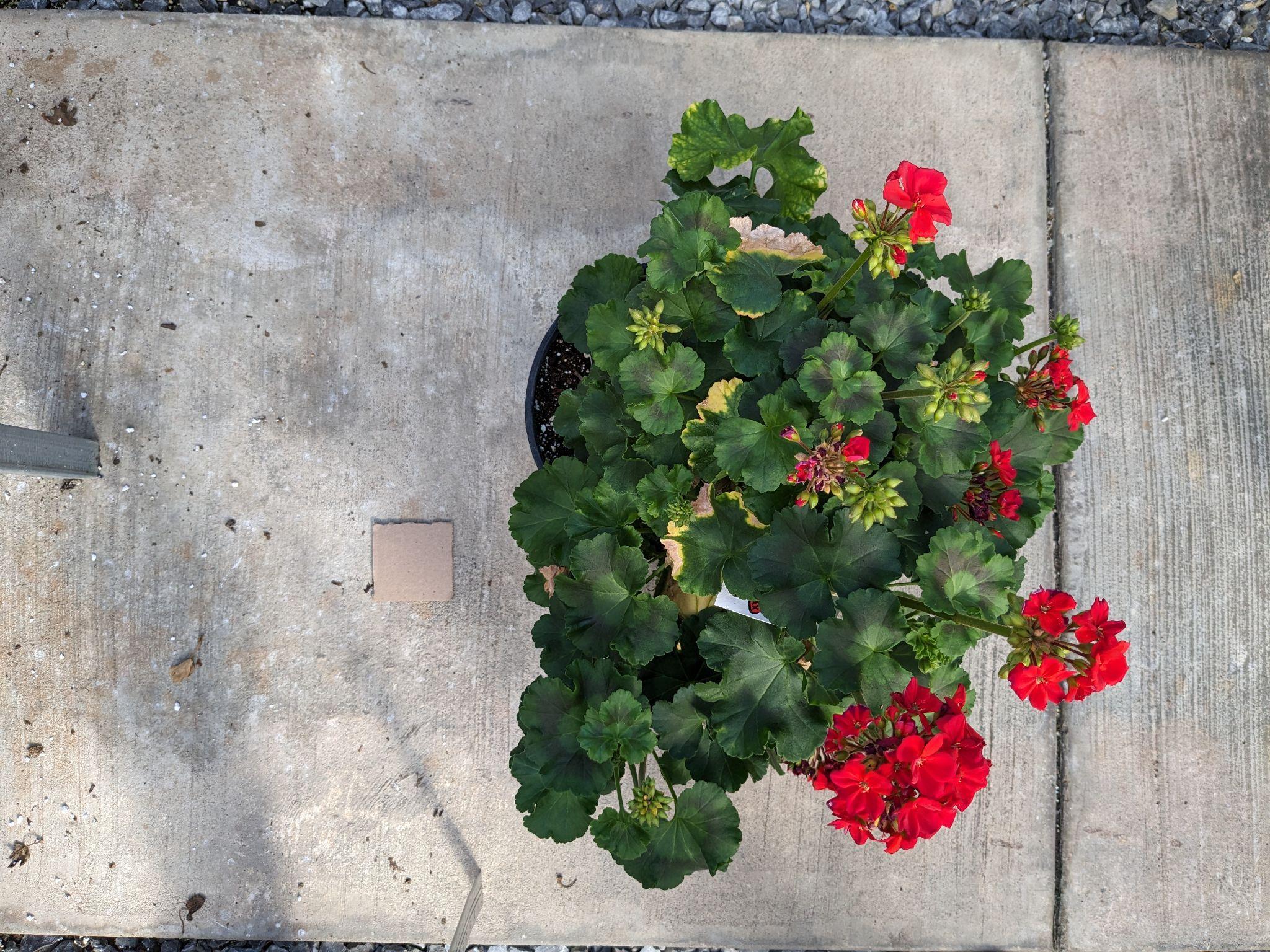 |  |
| 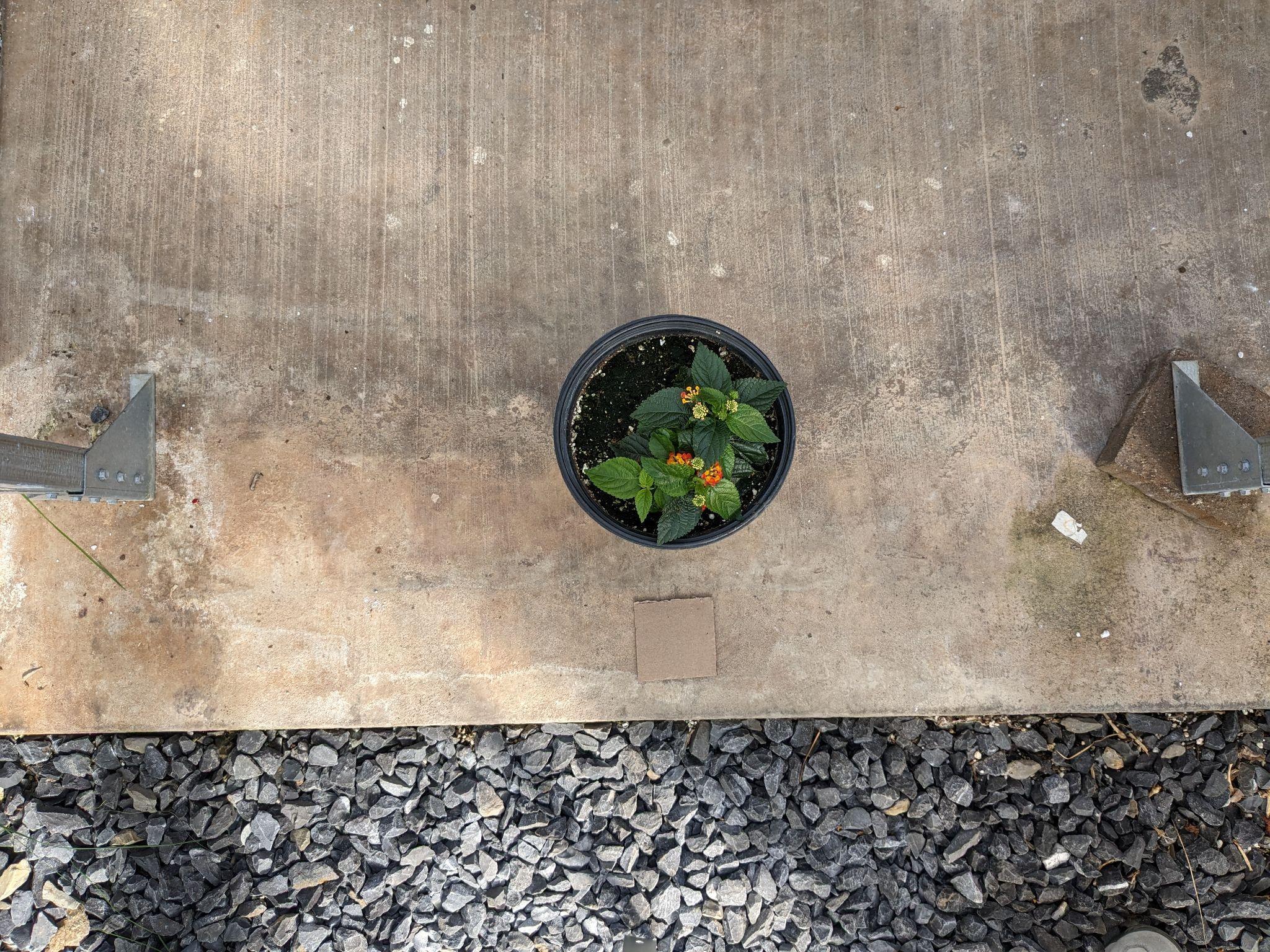 |  | 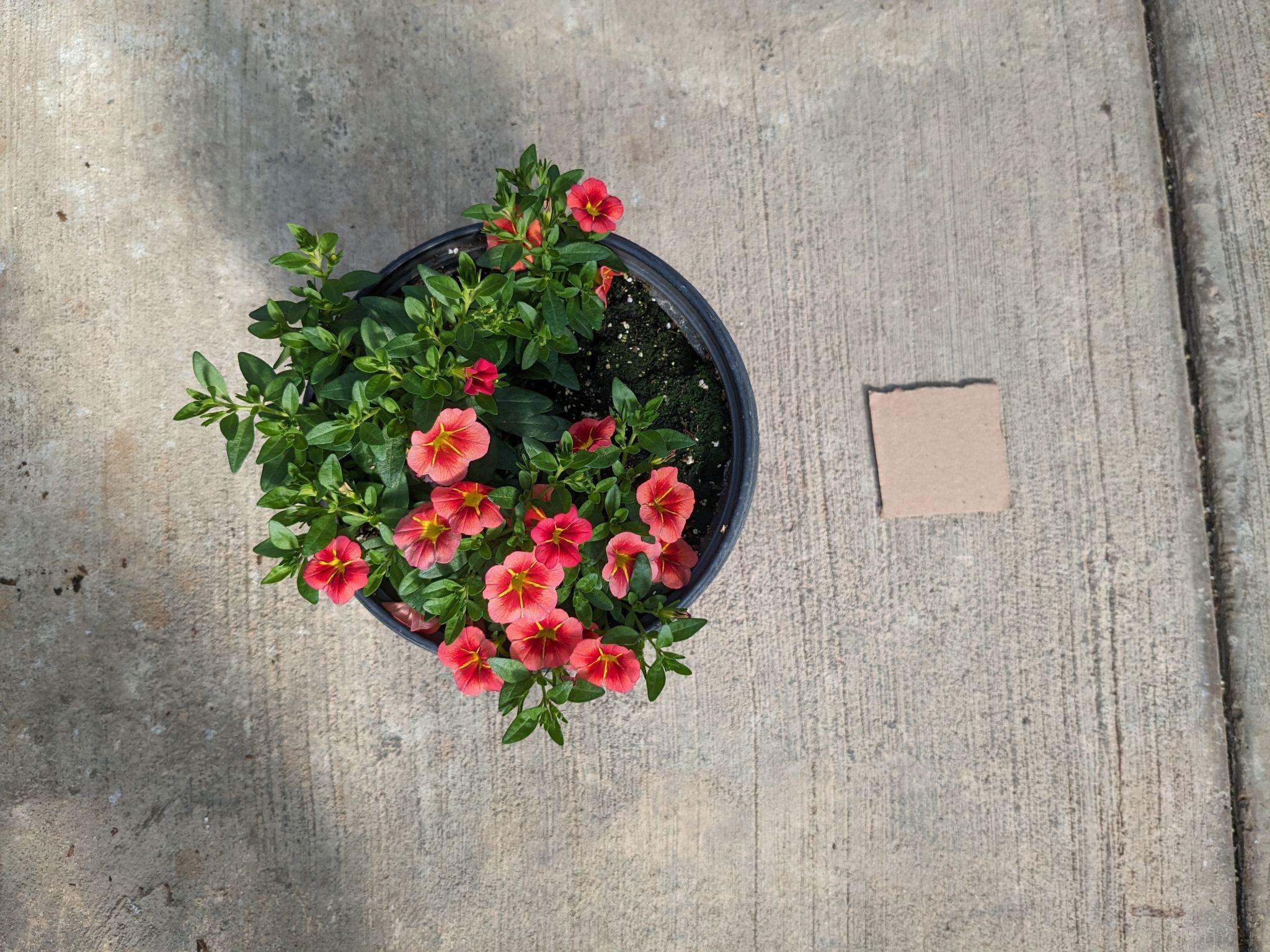 | 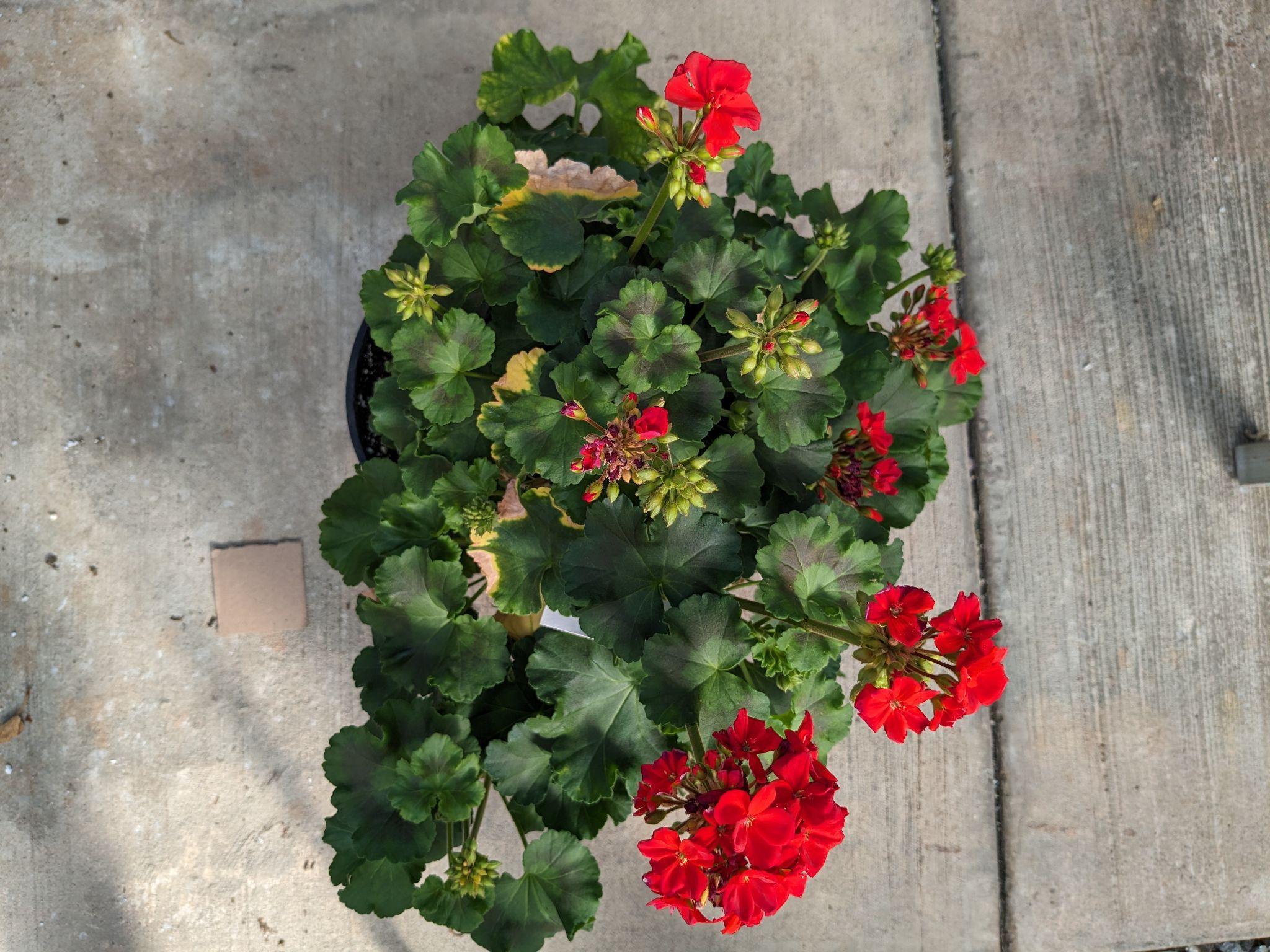 |  |
| 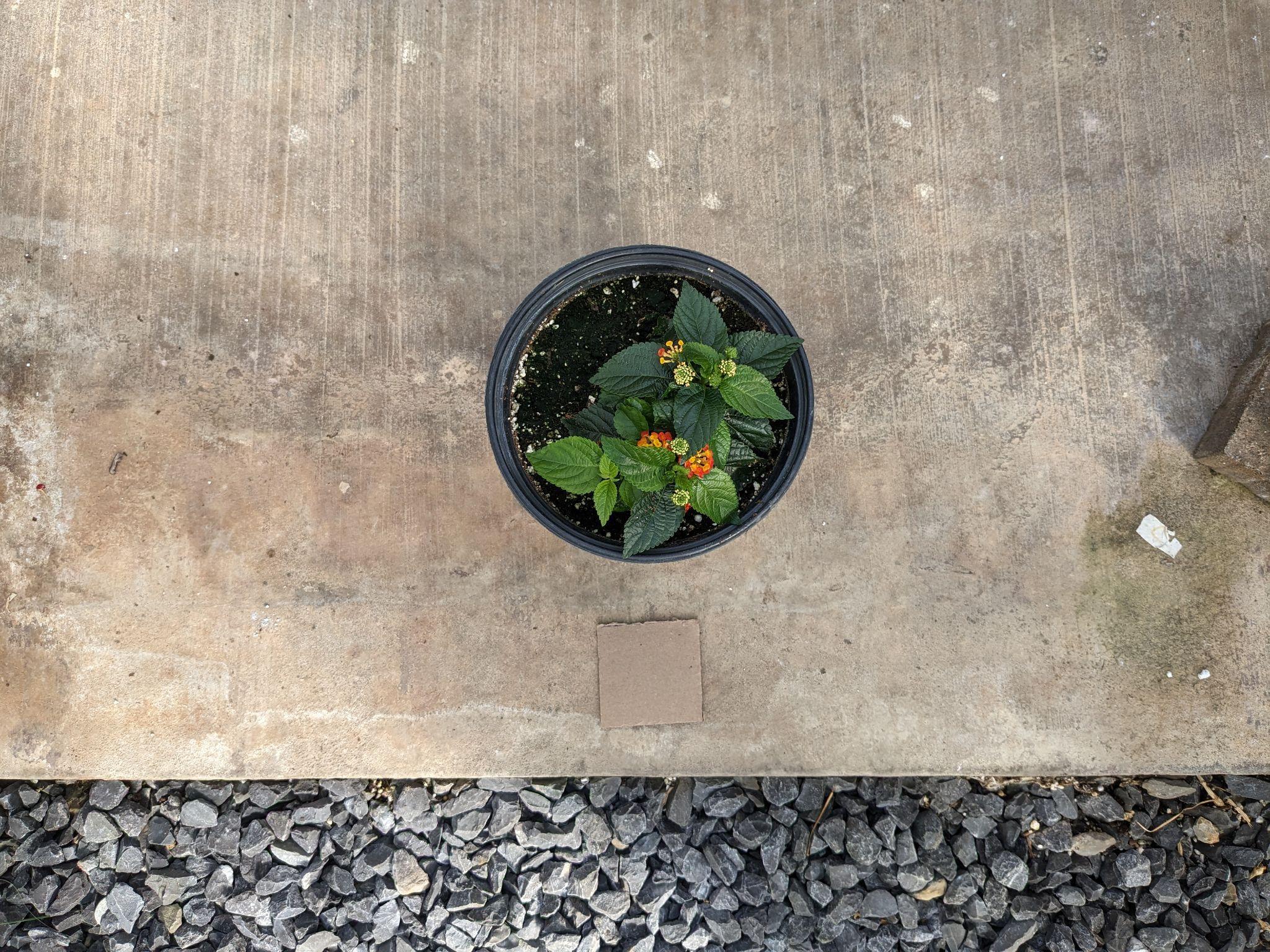 |  | 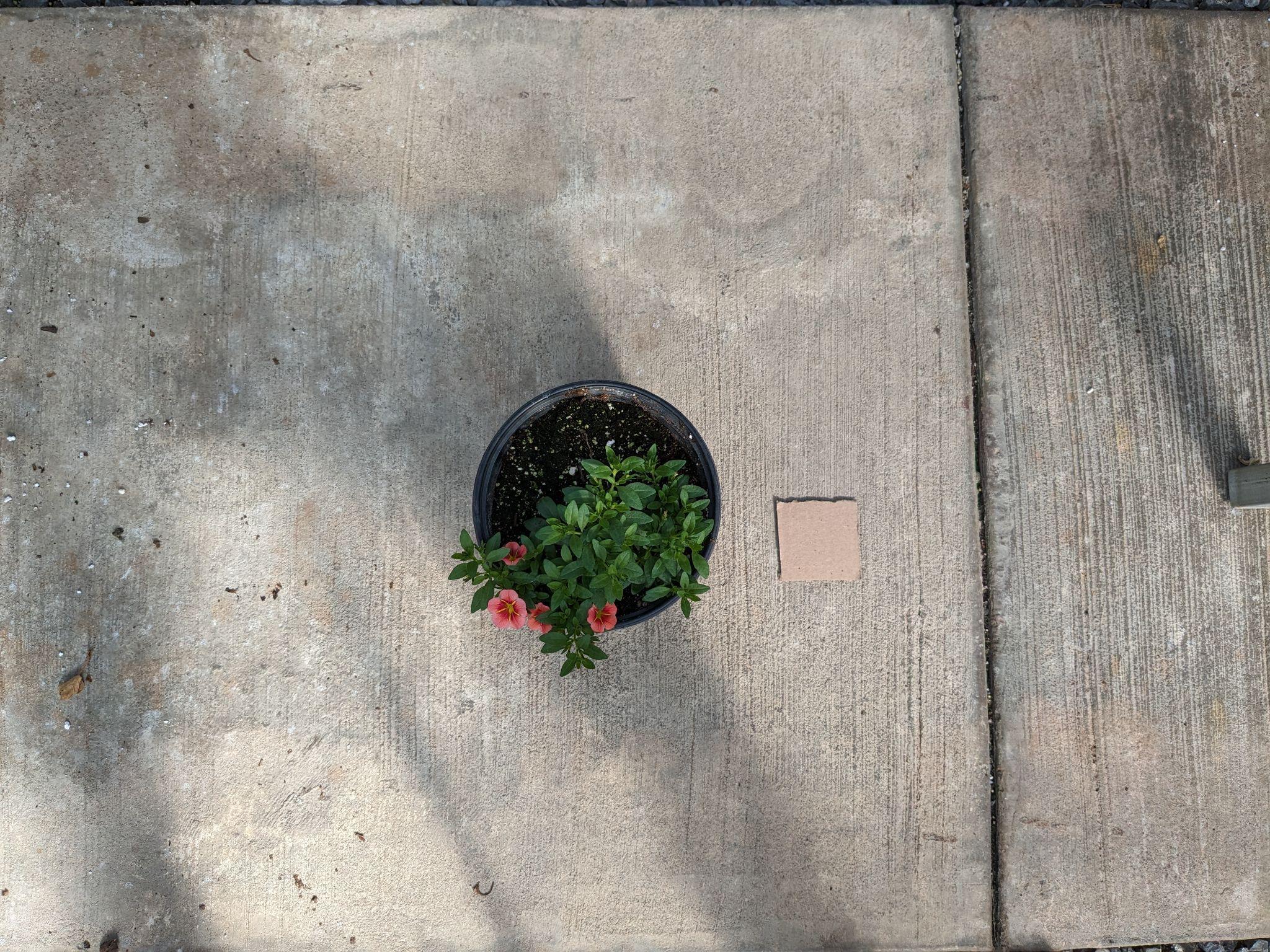 | 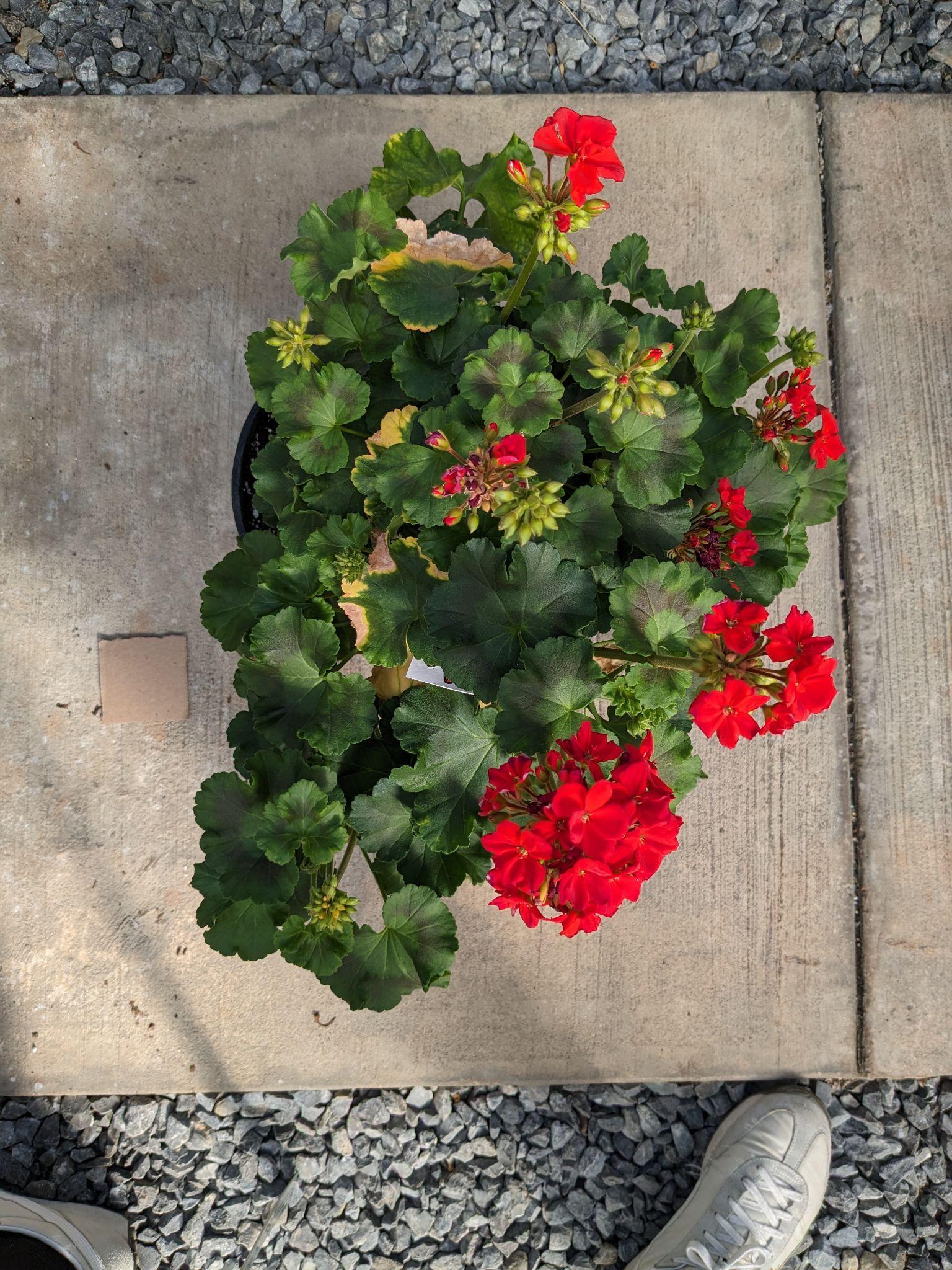 |  |
| 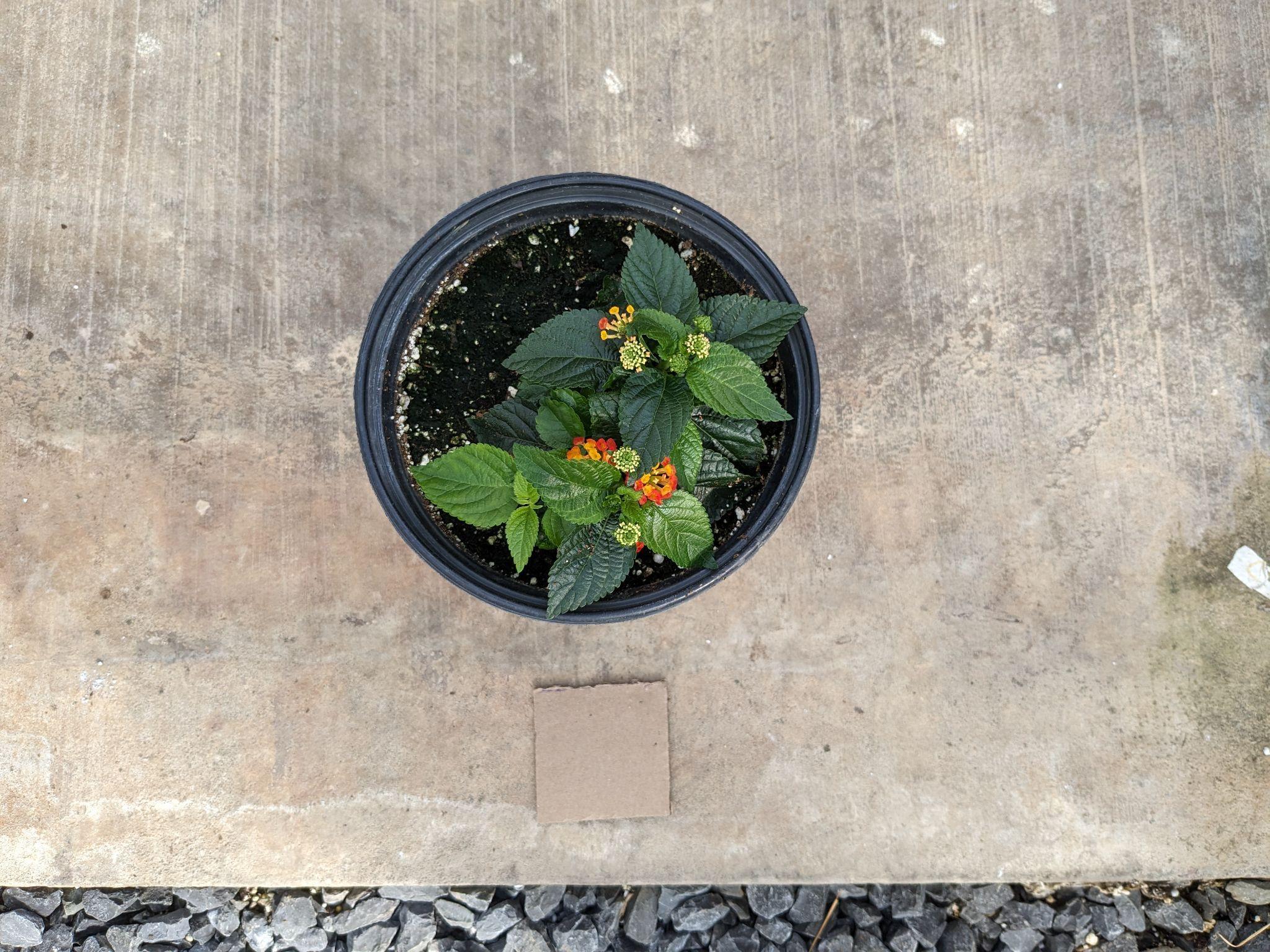 |  | 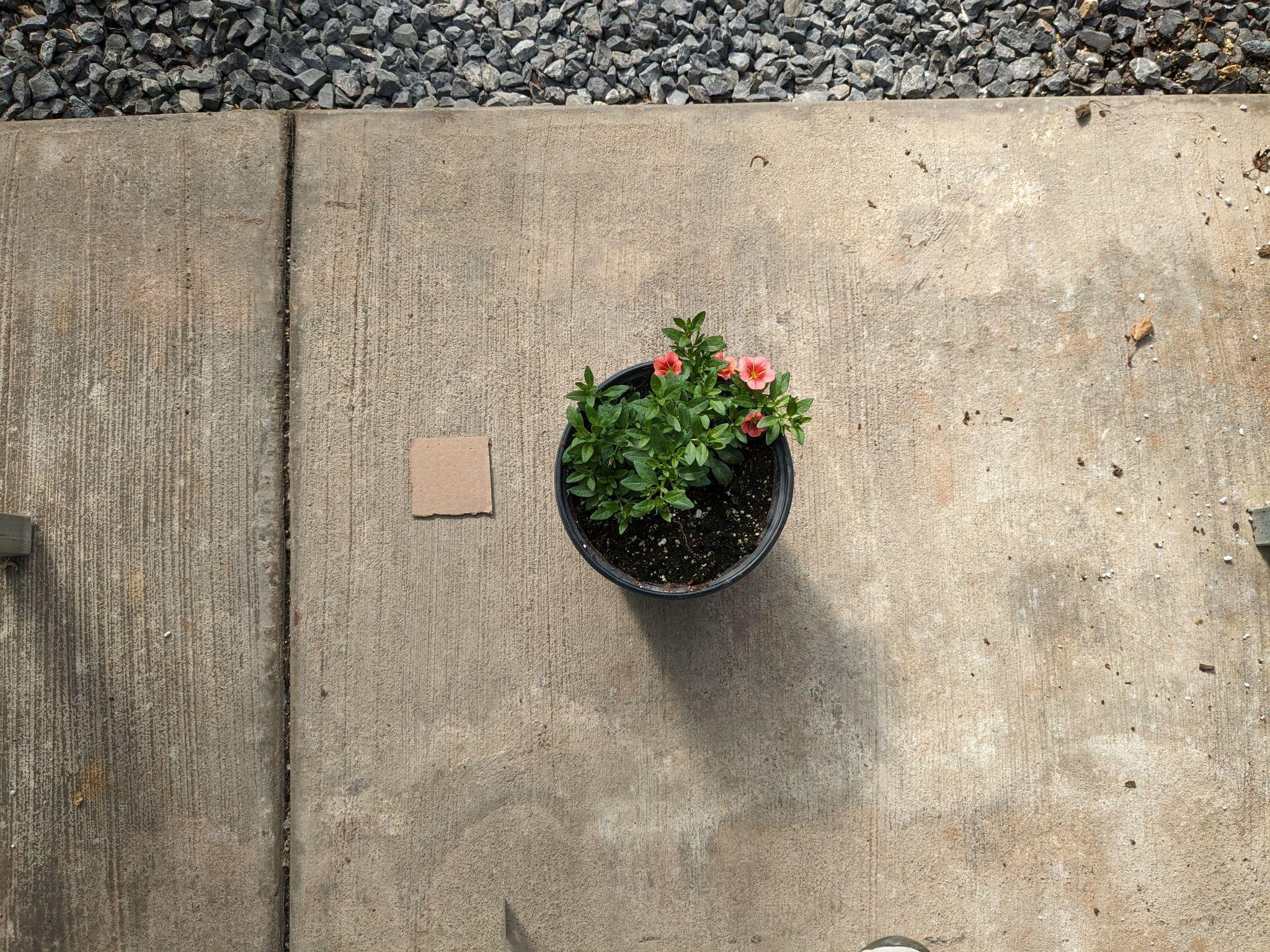 |  |  |
|  |  | 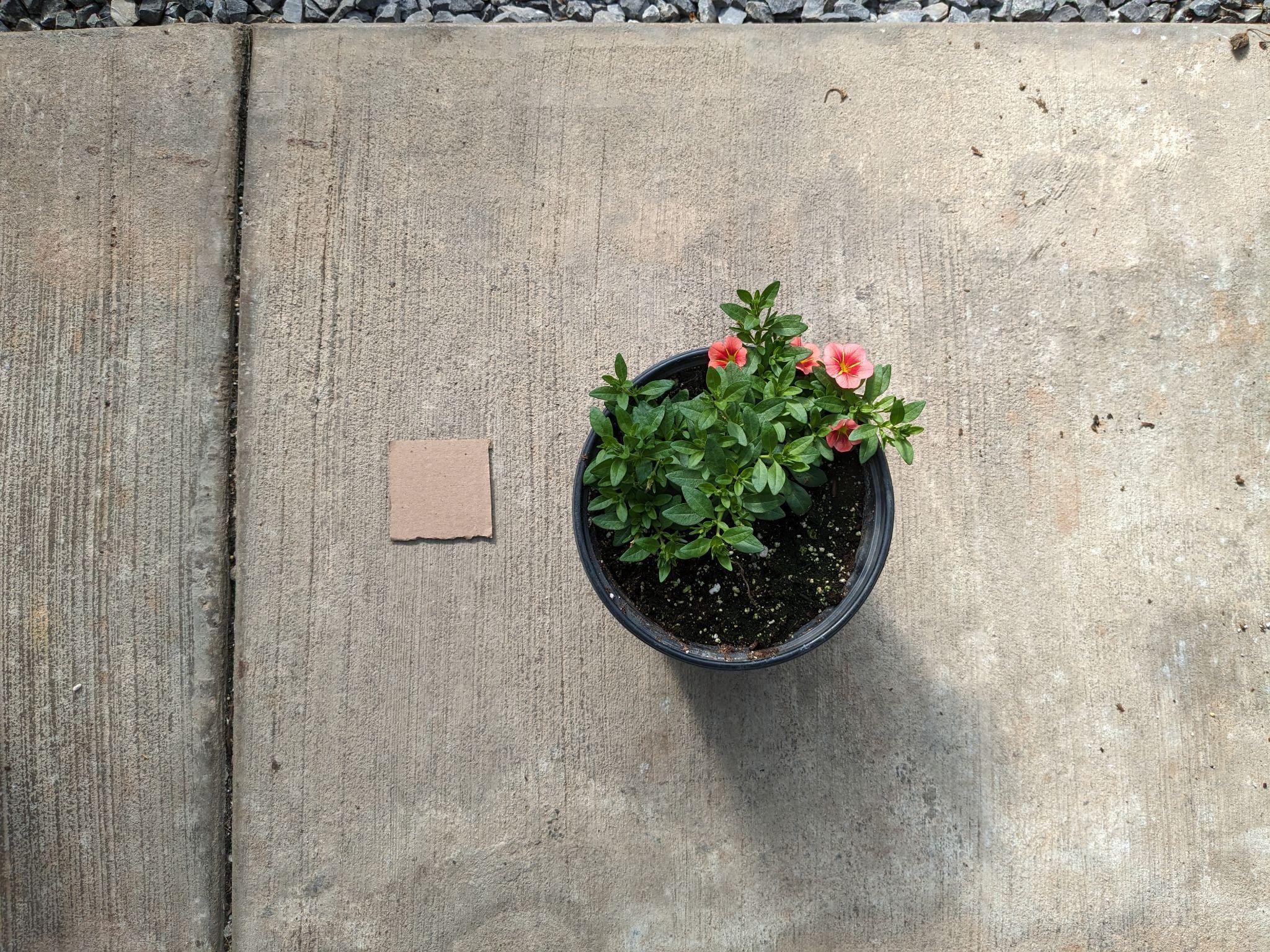 |  |  |
|  |  | 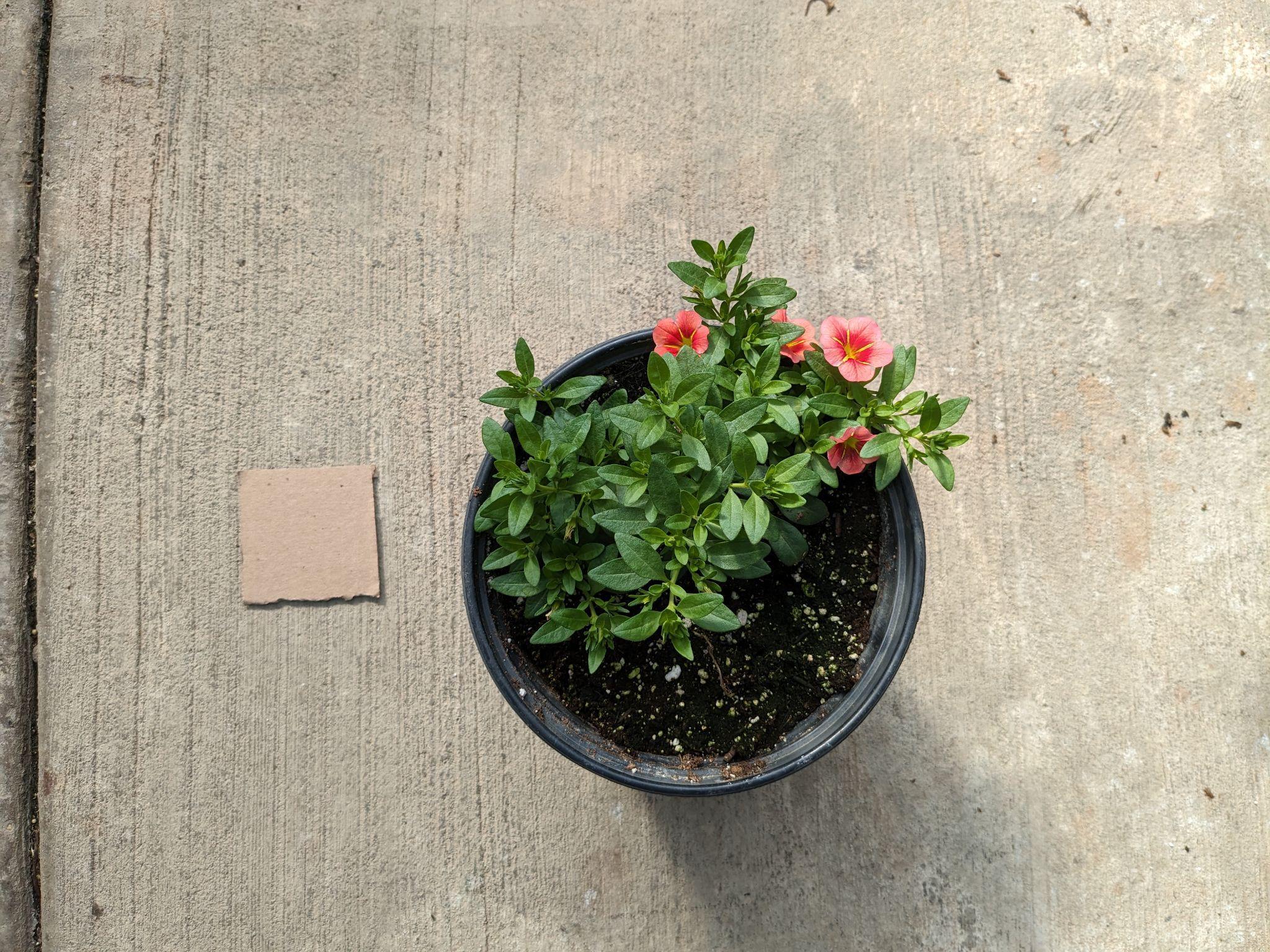 |  |  |
|  |  | 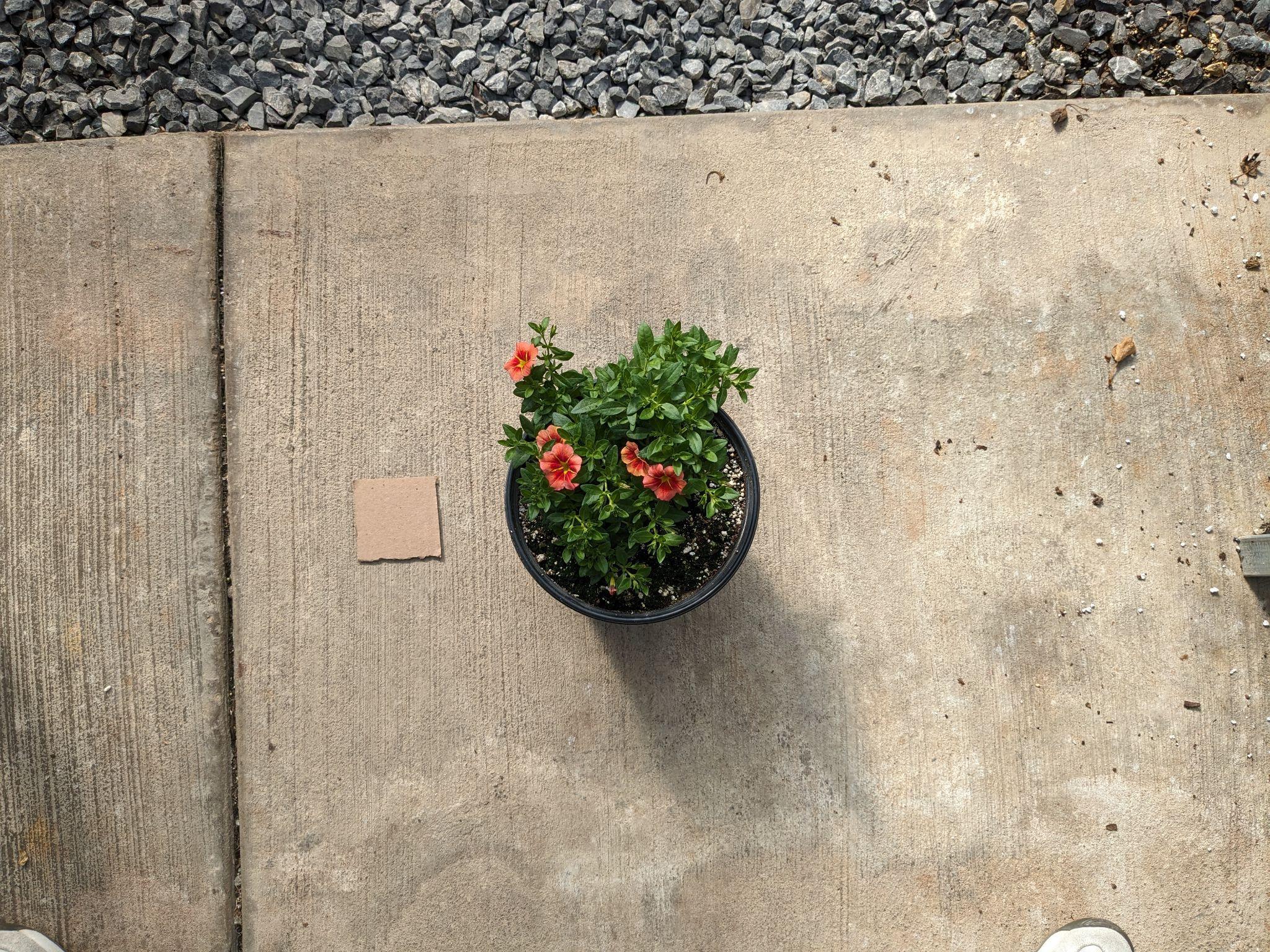 |  |  |

Supplement: S1 Table — Twenty-three flowering plants were used to evaluate the efficiency and accuracy of the FloralArea algorithm, and images were taken of these plants at multiple heights, resulting in 75 total images. These flowering plants were sorted into five categories (T1-5) to investigate the influence of color on the algorithm’s performance. (DOCX) [file pone.0332165.s004.docx]
