# Supplementary material for: FloralArea: AI-powered algorithm for automated calculation of floral area from flower images to support plant and pollinator research
Source: PLoS One. 2025 Sep 12;20(9):e0332165. doi: 10.1371/journal.pone.0332165 (PMC12431086; doi:10.1371/journal.pone.0332165)
Supplement: S2 Table — The flowering plants were organized into three categories (C1-3) according to flower traits to investigate the influence of flower traits on the algorithm’s performance. (DOCX) [file pone.0332165.s005.docx]

**Supplementary Table** **S2. Flower Petal Display Category Chart.**

| **C1- Compound Inflorescences** | **C2 - Continuous Petals** | **C2- Continuous Petals** | **C3 - Clumps of Loose Petals** | **C-3Clumps of Loose Petals** |
| --- | --- | --- | --- | --- |
| 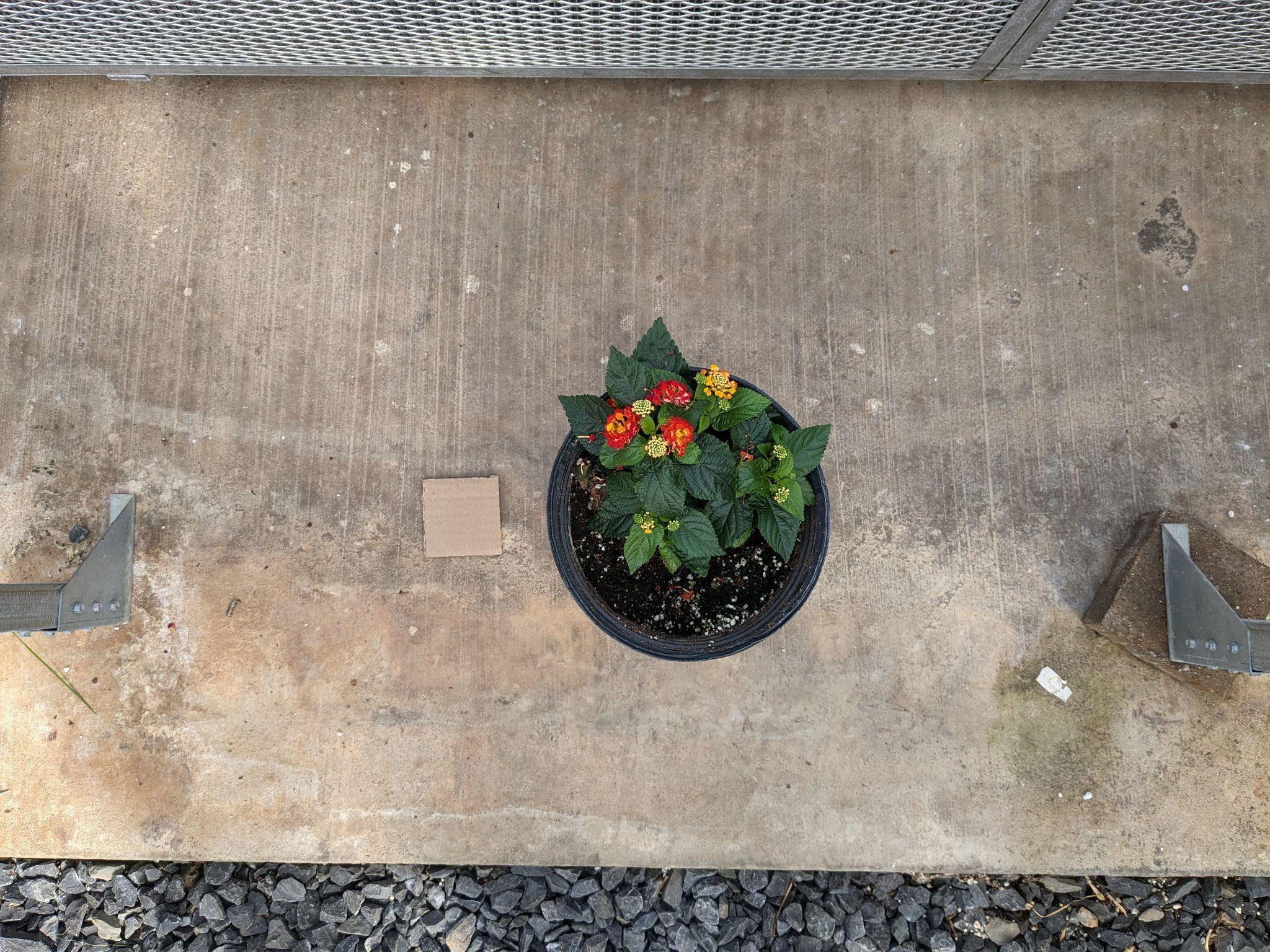 | 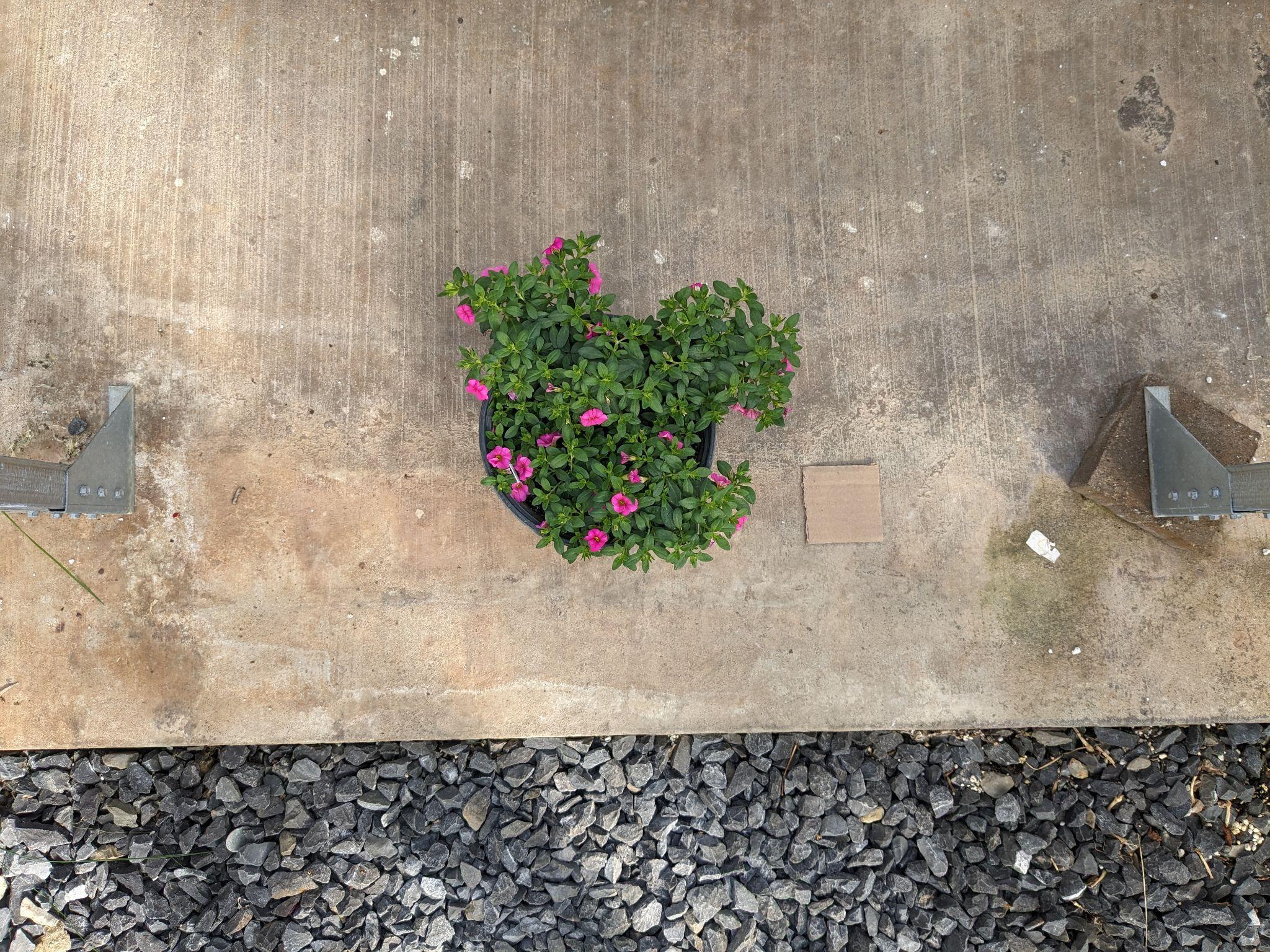 | 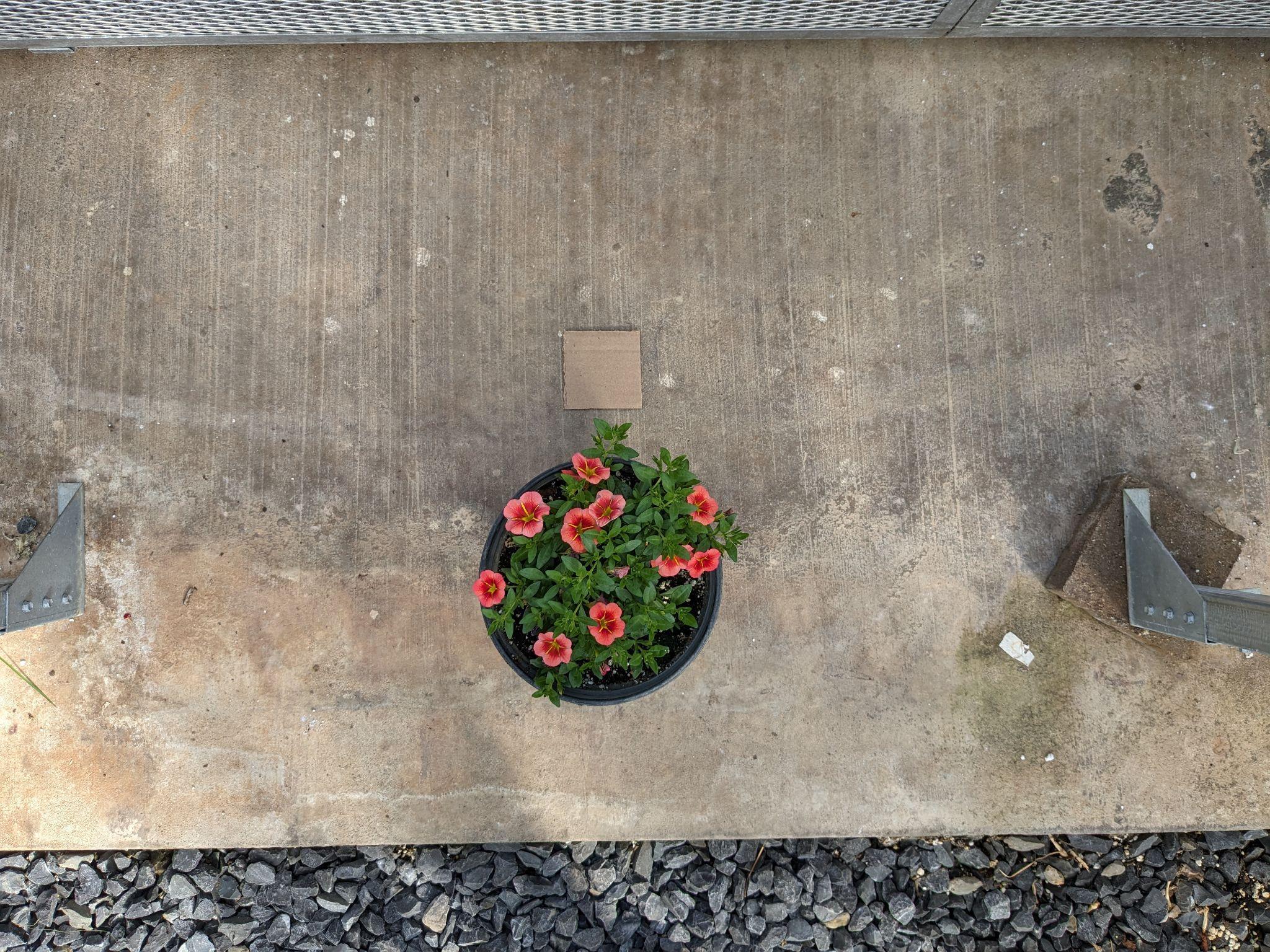 | 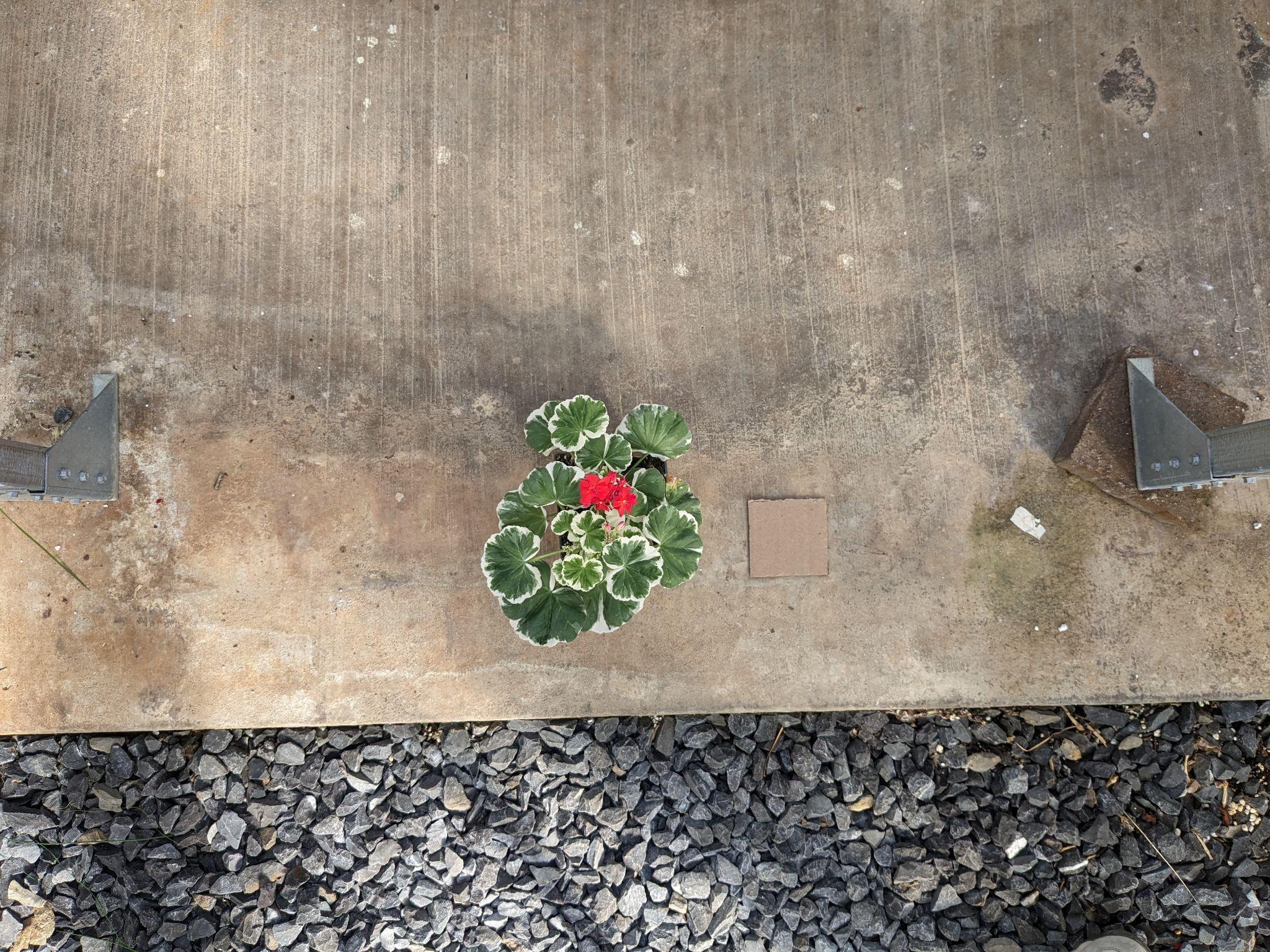 | 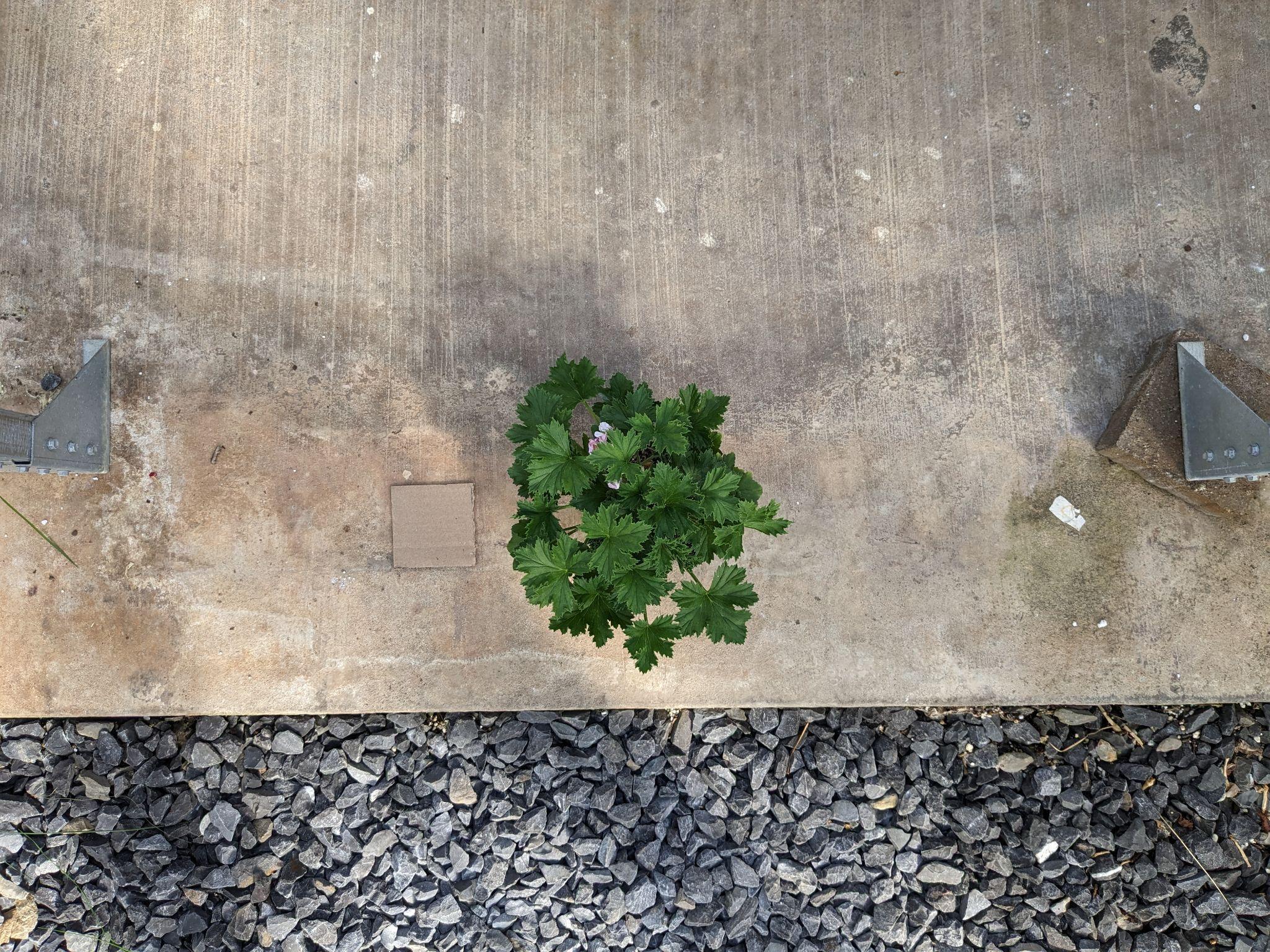 |
| 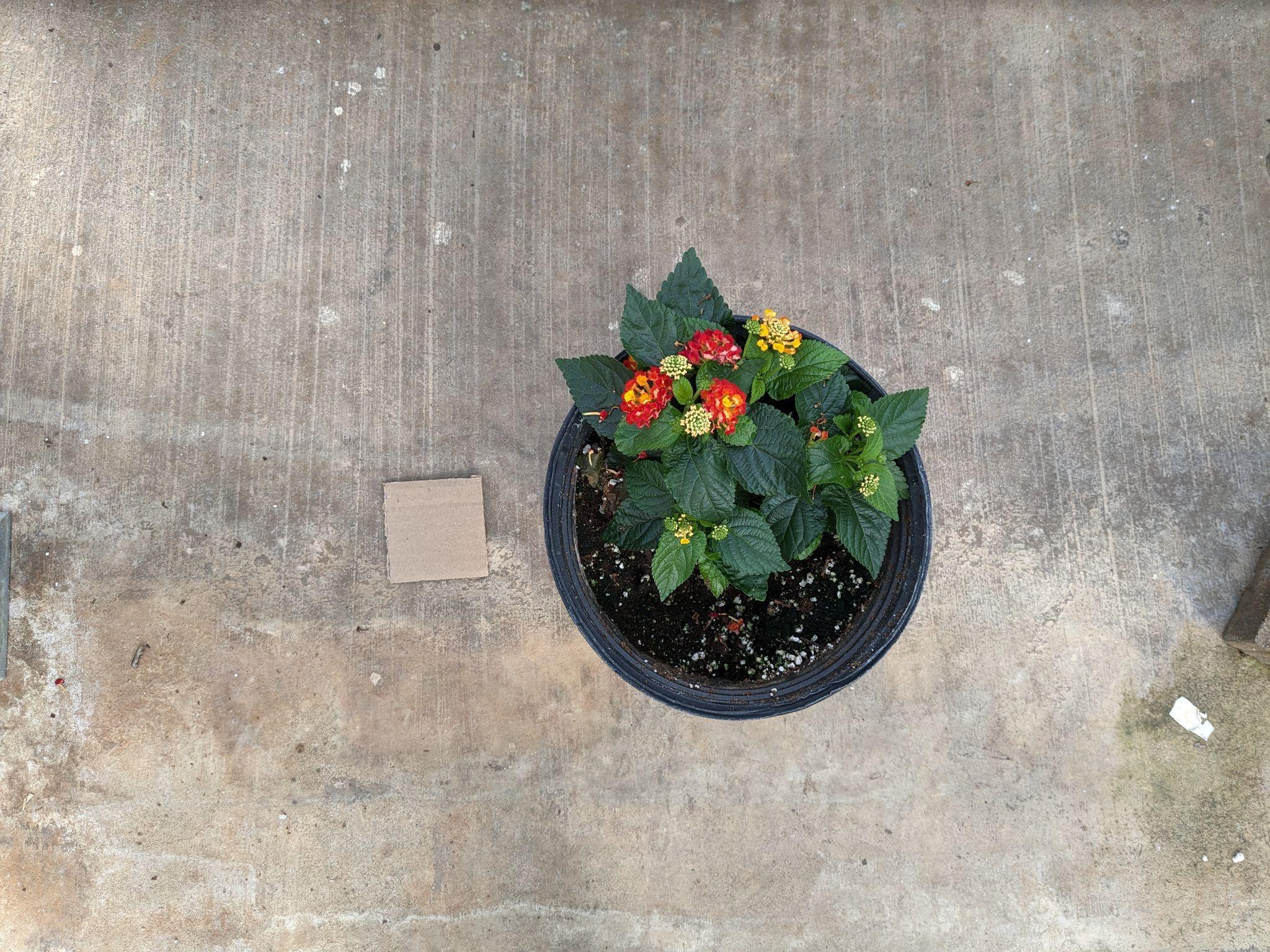 | 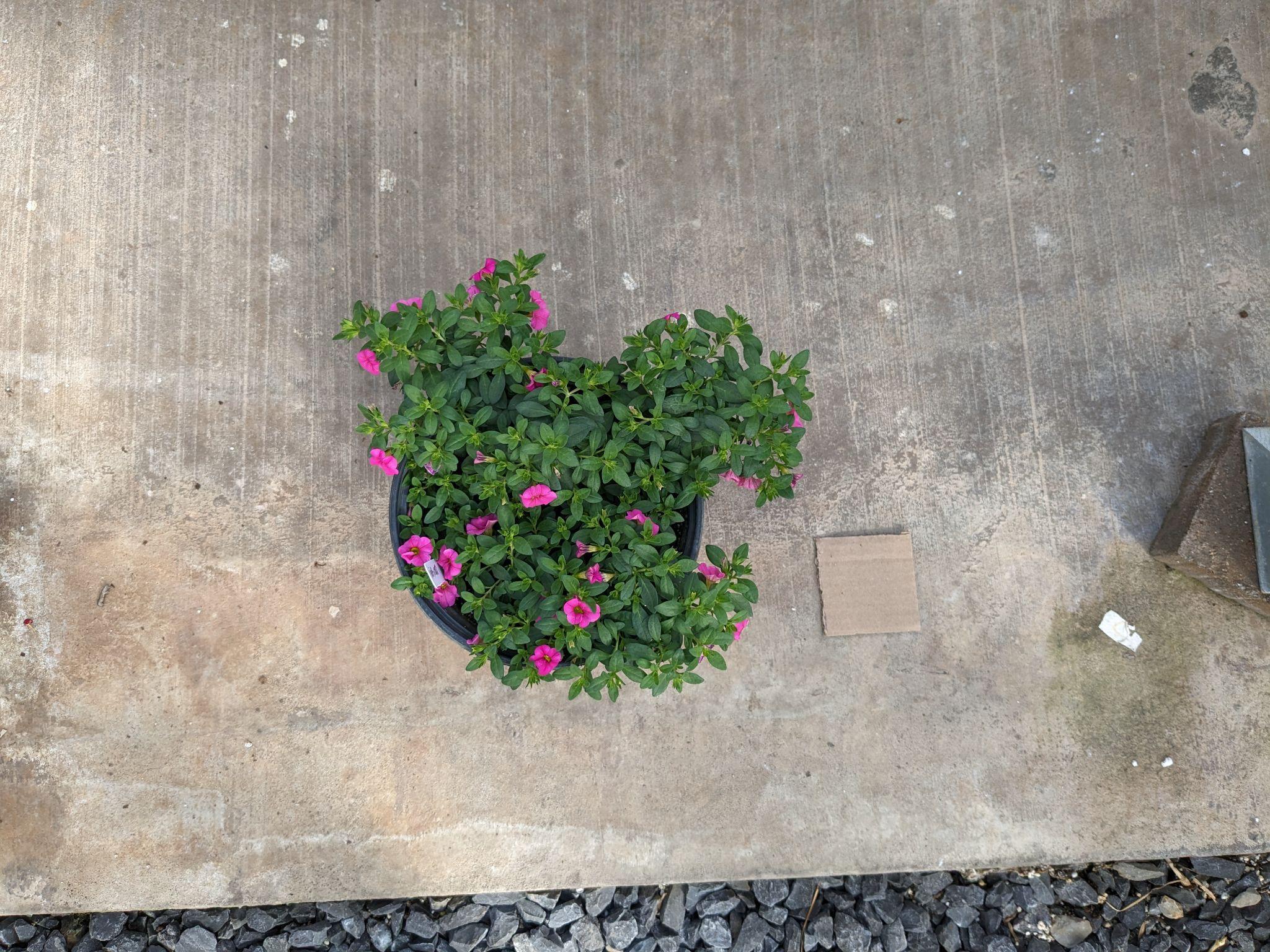 | 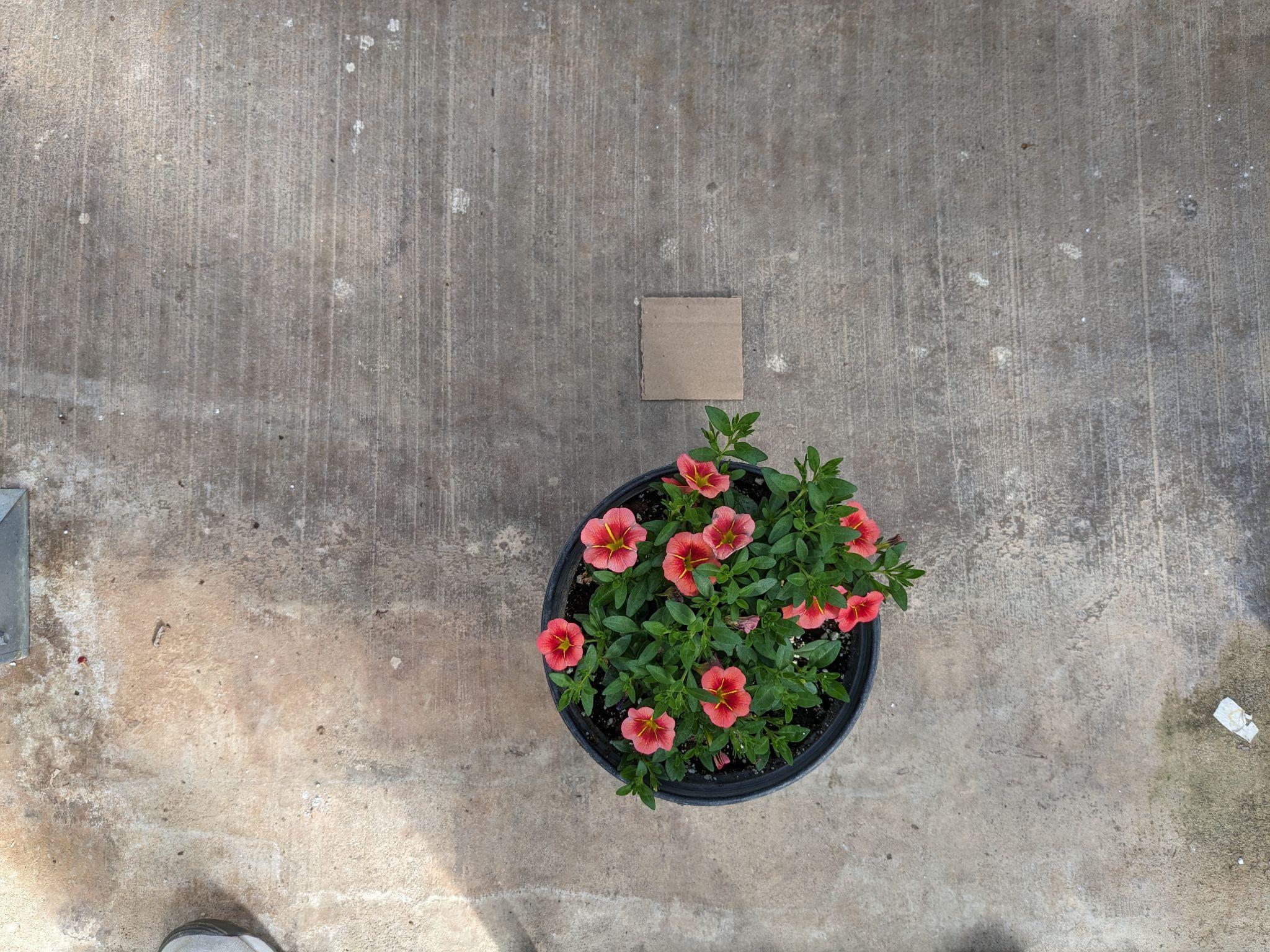 | 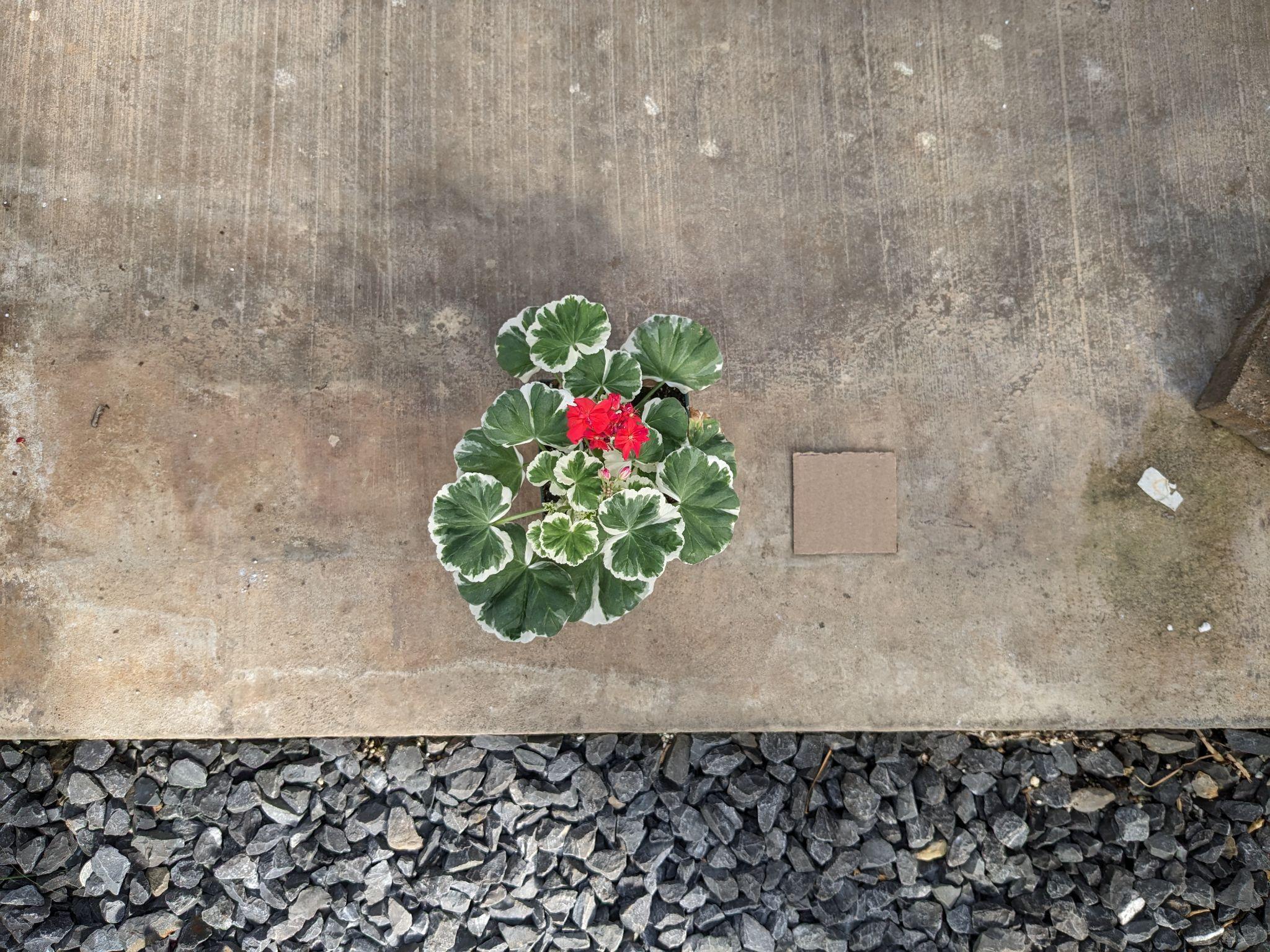 | 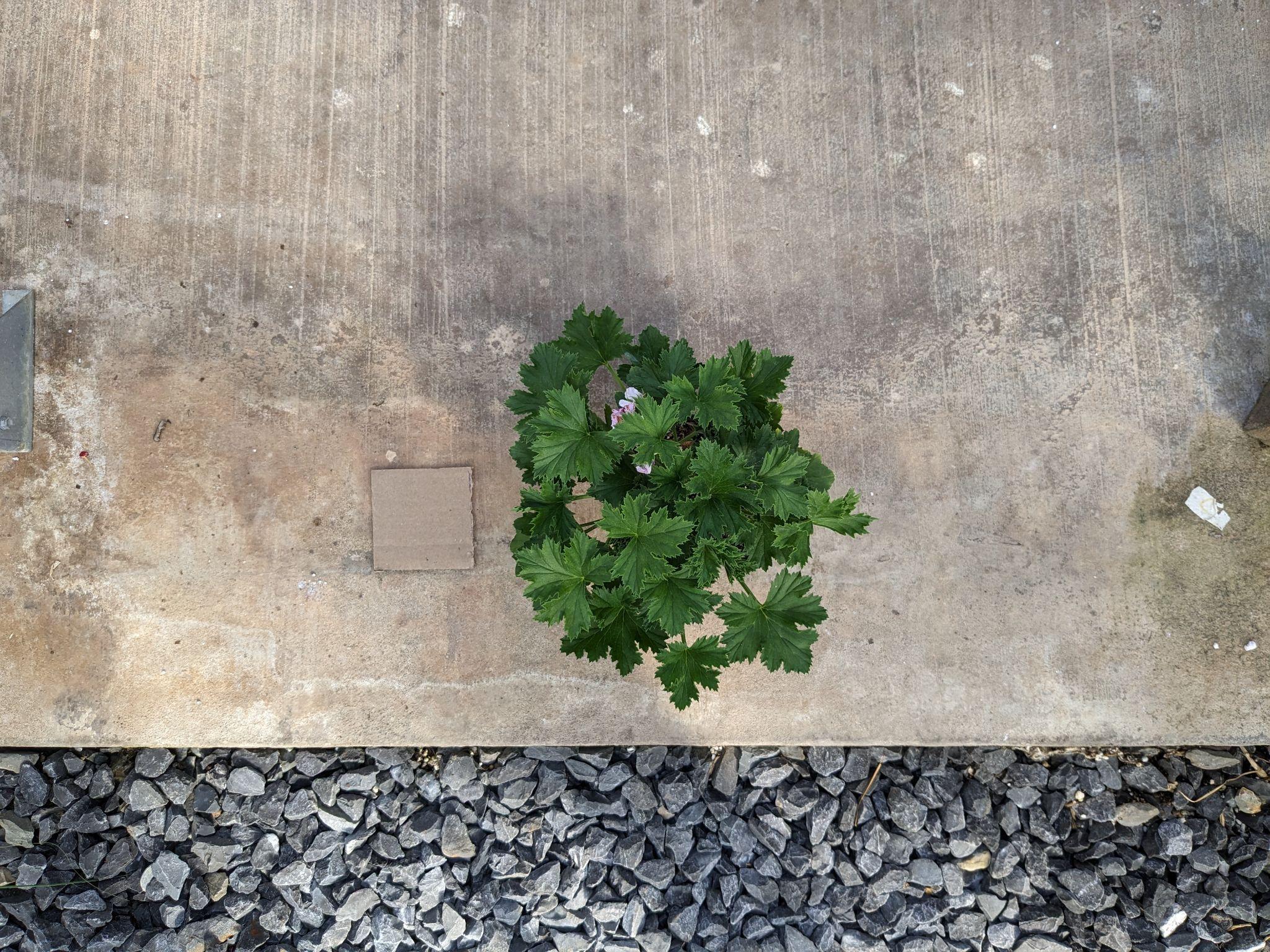 |
| 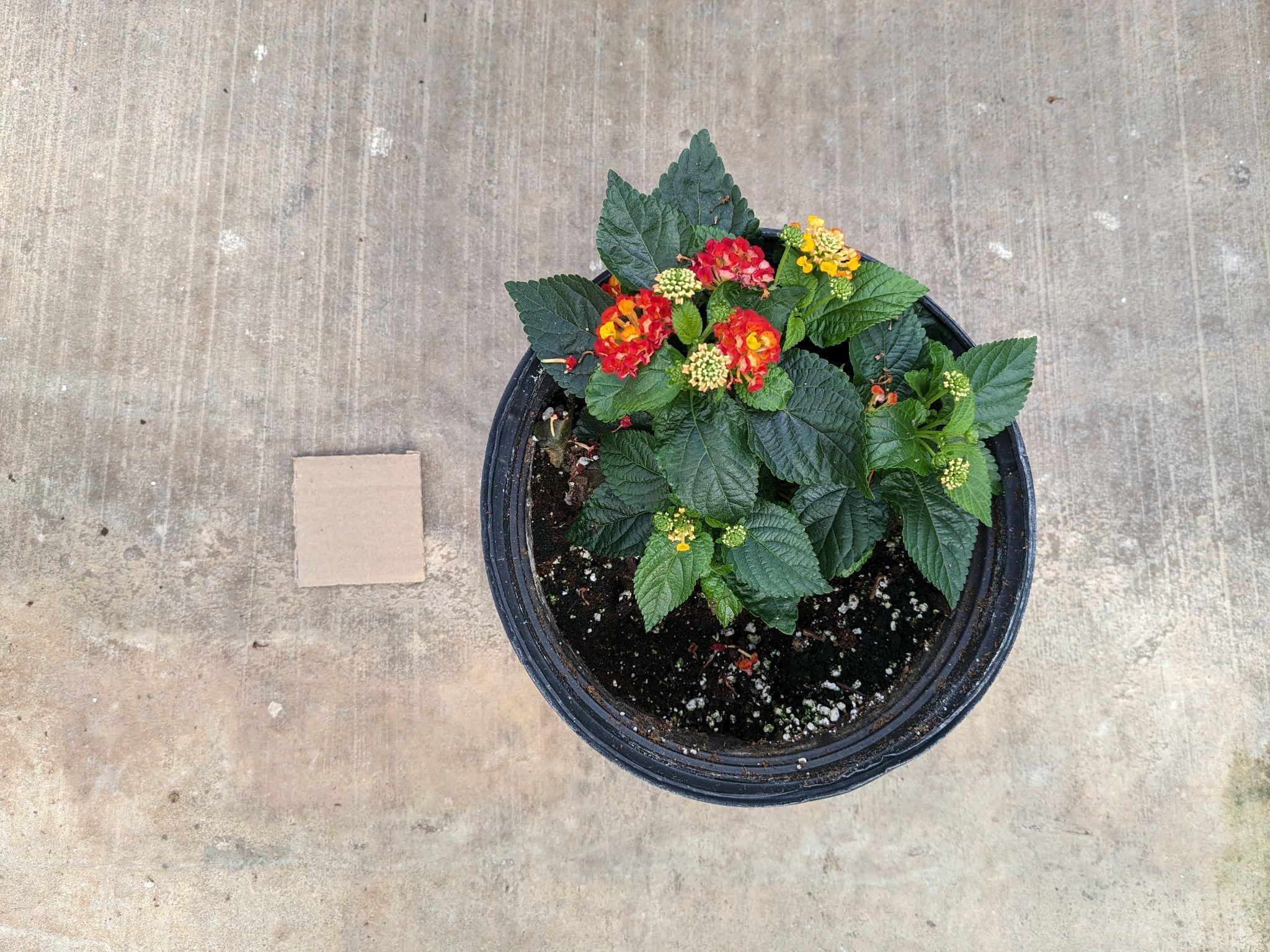 | 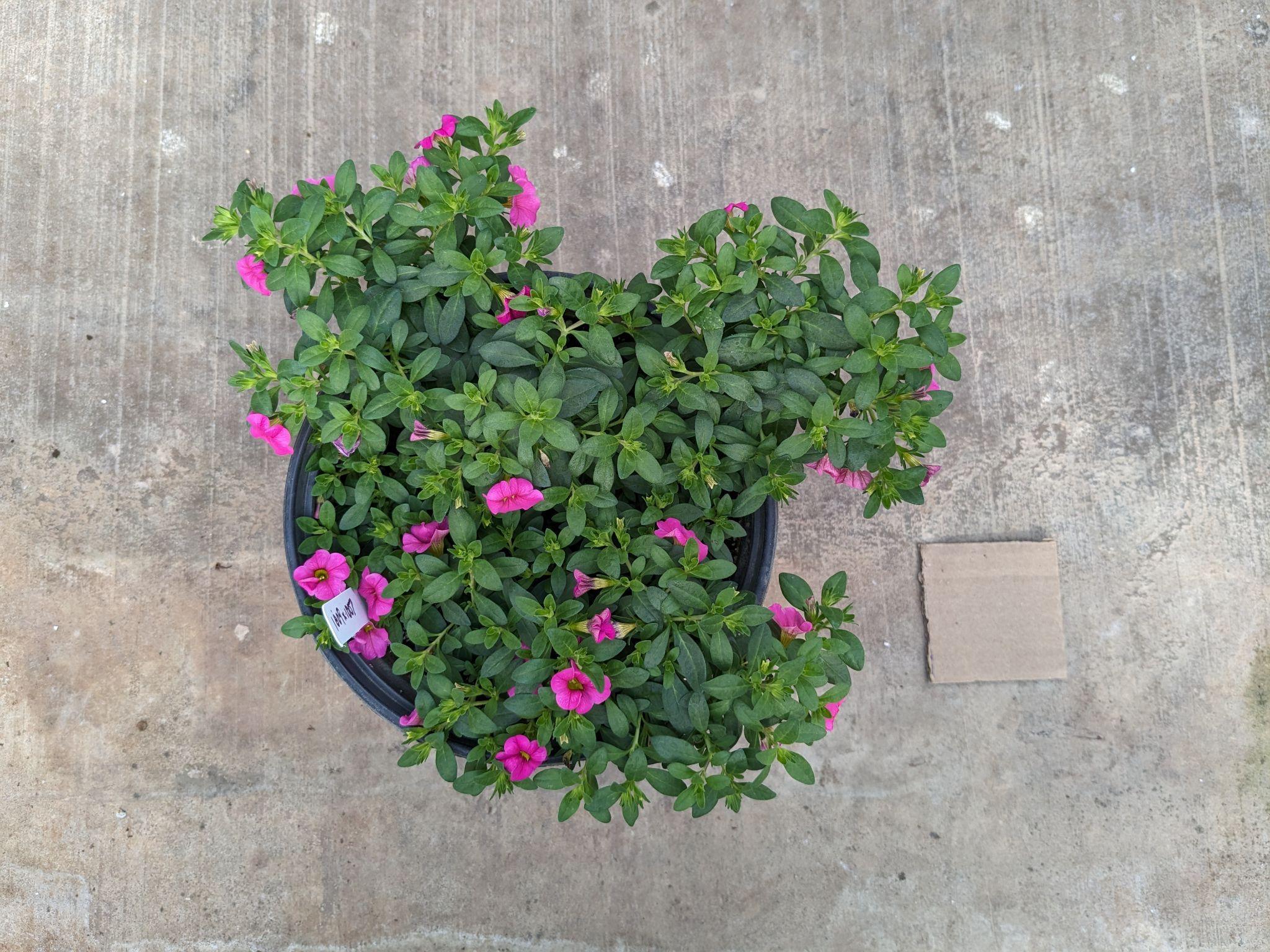 | 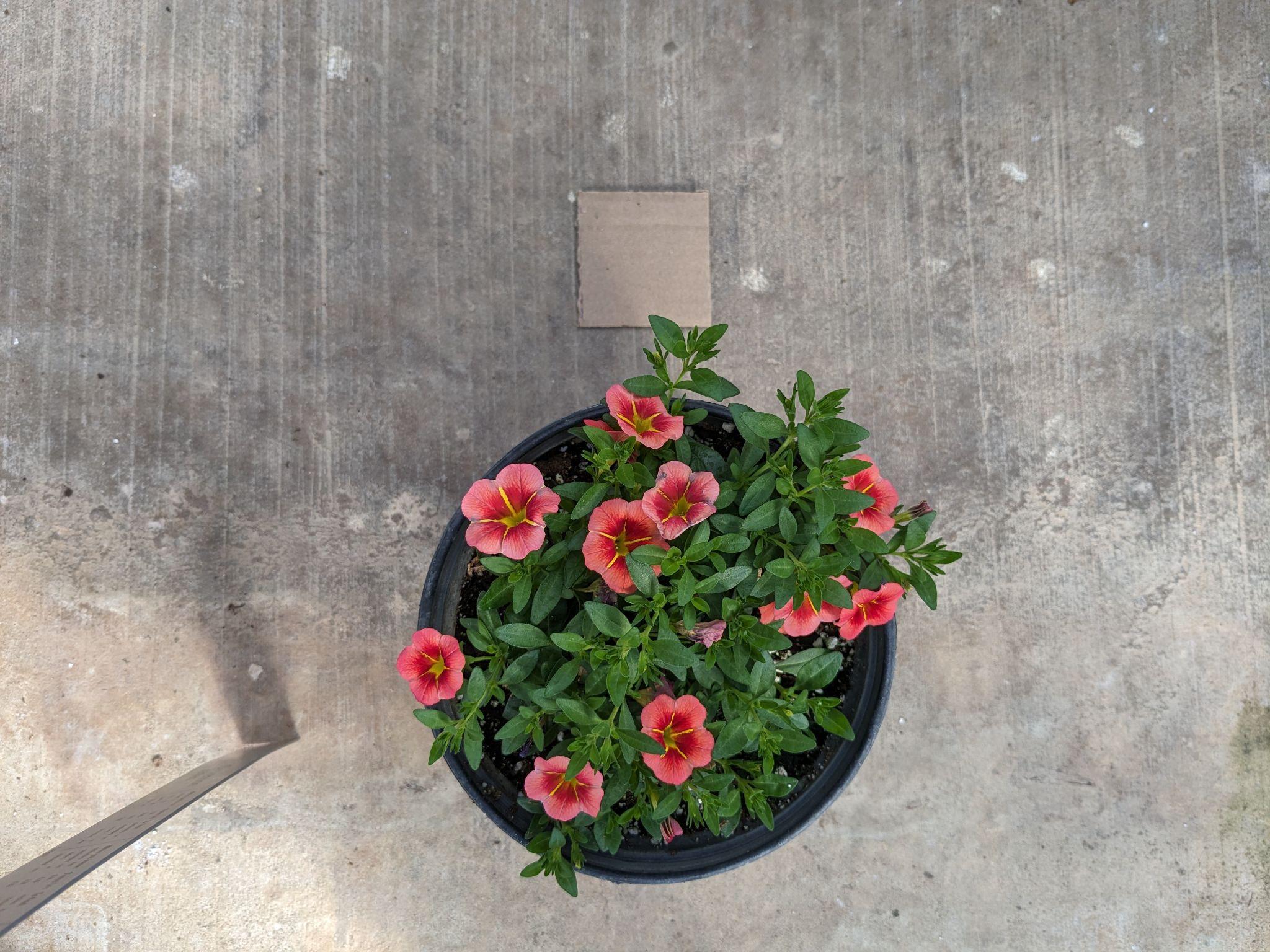 | 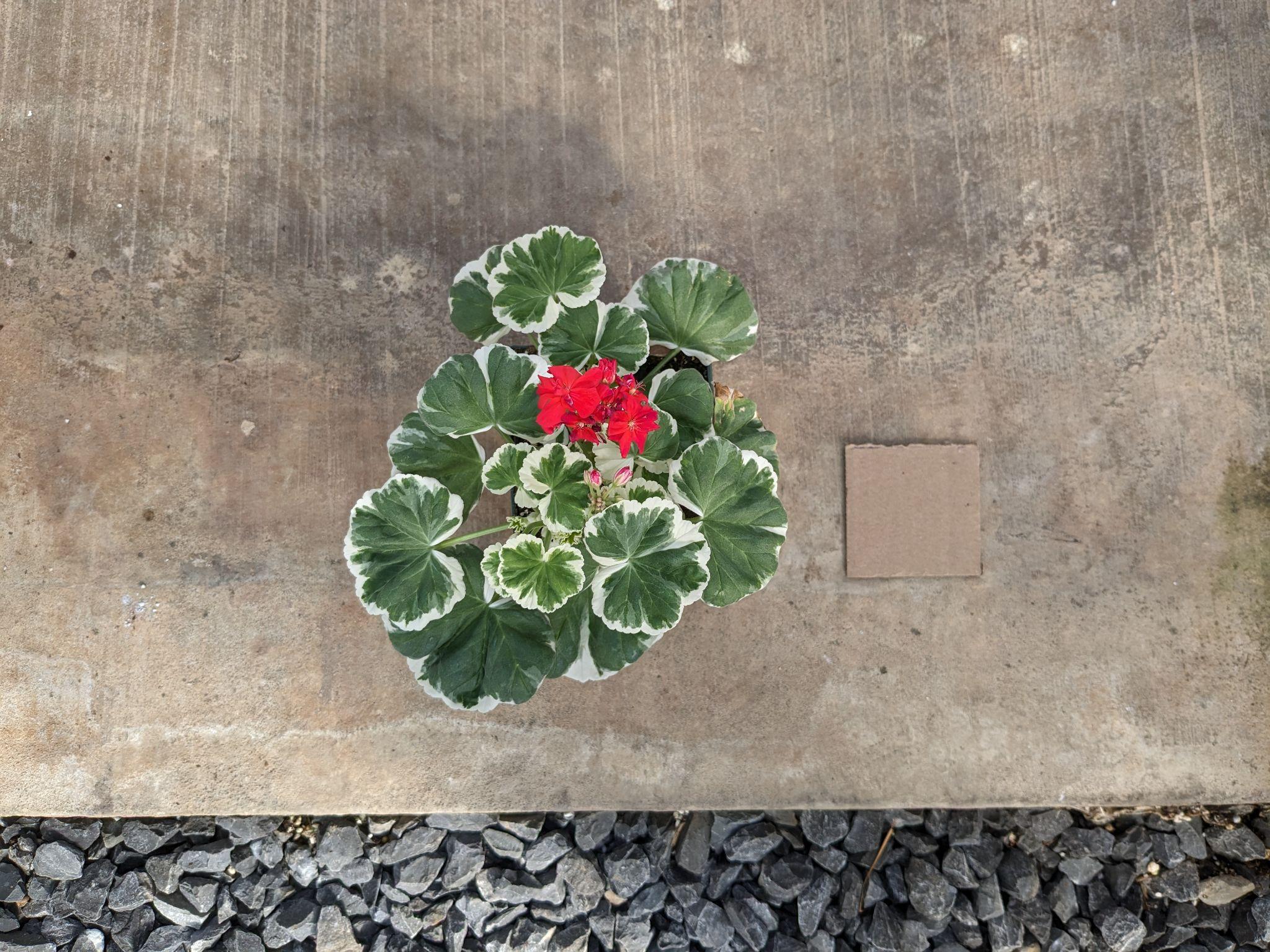 | 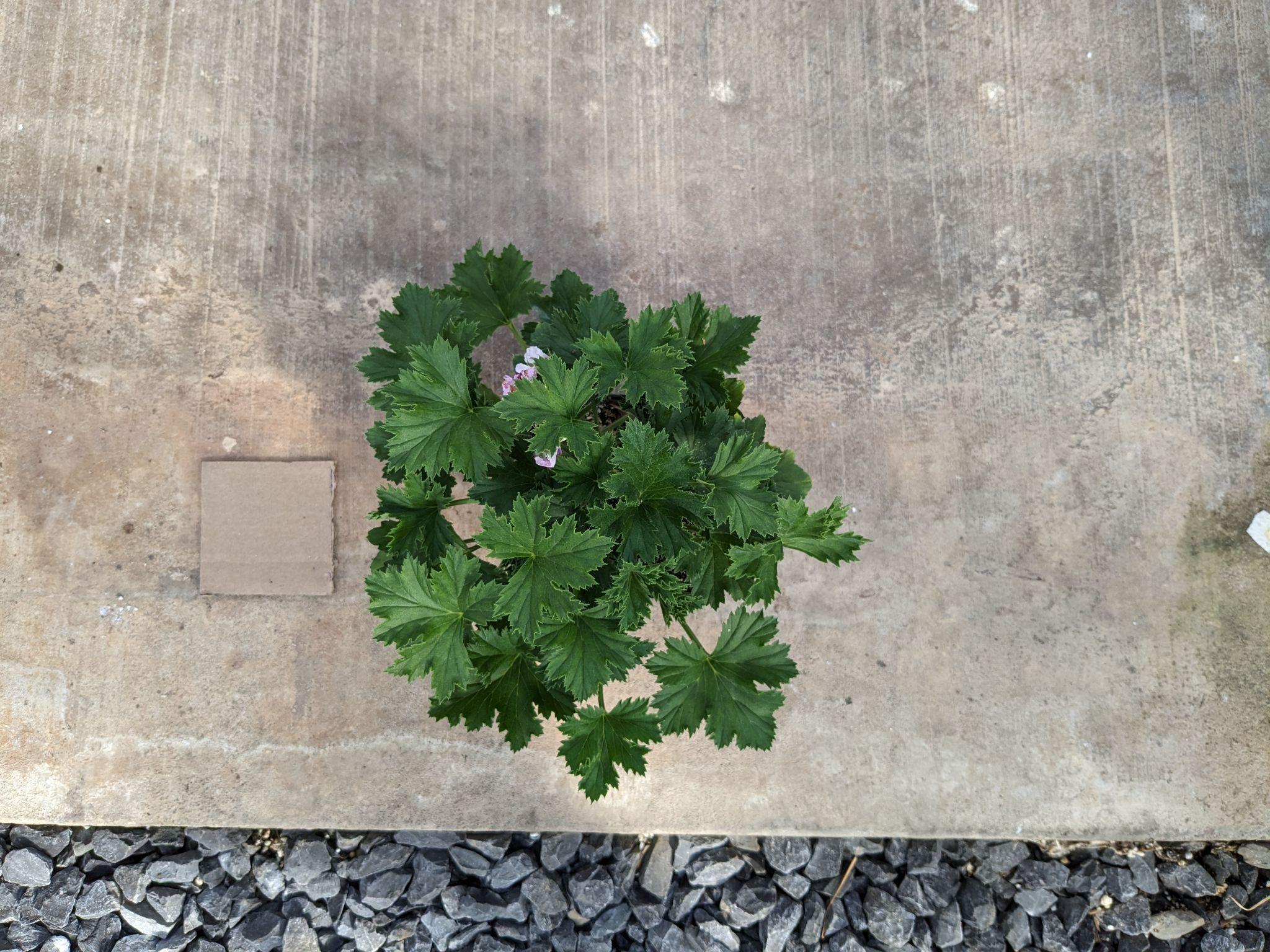 |
| 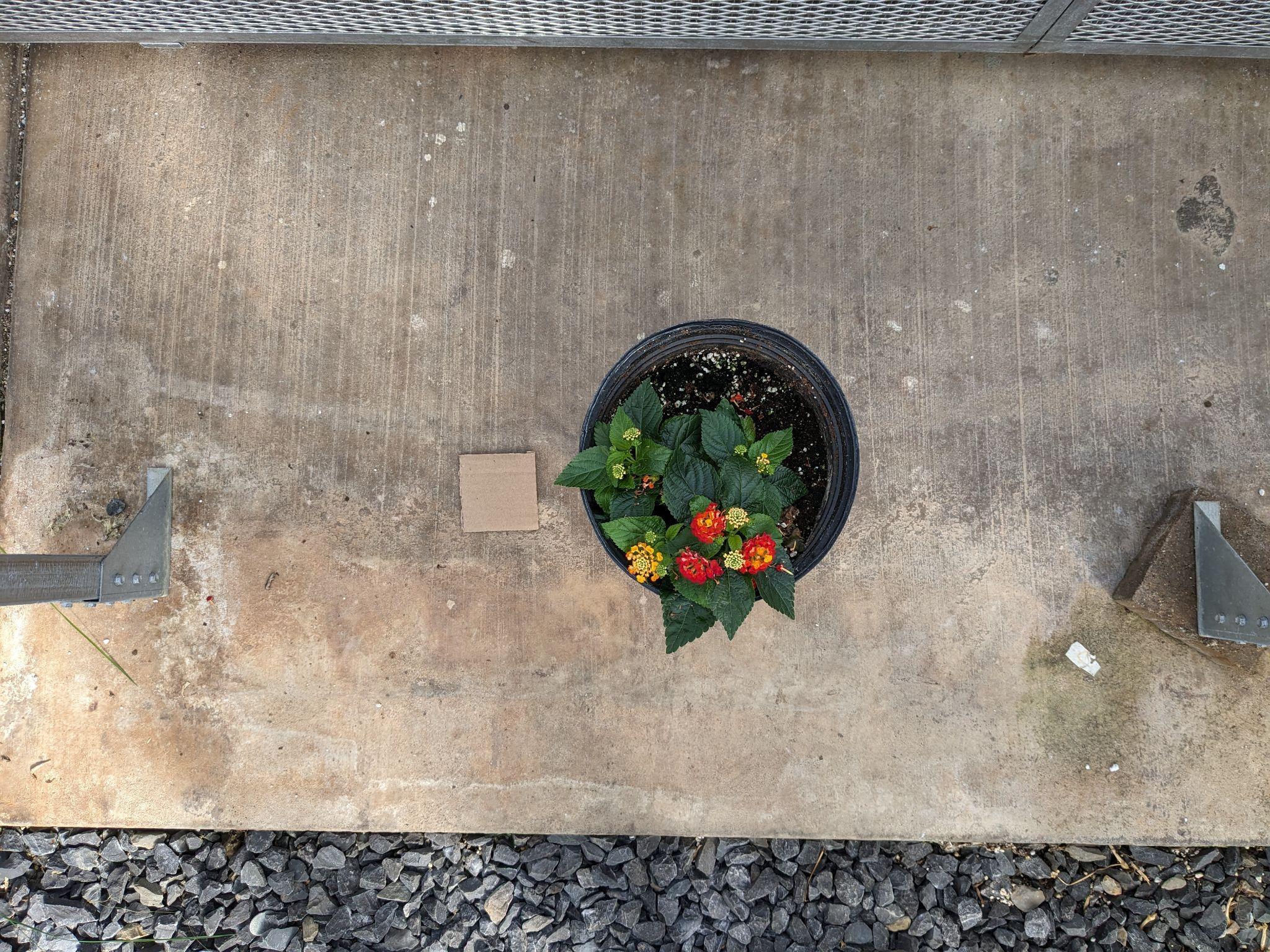 | 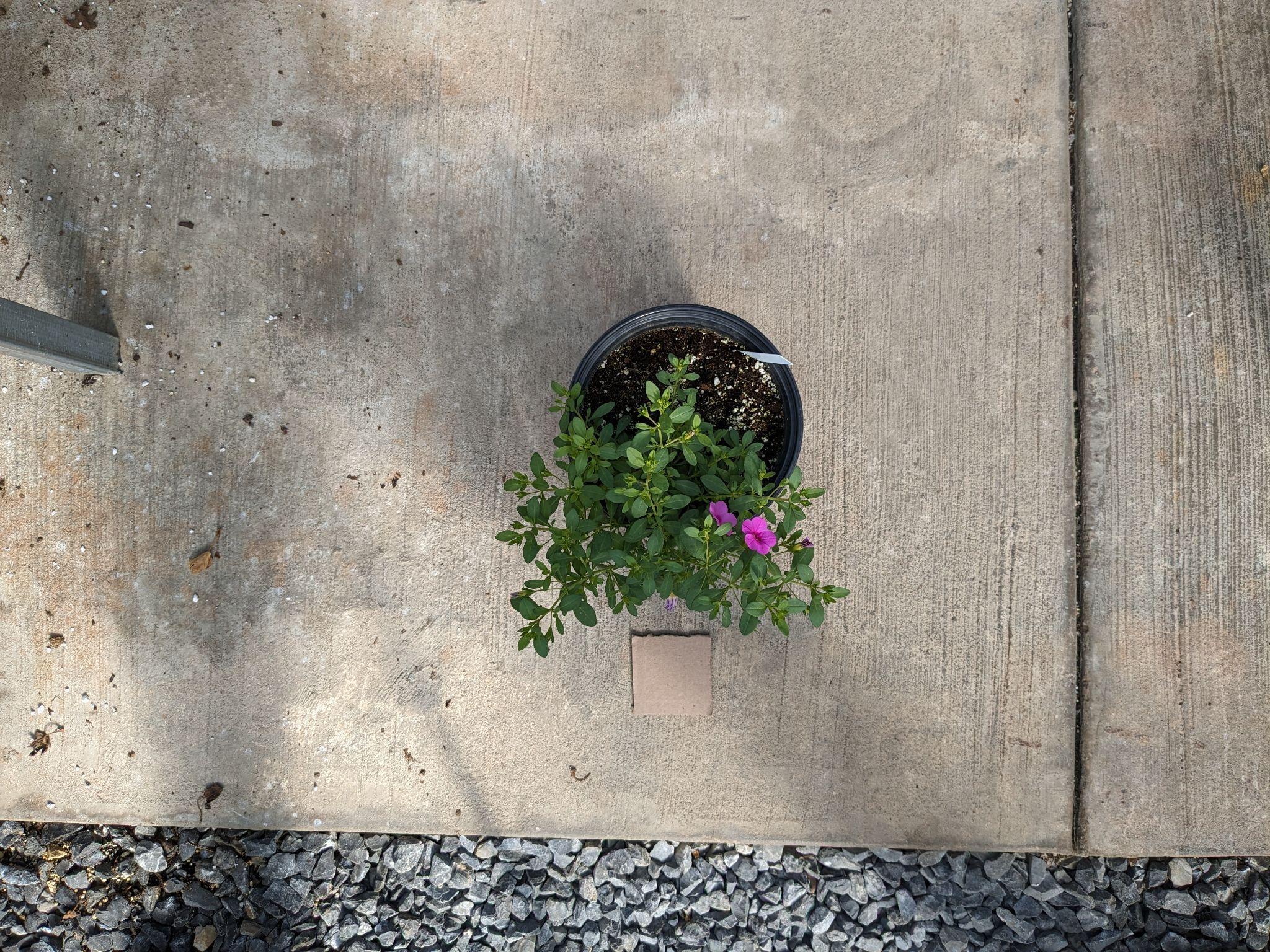 | 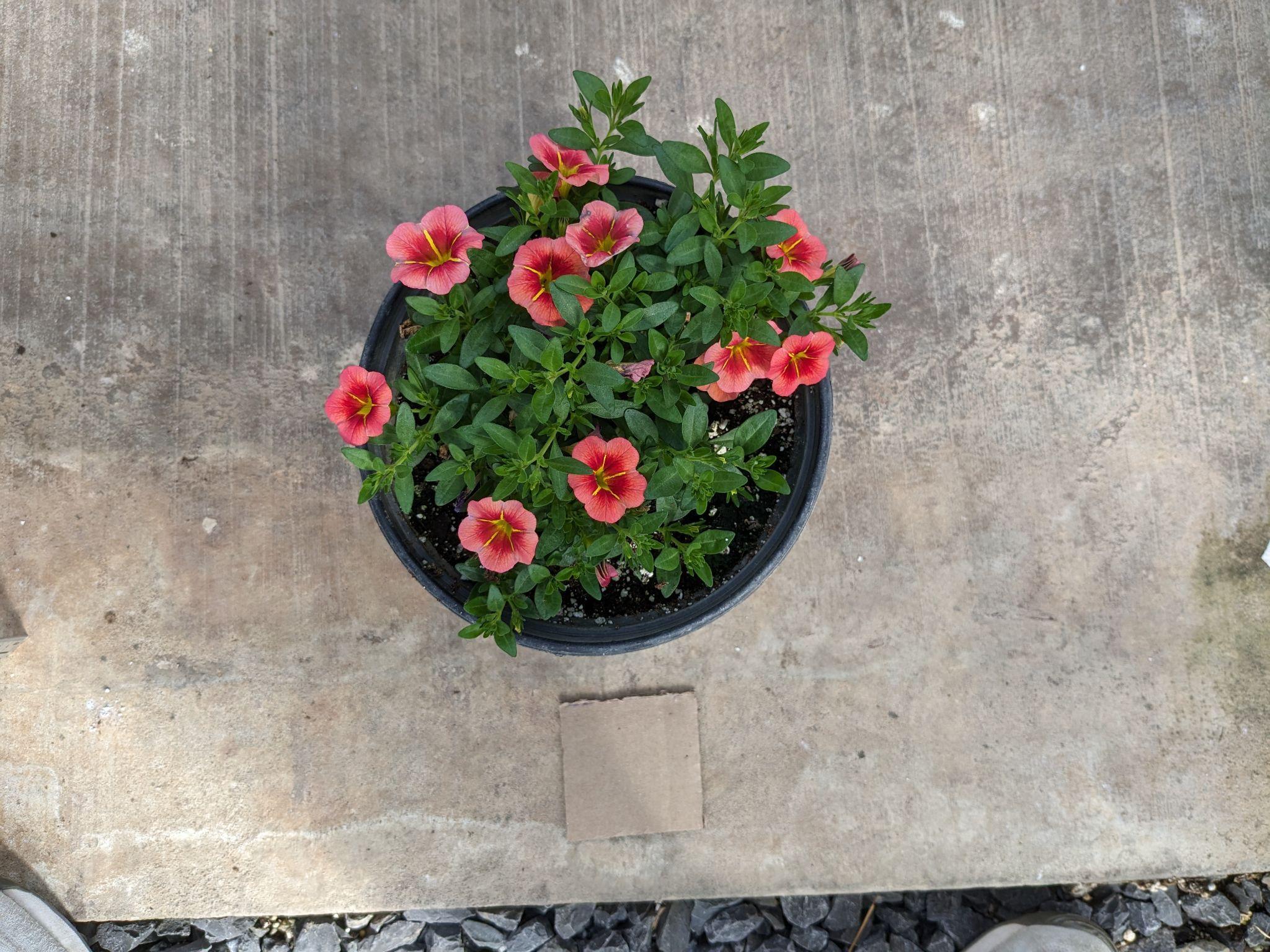 | 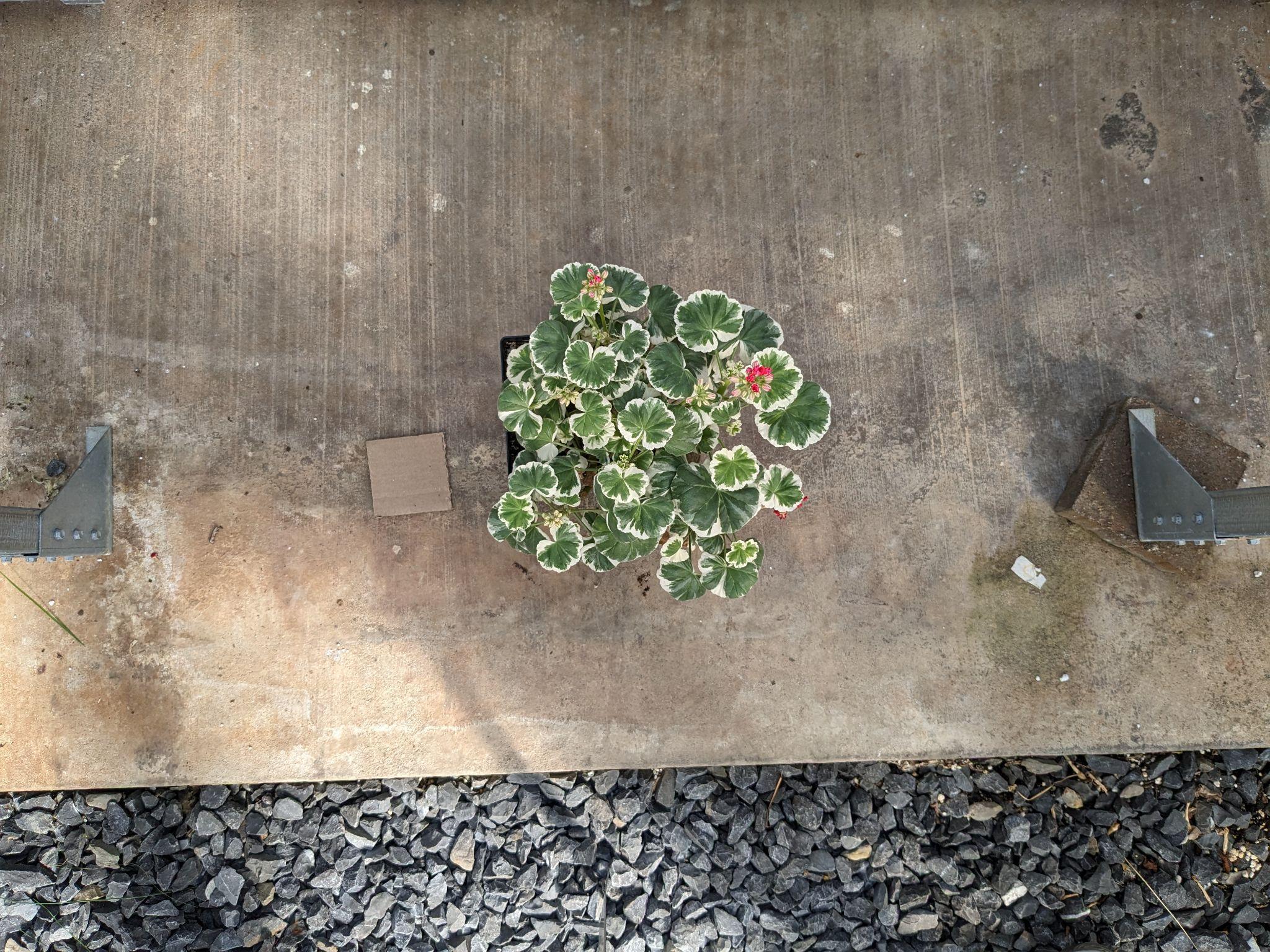 | 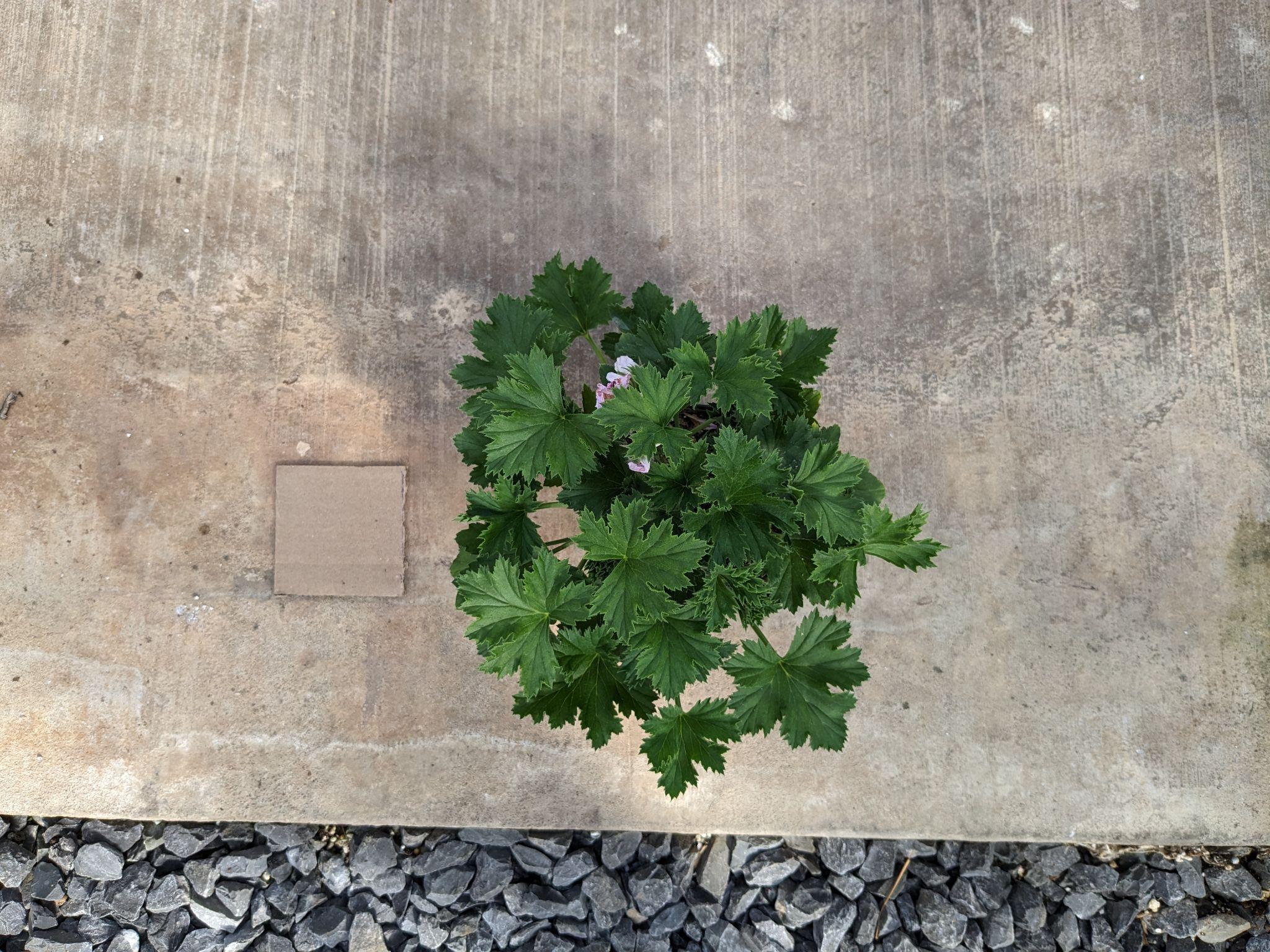 |
| 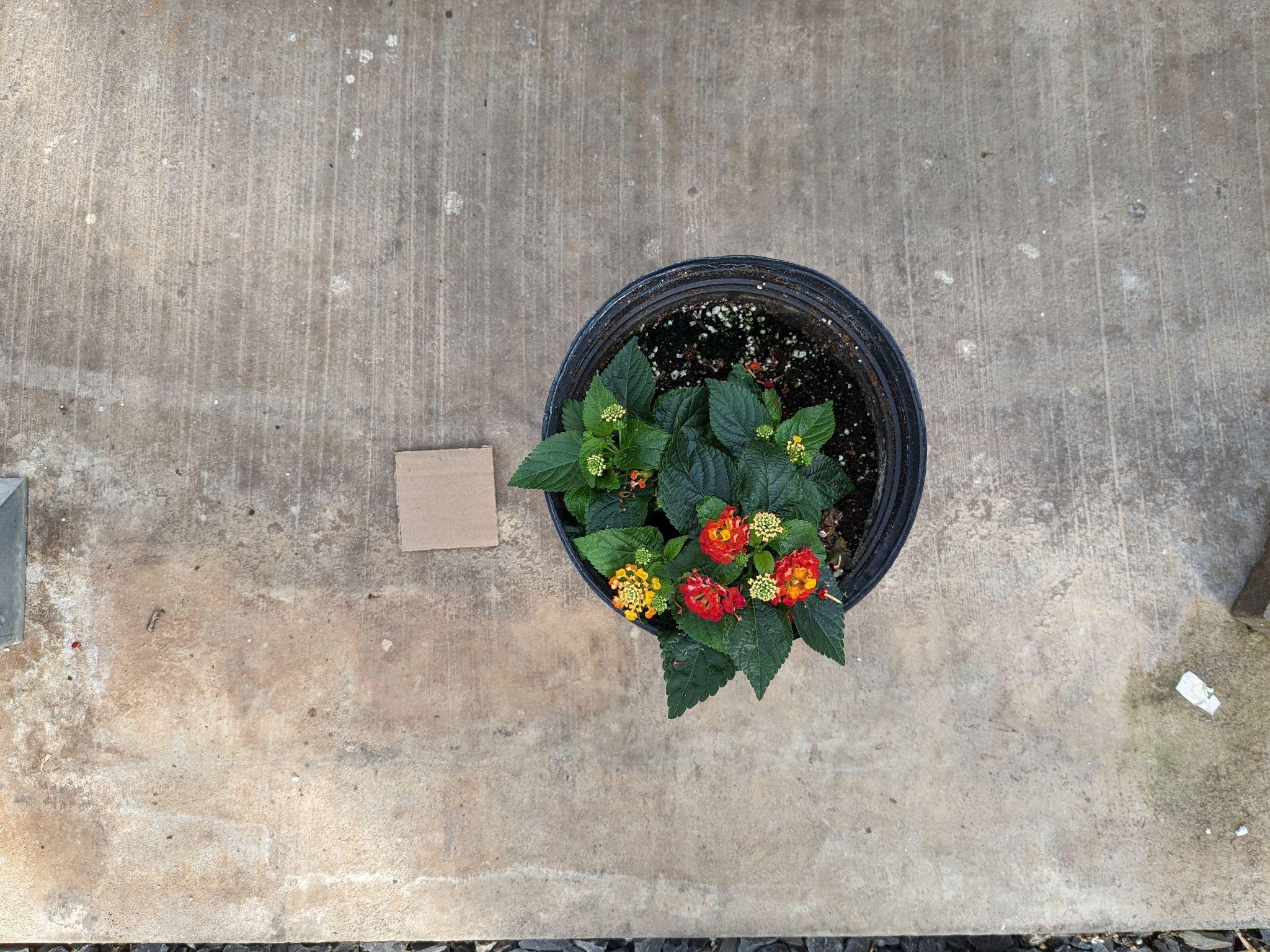 | 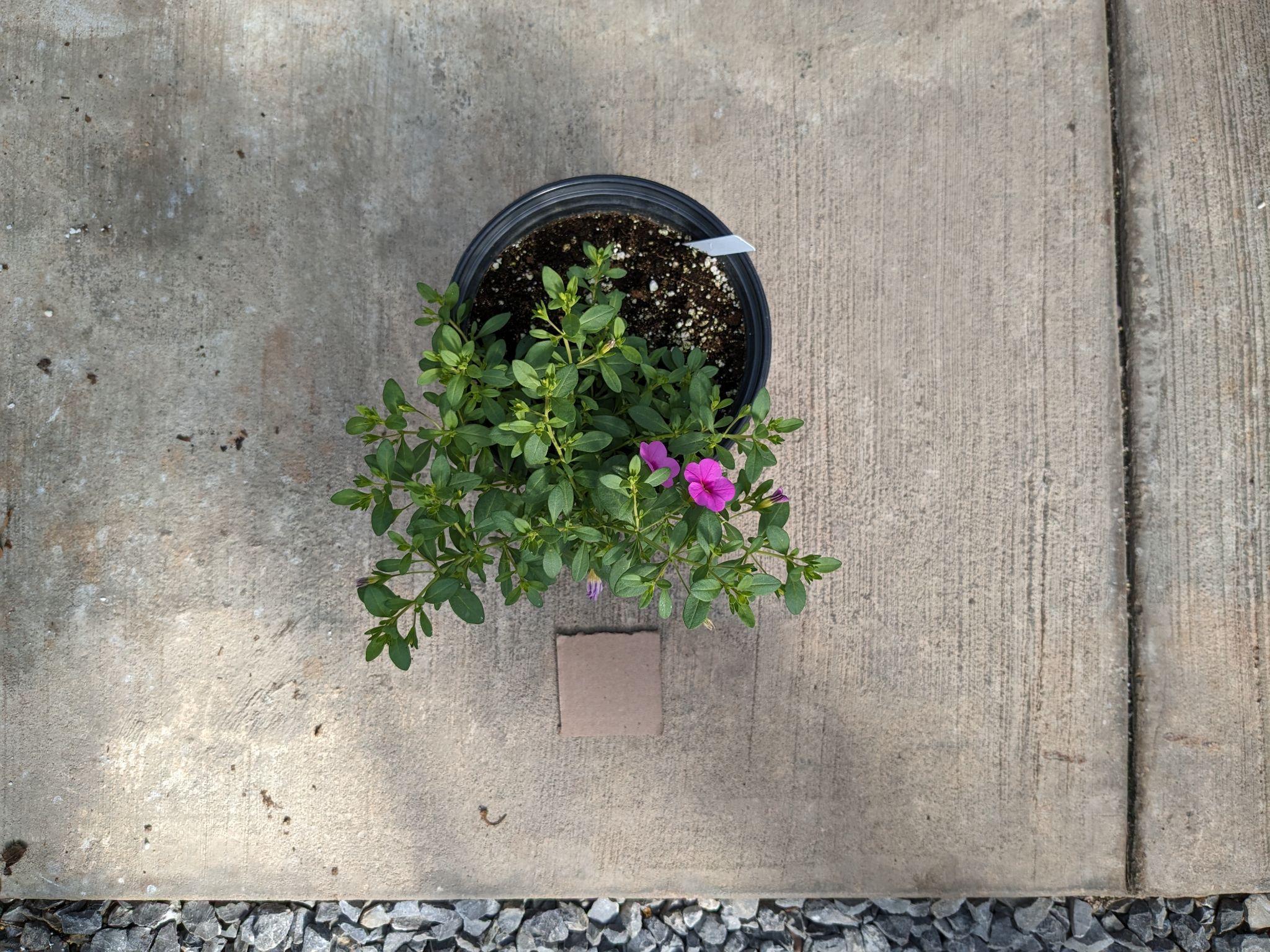 | 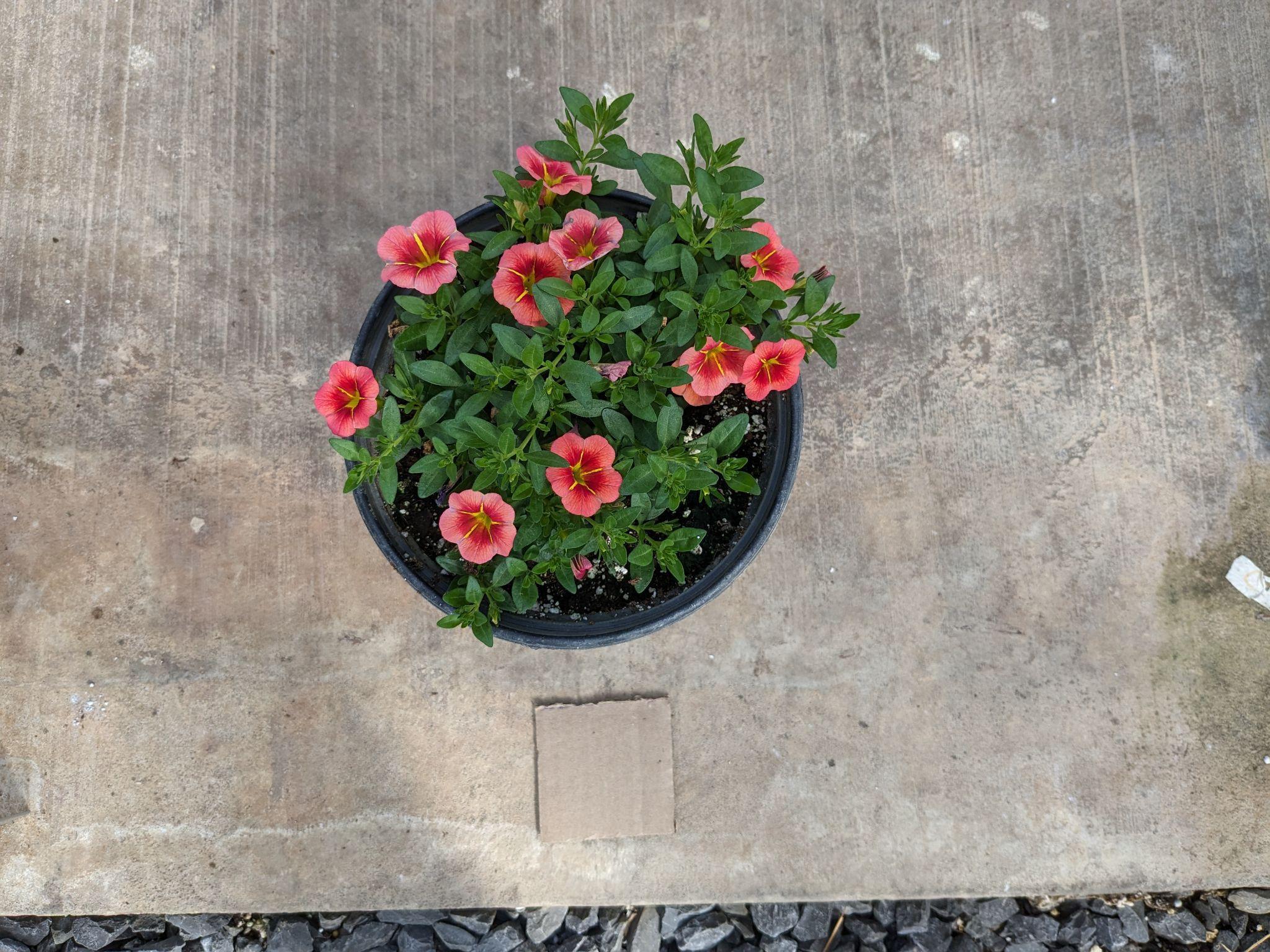 | 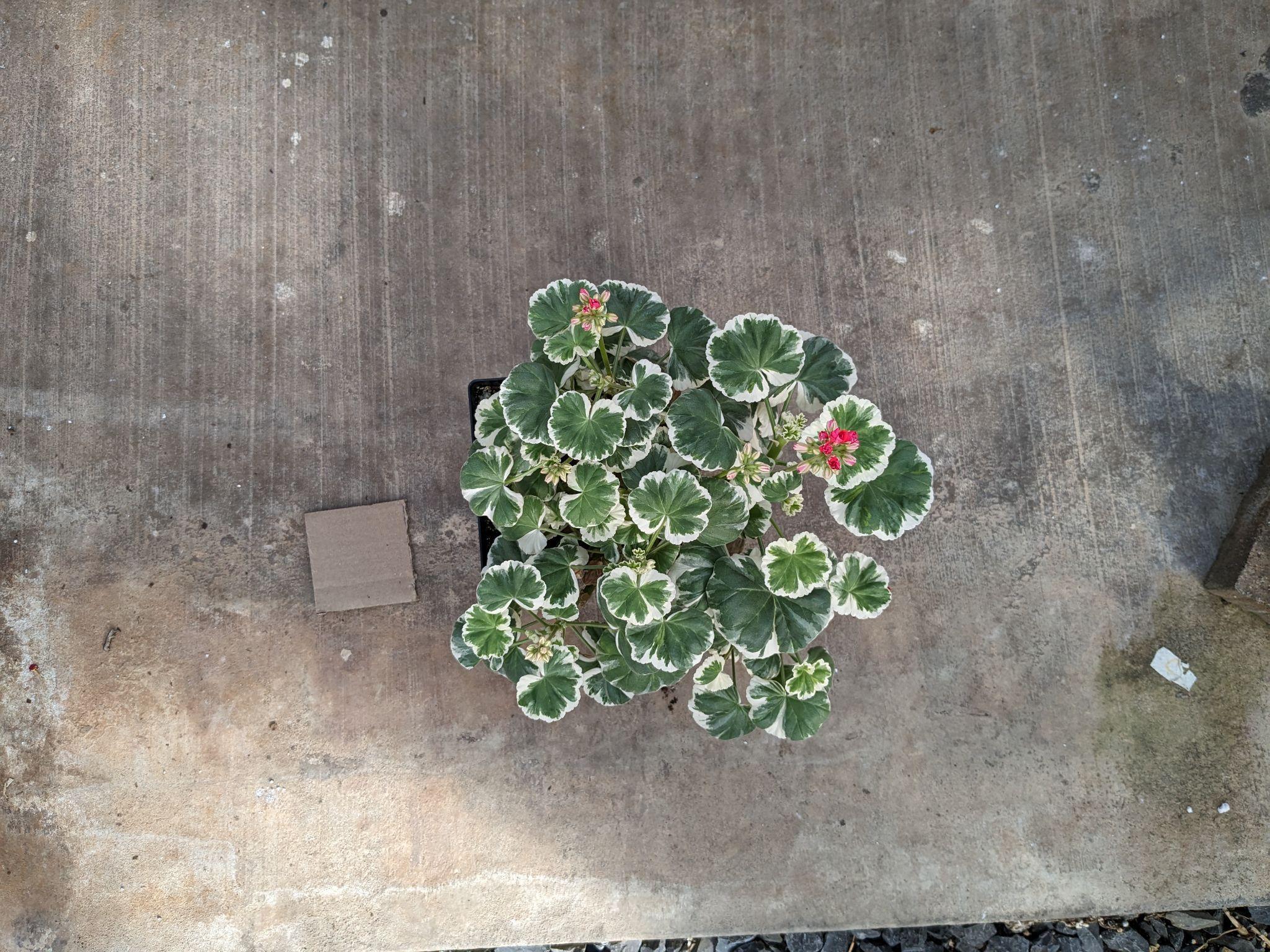 |  |
| 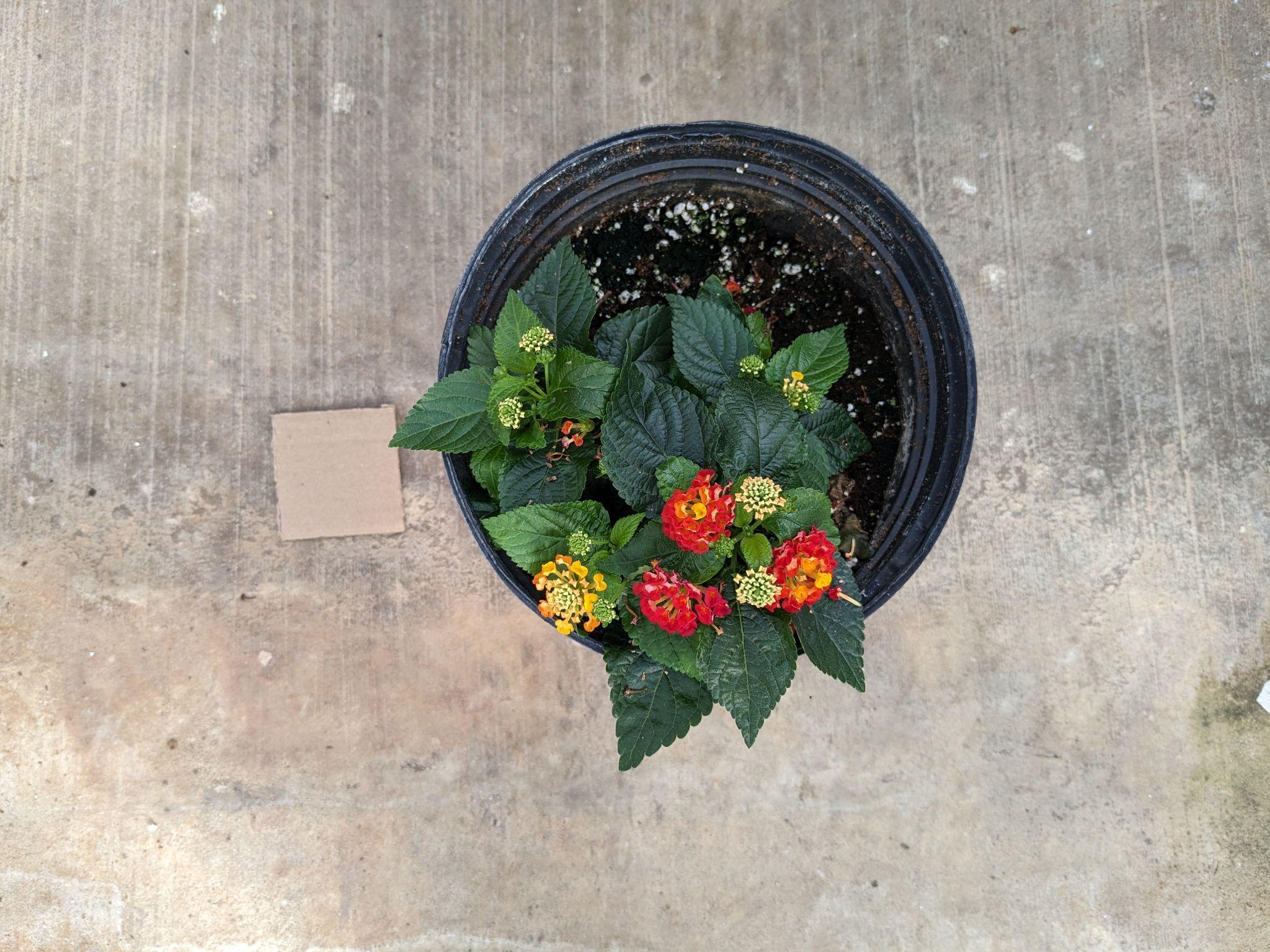 | 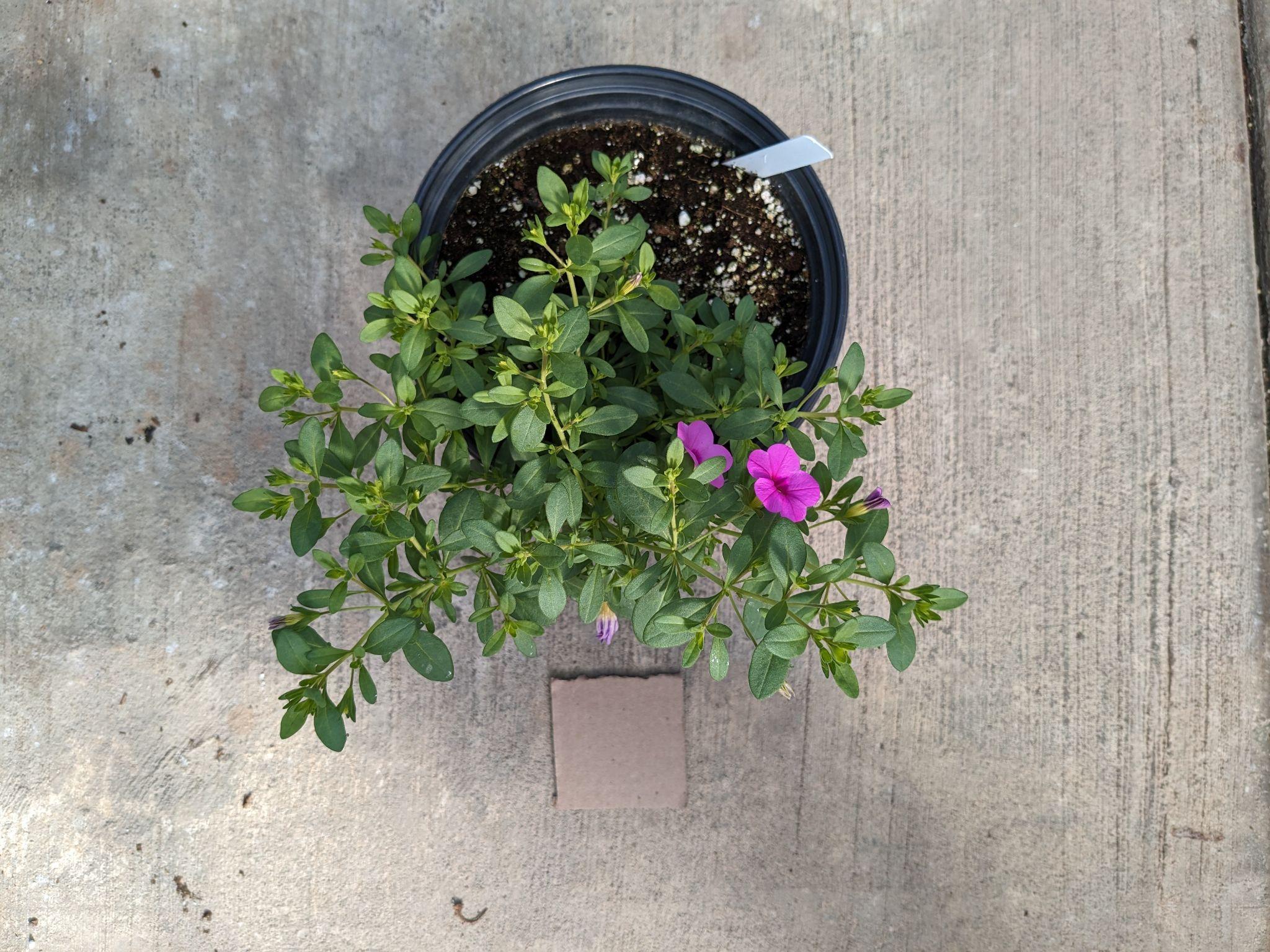 | 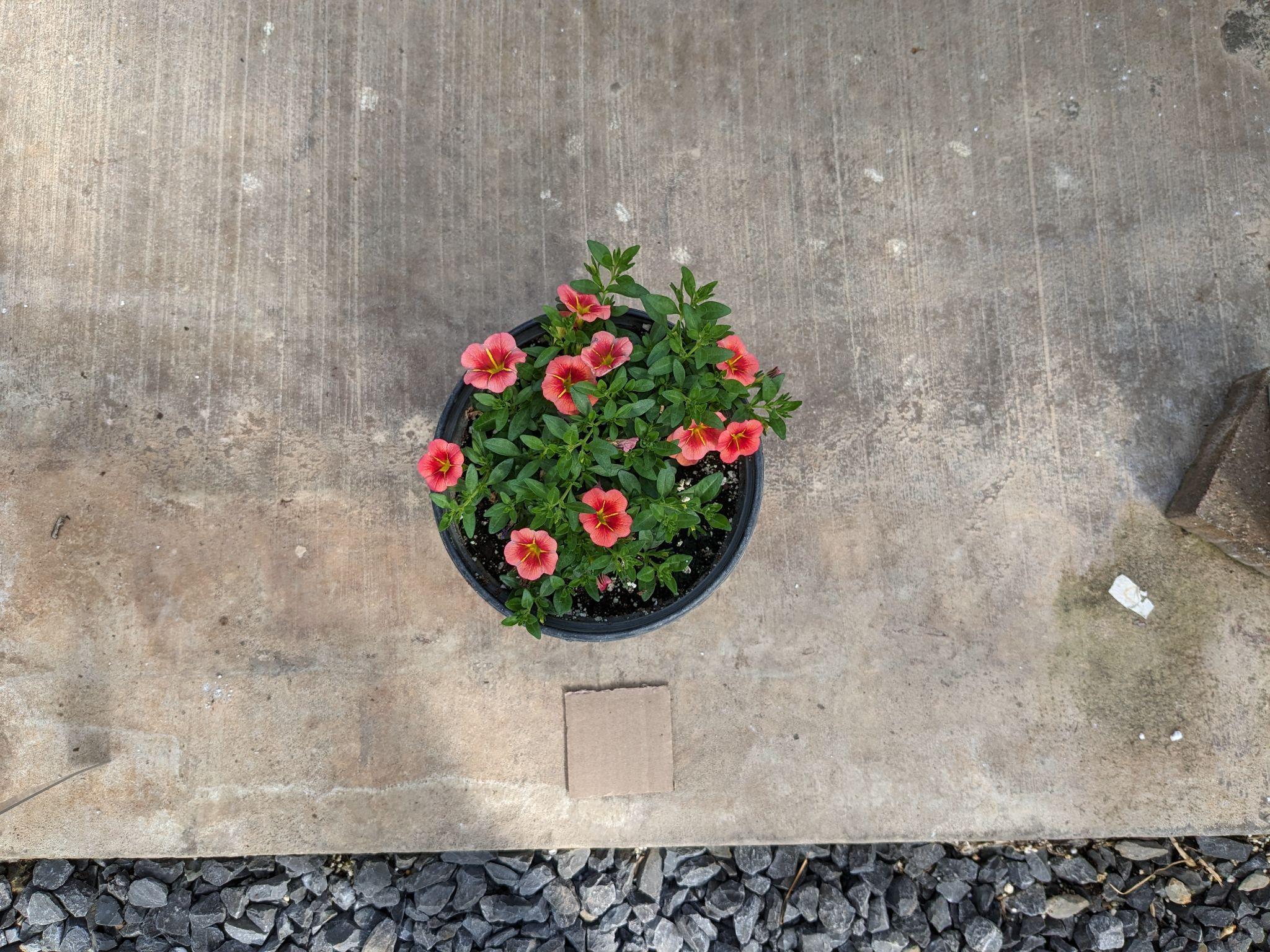 | 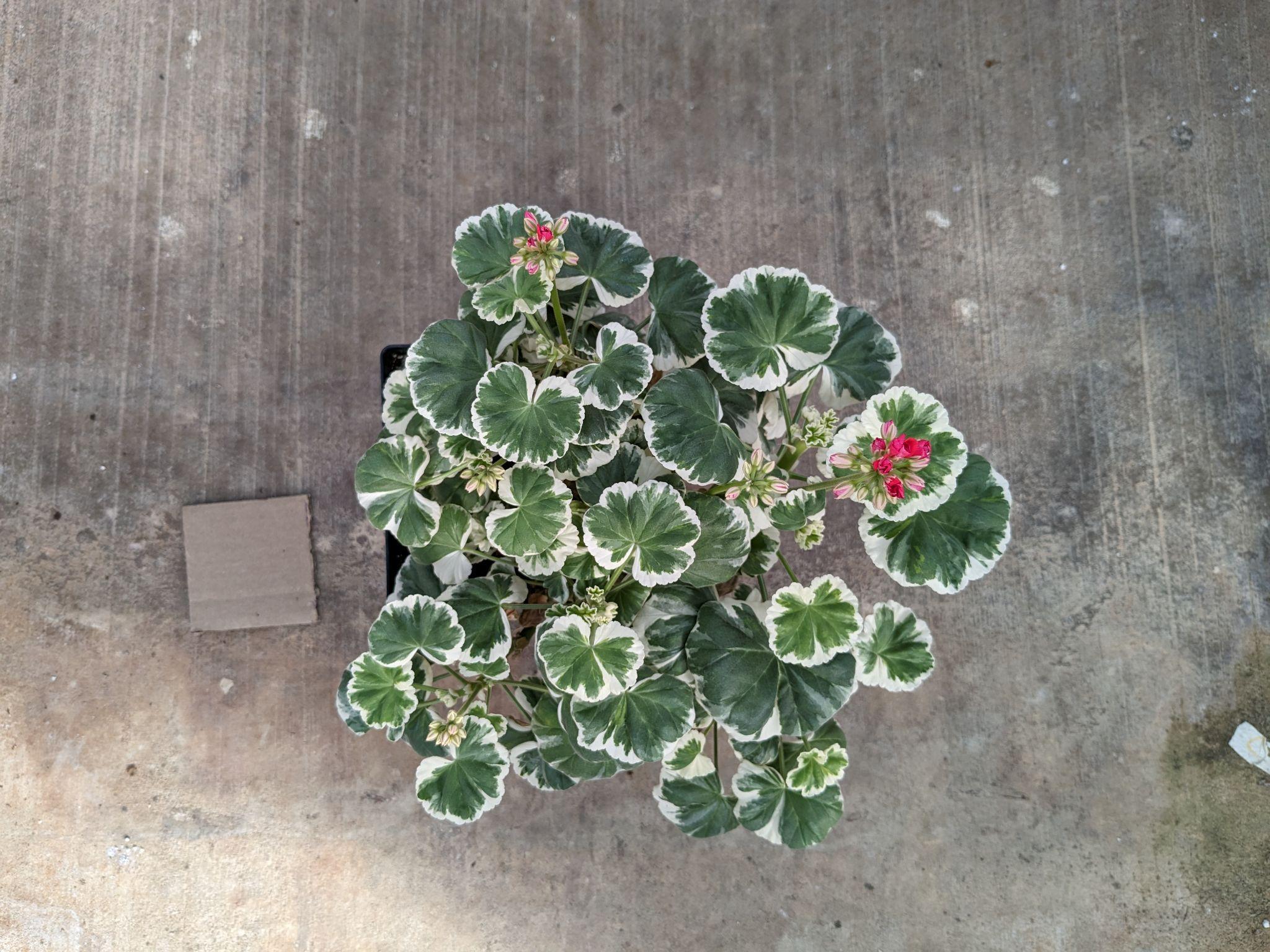 |  |
| 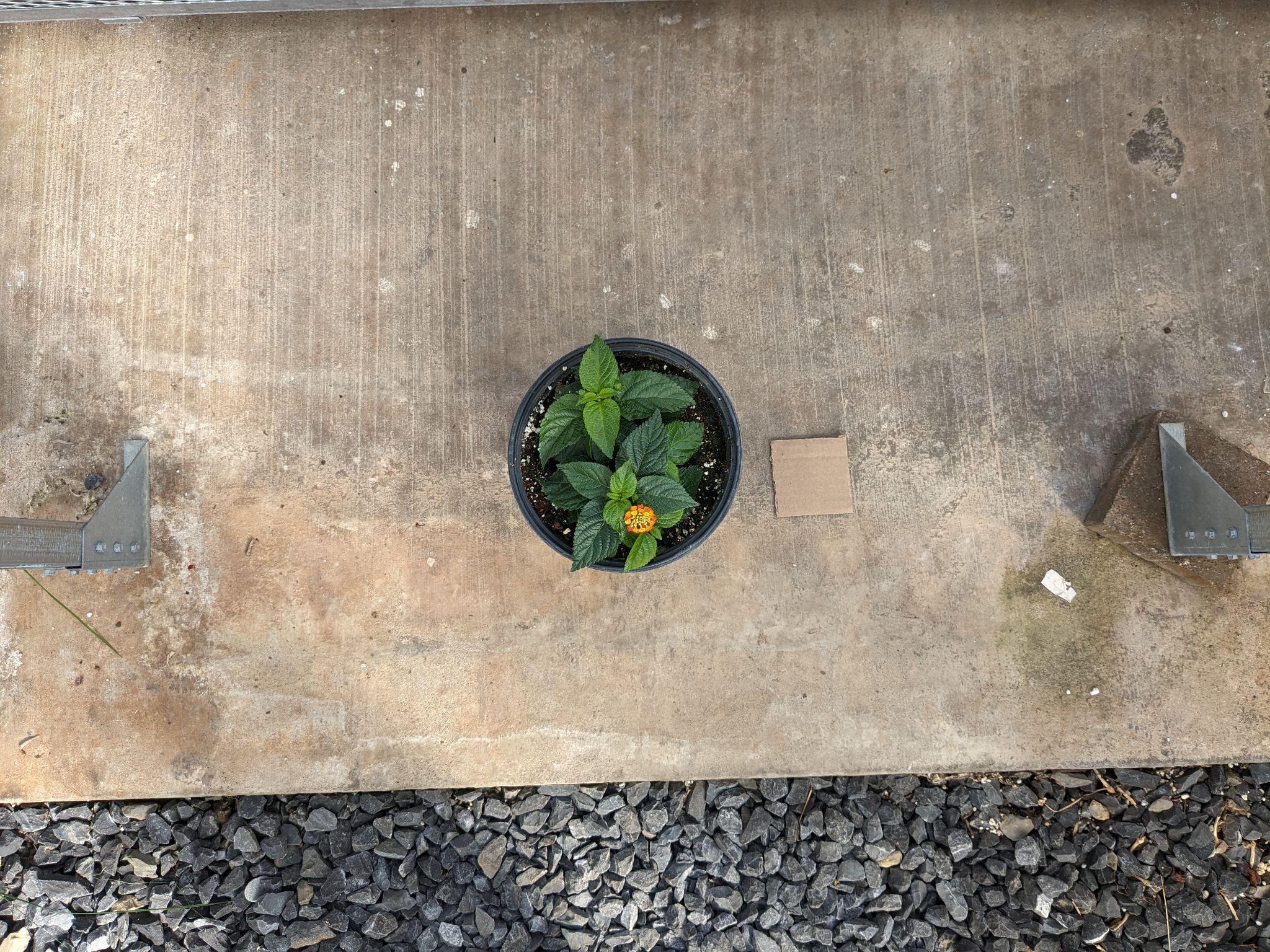 | 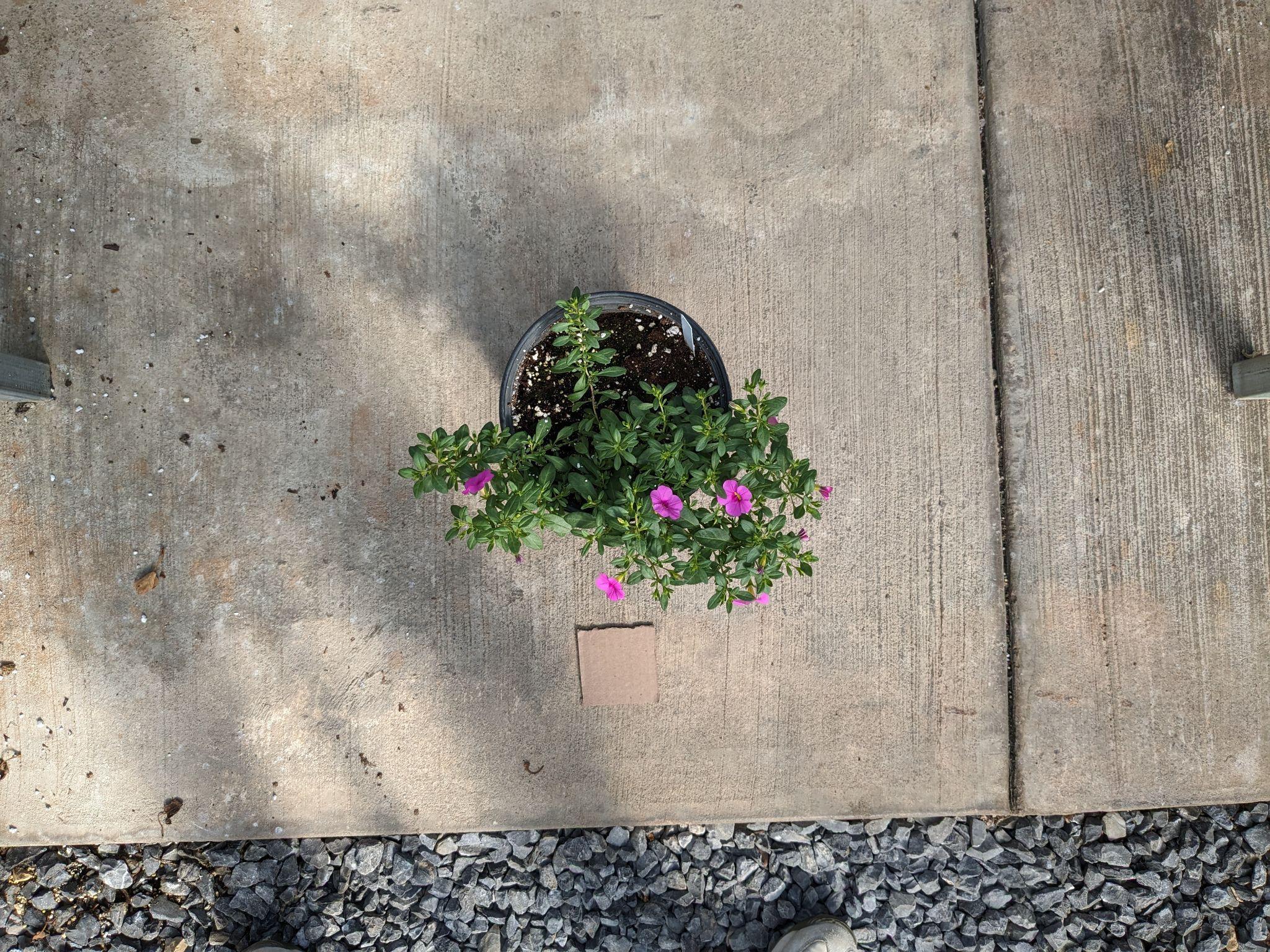 | 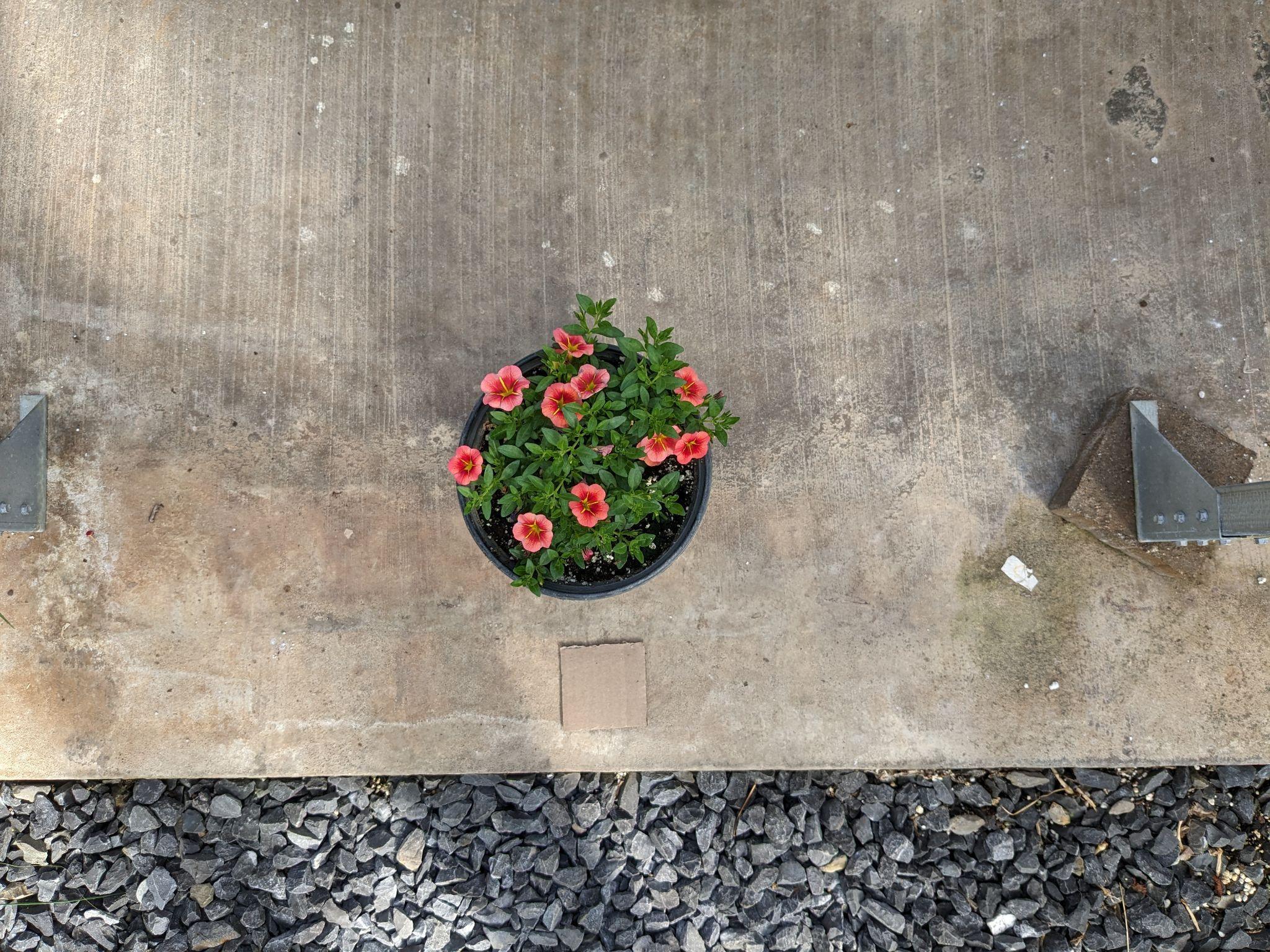 | 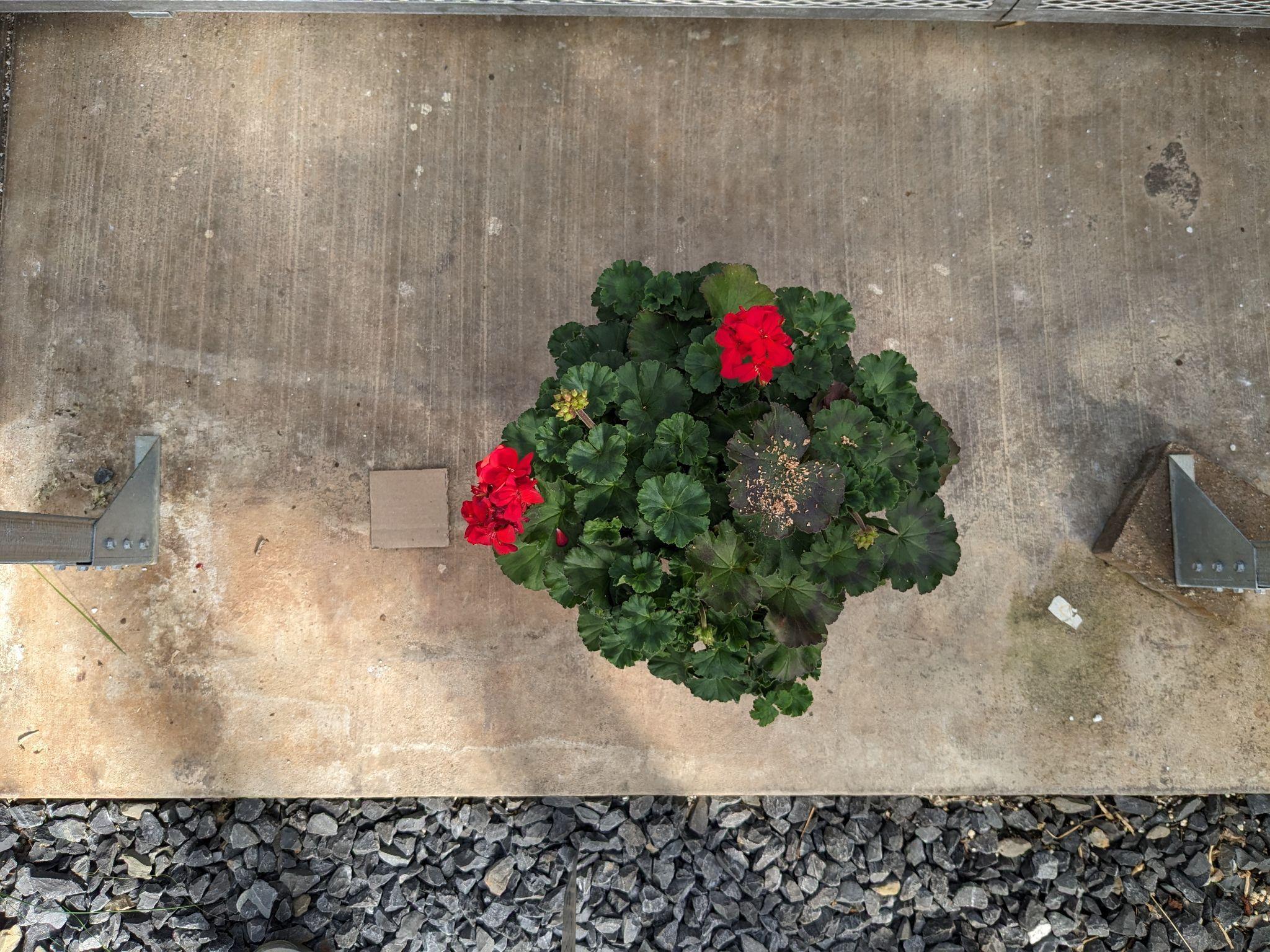 |  |
| 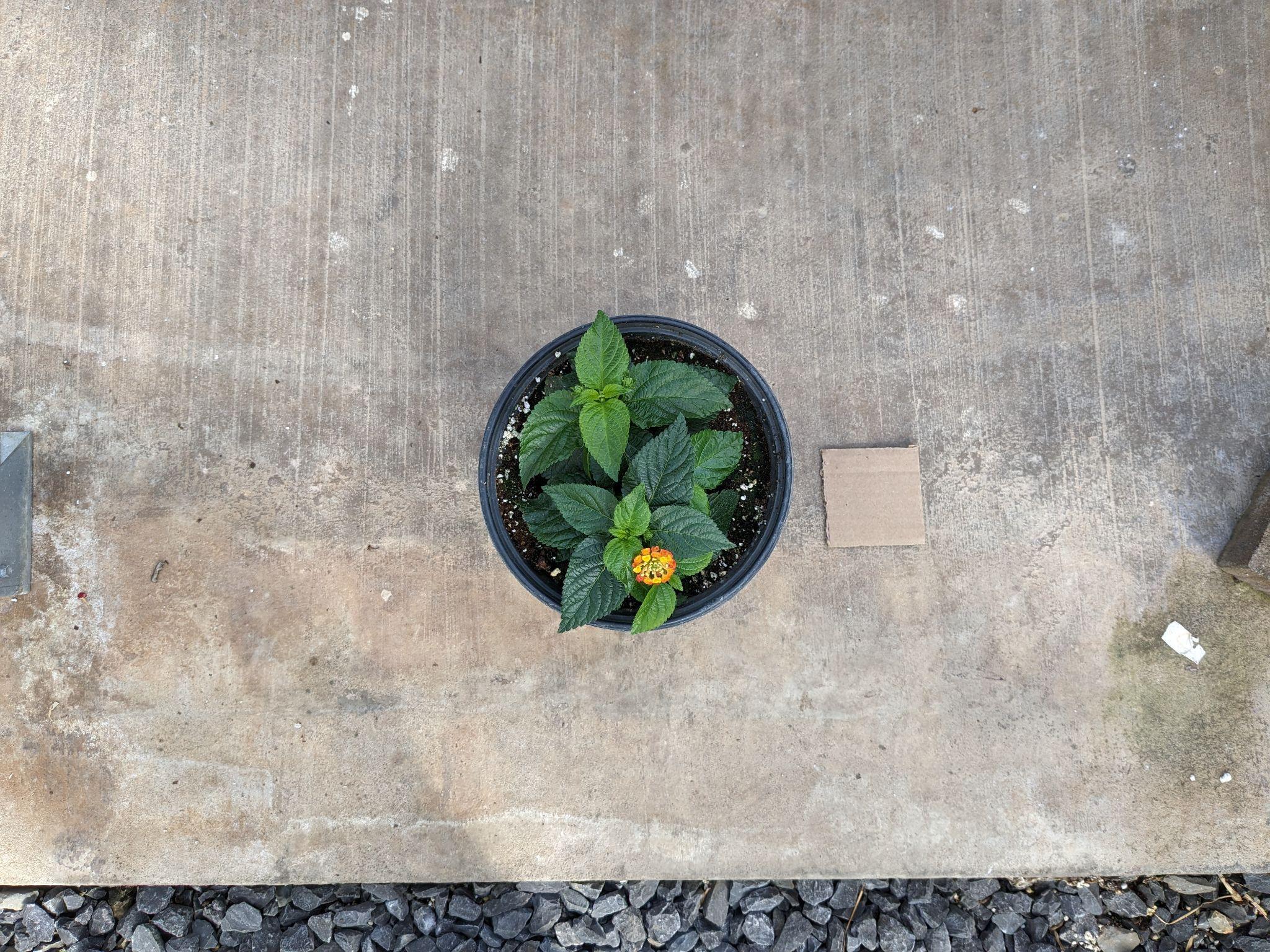 | 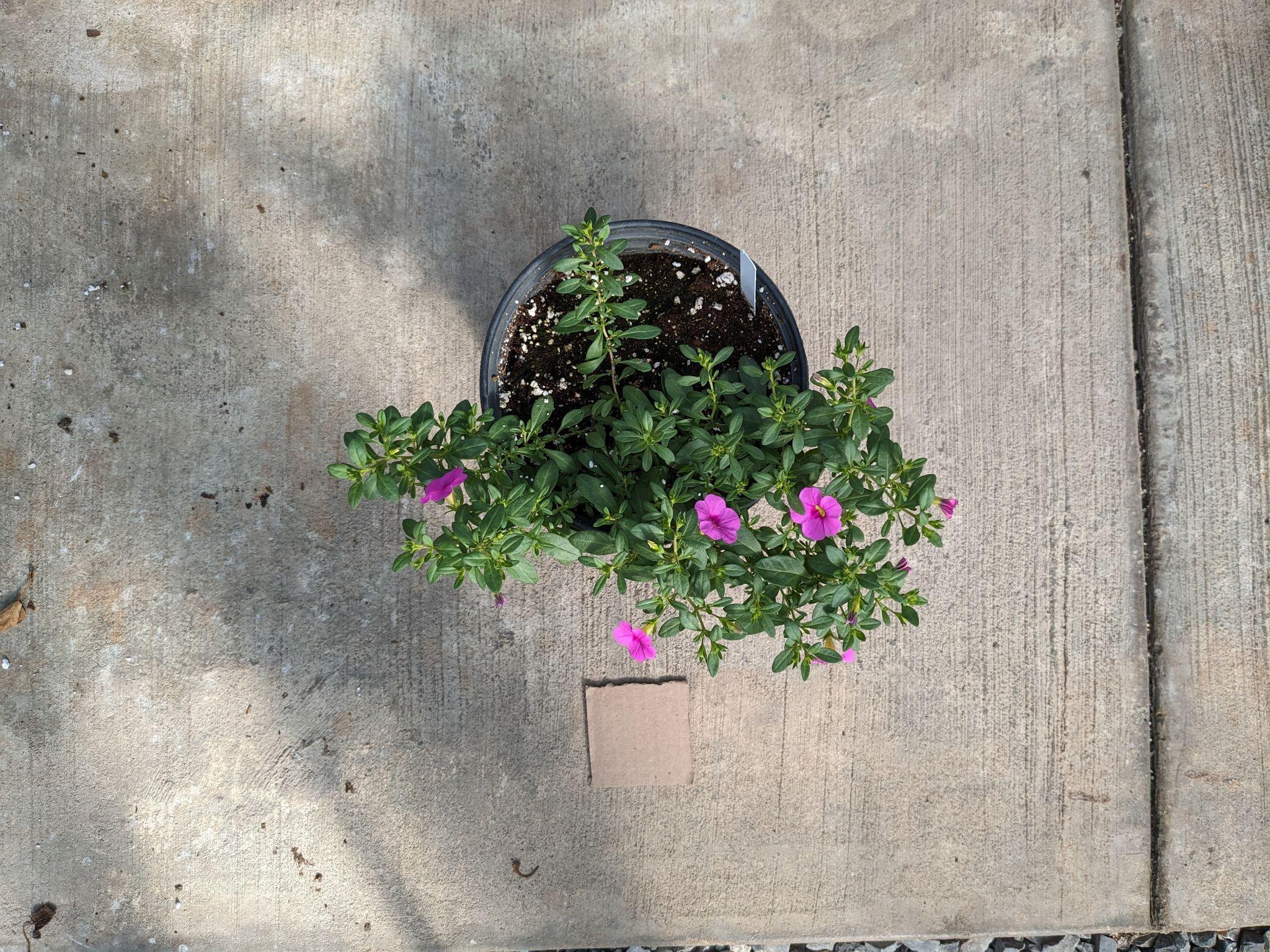 | 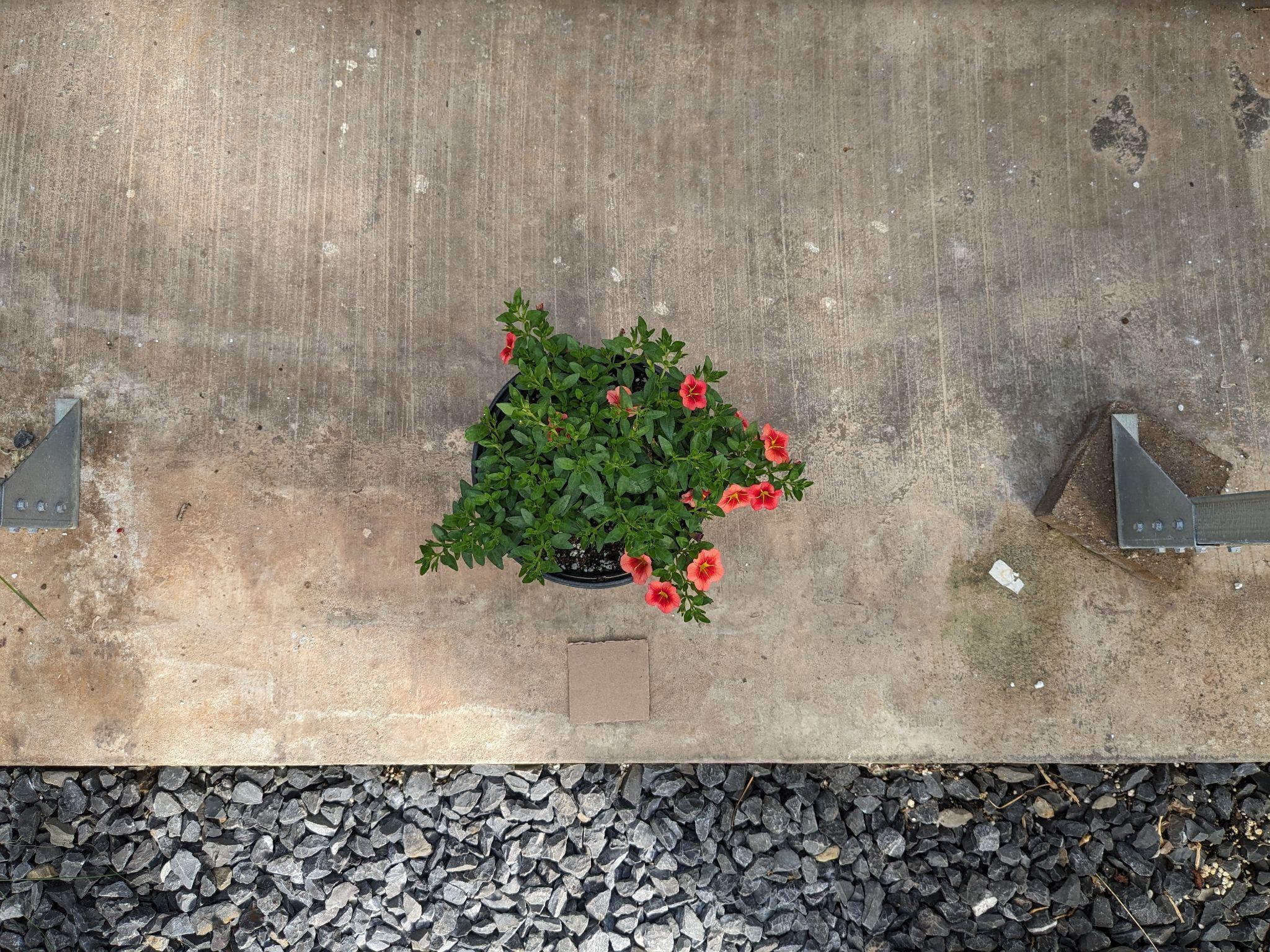 | 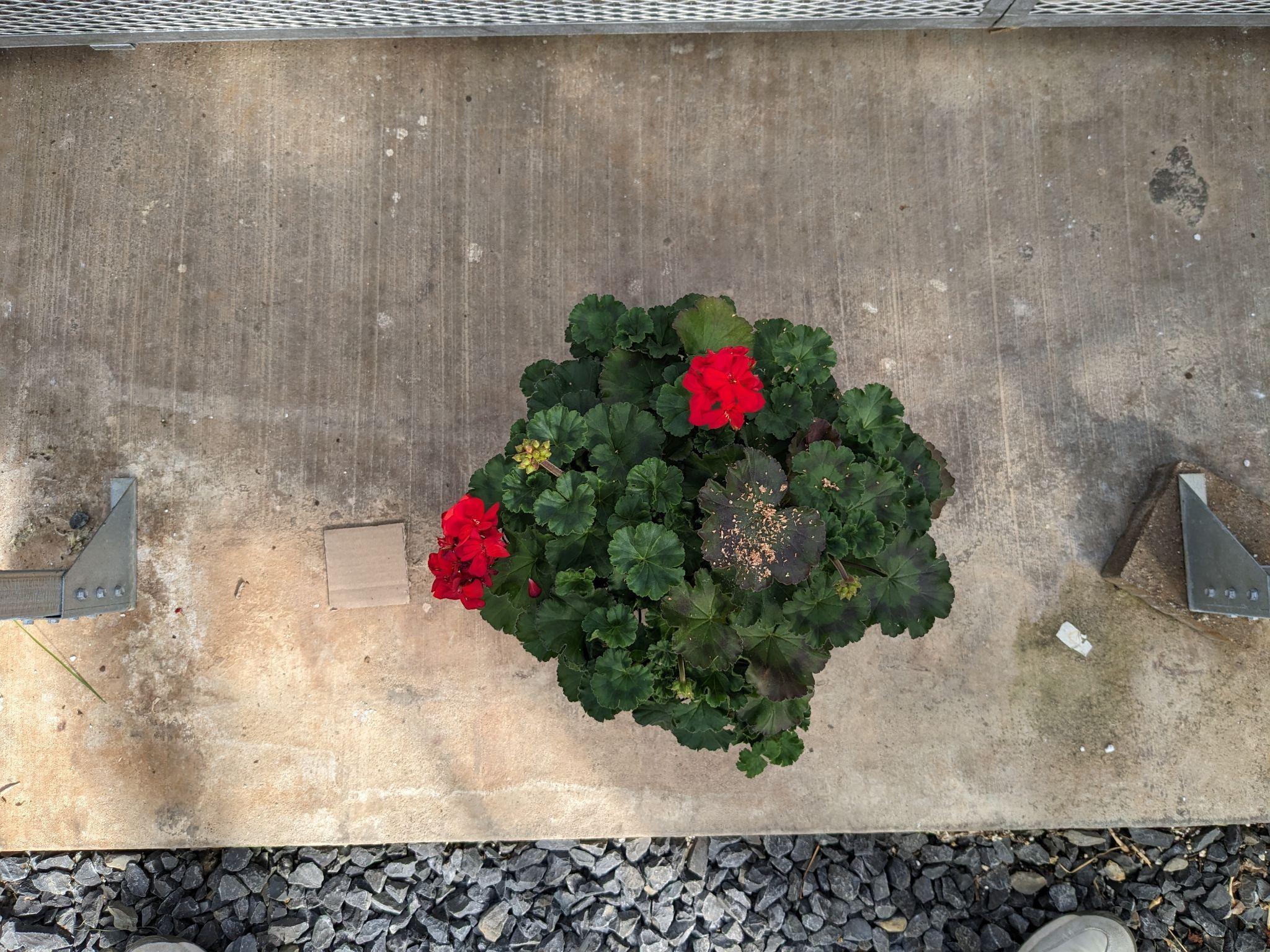 |  |
| 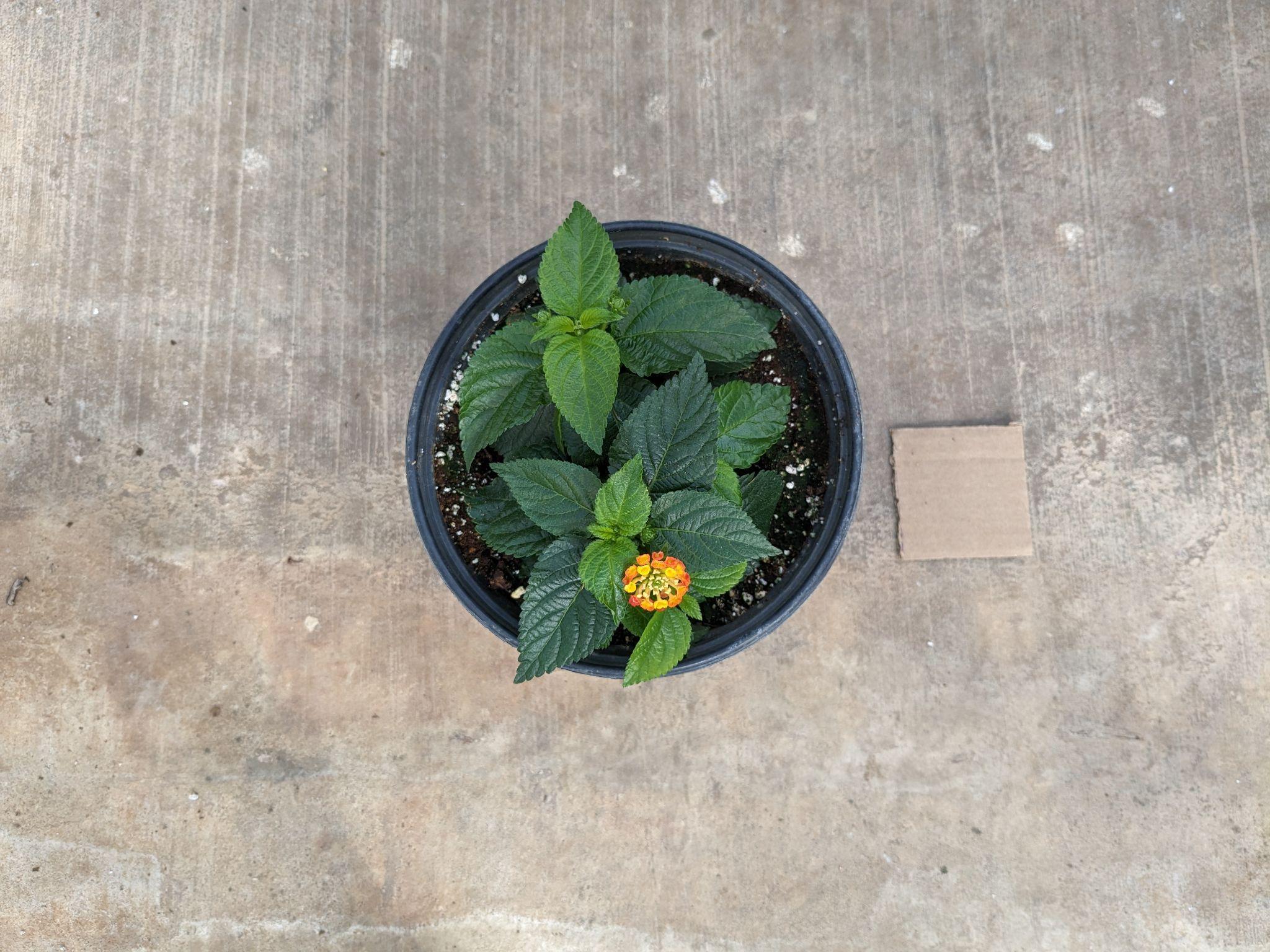 | 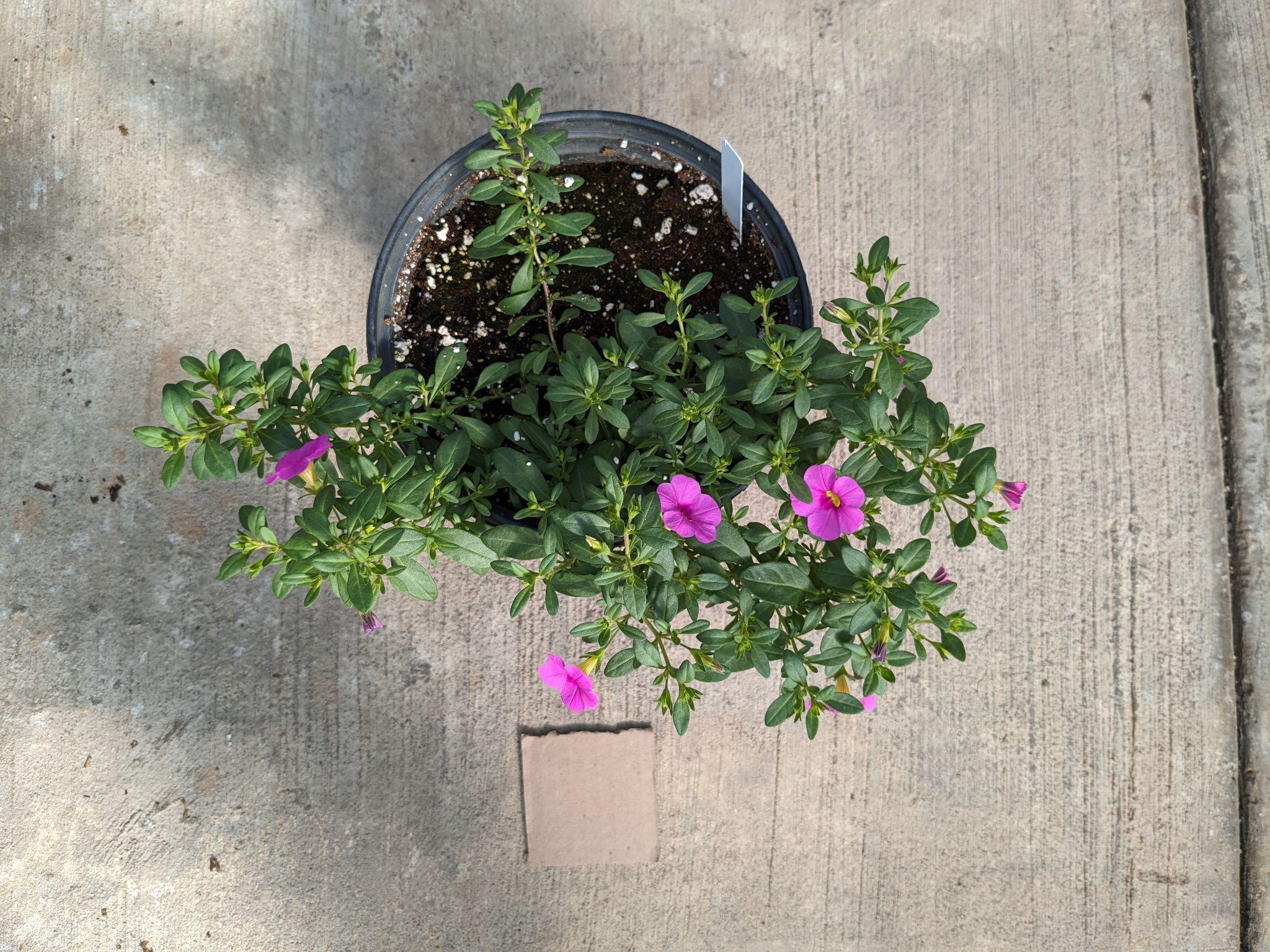 | 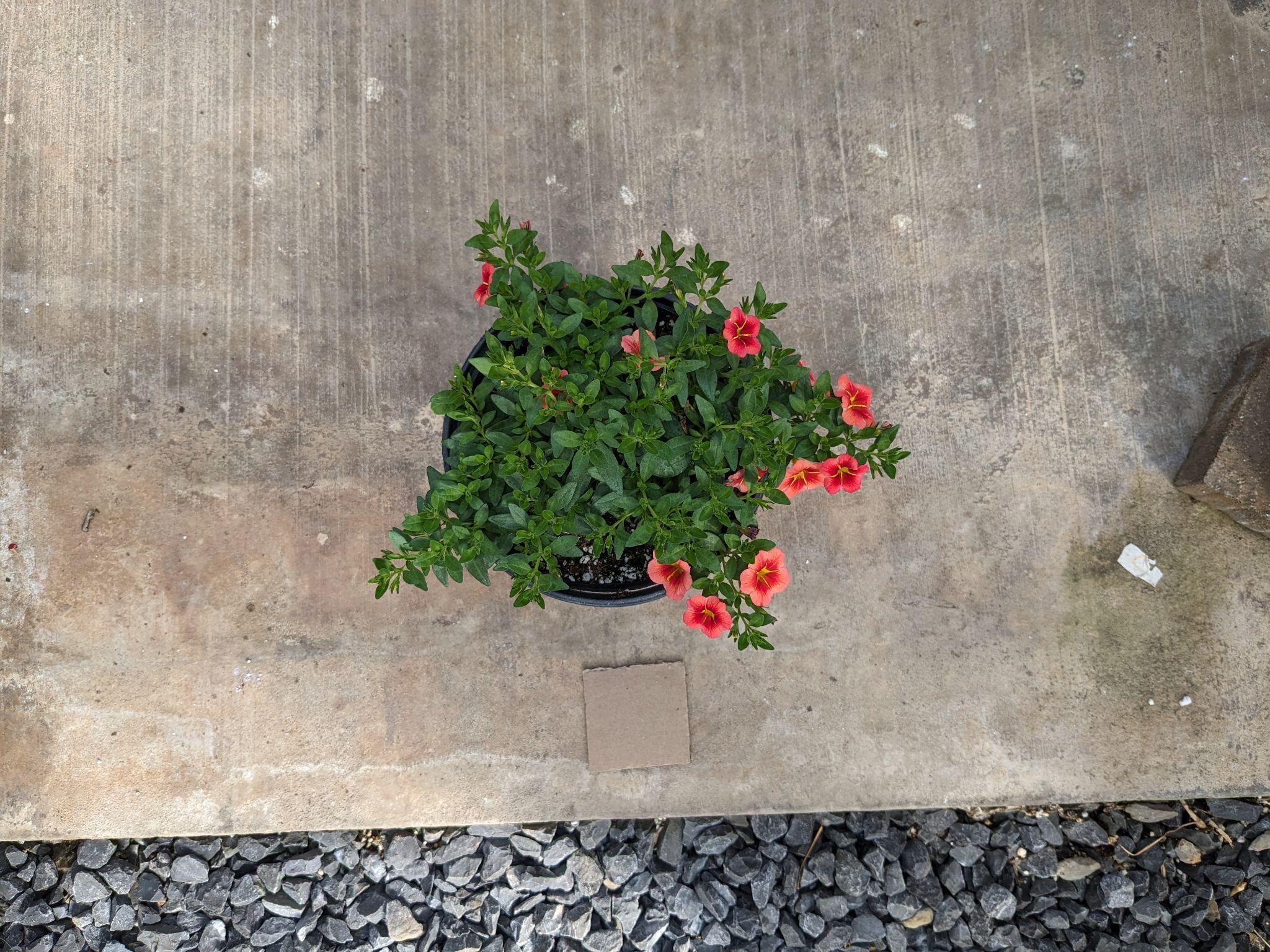 | 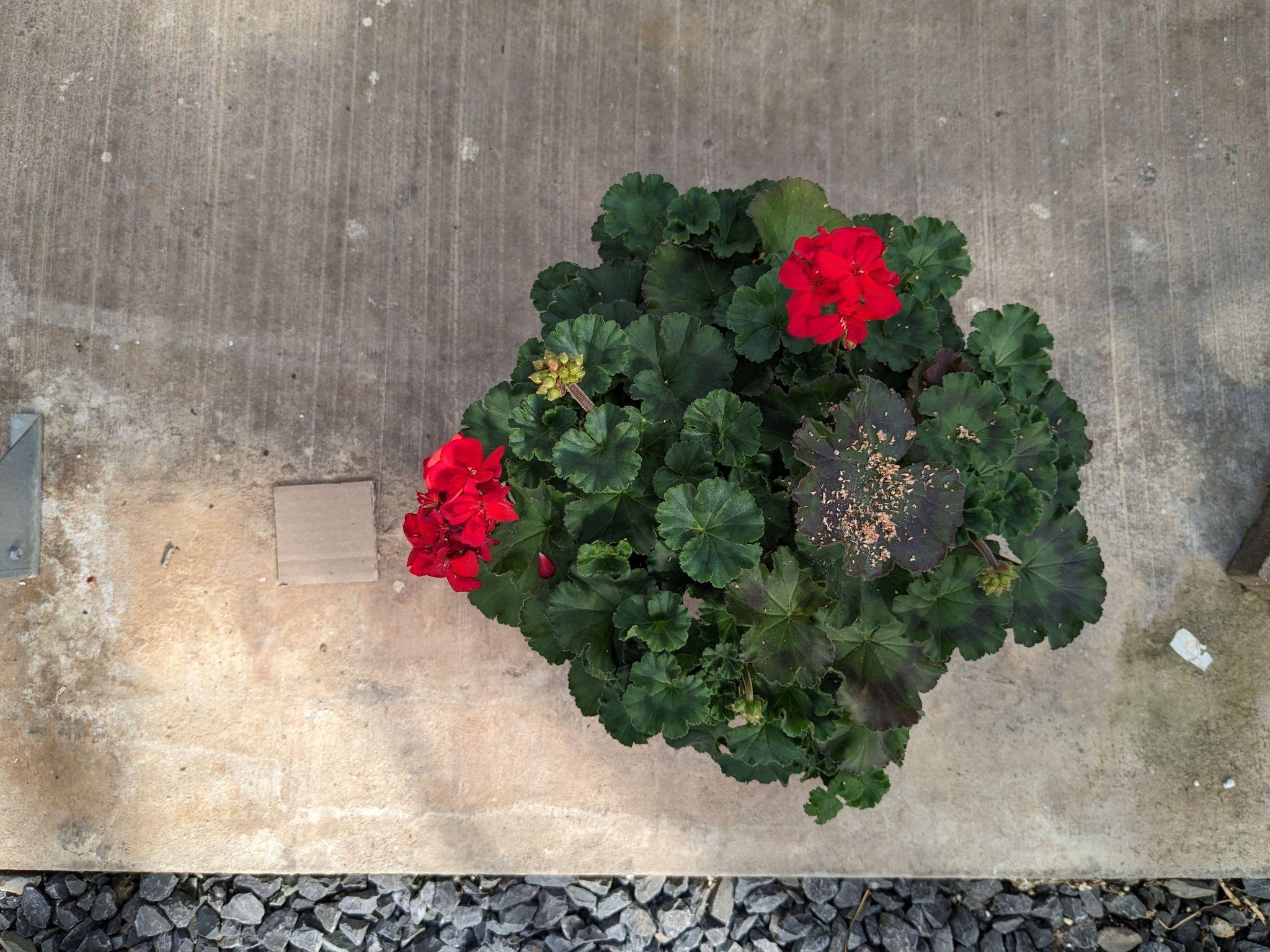 |  |
| 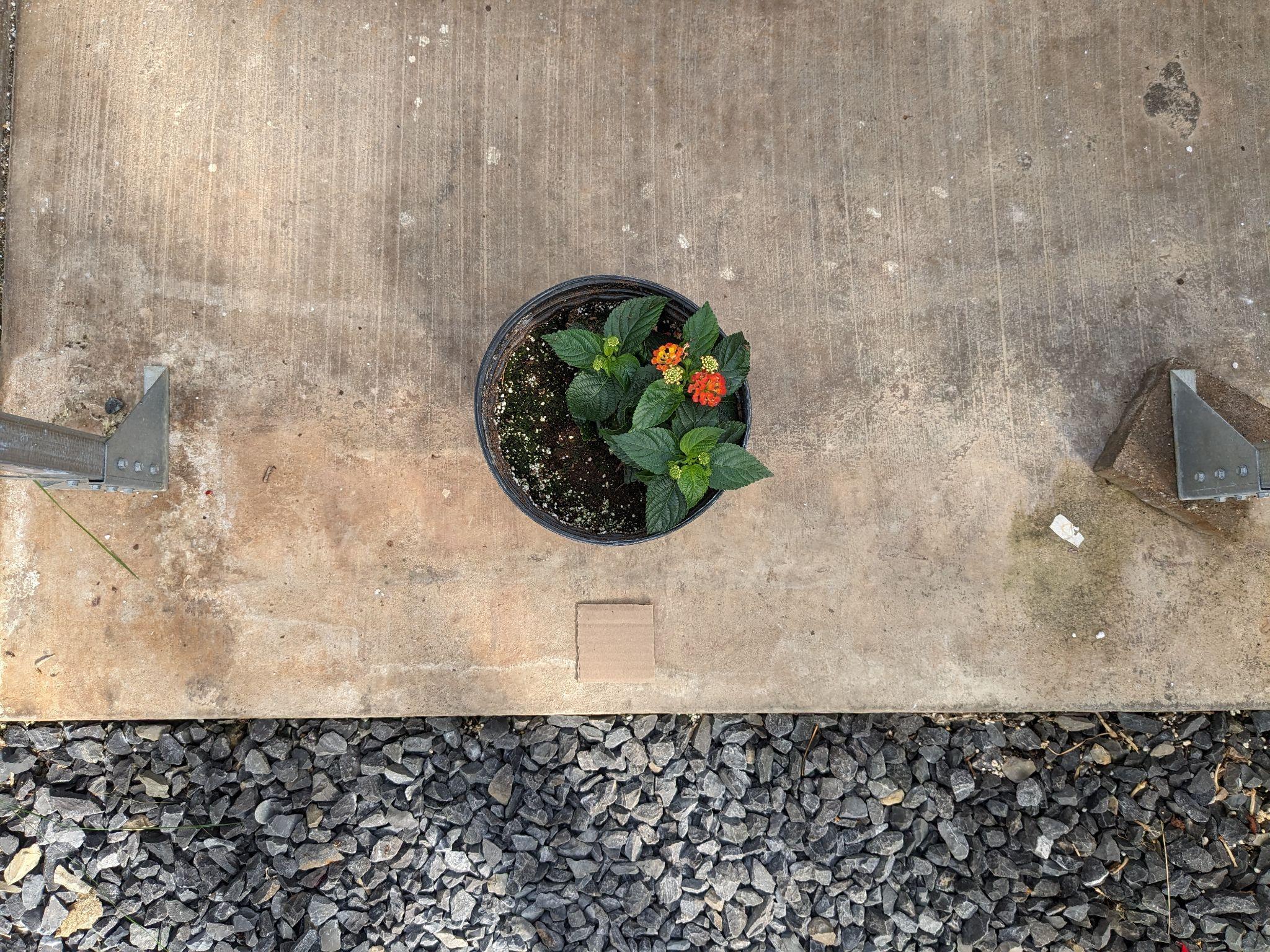 | 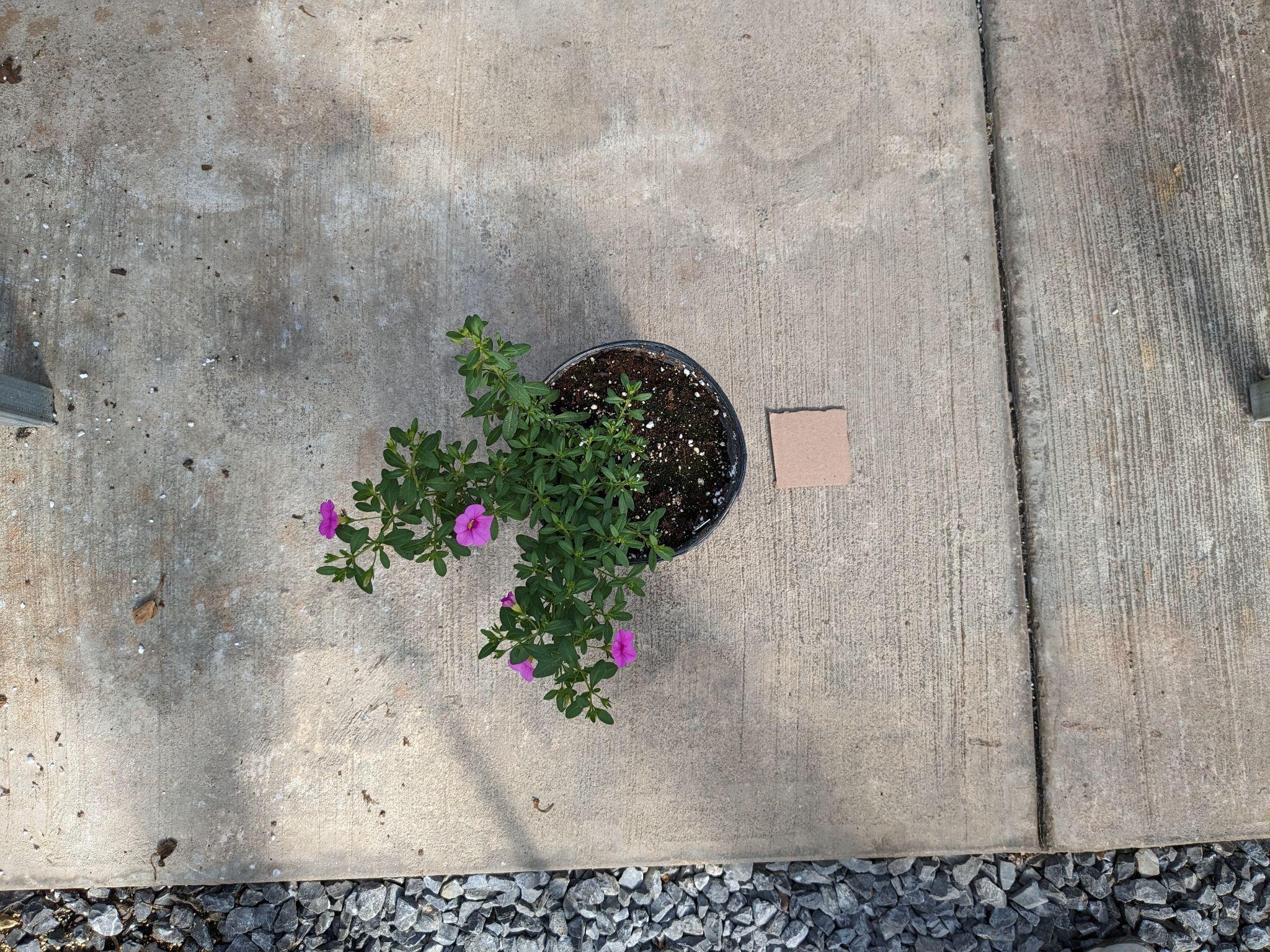 | 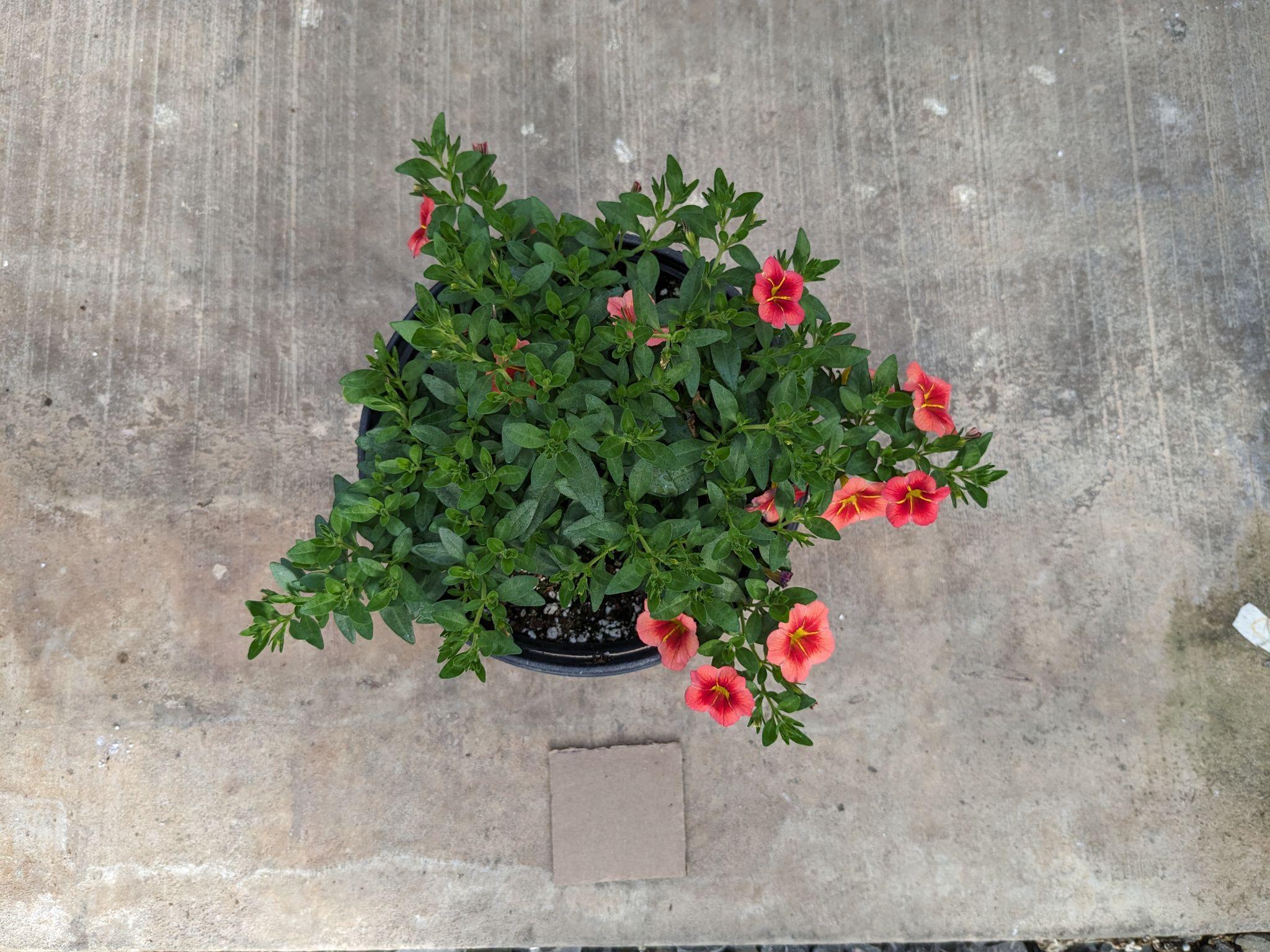 | 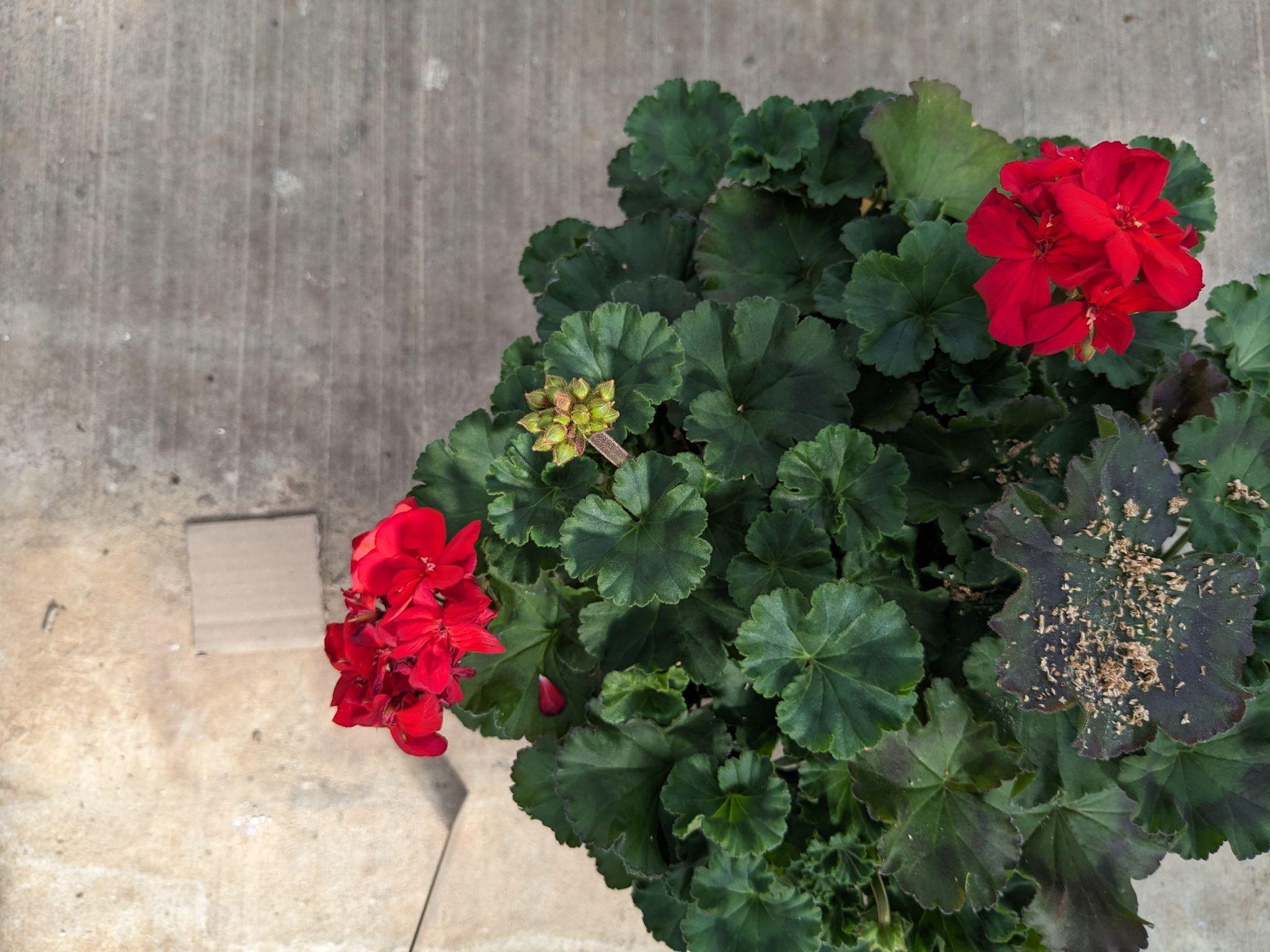 |  |
| 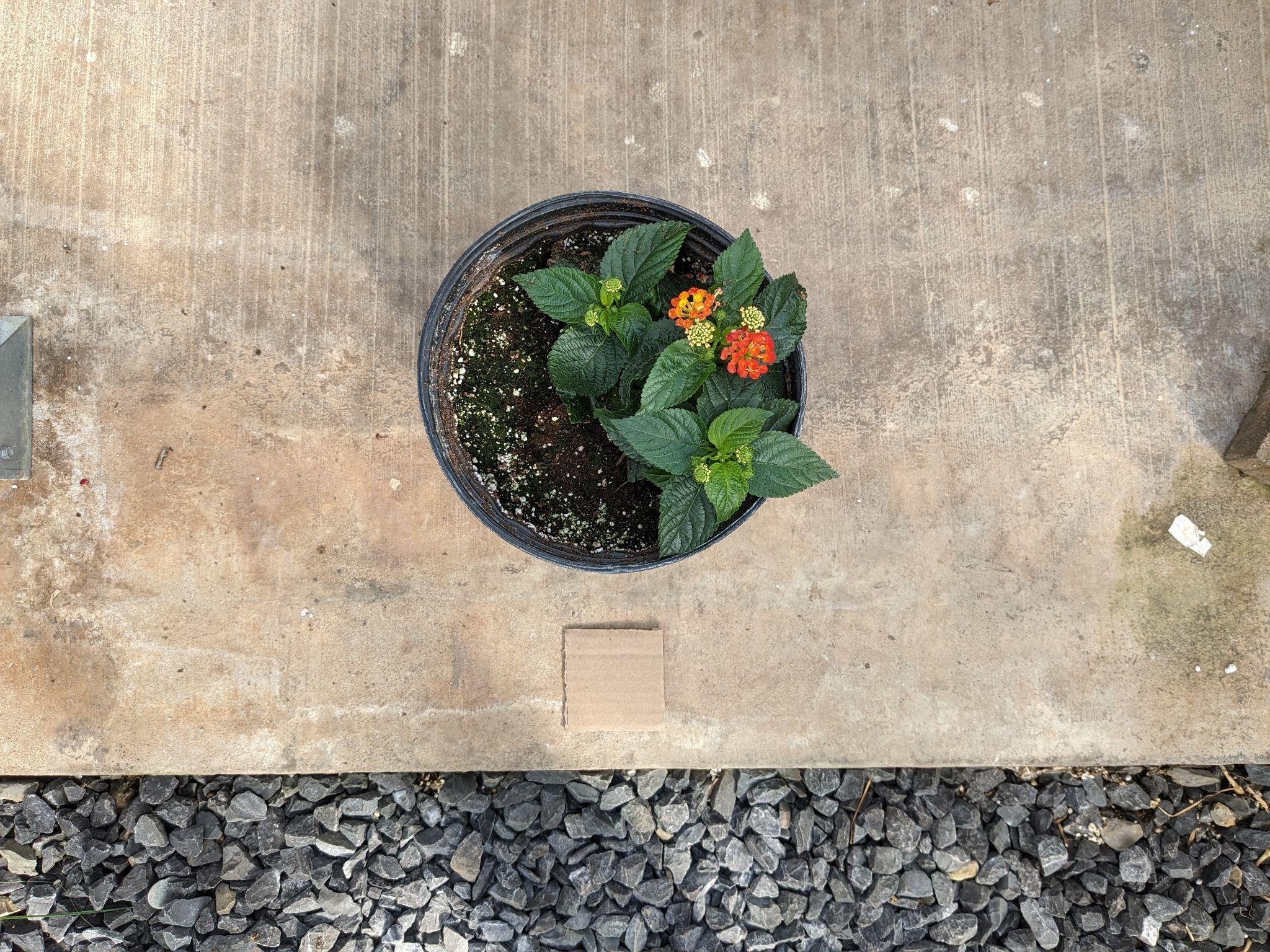 | 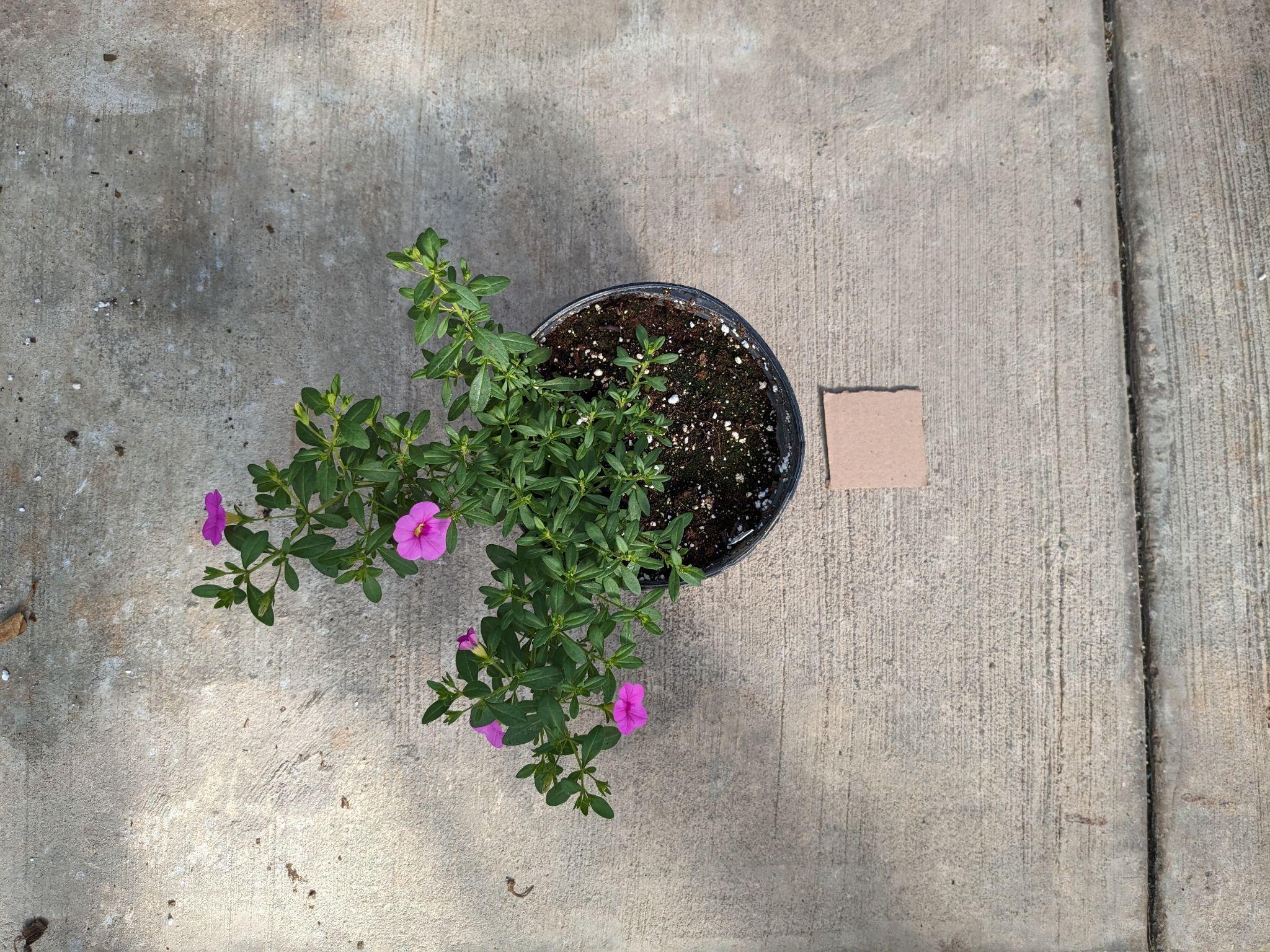 | 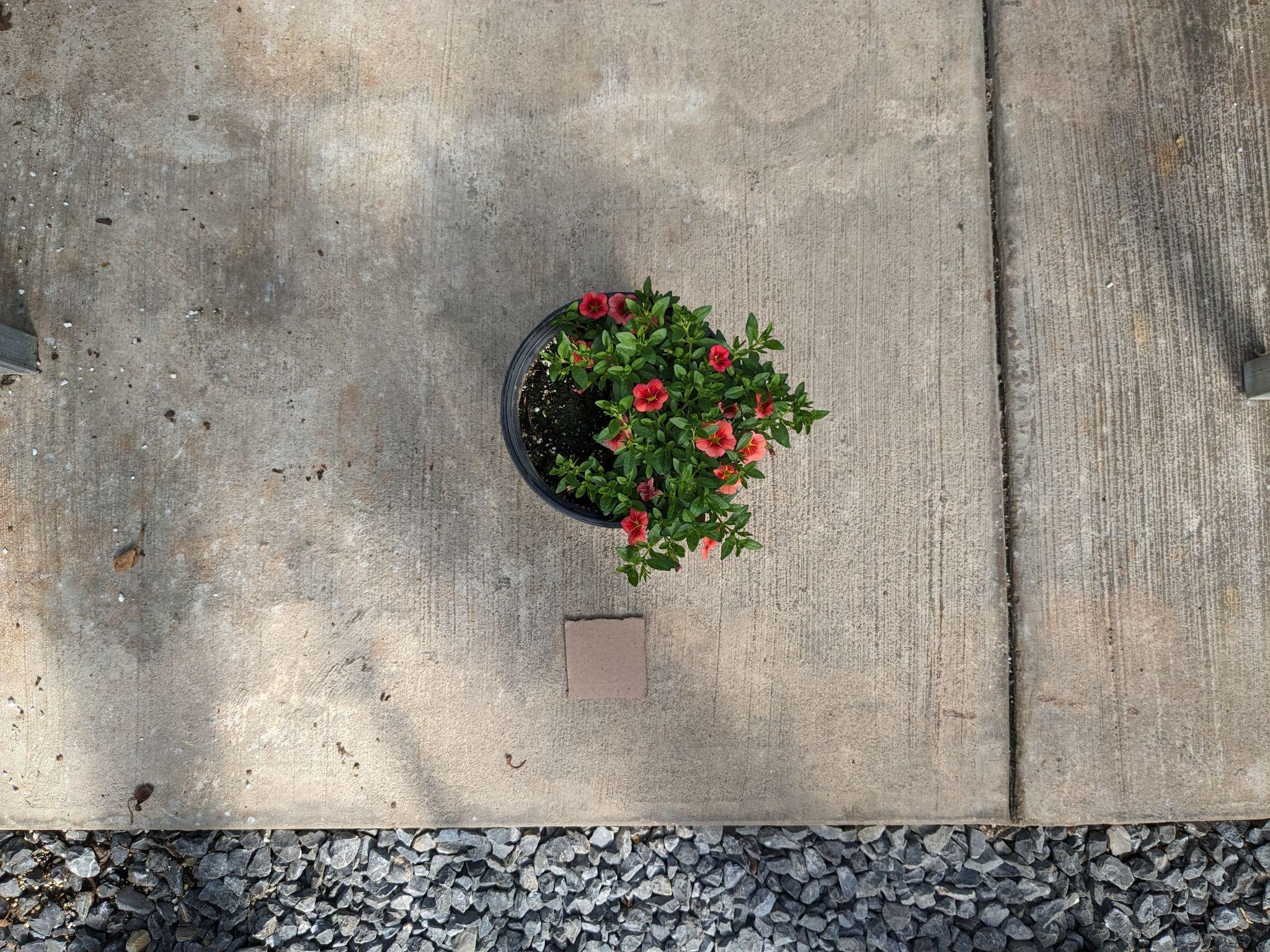 | 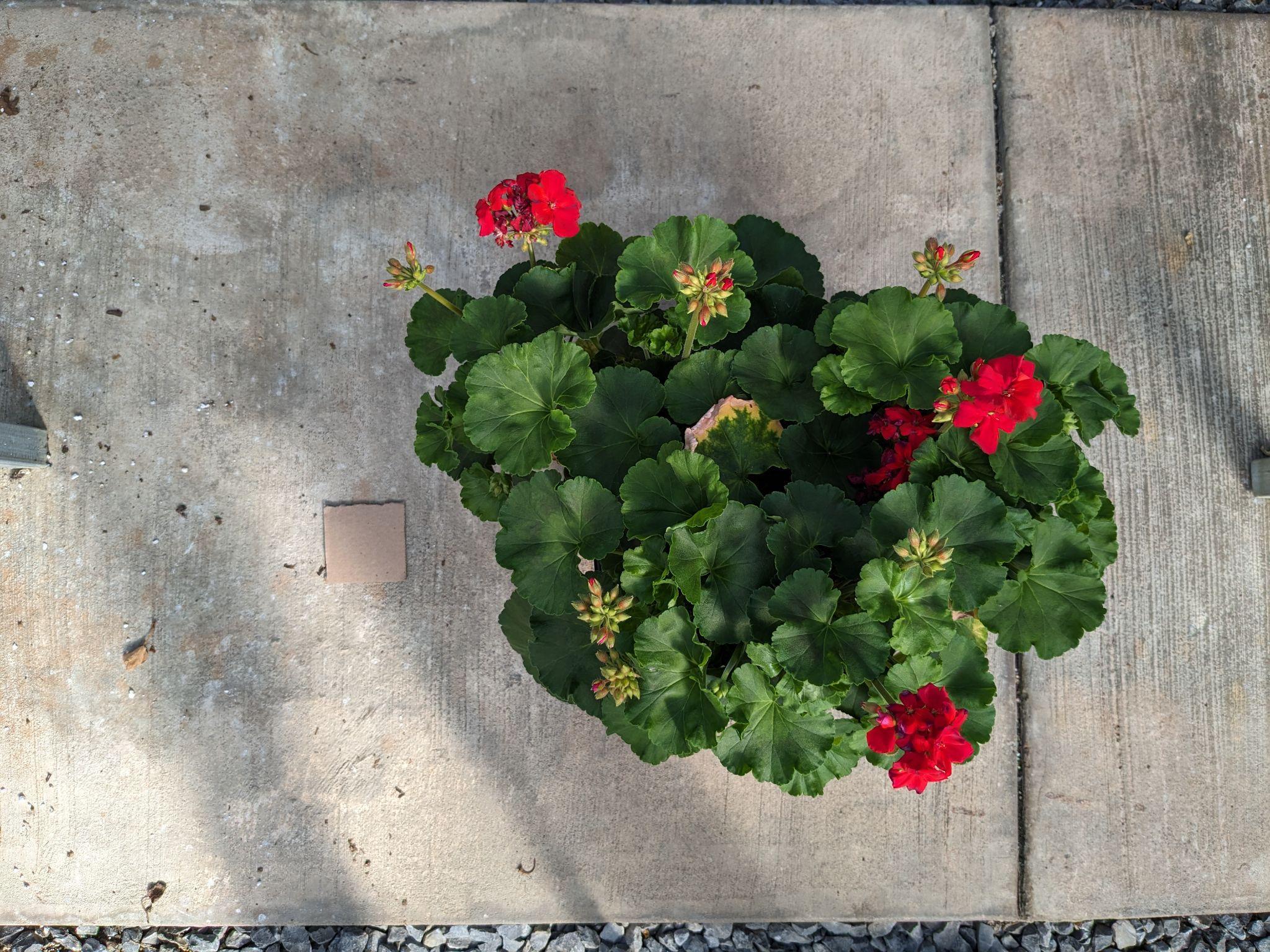 |  |
| 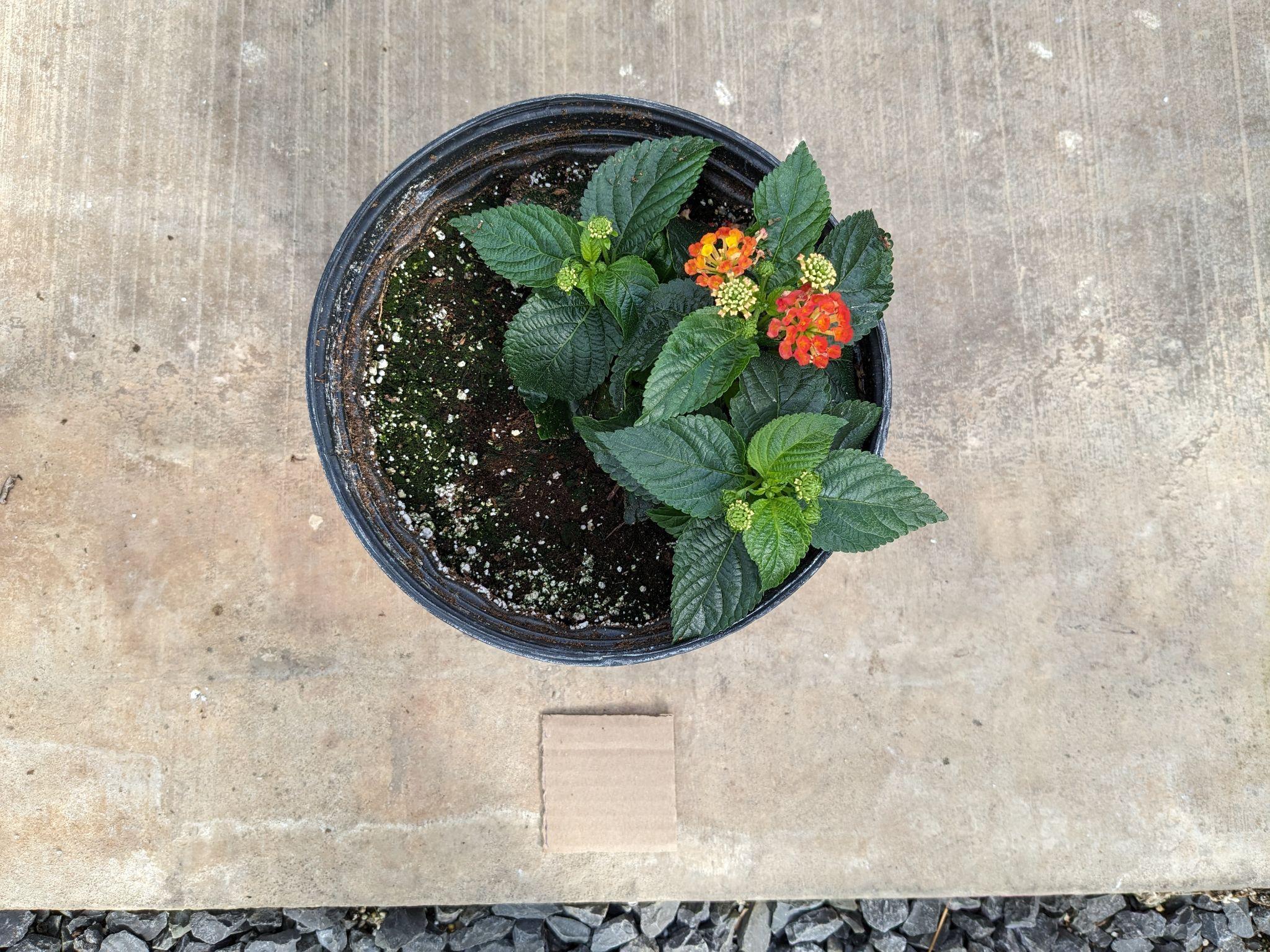 | 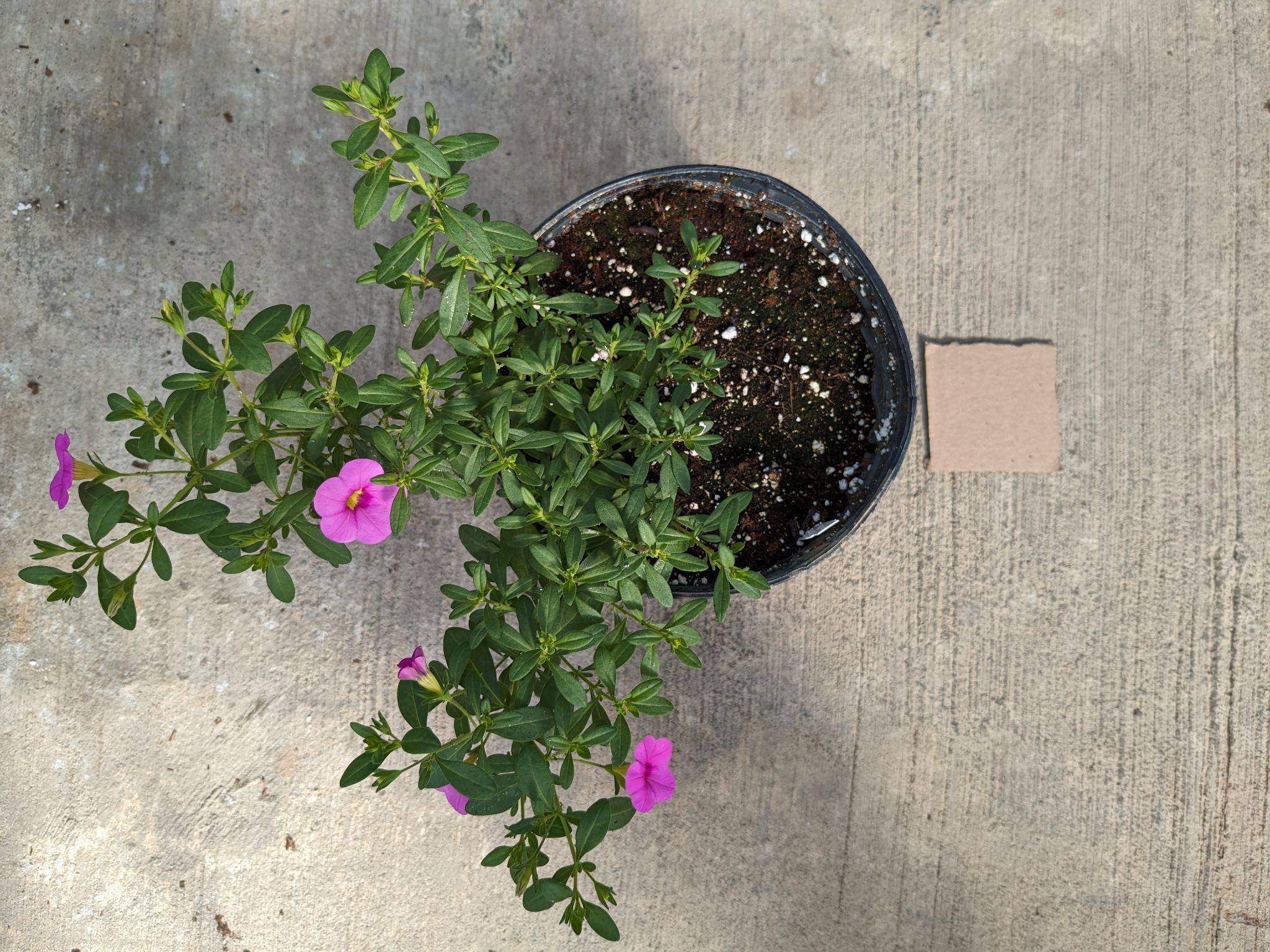 | 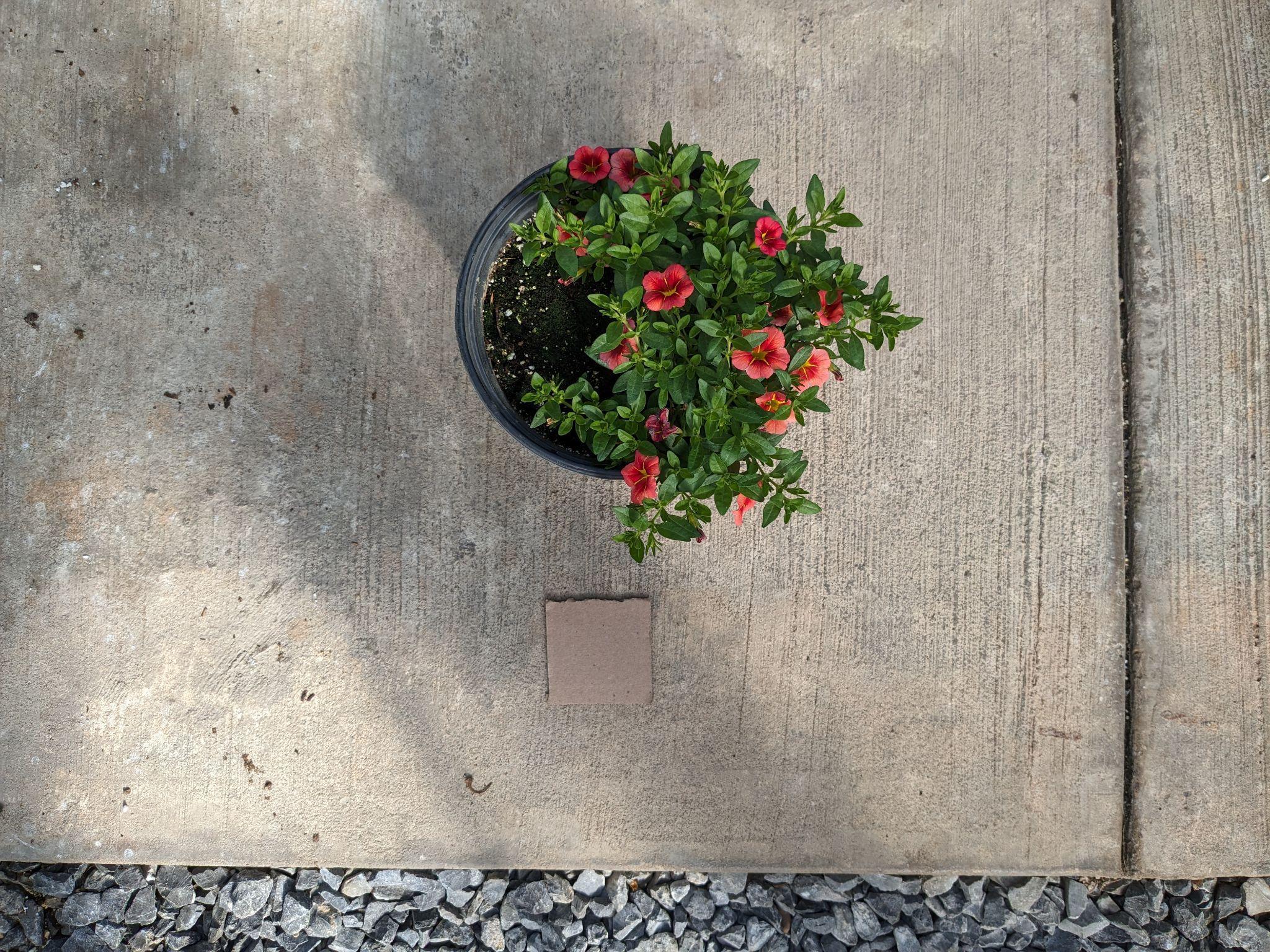 | 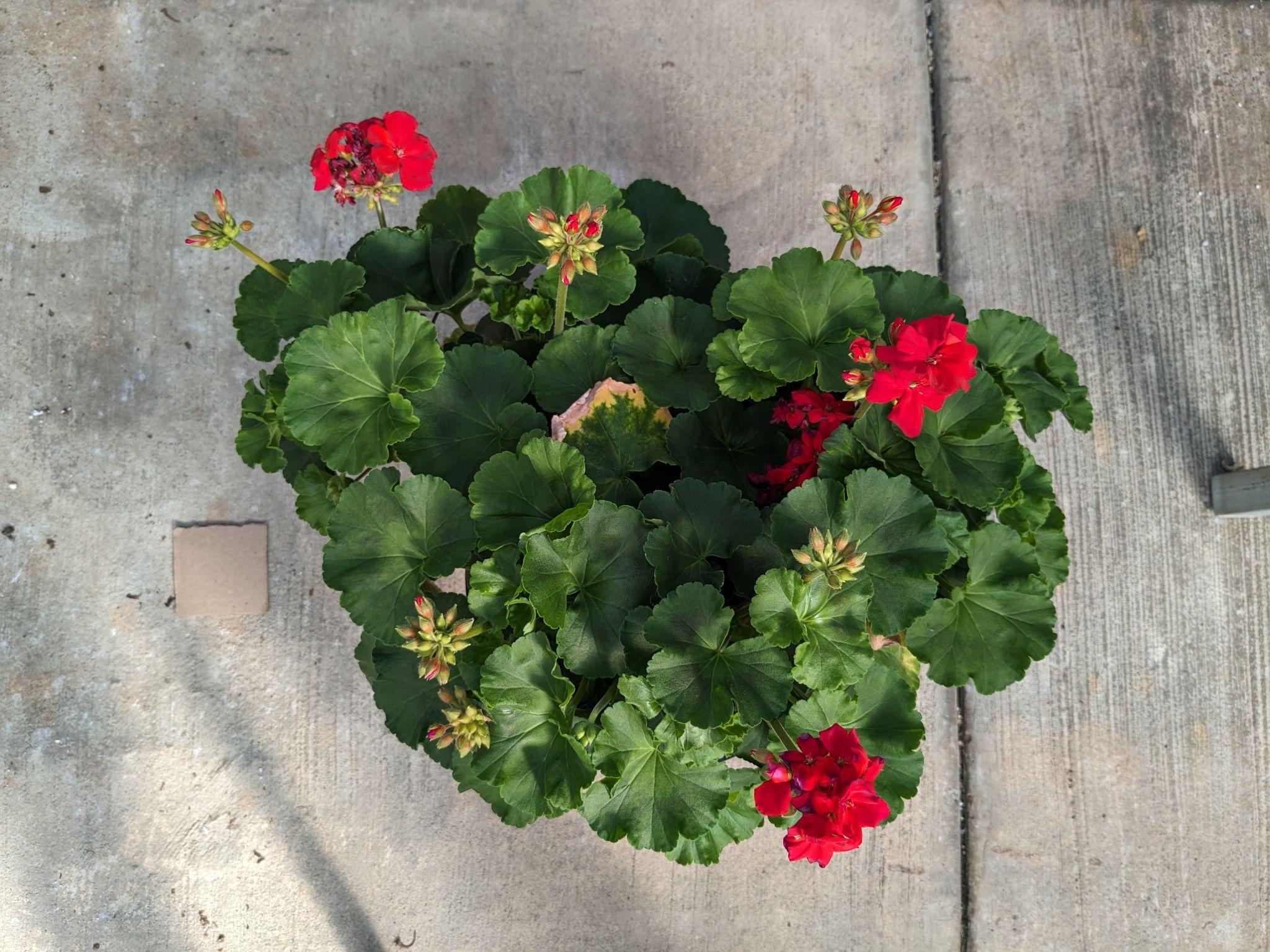 |  |
| 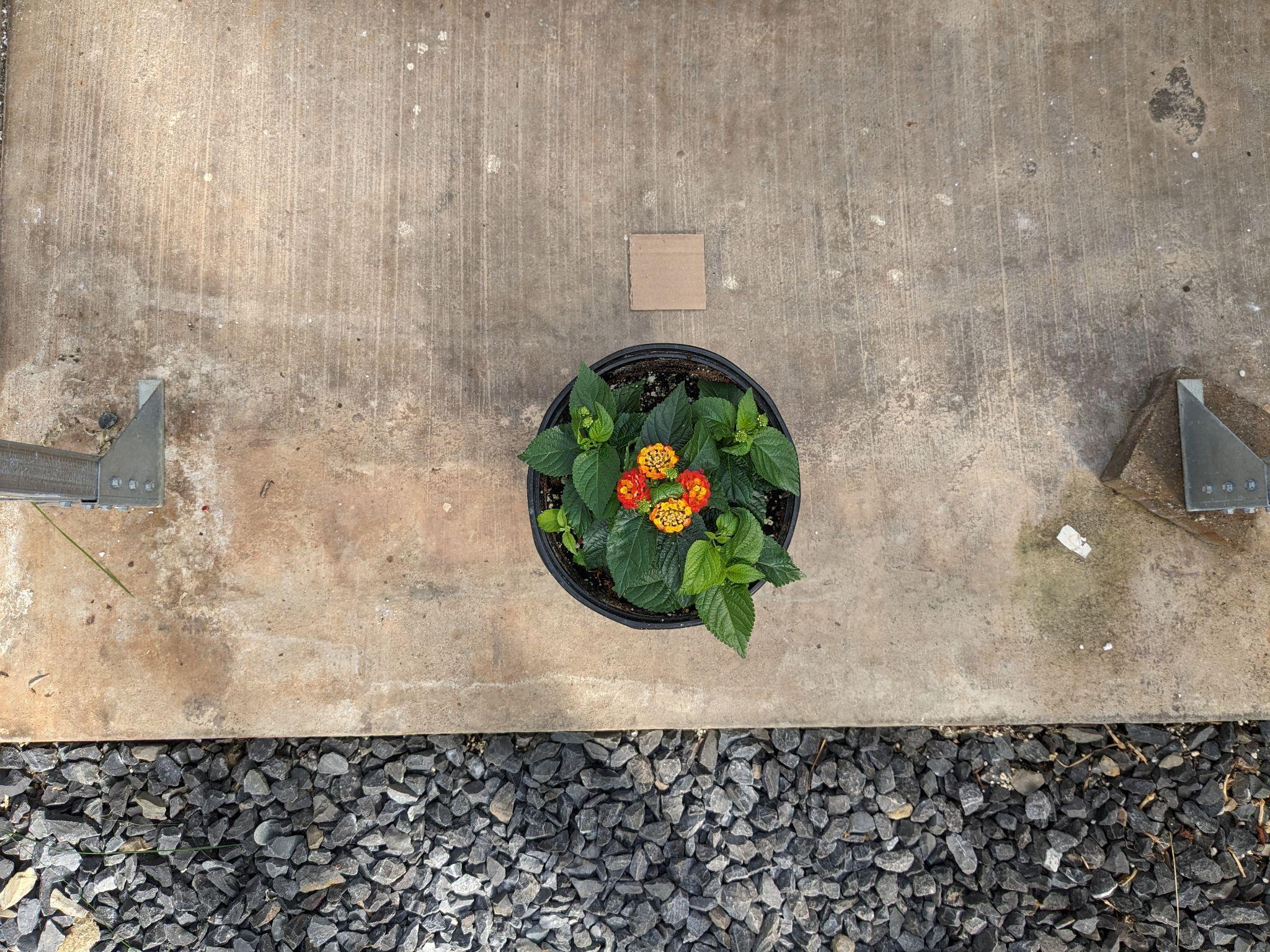 | 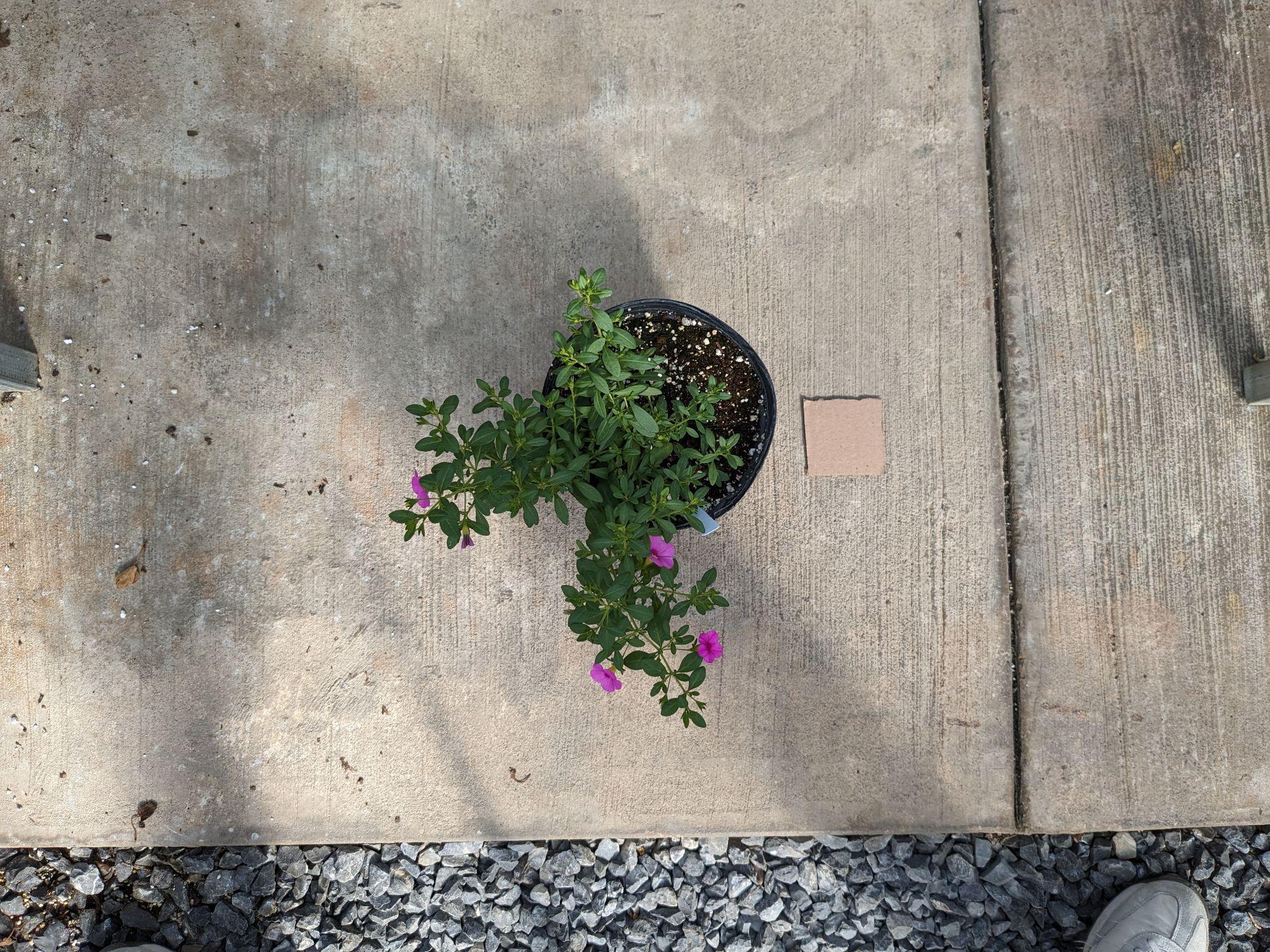 | 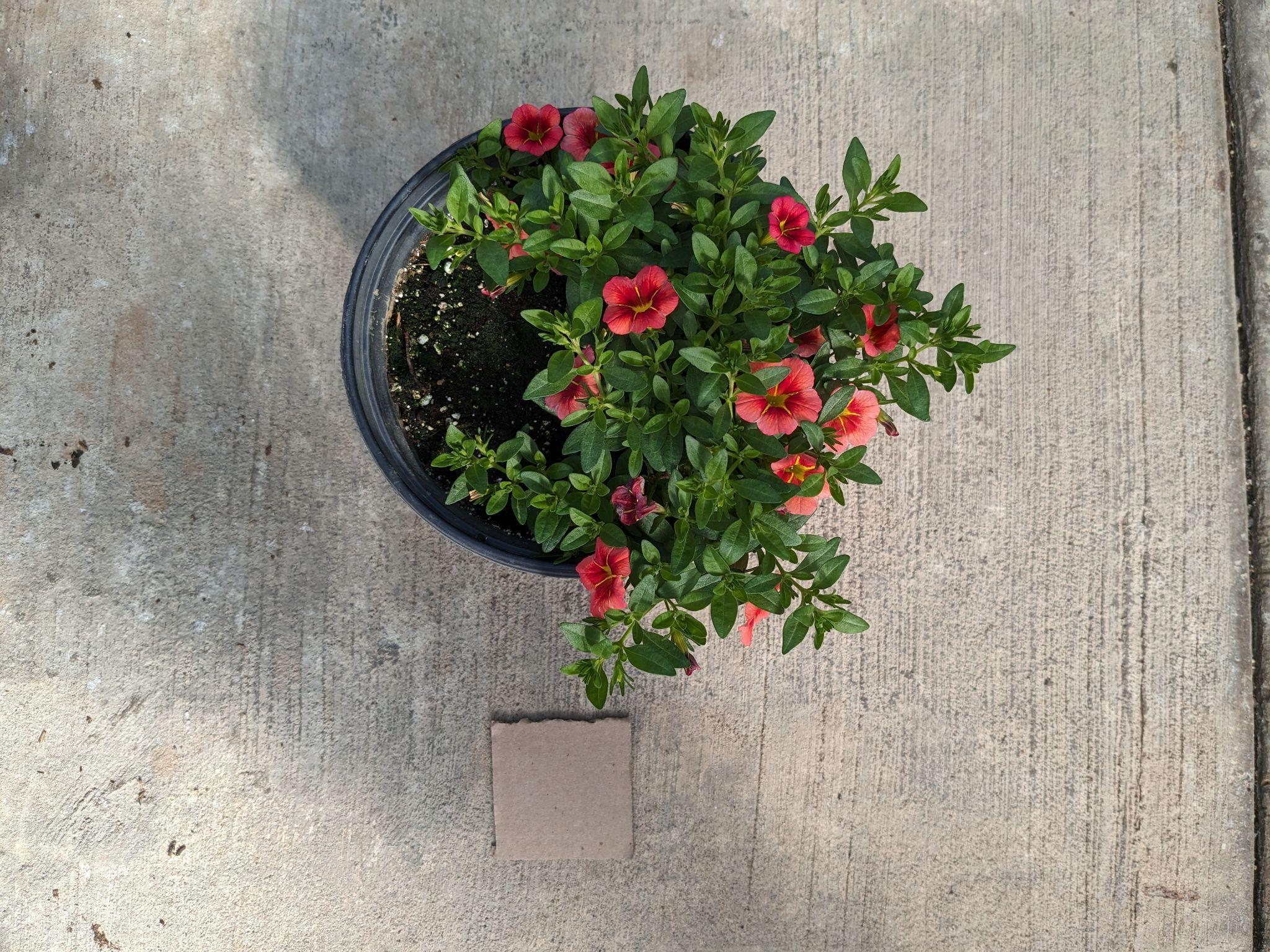 | 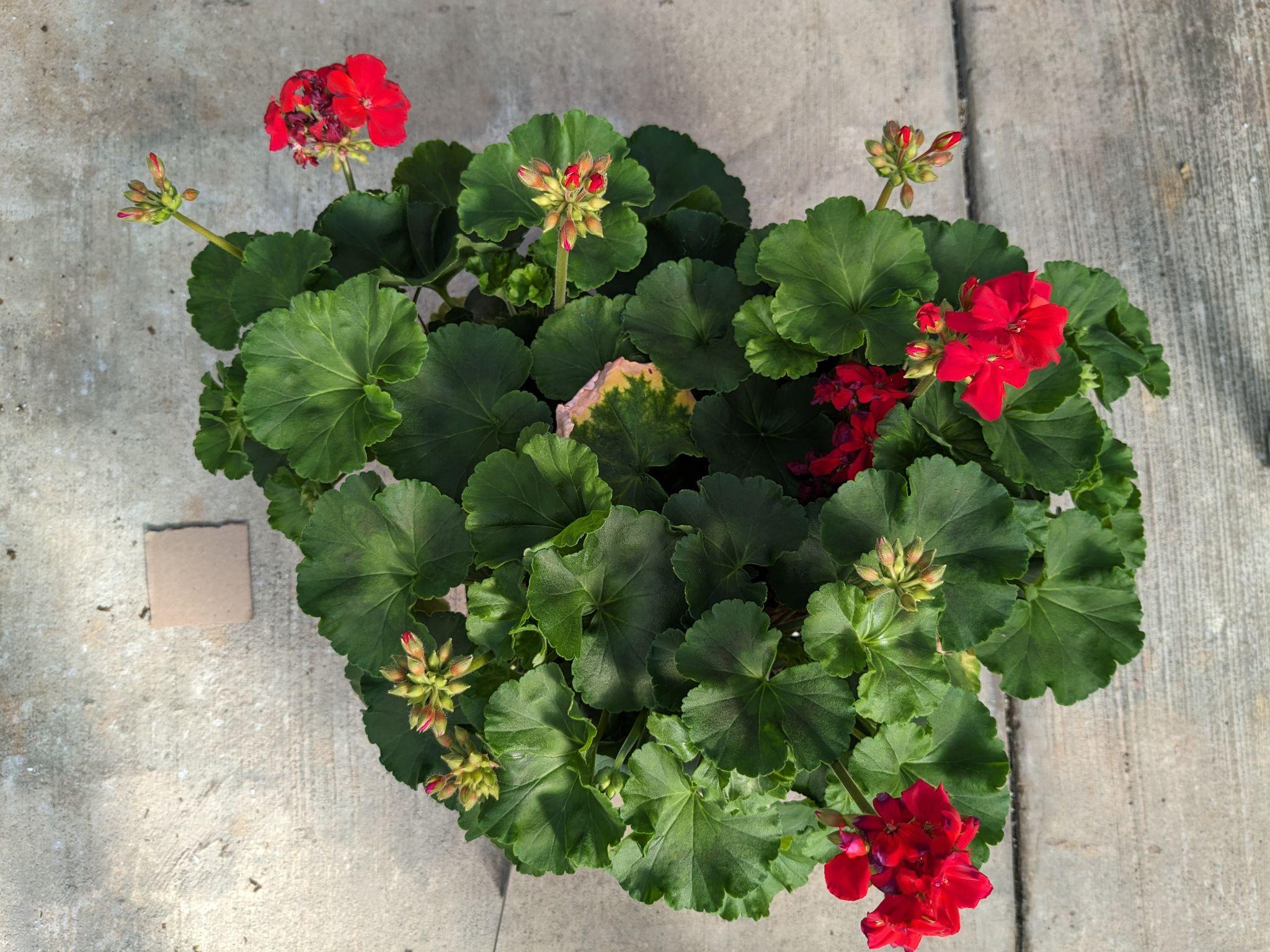 |  |
| 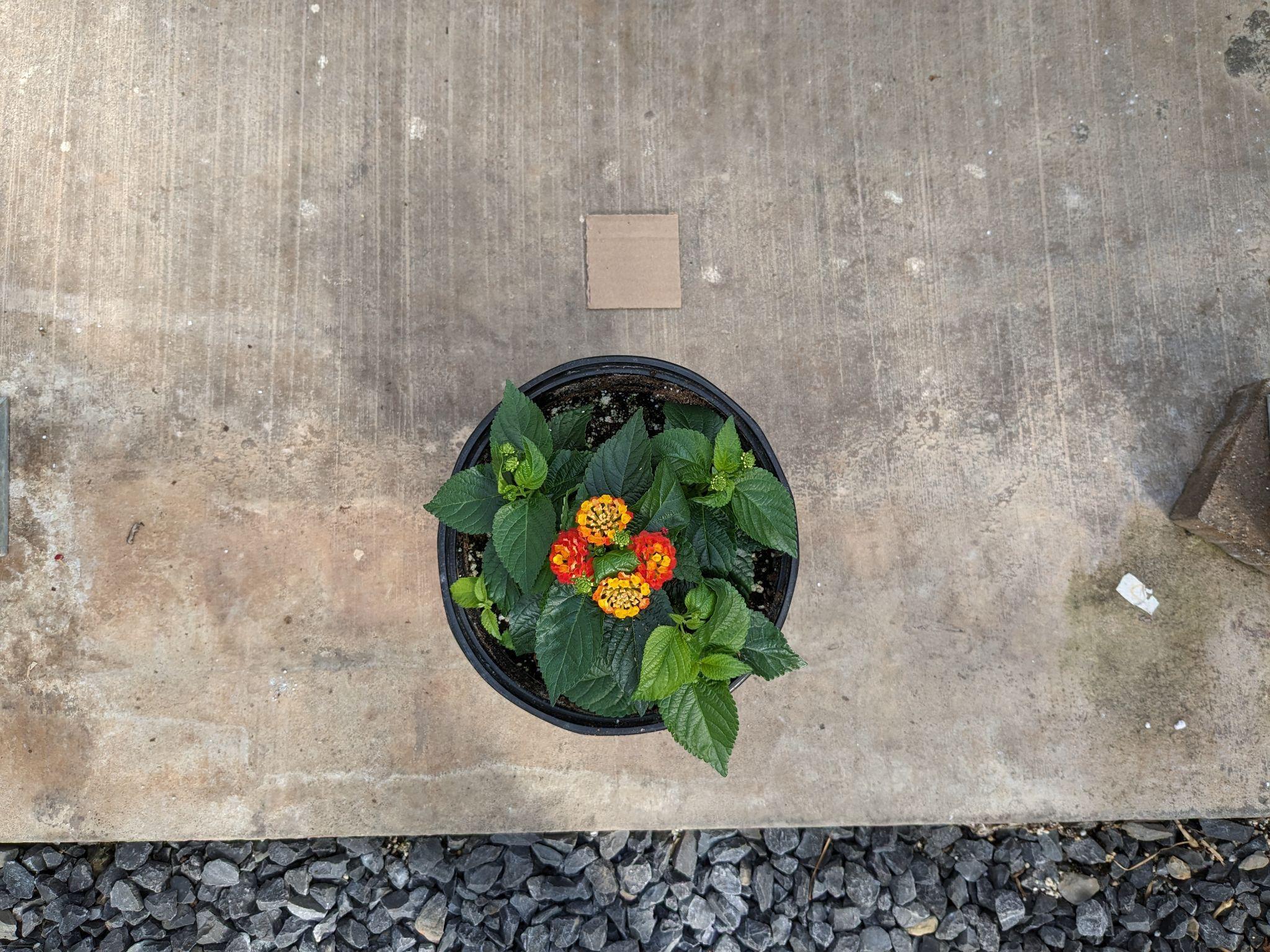 | 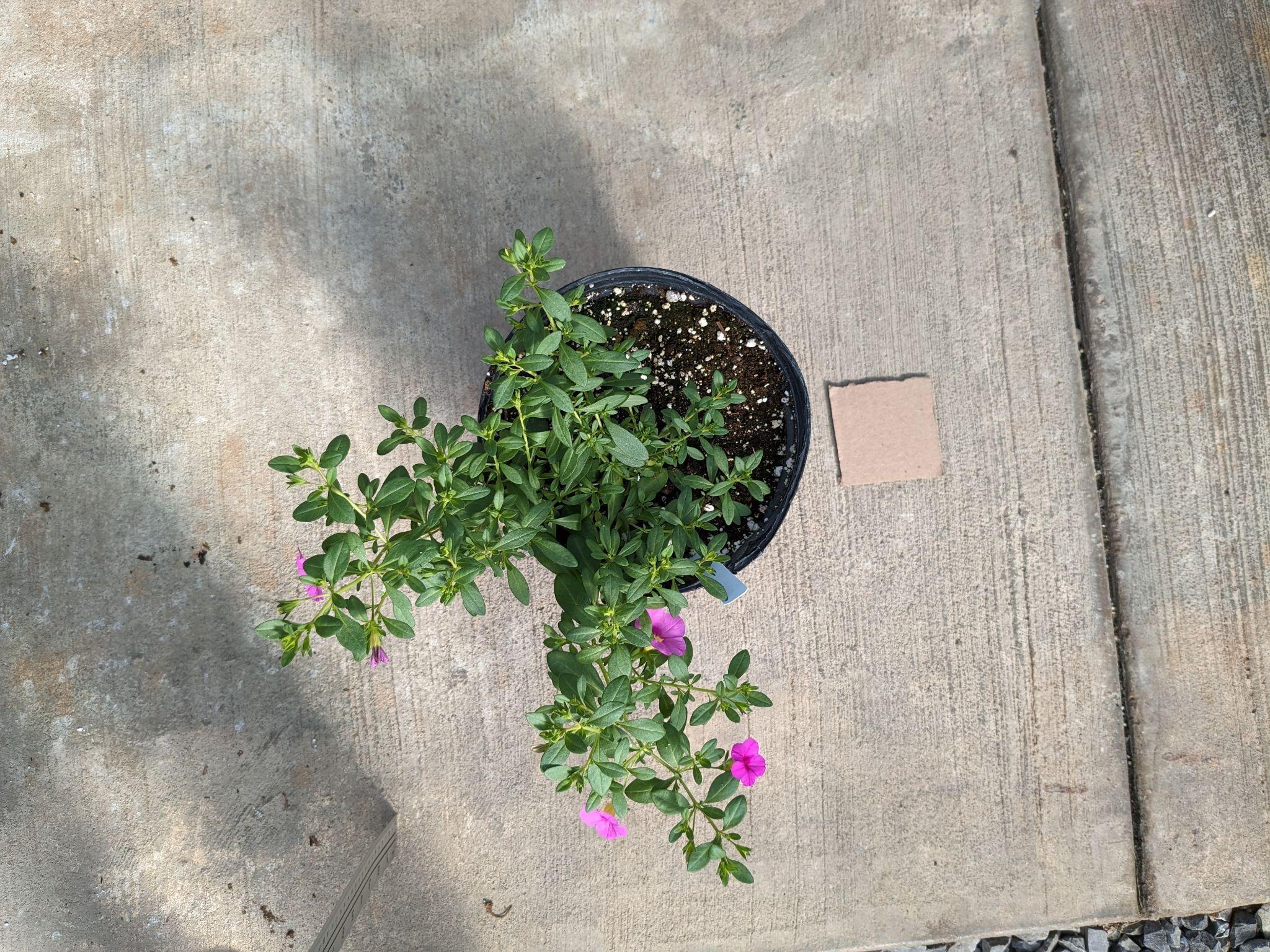 | 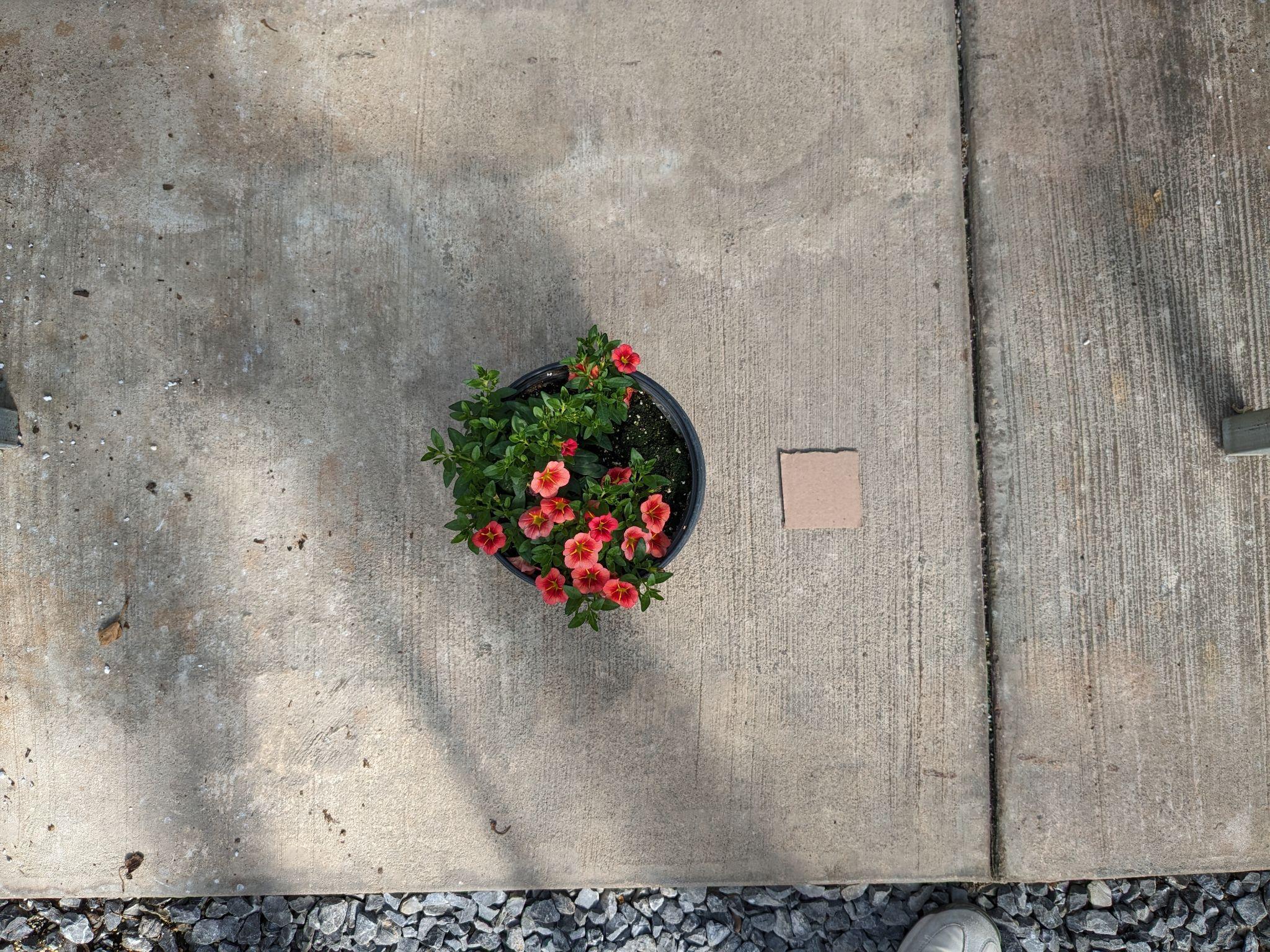 | 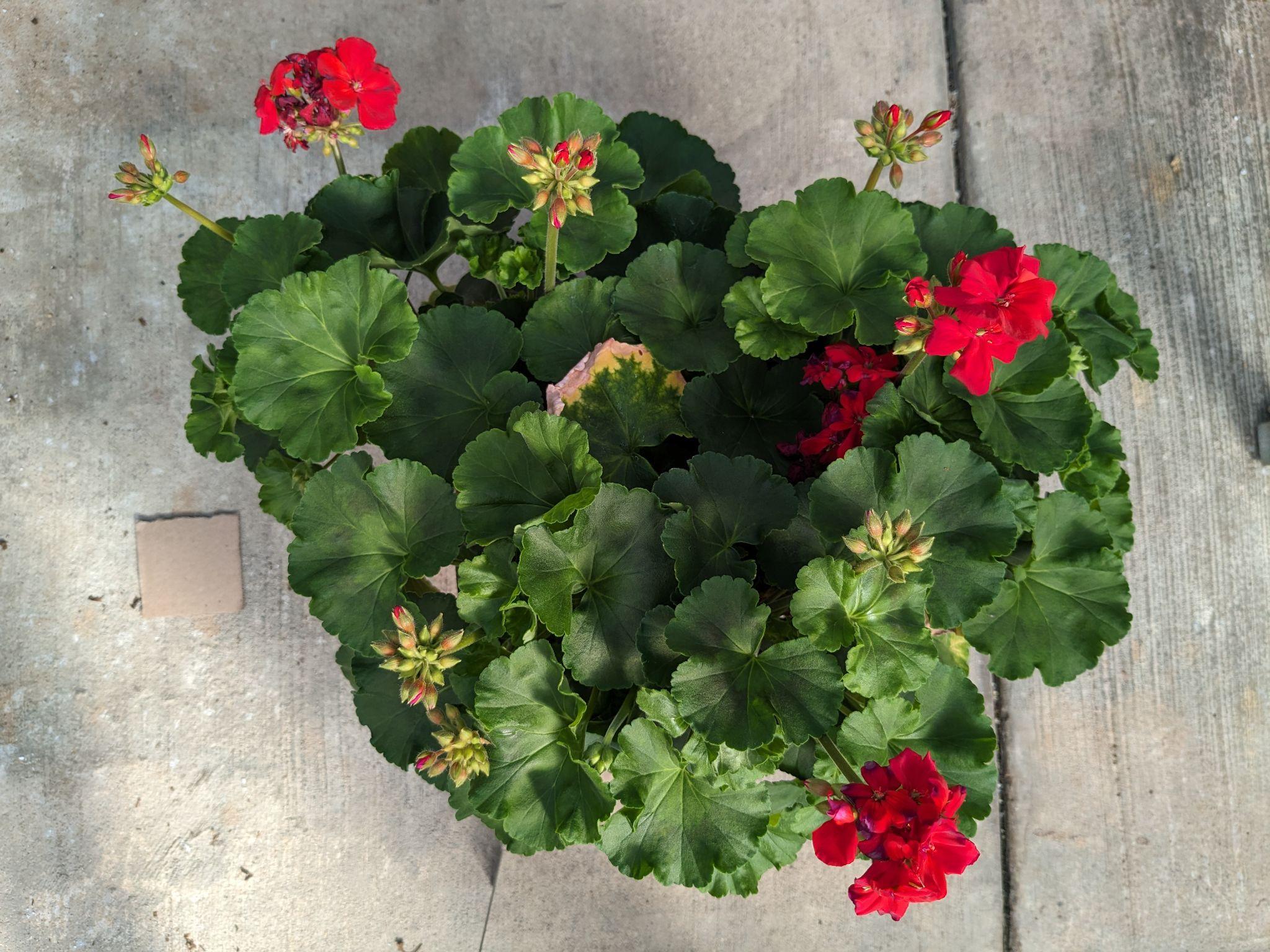 |  |
| 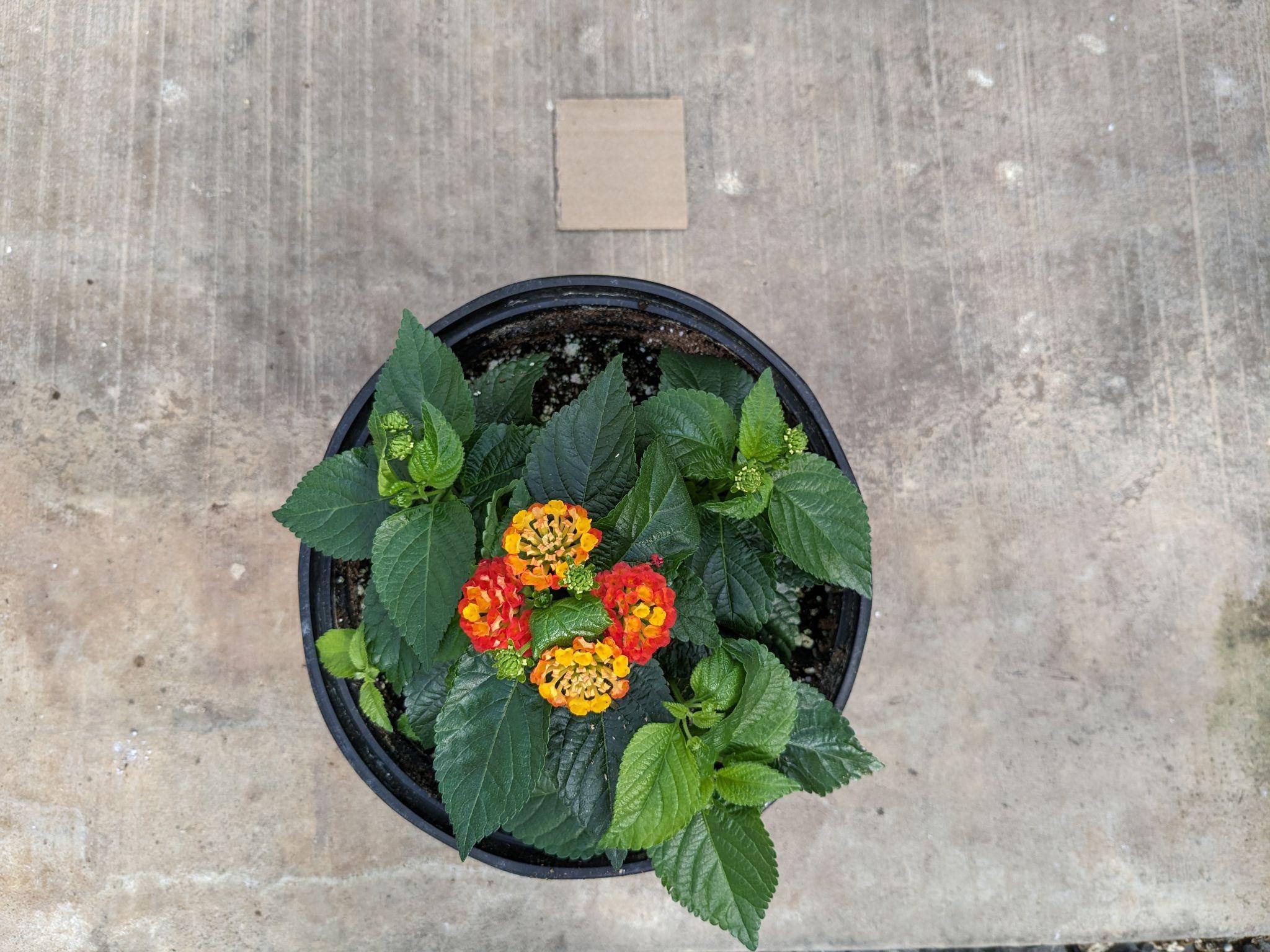 | 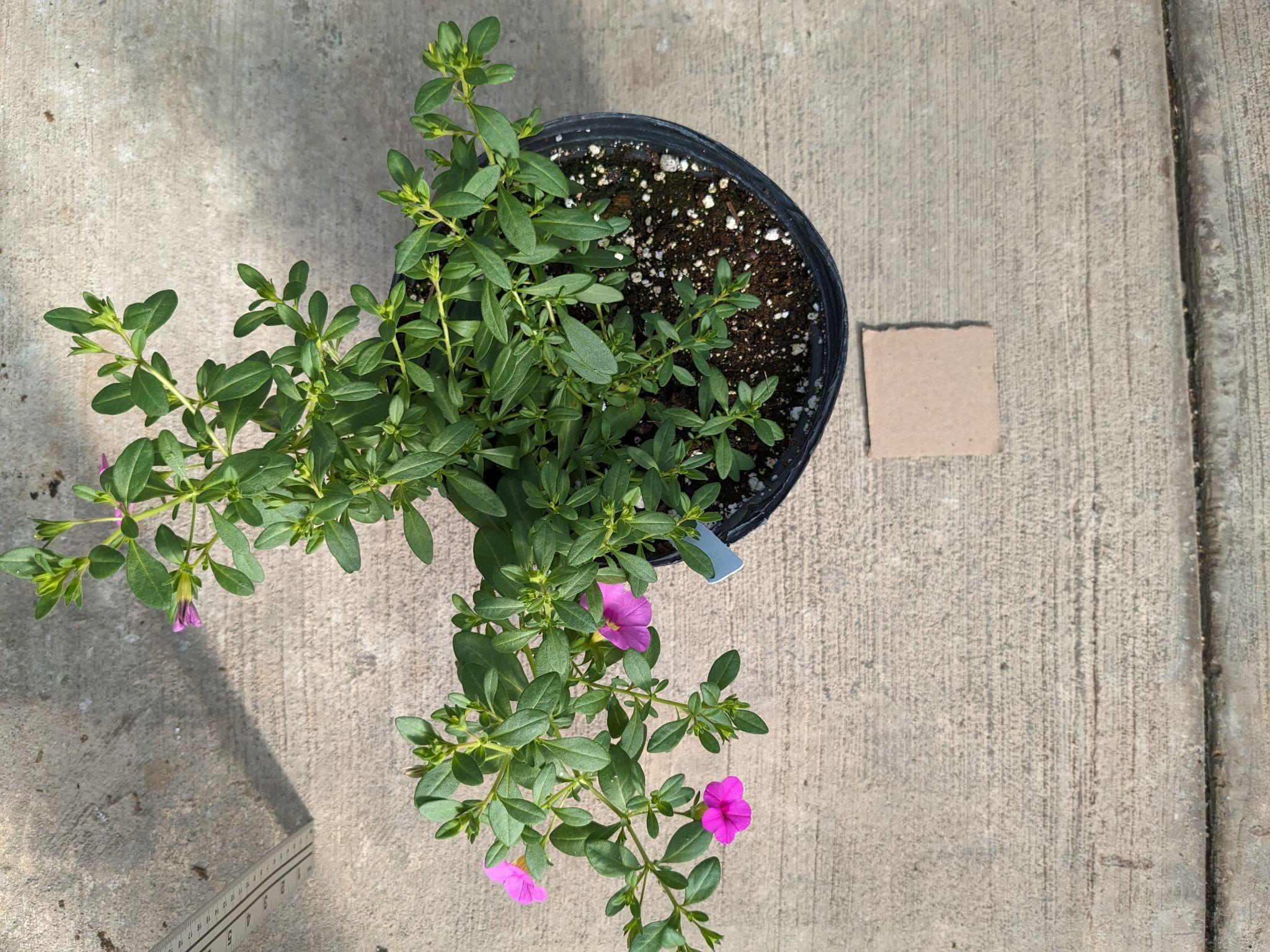 | 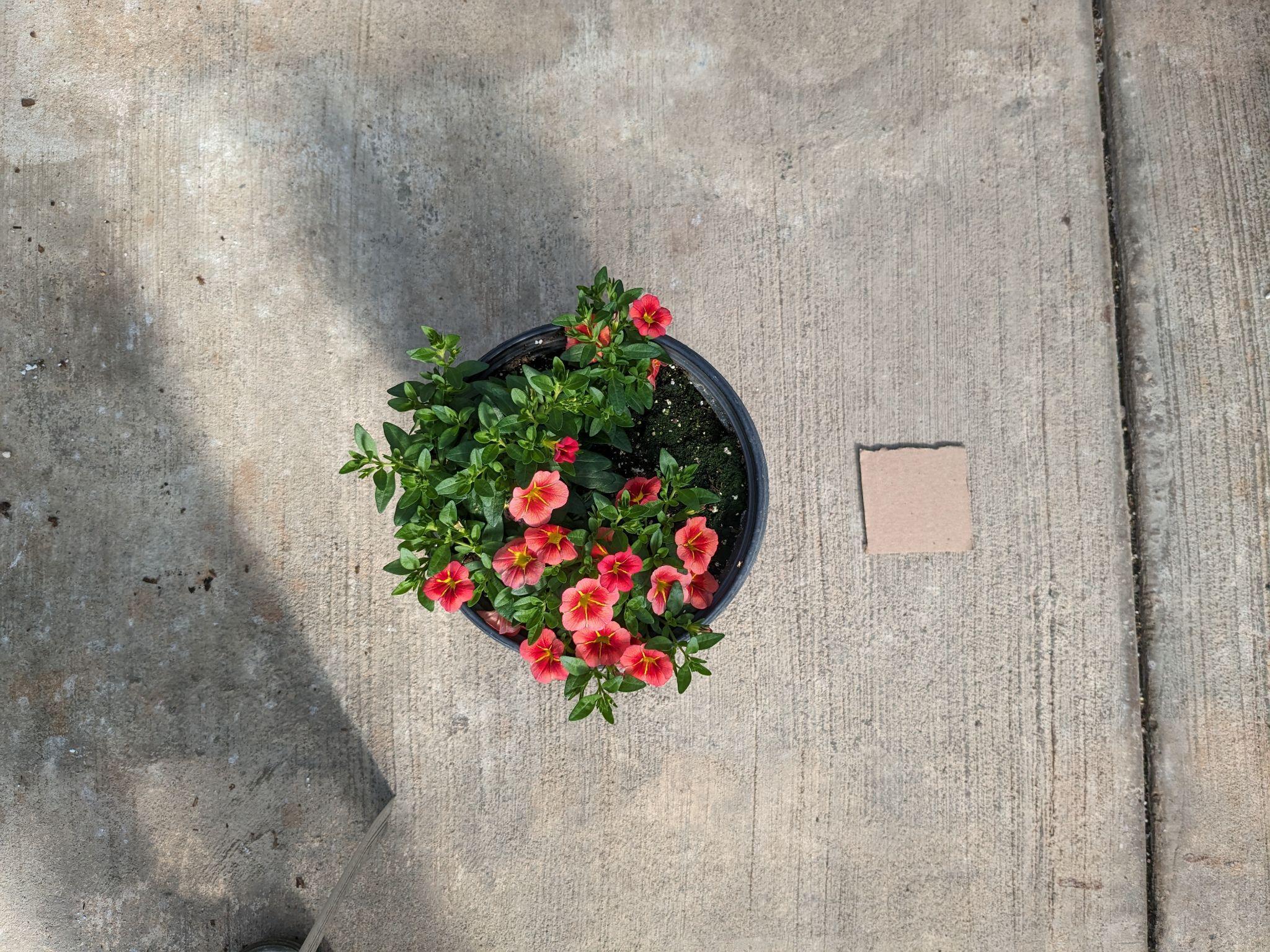 | 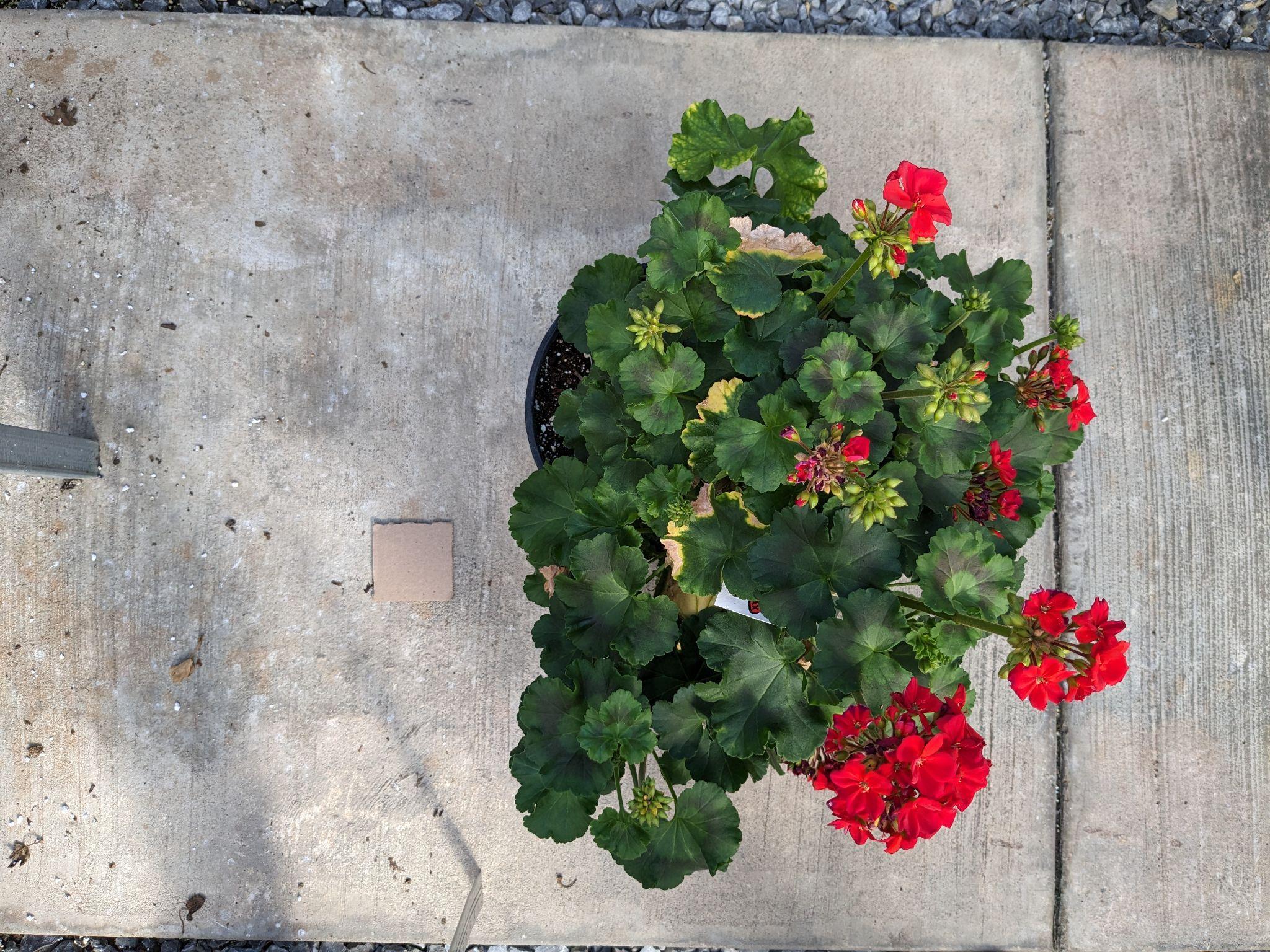 |  |
| 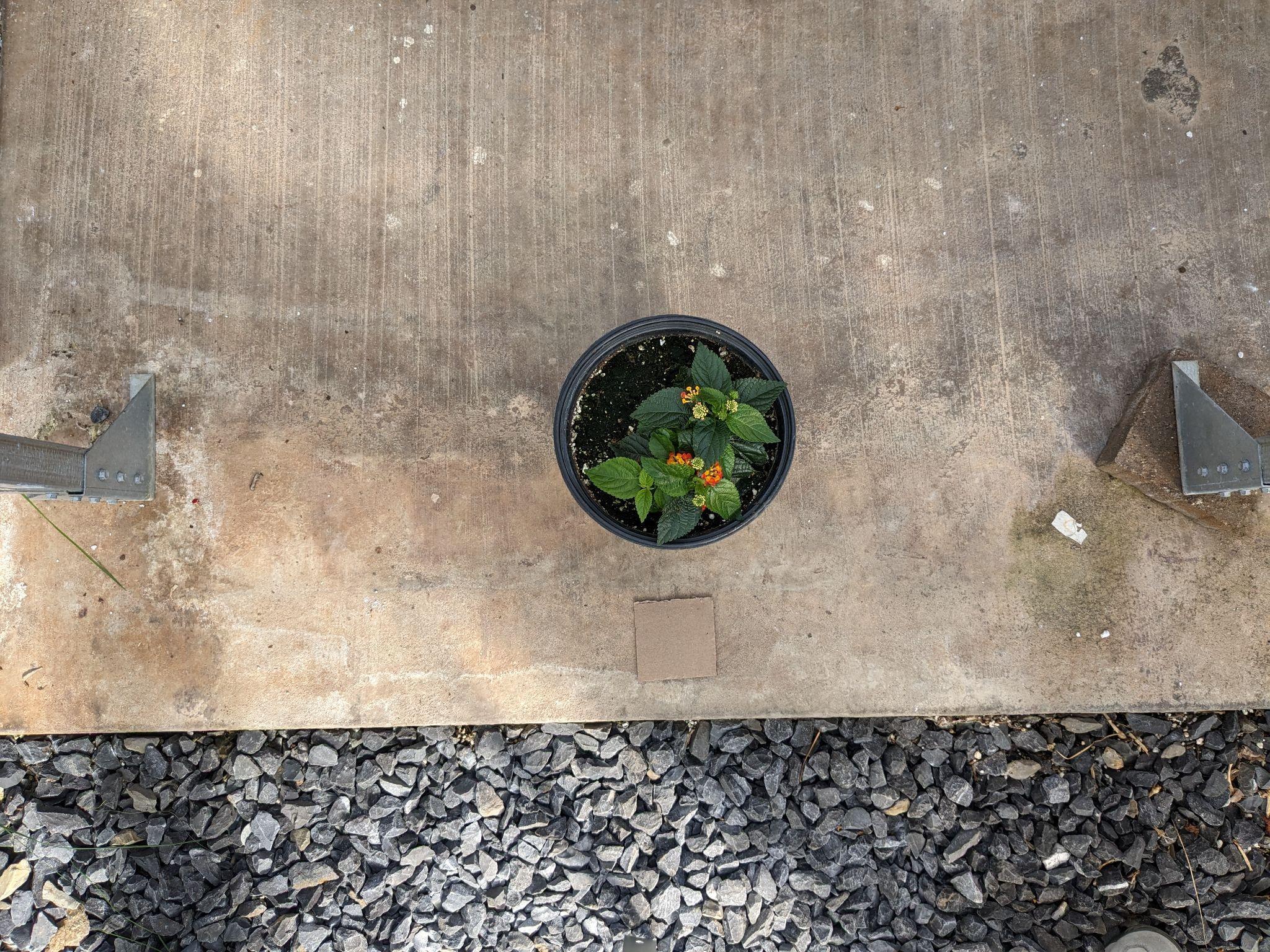 | 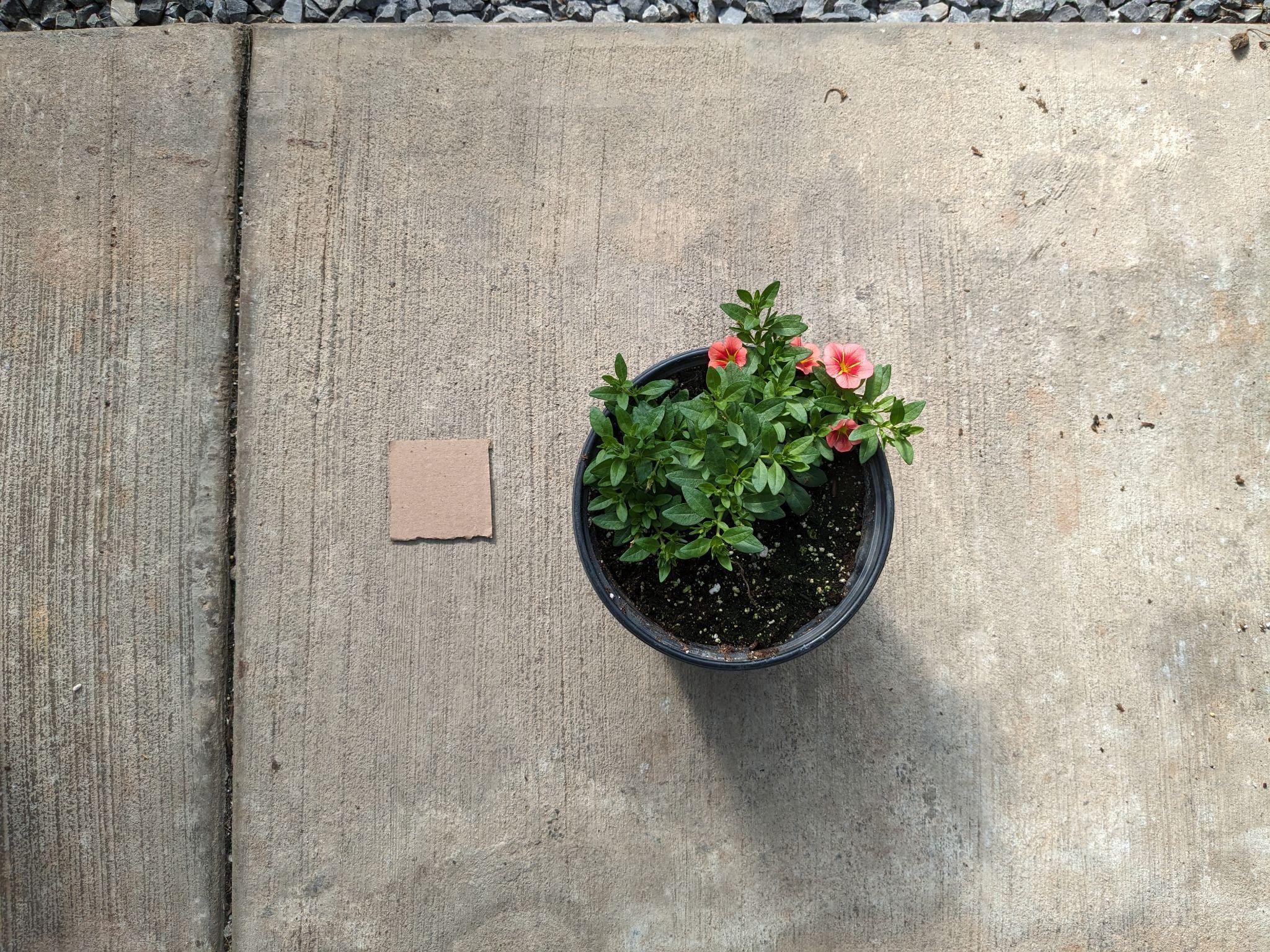 | 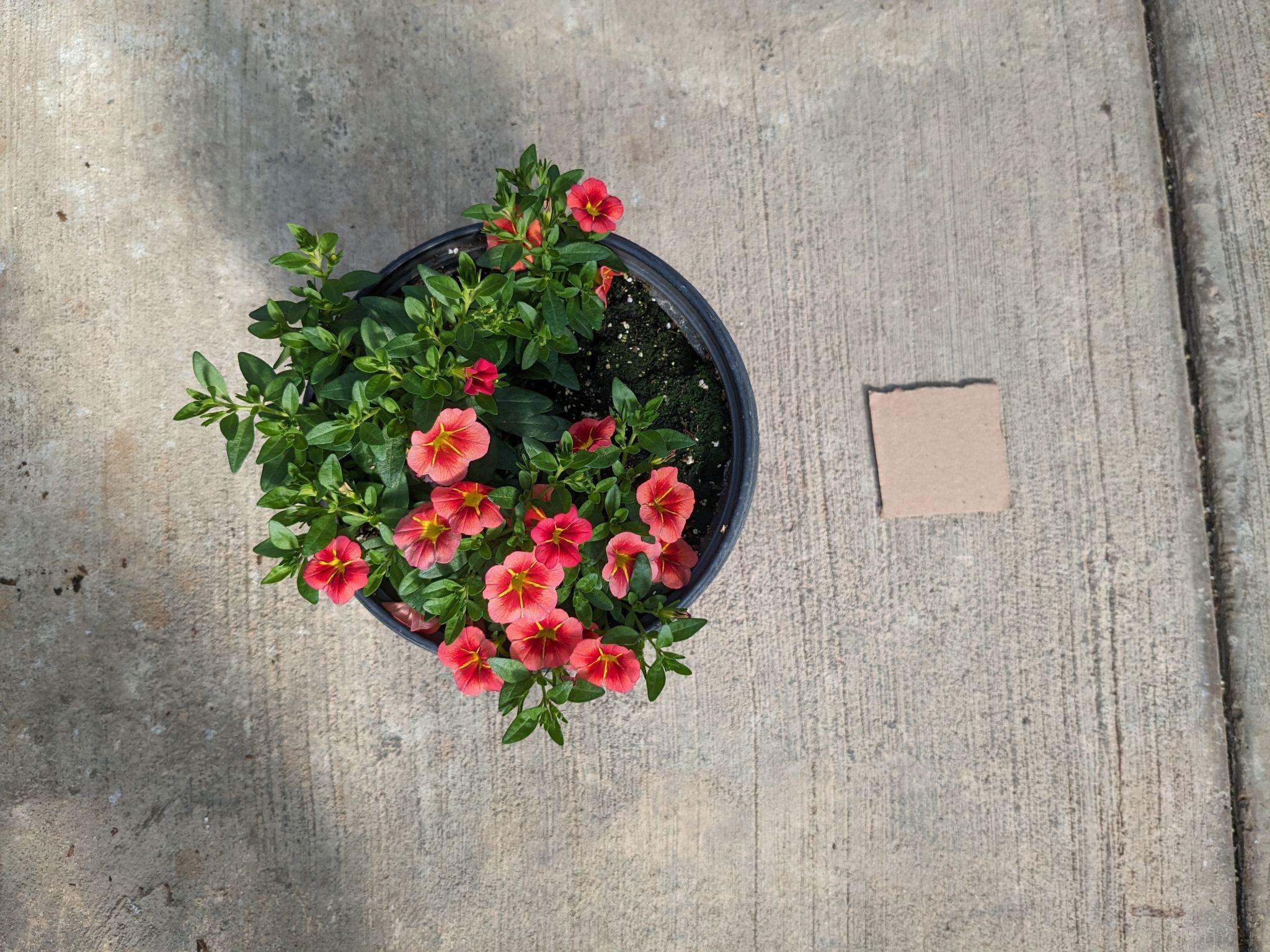 | 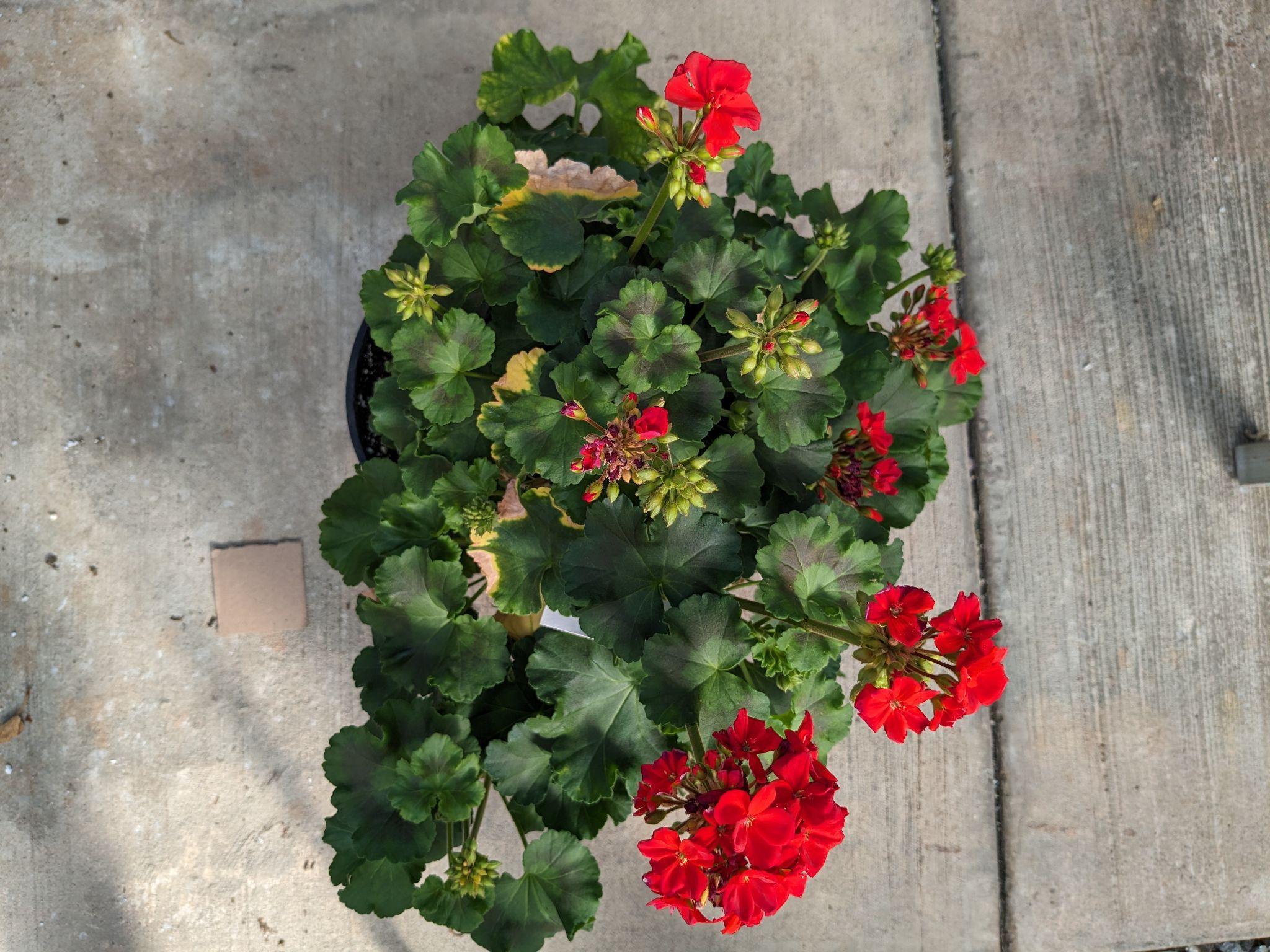 |  |
| 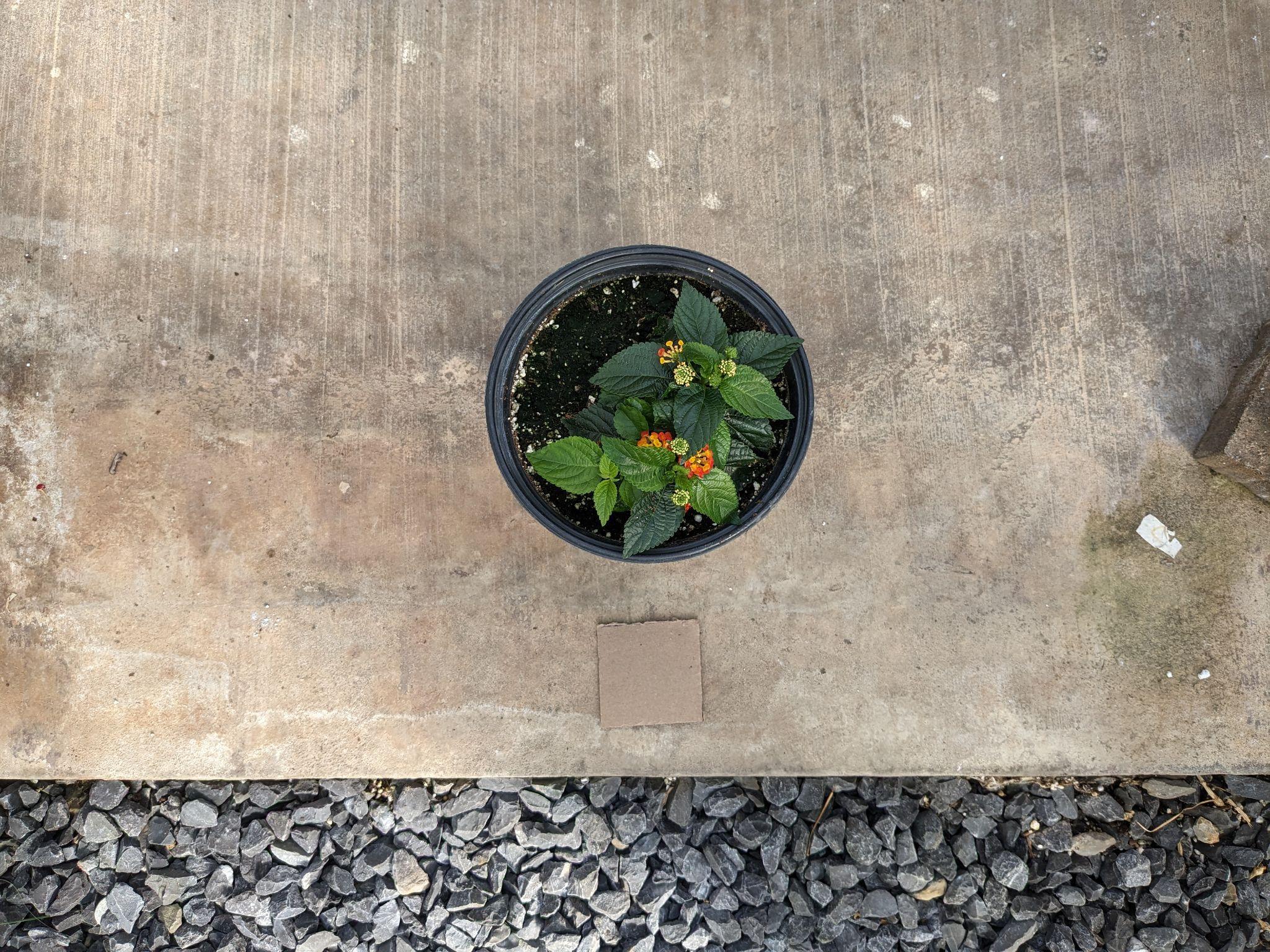 | 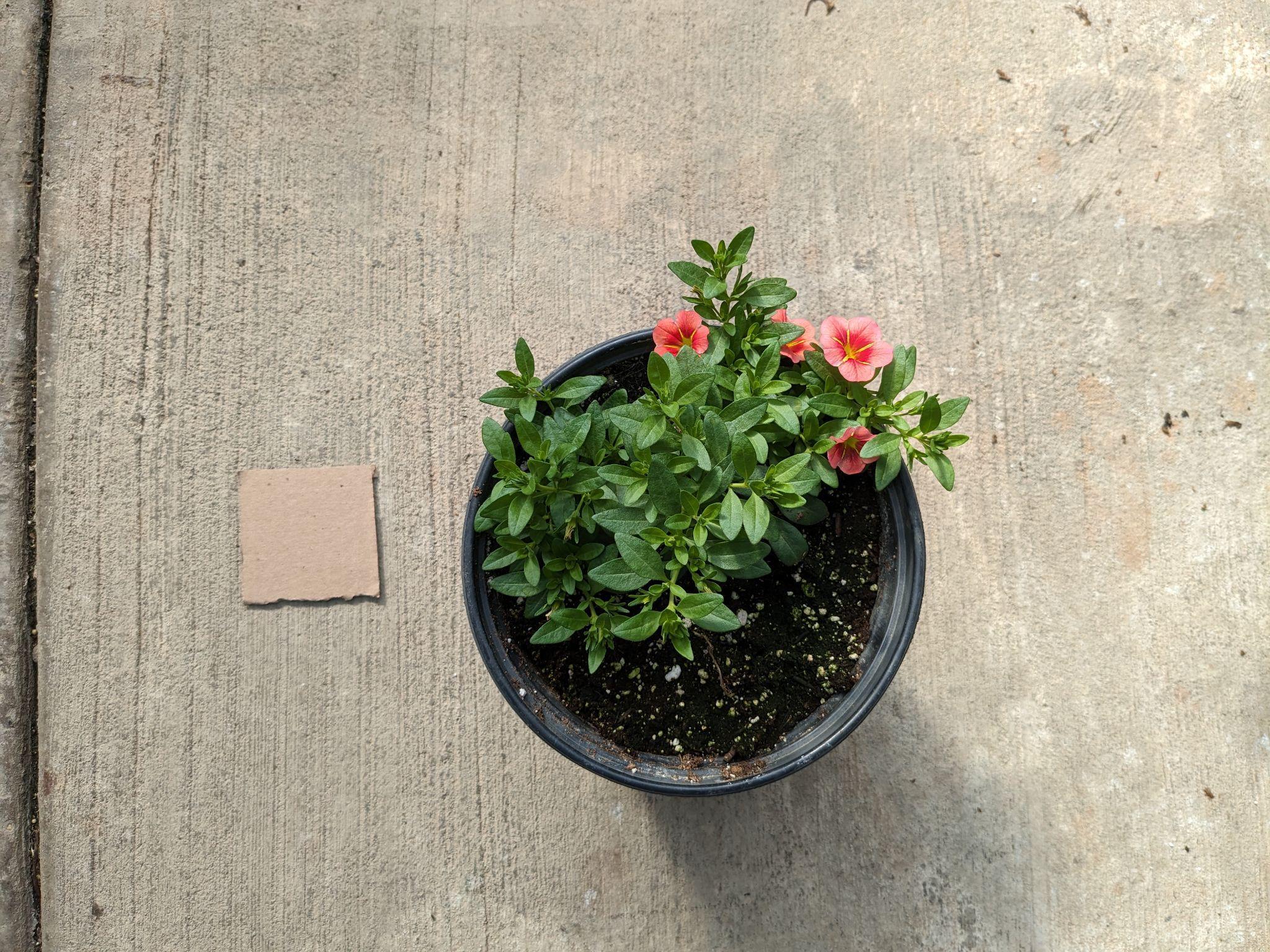 | 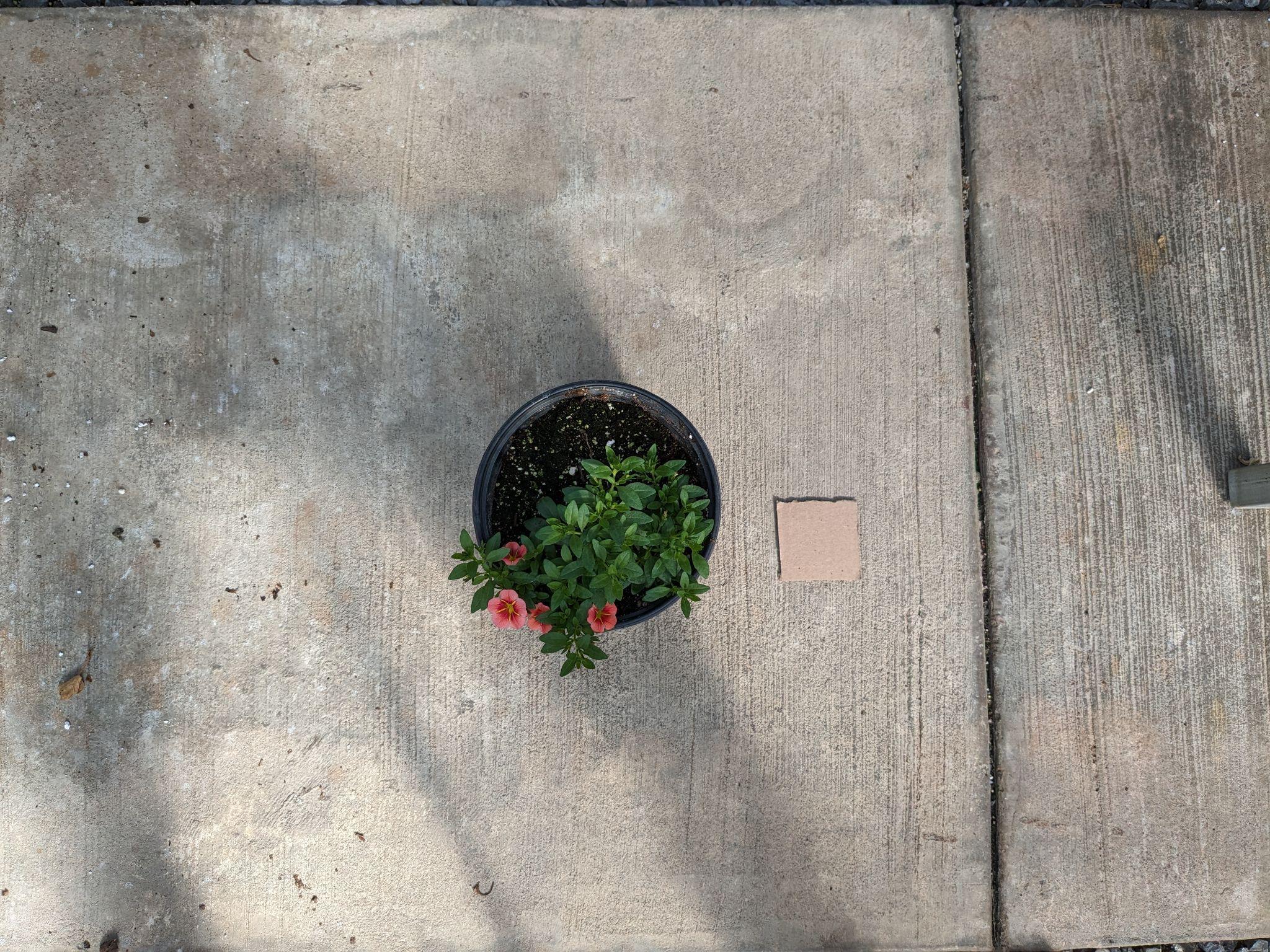 | 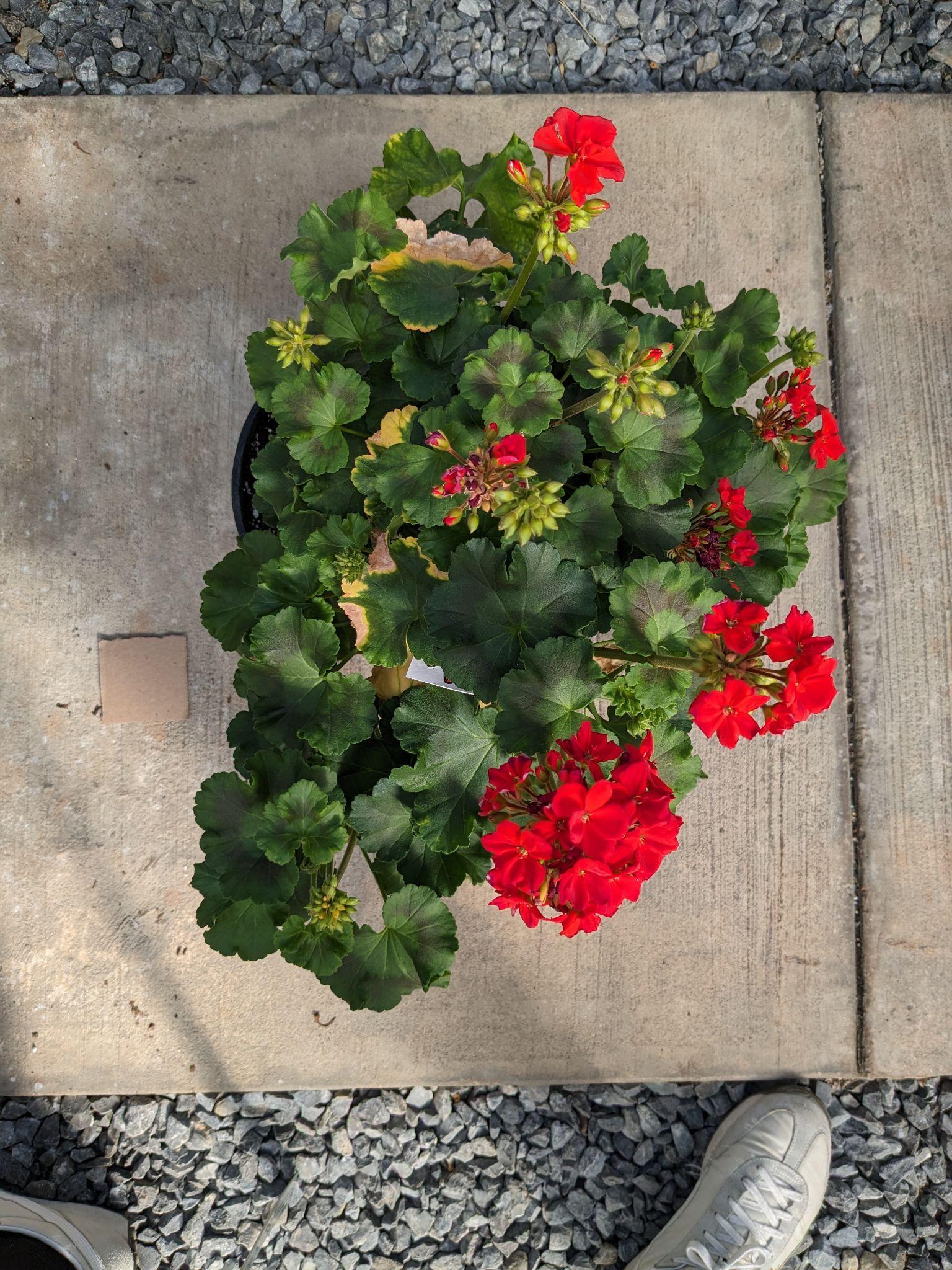 |  |
| 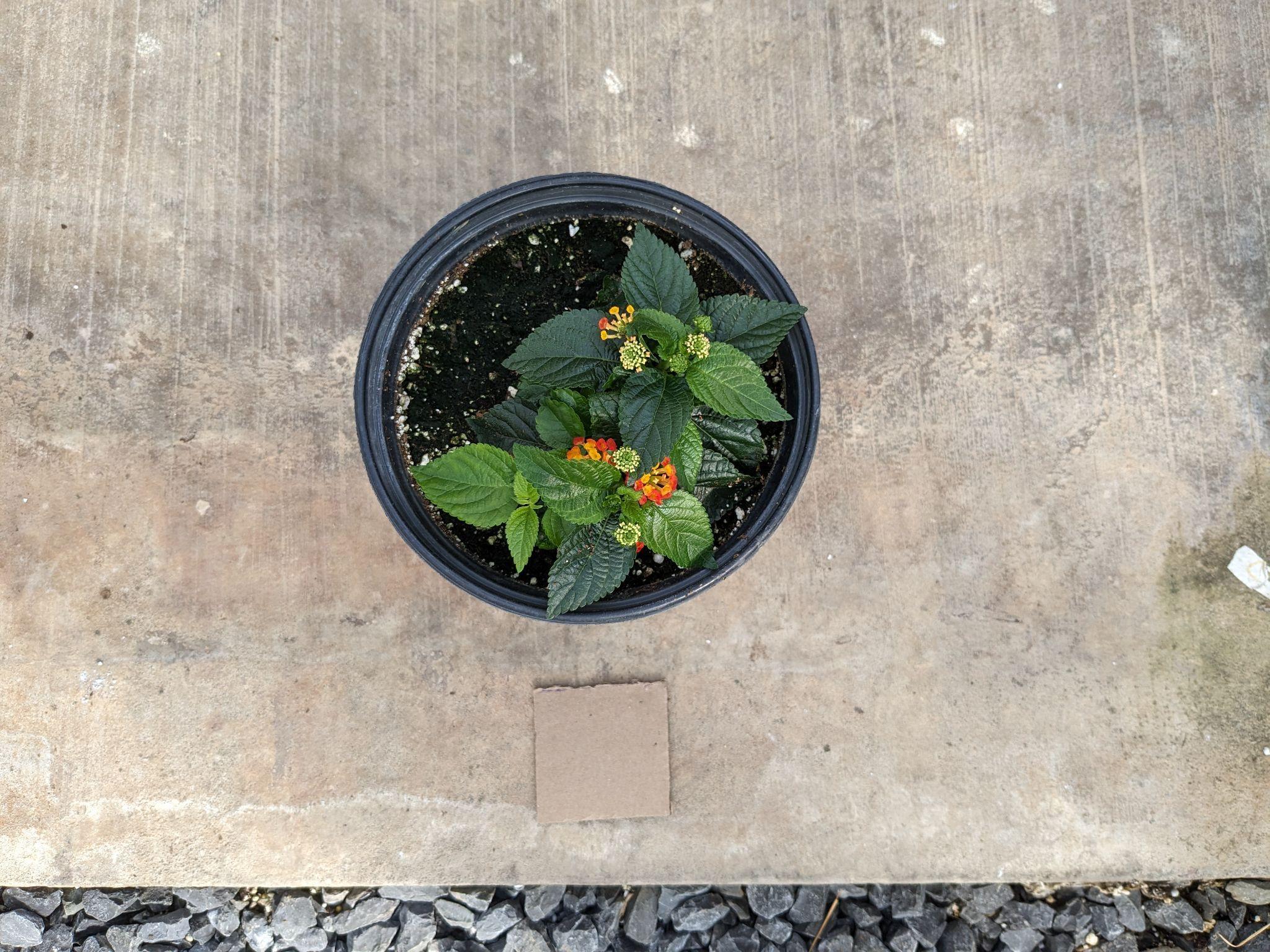 | 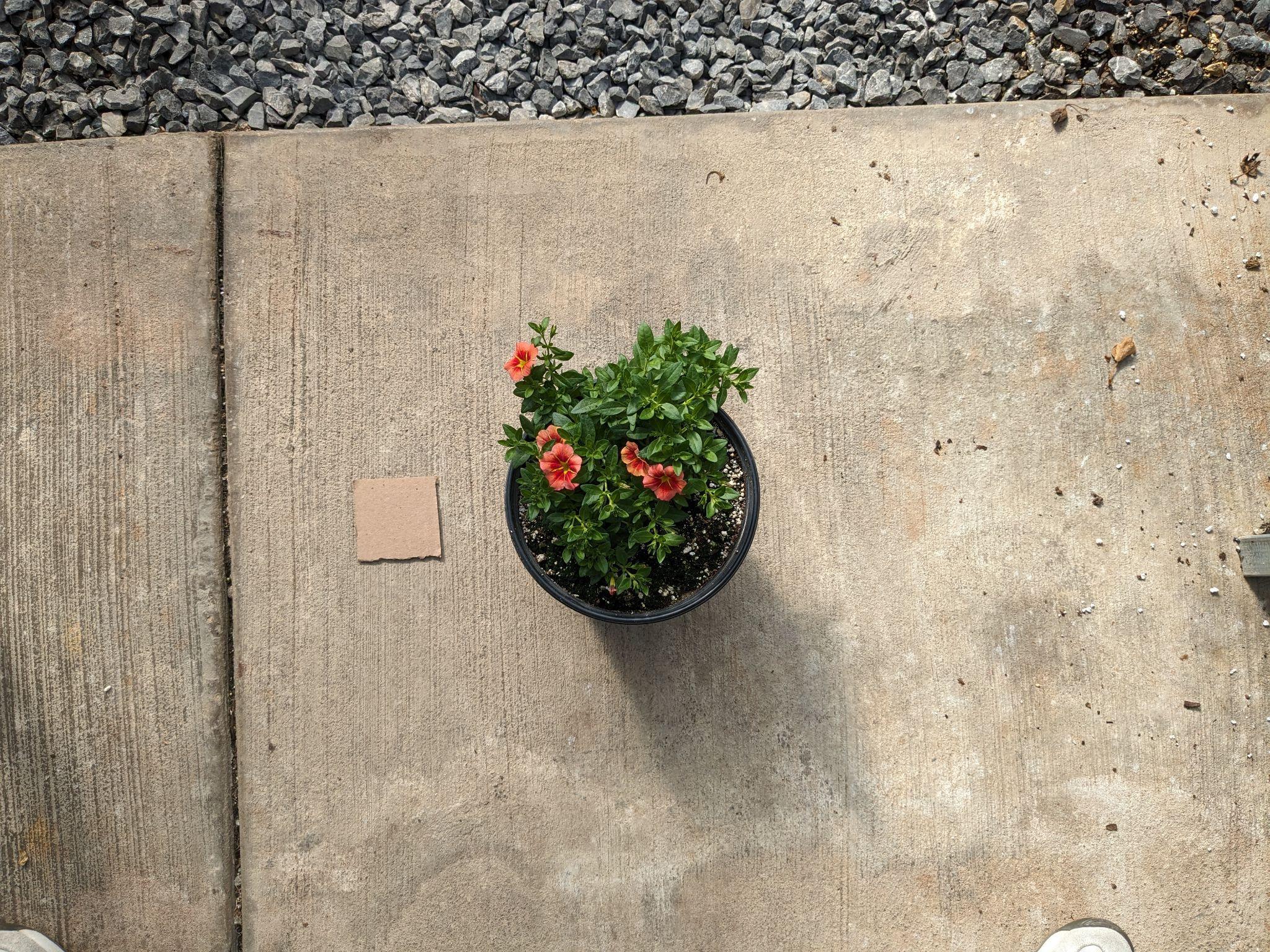 | 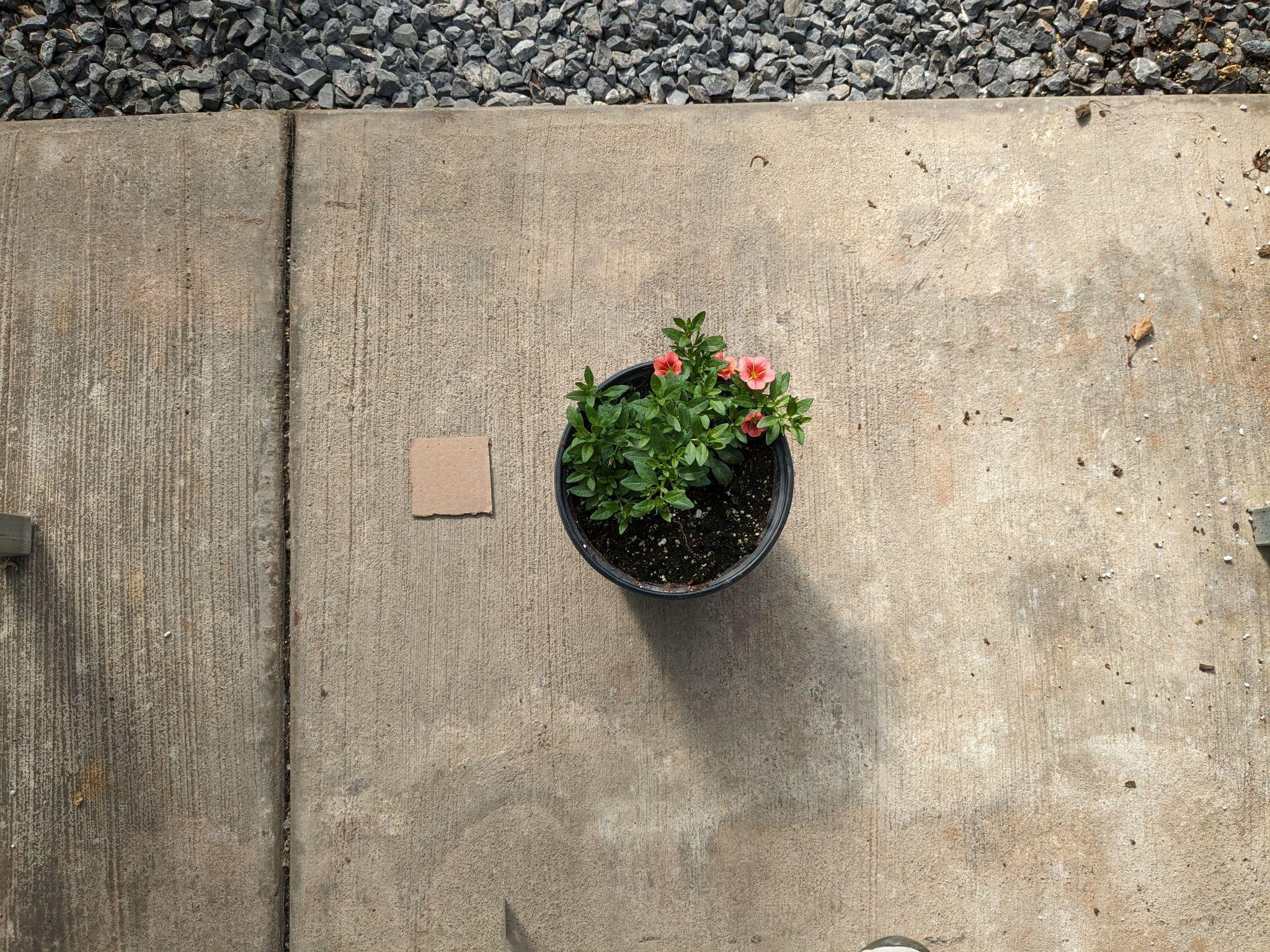 |  |  |
